# Development of a random background to understand ligand optimization

**DOI:** 10.1038/s41586-026-11013-5

**Published:** 2026-09-16

**Authors:** Xinyu Xu, Olivier Mailhot, Galen J. Correy, Xi-Ping Huang, Joao M. Braz, Da Shi, Karthik Srinivasan, Kara Zielinski, Yuliia Holota, Yuliia Kuziv, Christos Iliopoulos-Tsoutsouvas, Nathan D. Levinzon, Yagmur U. Doruk, Moira M. Rachman, Morgan E. Diolaiti, Maisie G. V. Stevens, Fangyu Liu, Katie L. Holland, Harald Hübner, Jing Wang, Yujin Wu, Alan Ashworth, Alexandros Makriyannis, Yuqi Zhang, Yurii S. Moroz, Peter Gmeiner, Robert Abel, Aashish Manglik, Allan I. Basbaum, Bryan L. Roth, James S. Fraser, Brian K. Shoichet

**Affiliations:** 1 Department of Pharmaceutical Chemistry, University of California San Francisco, San Francisco, CA USA; 2 Department of Bioengineering and Therapeutic Sciences, University of California San Francisco, San Francisco, CA USA; 3 Department of Pharmacology, NIMH Psychoactive Drug Screening Program, School of Medicine, University of North Carolina at Chapel Hill, Chapel Hill, NC USA; 4 Department of Anatomy, University of California San Francisco, San Francisco, CA USA; 5 Schrödinger Inc., San Diego, CA USA; 6 Enamine Ltd., Kyiv, Ukraine; 7 Center for Drug Discovery, Department of Pharmaceutical Sciences, Northeastern University, Boston, MA USA; 8 Department of Medicinal Chemistry, College of Pharmacy, University of Utah, Salt Lake City, UT USA; 9 Helen Diller Family Comprehensive Cancer Center, University of California, San Francisco, CA USA; 10 Department of Chemistry and Pharmacy, Medicinal Chemistry, Friedrich-Alexander-Universität Erlangen-Nürnberg, Erlangen, Germany; 11 Chemspace LLC, Kyiv, Ukraine; 12 Taras Shevchenko National University of Kyiv, Kyiv, Ukraine; 13 Schrödinger Inc., New York, NY USA

**Keywords:** Medicinal chemistry, Chemical modification

## Abstract

Ligand optimization is central to drug discovery, with hundreds of analogues often designed and synthesized between an initial hit and a therapeutic candidate^1,2^. The efficiency of this process is unclear, partly because there is no random background for optimization to compare against. Such a random background might emerge from systematic random small substitutions across starting ligands, measuring the likelihood of achieving a substantial improvement in affinity or potency, or other property by any single perturbation. Recent literature has suggested that perhaps 10% of analogues with minor modifications improve upon the potency of a parent by tenfold or more^3,4^, but this number is clouded by reporting bias, intentional improvement and inter-group variability. To begin to establish a background expectation for ligand optimization, here we systematically modified 18 lead molecules across six targets with single-atom changes; 257 compounds were synthesized. Unexpectedly, 11.3% of these random small perturbation analogues improved potency by tenfold or more. Conversely, they typically had worse in vitro pharmacokinetics. Although it was possible to find analogues where the potency increase compensated for inferior exposure and half-life, resulting in more potent compounds in vivo, overall, a frustrated landscape for ligand optimization is revealed. This study begins to establish a background expectation for ligand potency optimization and offers a simple strategy to do so. It also begins to quantify the challenges confronting the field in moving beyond in vitro potency.

## Abstract (web summary)

An approach to improve efficiency and establish a background expectation for ligand potency optimization is described.

## Main

**Extended Data Fig. 1:**
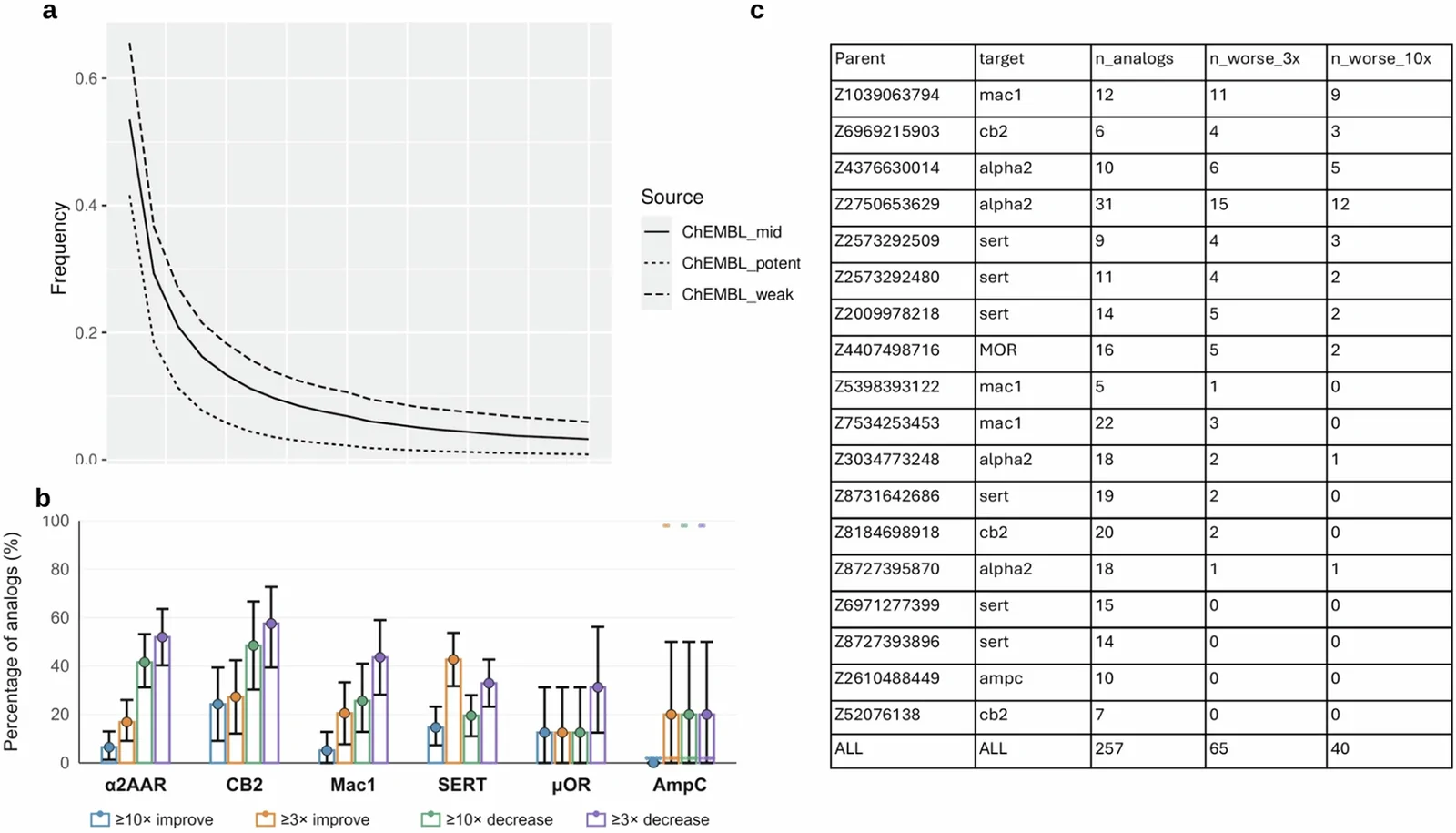
Frequency of activity changes from single-atom substitutions in ChEMBL and in this study. a, Cumulative frequency of single non-hydrogen atom substitutions (CH_3_, F, Cl, Br, N and OH) in ChEMBL that improve activity, stratified by that of the parent compound. “Potent” denotes parents with activity ≤32 nM, “mid” 32 nM–1 μM, and “weak” 1 μM–1 mM. b, For each target in this study, the percentage of analogs that improve or decrease activity by ≥3-fold or ≥10-fold versus their parent. Open bars and points show observed percentages; black error bars show two-sided 95% confidence intervals estimated by percentile bootstrap (20,000 resamples). Sample sizes were n = 77, 33, 39, 82, 16 and 10 analogs for alpha2AAR, CB2, Mac1, SERT, μOR and AmpC, respectively. Individual binary outcomes are overlaid for AmpC (n = 10). Fold changes were rounded to the nearest integer before thresholding (for example, 9.6–9.9 counted as 10-fold). Compounds yielding no measurable curves were counted as ≥10-fold decreases. c, Parent-level summary of analog effects in this study (rounded fold-change). Source data

Ligand optimization is central to chemical probe and drug discovery, as initial active molecules rarely have the potency or pharmacokinetic (PK) properties to be viable in vivo^1,2^. Between the discovery of an initial hit and a clinical candidate many hundreds, occasionally thousands of optimized analogues might be synthesized^1,5^. Several strategies^6–10^ have been developed to improve this process, ranging from early empirical approaches such as Topliss Trees^11^ and quantitative structure–activity relationships^12^ to contemporary artificial intelligence (AI)-guided PK^13^ prediction and free energy calculations^14,15^. The efficiency of these strategies remains uncertain as there is no random background against which to compare them. Using tenfold improvement between analogue and parent as a benchmark for substantial impact, 10% of analogues meet this standard in the ChEMBL database^16^ (Extended Data Fig. 1a). As these ChEMBL results suffer from success bias, sample multiple types of perturbation and suffer from inter-group irreproducibility^17^, the public domain offers no sure guidance on what level of improvement one might expect in ligand optimization from random conservative changes.

Random backgrounds have long been used in biology to quantify significance. In genomics, they distinguish between artificial and natural selection^18^. In epidemiology, random incidence distinguishes genetic diseases from those driven by environmental factors^19,20^. In protein engineering, random backgrounds help to evaluate improvements in enzyme function^21^, and alanine scanning introduces a minimal perturbation to find hot spots for binding and function^22^. A random background for ligand optimization might quantify progress across chemotypes and targets and compare different optimization strategies.

Like alanine scanning, an unbiased background for ligand optimization might involve conservative perturbations, systematically and comprehensively applied across multiple parents with different targets. This resembles a ‘positional analogue scanning’ approach that substitutes ligand non-polar hydrogens with groups such as CH_3_, OH, Cl, F and Br, and aromatic carbon atoms with nitrogen^23^. In a retrospective analysis of 110,000 matched molecular pairs in the ChEMBL24 database^3,4^, about 30% of analogues improved over threefold in affinity or potency versus their parent. This observation is tempered by design and reporting biases towards compound improvement, by the variability in experimental conditions across datasets that can reduce reproducibility^17^, and by the few parent compounds that had multiple conservative changes. Thus, it seemed interesting to create a set of parent ligands that were systematically and comprehensively modified by small perturbations, without design and without a bias towards improvement, using the positional analogue scanning approach^23,24^ where the differences between parent and the suite of analogues were quantified by a single laboratory.

**Fig. 1:**
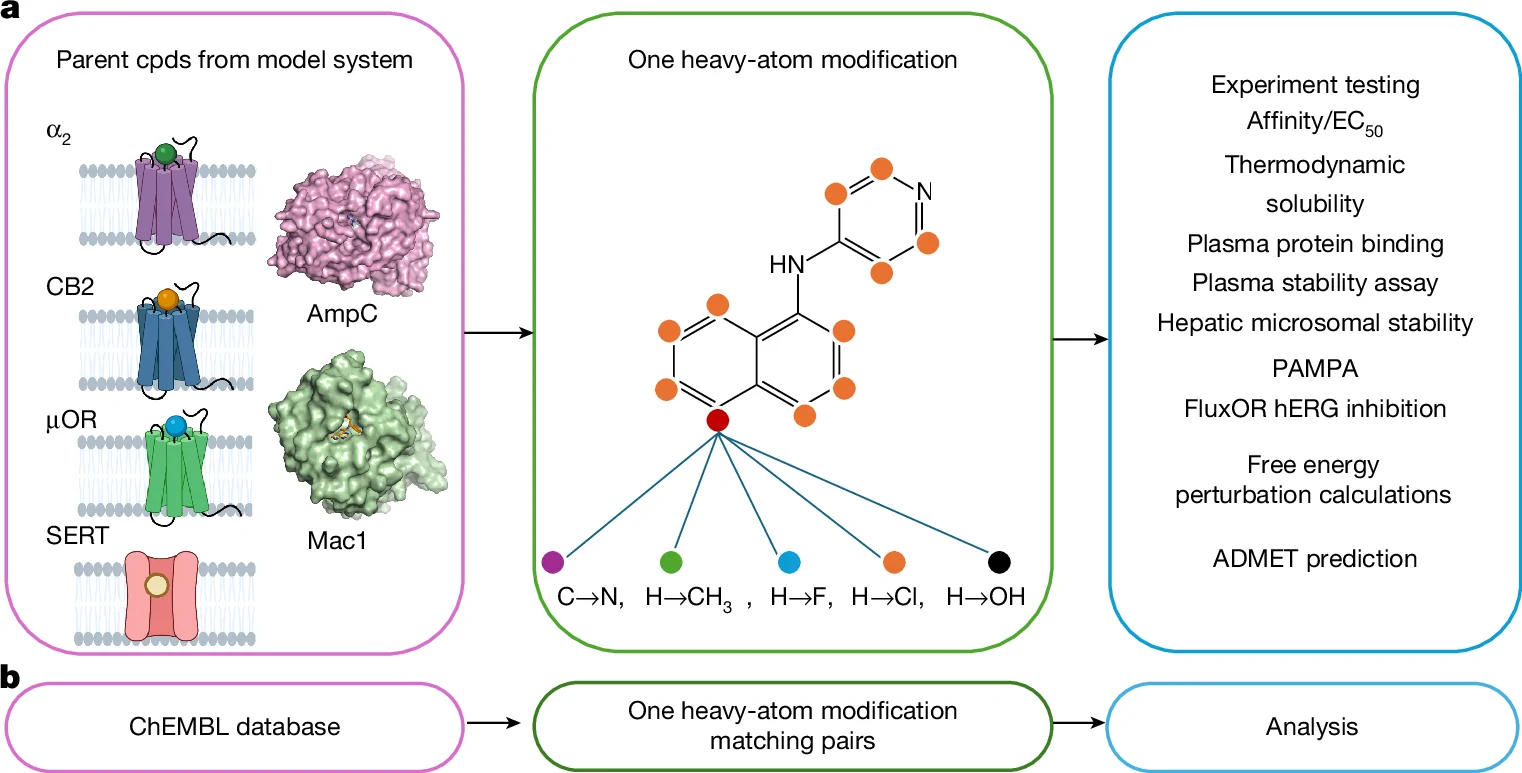
Experimental and analysis workflow for one heavy-atom analogues. **a**, Parent ligands from six drug targets (the aminergic GPCR α_2_AAR, the lipid GPCR CB2R, the peptide GPCR µOR, the serotonin SLC transporter SERT, and the soluble enzymes AmpC and Mac1) were modified by one-at-a-time, one-atom substitutions: H→CH_3_, H→Cl, H→OH, H→F and ring C→N. These modifications were made systematically and comprehensively; only compounds that were too expensive to synthesize or that changed the charge state of the parent were left out. The analogues were tested for changes in affinity or potency, solubility, plasma protein binding, plasma stability, hepatic microsomal stability, permeability and hERG inhibition, versus their parent ligands. Relative affinity and in vitro PK changes were also evaluated computationally using FEP+ and ADMET prediction models. cpd, compound. **b**, For comparison with the experimental set, matched parent–analogue pairs differing by one heavy-atom modification were identified in the ChEMBL database and analysed using the same activity-improvement framework. Figure created in BioRender; Xu, X. https://biorender.com/b6zqsv7 (2026).

**Extended Data Fig. 2:**
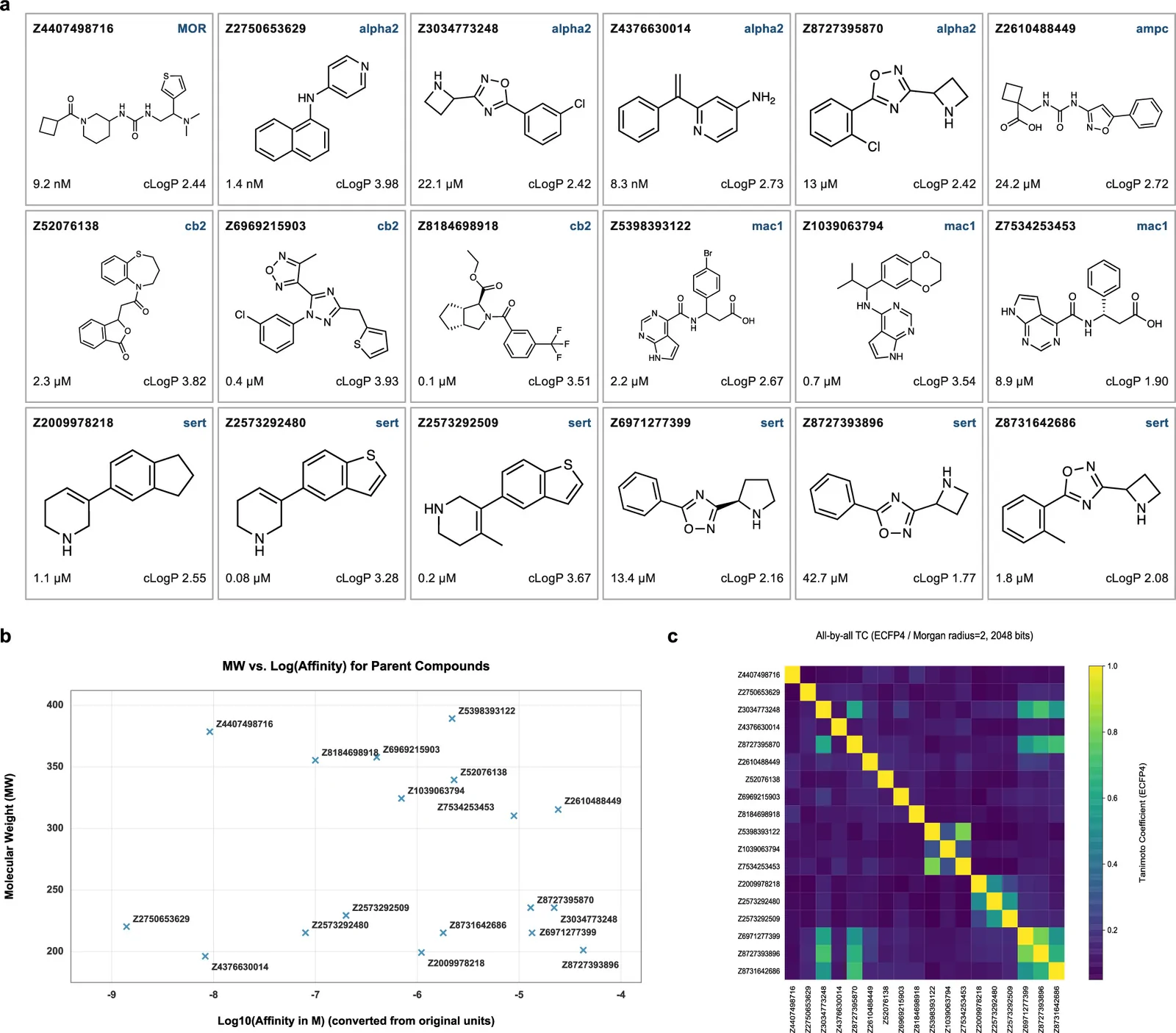
Overview of Parent Compounds’ Structures and Properties. a, 2D structures of the parent compounds, with their measured affinity or potency and calculated cLogP values. b, Molecular Weight (MW) and Affinity or potency values for the parent compounds. c, Structural Similarity matrix showing the relationships between parent compounds based on molecular fingerprint comparisons. Source data

To create what we refer to as a random background set for ligand optimization, we chose 18 parent ligands spanning six targets, including three GPCRs (α_2_A adrenergic receptor (α_2_AAR), μ-opioid receptor (μOR) and cannabinoid receptor type 2 (CB2)), one transporter (the serotonin transporter (SERT)) and two soluble enzymes (AmpC β-lactamase (AmpC) and macrodomain 1 of SARS-CoV-2 (Mac1)). This set was chosen for targets that we had under experimental control and is admittedly far from comprehensive. The 18 parents were chosen for similar pragmatic reasons; they were molecules to which we had ready access. Still, these proteins provide a range of target classes while the 18 parents cover a wide range of physicochemical properties (Extended Data Fig. 2): ranging from 1 nM to 43 µM activity (Table 1) and representing 11 unrelated scaffolds. For each parent, we systematically explored all analogues accessible by a single-atom or methyl modification where such modifications were synthetically feasible and where they did not modify the net charge of the parent at physiological pH. The single-atom modifications involved substituting carbon hydrogens with one of methyl^25^, hydroxyl^26^, chloro^27^, fluoro^28^ or rarely bromo, or substituting aromatic carbon atoms with nitrogens (Fig. 1). These modifications are not comprehensive but do represent those preferred by medicinal chemists.

**Table 1:** Analogues from all parents improving affinity or potency by threefold or more or tenfold or more

Protein	Assay readout	Parent ligand	Parent affinity or potency	Total analogues	Analogues with threefold or more affinity or potency improvement	Analogues with tenfold or more affinity or potency improvement
μOR	EC_50_	Z4407498716	9.2 nM	16	2	2
α_2_A	EC_50_	Z2750653629	1.4 nM	31	7	4^a^
α_2_A	*K*_i_	Z3034773248	22.1 µM	18	3	1
α_2_A	EC_50_	Z4376630014	8.3 nM	10	2	0
α_2_A	*K*_i_	Z8727395870	13 µM	18	1	0
AmpC	*K*_i_	Z2610488449	24.2 µM	10	2	0
CB2	*K*_i_	Z52076138	2.3 µM	7	7	6^b^
CB2	*K*_i_	Z6969215903	0.4 µM	6	0	0
CB2	EC_5__0_	Z8184698918	0.1 µM	20	2	2
Mac1	IC_50_	Z5398393122	2.2 µM	5	0	0
Mac1	IC_50_	Z1039063794	0.7 µM	12	0	0
Mac1	IC_50_	Z7534253453	8.9 µM	22	8	2
SERT	*K*_i_	Z2009978218	1.1 µM	14	4	0
SERT	*K*_i_	Z2573292480	0.08 µM	11	1	0
SERT	*K*_i_	Z2573292509	0.2 µM	9	2	1
SERT	*K*_i_	Z6971277399	13.4 µM	15	10	4
SERT	*K*_i_	Z8727393896	42.7 µM	14	8	3^c^
SERT	*K*_i_	Z8731642686	1.8 µM	19	10	4
Total		All parents		257	69	29

Fold changes are displayed as nearest-integer values; exact (unrounded) fold changes are provided in Supplementary Table 1. Using exact fold changes, 62 analogues improve threefold or more and 25 analogues improve tenfold or more. ^a^One of these had a fold change of 9.7. ^b^Two of these had fold changes of 9.7 and 9.8. ^c^One of these had a fold change of 9.9 (see Supplementary Table 1).

These substitutions were systematically and comprehensively applied without design, ensuring an approach unbiased by anything other than synthetic accessibility and price. Overall, 257 single-atom analogues were synthesized and tested for changes in activity on the target, allowing us to measure how often we might expect affinity or potency to improve tenfold or more by minimal perturbation. This begins to provide a background expectation for how often a designed analogue might meaningfully improve affinity or potency. As we show, these undesigned changes had an unexpectedly high success rate, suggesting a systematic strategy for optimization, even though that was not our goal. As affinity or potency maturation is only one criterion for advancing from hit to candidate, we also measured in vitro PK properties for the analogues, including permeability, metabolic stability, plasma protein binding and three other terms, versus the parent compounds. These studies explore trade-offs between PK and target affinity or potency in ligand optimization.

## Systematic small-change analogues

We began with 18 parent ligands, most of which are unrelated to one another topologically—that is, by their patterns of atoms and bonds (ECFP4 Tanimoto coefficients ≤ 0.35), with affinities ranging from 1 nM to 43 µM and molecular weights ranging from 200 to 400 amu (Extended Data Fig. 2). Each of the 18 parents was modified one atom at a time with five types of single-atom substitutions (Fig. 1a, central panel), subject only to expense constraints (all molecules cost US$400 or less). About 7% of the possible molecules met these criteria and of those approximately 80% were successfully synthesized, resulting in 257 analogues. In the analyses that follow, we pooled all 257 analogues for analysis, improving statistical power (Supplementary Fig. 1).

## Affinity or potency changes between parents and the small-change analogues

The 257 analogues were tested for activity changes (dissociation constant (*K*_d_), half-maximal inhibitory concentration (IC_50_) or half-maximal effective concentration (EC_50_)) versus the parents across the six targets. For μOR, EC_50_ values were determined in cAMP Glo-sensor assays. α_2_A and CB2 ligands were assessed by cAMP assays and radioligand competition binding, while SERT activity was assessed using a transporter uptake assay. AmpC inhibition was measured enzymatically and that of Mac1 was measured by ligand displacement. For every parent, we have only reported changes for one type of activity. For instance, if a parent inhibitory constant (*K*_i_) is reported, then only *K*_i_ values are reported for its analogues, and if a parent is an agonist, only EC_50_ values are reported for its analogues. Each functional or inhibition assay included a literature positive-control molecule (Supplementary Fig. 2) with representative concentration–response curves and *Z* values for each assay (Supplementary Fig. 3). The effects of receptor expression on agonism were controlled for (Supplementary Figs. 4 and 5). Full concentration–response curves and analyses are shown for every parent–analogue pair, allowing for direct inspection.

Overall, 23% of the analogues affected activity by twofold or less (fold changes rounded to the nearest integer; Supplementary Table 1) and another 30% reduced activity by tenfold or more (Extended Data Fig. 1b). Intriguingly, 29 of the 257 analogues (11.3%) improved activity by tenfold or more (Table 1). We note that four of these 29 had fold changes of between 9.7 and 9.9; we consider these effectively tenfold improvements and used the same convention in analysing the public ChEMBL data (see Table 1 and Supplementary Table 1 for details). Such tenfold or more improvement analogues were found for five of the six targets, and for 10 of the 18 parents (Table 1). The effects and chemical identities of all analogues versus their parents are listed in Supplementary Table 1.

**Fig. 2:**
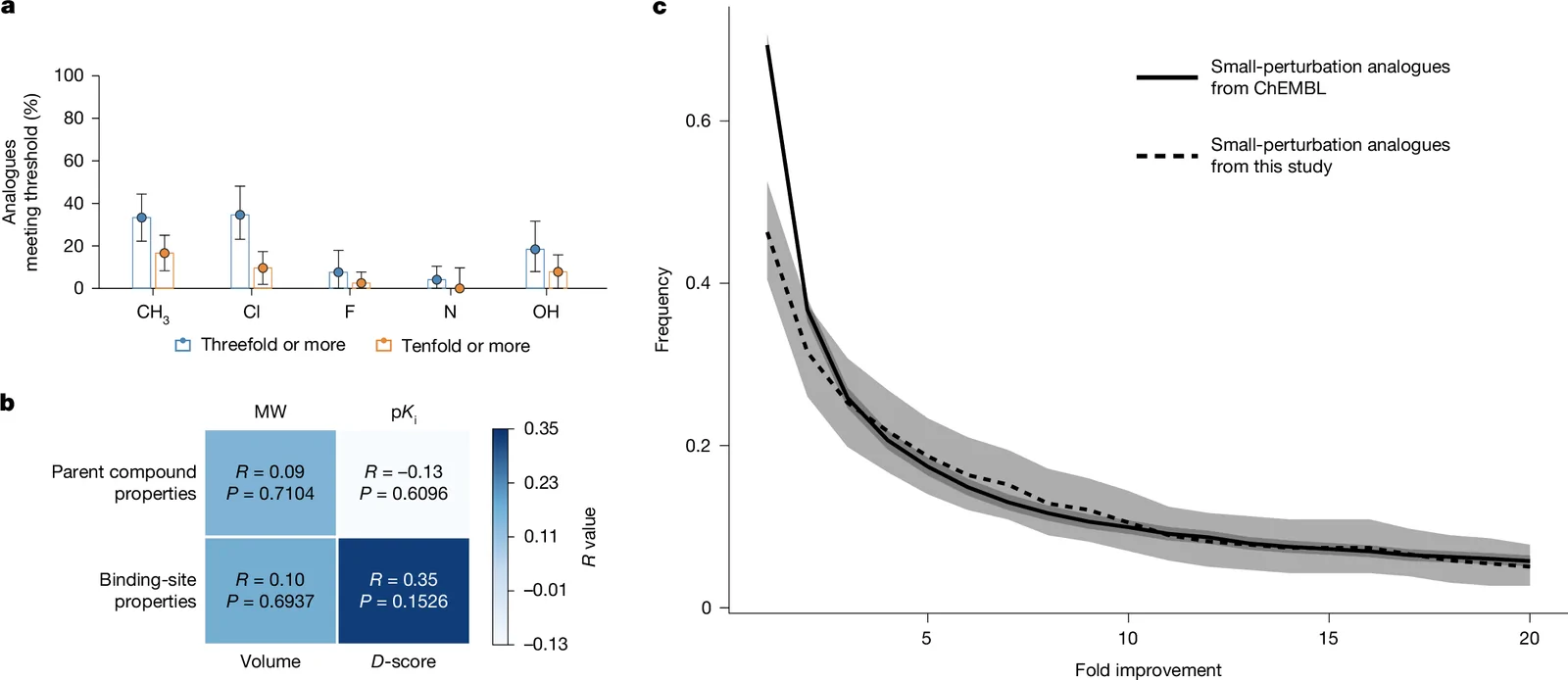
Property correlations of affinity or potency improvement and comparison with literature values. **a**, Observed percentages of compounds achieving threefold or more or tenfold or more activity improvement by single modification (CH_3_, Cl, F, N and OH). The open bars and points show observed percentages; the black error bars show two-sided 95% confidence intervals estimated by percentile bootstrap (10,000 resamples). Sample sizes were *n* = 72, 52, 39, 48 and 38 analogues for CH_3_, Cl, F, N and OH, respectively. **b**, Pearson product–moment correlations between the parent-level rate of analogues achieving tenfold or more affinity or potency improvement and parent molecular weight (MW), parent p*K*_i_, binding-site volume and *D*-score (*n* = 18 parent compounds for each correlation). *R* denotes Pearson’s correlation coefficient. *P* values are two-sided: *P* = 0.7104, 0.6096, 0.6937 and 0.1526 for MW, p*K*_i_, binding-site volume and *D*-score, respectively. **c**, The fraction of single-atom modifications (CH_3_, F, Cl, N and OH) that improve activity, comparing the experimental observations in this study with results from the ChEMBL database. The lines show the observed frequencies; the shaded regions indicate two-sided 95% confidence intervals estimated from 10,000 iterations of bootstrap resampling. The ChEMBL ligands are a composite of 54,021 parents and 79,984 small-change analogues; for only 4.7% of these were there more than three analogues per parent. Source data

We were interested in which perturbations had the biggest effects, how the rates of affinity or potency improvements compared with the literature, with its admitted biases, and if physical properties of the parents or of the binding sites correlated with greater likelihoods of improvement. Among the analogues, methyl substitutions were the most likely to improve activity tenfold or more, with chlorine substitutions the second most likely; for threefold or more improvements, of which there were 69 among the 257 analogues, both chlorine and methyl substitutions were the most common (Fig. 2a). Given the similar size and similar hydrophobicity of methyls and chlorines, their similar effects seem sensible. Conversely, fluorine substitutions, which are often used to increase metabolic stability, yielded only two tenfold or more and five threefold or more activity improvements after rounding fold changes to the nearest integer. Aromatic carbon-to-nitrogen substitutions also rarely improved affinity or potency, although they often had the greatest effects on PK properties.

**Extended Data Fig. 3:**
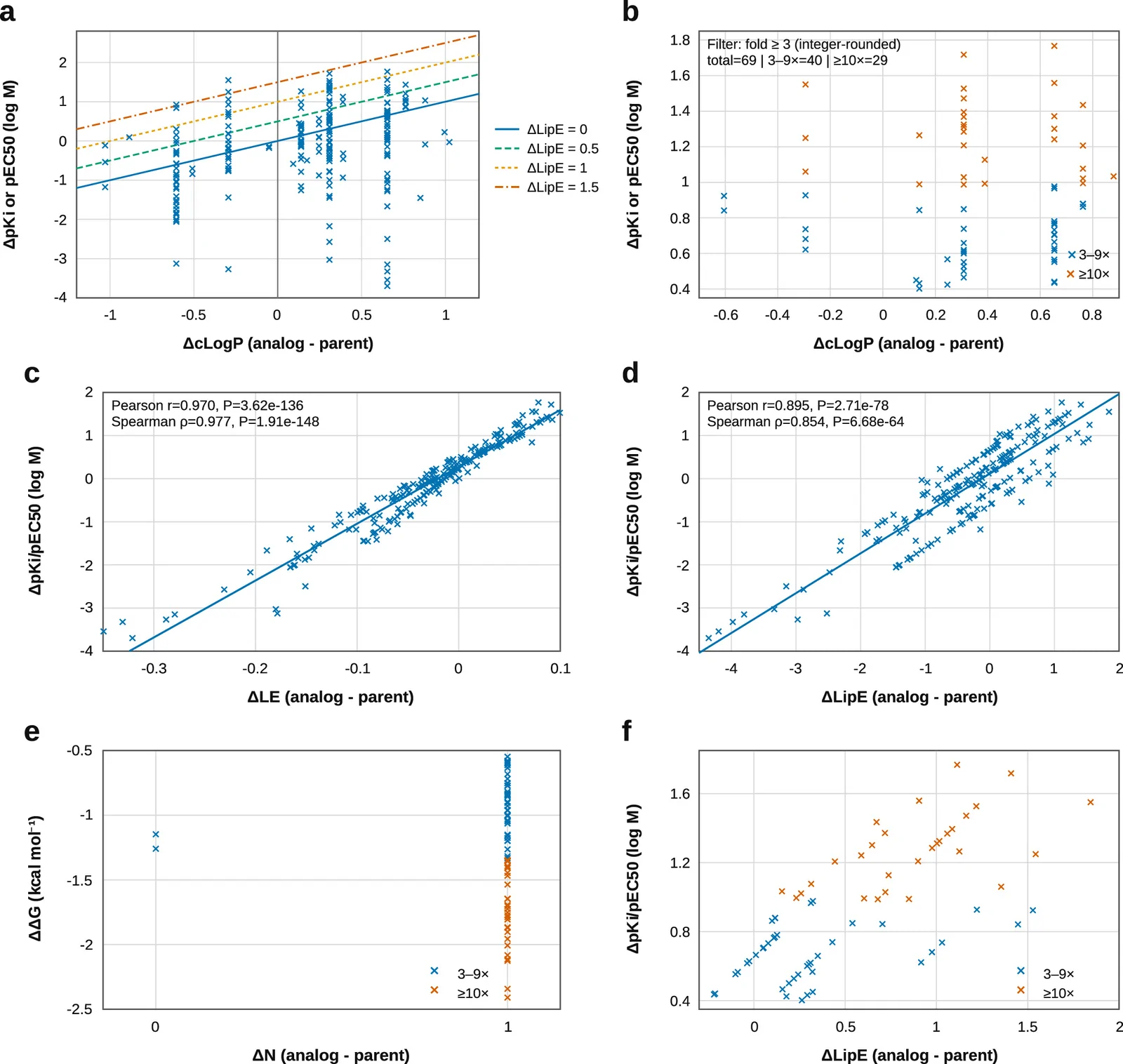
Correlations between physicochemical descriptors and ΔpKi/pEC50 (log M). a, Change in affinity or potency (ΔpActivity = pActivity(analog) − pActivity(parent), where pActivity is pKi, pIC50 or pEC50) plotted against ΔcLogP (analog − parent) for all analog–parent pairs (n = 220). Diagonal isoclines indicate ΔLipE values of 0, 0.5, 1.0 and 1.5, where LipE = pActivity − cLogP; a point above a given isocline has a larger gain in lipophilic efficiency than the indicated value. b, ΔpKi or pEC50 (analog − parent) against ΔcLogP for all analog–parent pairs with ≥3× rounded potency/affinity improvement; ≥10× improvements are highlighted in red. c, Scatter plot of ΔpKi or pEC50 (log M) versus ΔLE (analog − parent), where LE = 1.37 × pKi or pEC50/Nheavy (n = 220 analog–parent pairs). Pearson product–moment and Spearman rank correlations were assessed using two-sided tests (R = 0.970, P = 3.62 × 10 − 136; ρ = 0.977, P = 1.91 × 10 − 148). d, Scatter plot of ΔpKi or pEC50 (log M) versus ΔLipE (analog − parent), where LipE = pKi or pEC50 − cLogP (n = 220 analog–parent pairs). Pearson product–moment and Spearman rank correlations were assessed using two-sided tests (R = 0.895, P = 2.71 × 10 − 78; ρ = 0.854, P = 6.68 × 10 − 64). e, Binding free-energy change versus heavy-atom change for improved analogs. The y-axis shows ΔΔG (kcal/mol), computed from the affinity metric (Ki/IC50/EC50) as ΔG = RT ln(K) (298 K) and ΔΔG = ΔG_analog − ΔG_parent; negative ΔΔG indicates improved binding. The x-axis is ΔN (analog − parent; heavy-atom count difference). Points with rounded fold ≥10 are highlighted in red; rounded 3–9 are shown in blue. f, Affinity or potency gains versus changes in lipophilic efficiency. Scatter plot of ΔpKi or pEC50 (analog − parent) against ΔLipE (analog − parent) for all analog–parent pairs with ≥3× rounded potency/affinity improvement; ≥10× improvements are highlighted in red. Source data

Because hydrophobicity increases will increase affinity simply by disfavouring the unbound state, and will have physical property liabilities, it is useful to consider the improved affinity or potency of the analogues in light of ligand efficiency and lipophilic efficiency (LipE). If affinity or potency improvements were largely driven by hydrophobicity, we would expect ligand efficiency to track with hydrophobicity (here, the calculated octanol–water partition coefficient (clog *P*)) and LipE to remain constant or deteriorate. Instead, analogues that improved tenfold or more had ligand efficiencies that increased much more than hydrophobicity, and had substantially increased LipEs. For instance, an analogue with an added methyl, chloro or other atom that improved in binding or in EC_50_ by even threefold had a ligand efficiency of 0.65 kcal per atom for the added atom, well above the 0.3 kcal per atom thought to be useful for drug optimization^29,30^, whereas for analogues that improved activity tenfold or more, the single-atom ligand efficiency was 1.4 or more, close to the efficiency limit^31^. Similarly, every analogue that improved activity by more than tenfold also improved LipE (Extended Data Fig. 3f). More broadly, among the 220 analogue–parent pairs with complete numerical activity data, Δclog*P* showed little quantitative correlation with ΔpActivity (Extended Data Fig. 3a).

We investigated whether there were correlations between likelihood of potency improvement and ligand and binding-site properties. For instance, one might expect that weaker ligands might be easier to optimize, or that smaller binding sites might be better suited to big affinity jumps. We calculated whether the parent molecular weight, binding-site volume, the ligandability of the binding sites (calculated using *D*-score^32^) or the affinity or potency of the starting parent compound was correlated with the likelihood of finding analogues with substantial affinity or potency improvement. Using the fraction of analogues achieving tenfold or more improvement as the outcome, none of these properties was meaningfully correlated (Fig. 2b). If one considers the magnitude of the affinity or potency change overall, and not just whether it improved tenfold or more, correlations did emerge, with weaker versus stronger parents more likely to support improved affinity (*R* = −0.34, *P* = 3.4 × 10^−7^) and with larger binding sites also tending to do so (*R* = 0.24, *P* = 4.0 × 10^−4^; Supplementary Fig. 6). Although these trends were echoed among matched pairs in ChEMBL (Fig. 2c and Extended Data Fig. 1), the correlations overall were weak. Together, these results suggest that: (1) as a strategy, systematic small perturbations can be unexpectedly successful at improving ligand affinity or potency^23,24^, perhaps even compared with design-heavy approaches^33^; (2) this effect is common across diverse parents and binding sites; (3) it can find echoes in the literature; and (4) random expectations for substantial affinity or potency improvement by small perturbation may be as high as 11% (95% CI 7.5–15.2%).

## Structural bases of activity changes

To investigate the structural bases for the affinity or potency changes, we determined the structure of the parent inhibitors in complex with Mac1 and six analogues from the ‘3122 series, 12 from the ‘3453 series and four from the ‘3794 series, each to between 0.97 and 1.02 Å resolution (Extended Data Tables 1–4). The analogues with structures determined represented all six types of single-atom modifications in this study; 15 of the analogues differed in affinity or potency from their parents by threefold or more, two by tenfold or more and among themselves by up to 680-fold.

**Extended Data Fig. 4:**
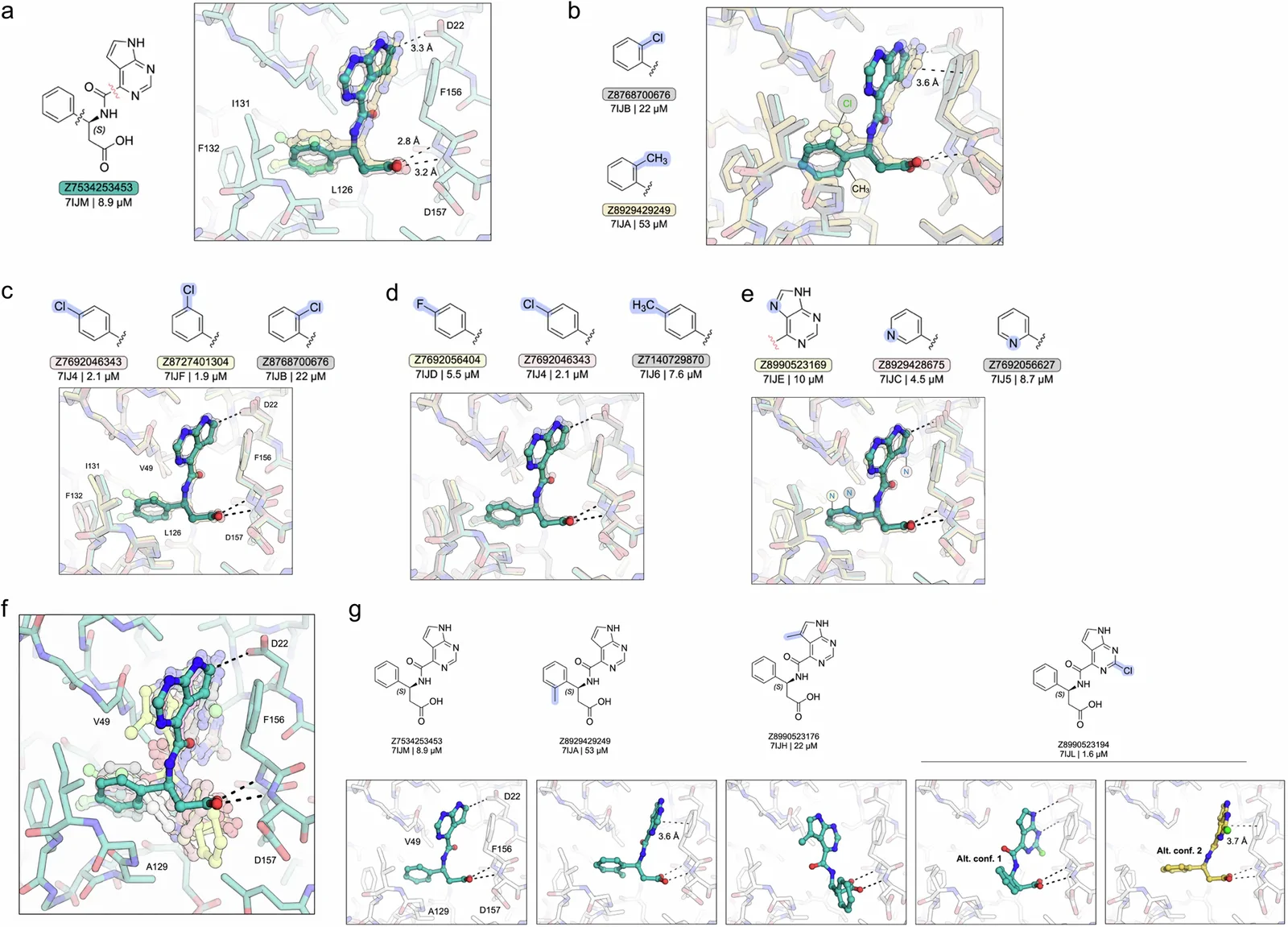
Structural basis and variability of potency changes induced by single-atom modifications in Mac1 inhibitors. a, Chemical structure of the ‘3453 parent and overlay of X-ray crystal structures of 12 analogs. b, Alignment of ‘0676 (21 μM) and ‘9249 (53 μM) with the ‘3453 parent, showing the different binding poses of the ortho-chloro and ortho-methyl analogs. c, Comparison of ‘6343 (2.1 μM), ‘1304 (1.9 μM) and ‘0676 (21 μM) shows that repositioning the aryl chloride disrupts packing with I131 and F132, reducing potency. d, Para-methyl (‘9870, 7.6 μM) and para-chloro (‘6343, 2.1 μM) substitutions have different effects despite similar sterics. The fluoro analog ‘6404 (5.5 μM) binds more weakly than the chloro analog ‘6343, consistent with weaker non-covalent interactions. e, Nitrogen substitutions (C → N) in ‘3169, ‘6627 and ‘8675 have different effects relative to their shared parent (8.9 μM): ‘8675 (meta-N, 4.5 μM) improves potency by approximately twofold, whereas ‘6627 (8.7 μM) and ‘3169 (10 μM) show little or no improvement. f, Overlay of the X-ray crystal structures of 12 ligand-bound complexes from the ‘3453 series, showing that single-atom modifications can cause substantial ligand movement or multiple binding poses. g, Examples of binding-pose shifts: ‘9249, ‘3176 and ‘3194 adopt markedly different poses relative to the ‘3453 parent, and two conformations are observed for ‘3194.

For some parents, analogues superposed well, facilitating analysis. For the Mac1 ‘3453 (IC_50_ of 8.9 µM) inhibitor series (Extended Data Fig. 4a), the introduced chloro at the ortho-position of the phenyl ring (‘0676, IC_50_ of 21 µM) projects into solvent and loses activity versus the parent (Extended Data Fig. 4b). Moving this chloro to the meta-position (‘1304), where it packs with Phe132, Ile131 and Gly48, improves inhibition 11-fold (*P* < 0.01) to 1.9 µM (Extended Data Fig. 4c). Moving it one atom further over, to the para-position, had little effect (‘6343, IC_50_ of 2.1 µM), although a methyl at the same position (‘9870, IC_50_ of 7.6 µM) loses fourfold affinity (*P* < 0.01), presumably reflecting its poorer packing (Extended Data Fig. 4d).

Many of the effects of the small perturbation analogues could be explained post hoc from their structures; however, fewer were easily anticipated. Even the apparent simplicity of the ‘3453 series is belied by structural accommodations in the enzyme. For instance, the para-chloro of the 2 µM ‘6343 would have clashed with the enzyme without Phe132 and Ile131 rotating away. In doing so, Phe132 adopts a partially eclipsed conformation. Presumably, the resulting strain is overcome by improved ligand packing, but this balance of terms is non-obvious (encouragingly, free energy calculations did correctly predict that the para-chloro and meta-chloro would improve affinity, whereas the ortho-chloro would lose it, although the agreement was more qualitative than quantitative; see below). Conversely, the methyl analogue (‘9249, 53 µM) of ‘0676 (IC_50_ of 22 µM) is buried from solvent and might be expected to be more potent than its chloro cousin, but instead it loses another 2.5-fold in *K*_i_ (*P* < 0.01; here, the opposite effect was anticipated by the free energy calculation). Meanwhile, in the complex between ‘4905 and the α_2_AAR, an added methyl group fits into a pre-existing sub-pocket and qualitatively its improved potency is reasonable (Fig. 5a). Quantitatively, its 52-fold effect on EC_50_ is at the outer edge of what might be expected, especially as the methyl buries a serine hydroxyl. This is echoed by the free energy calculation, which here too correctly predicted improved activity but by twofold not 52-fold. These are examples of complexes where the analogues superpose on the parent and each other. In complexes in which the single-atom changes led to substantial inhibitor movement or to the adoption of multiple ligand conformations, prediction was harder still (Extended Data Fig. 4f,g). For example, the Mac1 methyl analogue ‘3176 repositions the inhibitors in the pocket (Extended Data Fig. 4g), whereas compounds ‘9249 and ‘3194 bind in two distinct conformations (Extended Data Fig. 4g), a phenomenon that is probably underreported in the Protein Data Bank^34^ but is readily seen at the ultra-high resolution of these structures. Together, this set of perturbations may provide an interesting and sometimes challenging test for prediction methods.

## A frustrated landscape for ligand affinity or potency and PK

**Fig. 3:**
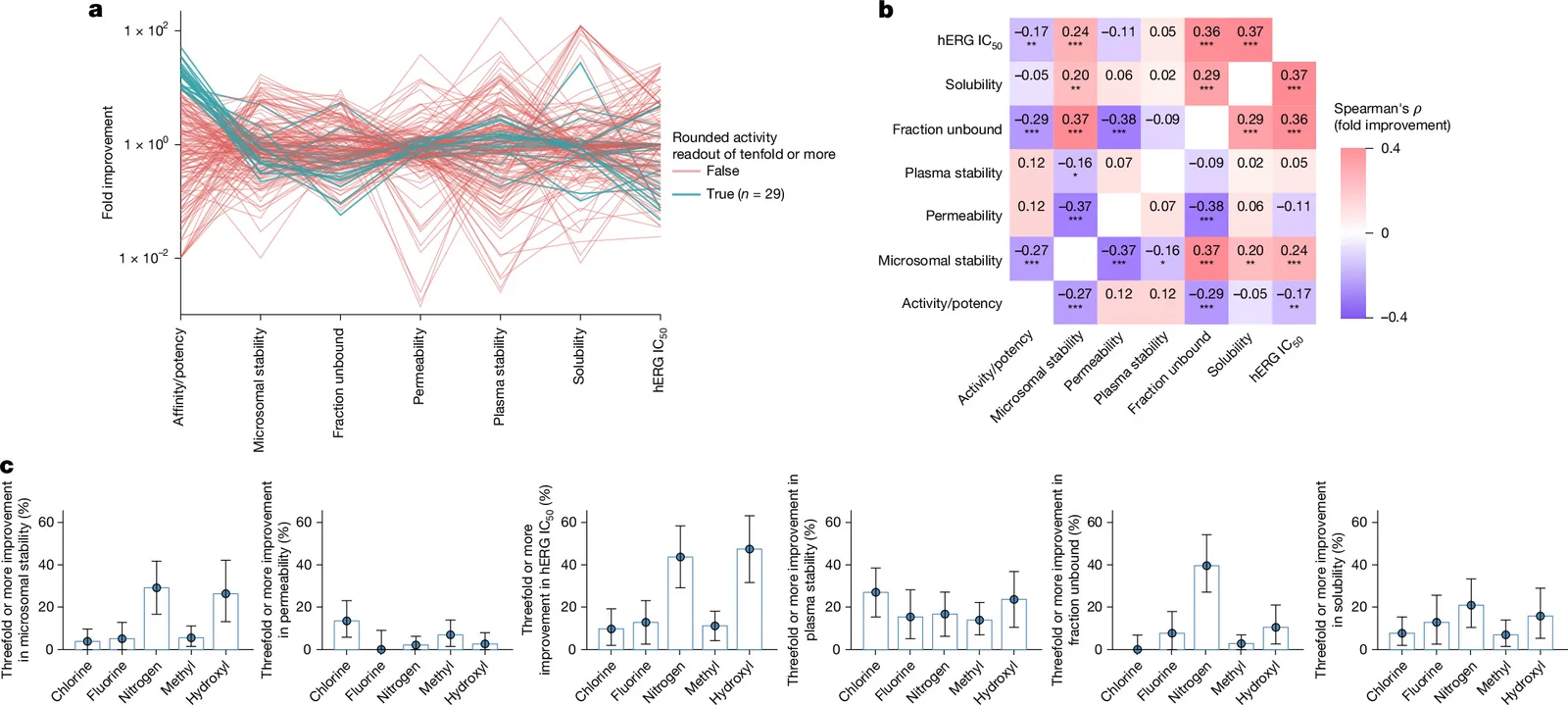
A frustrated landscape for ligand optimization. **a**, Fold changes in PK properties for the 29 analogues classified as tenfold or more improved after integer rounding in affinity or potency versus a parent. **b**, Correlations between affinity or potency fold changes and those of the other PK properties. Correlations were assessed by two-sided Spearman rank correlation; exact pairwise-complete sample sizes (analogue–parent pairs) were: activity orpotency versus microsomal stability, permeability, plasma stability, fraction unbound, solubility and hERG IC_50_, *n* = 203, 203, 203, 193, 191 and 143, respectively; microsomal stability versus permeability, plasma stability, fraction unbound, solubility and hERG IC_50_, *n* = 240, 240, 213, 220 and 166, respectively; permeability versus plasma stability, fraction unbound, solubility and hERG IC_50_, *n* = 240, 213, 220 and 166, respectively; plasma stability versus fraction unbound, solubility and hERG IC_50_, *n* = 213, 220 and 166, respectively; fraction unbound versus solubility and hERG IC_50_, *n* = 200 and 143, respectively; and solubility versus hERG IC_50_, *n* = 154. *P < 0.05, **P < 0.01 and ***P < 0.001. Exact two-sided *P* values for all correlations are provided in the Source Data. **c**, Observed percentages of single-atom changes achieving threefold or more improvement in each parameter. The open bars and points show observed percentages; the black error bars show two-sided 95% confidence intervals estimated by percentile bootstrap resampling (10,000 resamples). Sample sizes by modification were *n* = 52, 39, 48, 72 and 38 analogues for chlorine, fluorine, nitrogen, methyl and hydroxyl, respectively. Source data

**Extended Data Fig. 5:**
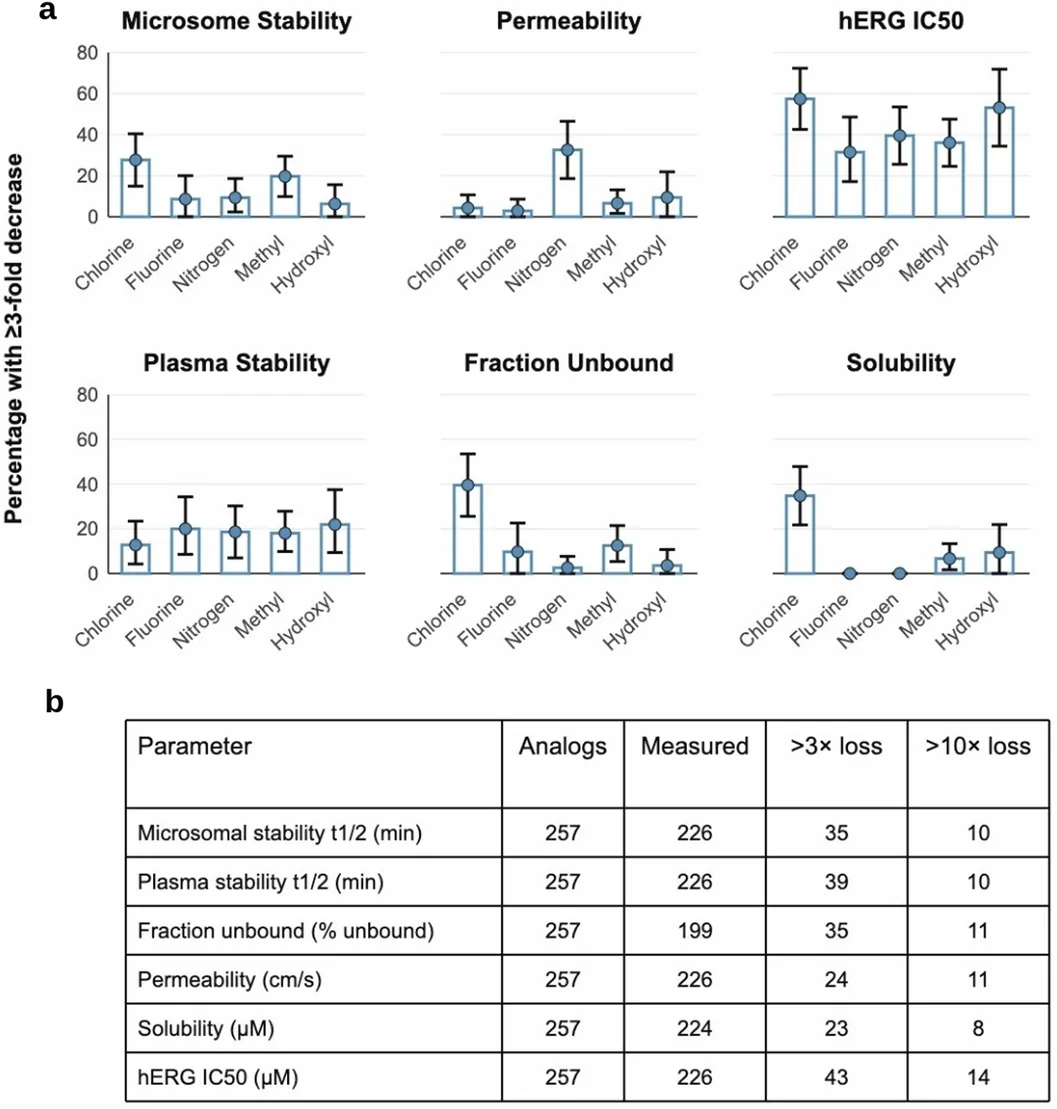
Single-atom substitutions frequently worsen PK properties. a, Observed percentages of single-atom substitutions with ≥3-fold decreases in PK properties. Open bars and points show observed percentages; black error bars show two-sided 95% confidence intervals estimated by percentile bootstrap (10,000 resamples). Exact numbers of measured analogs (Cl, F, N, CH_3_ and OH, in that order) were: microsomal stability, n = 47, 35, 43, 61 and 32; permeability, n = 47, 35, 43, 61 and 32; hERG IC_50_, n = 36, 28, 26, 52 and 17; plasma stability, n = 47, 35, 43, 61 and 32; fraction unbound, n = 43, 31, 39, 56 and 28; and solubility, n = 46, 35, 43, 60 and 32. b, Counts of analogs with measured PK parameters and >3-fold or >10-fold losses. Source data

In optimizing chemical probes and leads, compound PK are as important as molecular target affinity or potency^35,36^. We thus explored how the small-perturbation analogues affected PK properties versus those of the parent ligands, and how these changes related to alterations of affinity or potency. We measured the following in vitro PK properties: metabolic stability in microsomes (half-life), plasma stability (half-life), plasma protein binding (fraction unbound), solubility (µM), hERG inhibition (IC_50_, µM) and membrane permeability (parallel artificial membrane permeability assay (PAMPA); cm s^−1^), for both the parent ligands and their small-change analogues (Fig. 3 and Supplementary Tables 2–7). These admittedly in vitro measurements are considered predictive of in vivo PK^37–39^. Some of the small-change analogues showed meaningful improvements relative to the parent ligands for each of these properties (Supplementary Table 8). For example, 12.4% of the analogues had threefold or more improvement in microsomal stability, 20.8% improved threefold or more in plasma stability, 13.8% improved solubility by threefold or more, 6.2% improved threefold or more in permeability, and 13.2% of the analogues improved threefold or more in plasma fraction unbound. Thus, each property can be meaningfully improved by simple changes. Correspondingly, a substantial fraction of the analogues experienced threefold or more decreases in these same properties (Extended Data Fig. 5). Considering what are perhaps the three most impactful in vitro PK measurements, microsomal stability, fraction unbound and permeability, 41.4% of the analogues experienced more than threefold losses in at least one of these, almost all (93.1%) experienced at least some deterioration by the same criterion, and 34.5% deteriorated in all three in vitro PK properties.

Although many of the small-perturbation analogues substantially improved individual PK properties, none of the 29 analogues classified as tenfold or more improved after integer rounding showed improvements across all three key properties: metabolic stability, PAMPA permeability and plasma fraction unbound (Fig. 3a and Supplementary Fig. 7); many were worse in all three properties. Indeed, the changes in in vitro PK were, at best, orthogonal to affinity or potency fold change (Fig. 3b), and most were, if anything, anti-correlated with it, including plasma fraction unbound, microsomal stability, hERG inhibition and, to a minor extent, solubility. This is borne out at the level of individual substitutions: the substituent groups that most often improved affinity or potency substantially, such as methyl and chloro groups (Fig. 2a), were least associated with improved PK properties (Fig. 3c and Supplementary Fig. 8). Meanwhile, those changes least likely to improve affinity or potency substantially, such as aromatic C→N, were most likely to improve microsomal stability or fraction unbound (Supplementary Fig. 8). More broadly, most of the PK properties were largely independent of one another, with no correlations exceeding |*ρ* | > 0.5 and those that were statistically significant having Spearman *ρ* values ranging only from |0.16| to |0.38|. What emerges is a frustrated landscape for ligand optimization, with affinity or potency improvements counterbalanced by worsening PK. Although this trade-off has been well noted in medicinal chemistry^40,41^, this set can quantify its systematic impact.

## Prediction of analogue properties

**Fig. 4:**
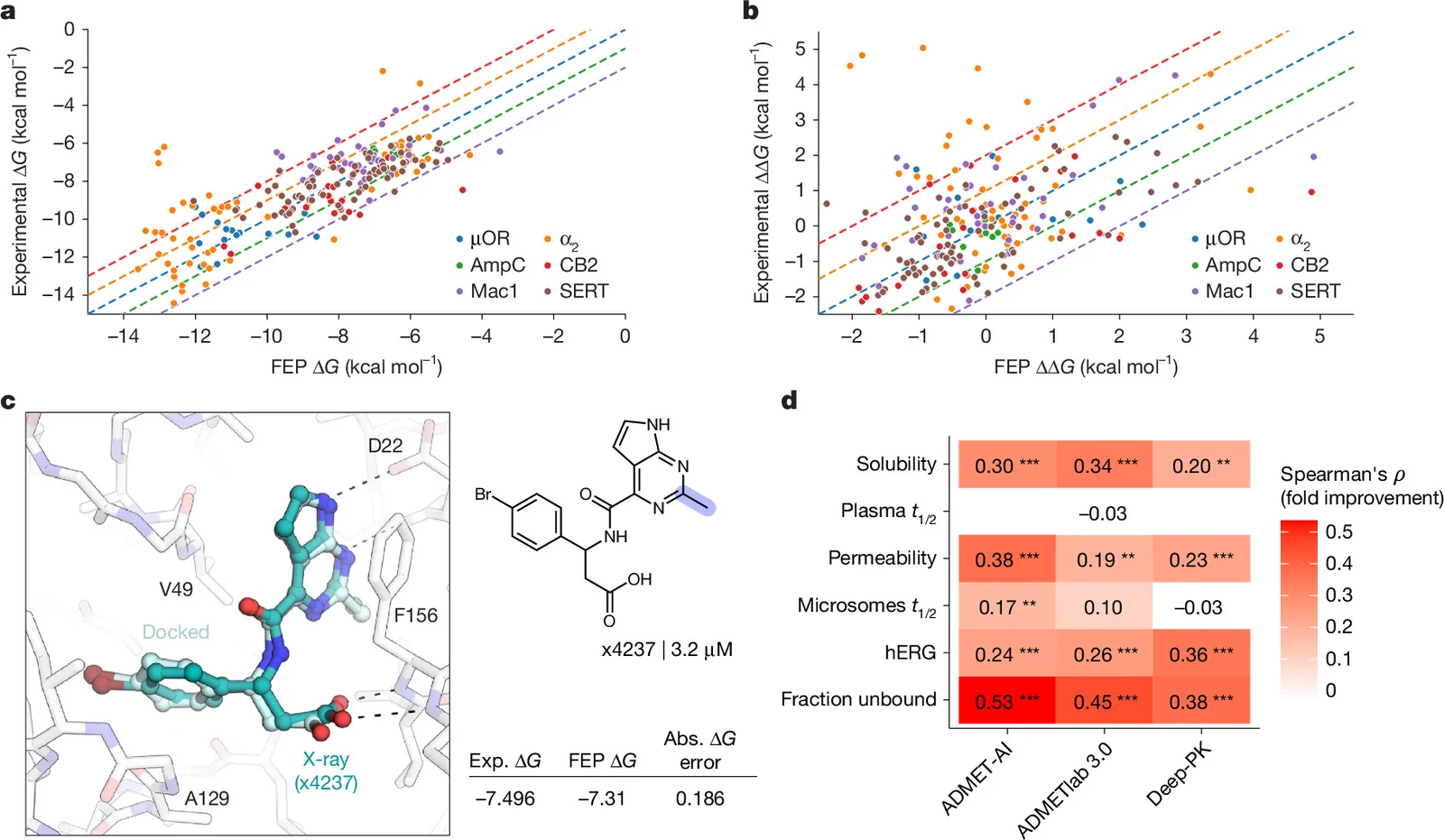
Accuracy of computational predictions of analogue relative affinity and in vitro PK. **a**, Comparison of FEP-predicted and experimentally measured binding free energies (Δ*G*). The dashed lines indicate ±1 kcal mol^−1^ and ±2 kcal mol^−1^ error margins. **b**, Correlation between FEP-predicted and experimentally measured relative binding free energy changes (ΔΔ*G*). Dashed lines are as in panel **a**. **c**, An example of good structural alignment between the predicted (cyan) and experimental (light blue) poses, accompanied by a small error in the predicted ΔΔ*G*. Dashed lines indicate hydrogen-bonding interactions. Abs., absolute; Exp., experimental. **d**, Heatmap of Spearman correlations between AI-predicted and experimentally measured fold changes across six in vitro PK properties, evaluated using three models. The colour intensity indicates correlation strength; correlations were assessed by two-sided Spearman rank correlation; pairwise complete-case *n* for Deep-PK, ADMET-AI and ADMETlab 3.0 were 218, 218 and 218, respectively, for solubility; 222 for the comparable ADMETlab 3.0 plasma-stability end point (the Deep-PK and ADMET-AI end points were not directly comparable); 222, 222 and 222, respectively, for permeability; 222, 222 and 222, respectively, for microsomal stability; 221, 221 and 221, respectively, for hERG; and 195, 195 and 195, respectively, for fraction unbound. Exact two-sided *P* values for all correlations are provided in the Source Data. **P < 0.01 and ***P < 0.001. *n* = 219 analogues (**a**,**b**). Source data

The current benchmark only comprises several hundred analogues. If we could predict the results of the small perturbations, rather than having to experimentally test them, it could be much extended. We therefore asked how well modern methods could predict the affinity or potency and PK changes that we observed. In blinded experiments, we used free energy perturbation, arguably the highest level of theory available to the field, with the program FEP+ (Schrödinger) to predict the changes in activity between the parents and analogues (Fig. 4a–c). Similarly, we asked how well we could predict in vitro PK changes between the parents and analogues, using widely accessible tools (Fig. 4d).

There was a strong overall correlation between the FEP+-predicted and experimentally measured binding free energies (Δ*G*), with 188 of the 219 predictions falling within ±2 kcal mol^−1^ of the experimental values, and 129 within ±1 kcal mol^−1^, whereas the set had a mean unsigned error for Δ*G* of 1.06 kcal mol^−1^ (Fig. 4a,b and Supplementary Table 9). Of the 18 parents, the FEP+ predictions on 14 had Δ*G* mean unsigned error below 1.2 kcal mol^−1^ (Supplementary Table 10). If one considers the correlations qualitatively, of the 35 analogues predicted to lose affinity by 1 kcal mol^−1^ or more by FEP+ (that is, in the range of confident prediction), 20 did so experimentally. Meanwhile, of the 34 predicted to increase in affinity or potency by more than 1 kcal mol^−1^ by FEP+, 14 did so experimentally, whereas 12 were experimentally neutral and eight showed decreased potency. Categorization with a 1 kcal mol^−1^ threshold revealed a significant association between the direction and strength of FEP+ predictions and experimentally observed affinity or potency changes (*χ*^2^ = 36.4 and *P* < 0.001; Supplementary Table 11).

Several challenges faced FEP predictions here, which future work may share. The predictive power was probably weakened by our inability to measure activity for weak analogues, reducing activity range and making it impossible to measure affinities predicted to be very low by FEP+. We used an automated workflow to generate initial poses for FEP; on inspection, most of the outliers reflected errors in these poses. FEP accuracy is impacted by the quality of the ligand-complexed structures^42^, and the predictions here began with docked poses, which are less reliable than experimental poses. For the 25 analogue structures that we determined by crystallography (for Mac1) and by cryo-electron microscopy (cryo-EM; for an α_2_A analogue, below), where predicted and experimental poses closely superimposed, FEP+ energies were largely consistent with experiment (Fig. 4c).

To predict in vitro PK^43,44^, we used ADMET-AI, ADMETlab 3.0 and Deep-PK^45–47^. The predictions of all three correlated with the experimental measurements of plasma fraction unbound and with permeability, whereas microsomal stability, plasma stability and solubility, which are difficult to predict^48,49^, were essentially uncorrelated. Correlations in the 0.4–0.5 range for fraction unbound and permeability may often be strong enough to guide compound selection and these open-access tools may have broad impact. Still, even with a correlation of 0.53 in fraction unbound, the highest that we observed, 28 analogues that had substantially increased fraction unbound experimentally were predicted to have lower levels than their parents, and 16 analogues that had higher protein binding than their parents experimentally were predicted to have less. Together, although the FEP and in vitro PK predictions can profitably model the effects of the small-perturbation analogues, their correlations with experiment are not yet strong enough to allow this benchmark to be extended by calculation alone.

## Multi-parameter optimization for an improved analgesic

**Fig. 5:**
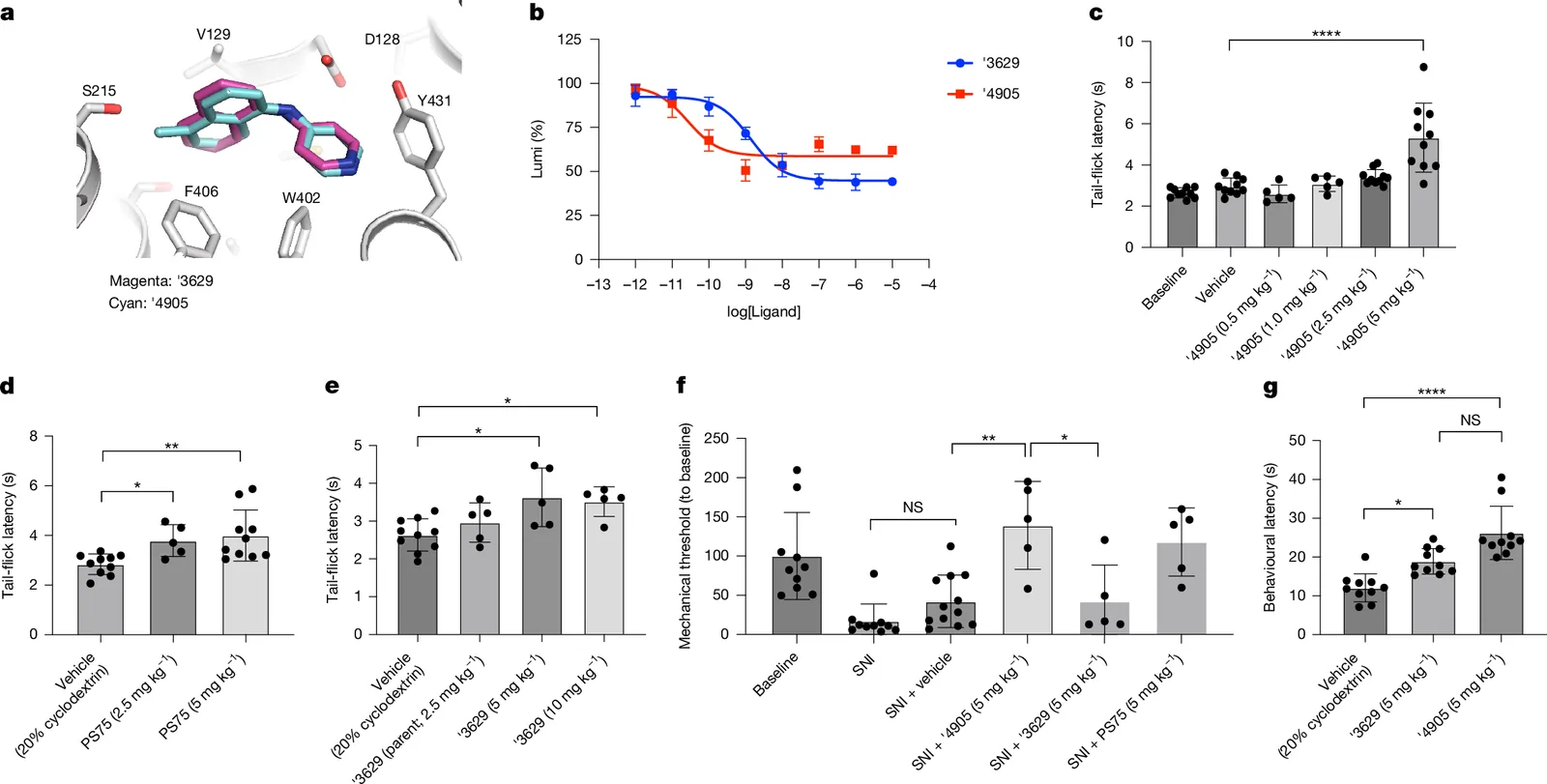
Methyl addition enhances α_2_A agonist analgesia in vivo. **a**, Docked parent ‘3629 superposed on the cryo-EM structure of the methyl analogue ‘4905. **b**, Concentration–response curves for α_2_AAR activation by ‘3629 and ‘4905; the high-concentration upturn for ‘4905 may reflect G_s_ activation. Data are mean ± s.e.m. from *n* = 3 independent experiments, each with two technical replicates. **c**–**e**, Tail-flick latencies after vehicle and ‘4905 (**c**), PS75 (**d**) or ‘3629 (**e**) at the indicated doses. **f**, Mechanical withdrawal threshold at baseline, after spared nerve injury (SNI), and after SNI + vehicle, ‘4905, ‘3629 or PS75 (5 mg kg^−1^ each). **g**, Hotplate latency after vehicle, ‘3629 or ‘4905 (5 mg kg^−1^). Mice were male C57BL/6J, 7–8 weeks of age; assignment was randomized and testing was blinded. For **c**–**g**, data are mean ± s.d.; points are individual mice; and group sizes (left to right) were: *n* = 10, 10, 5, 5, 10 and 10 (**c**); *n* = 10, 5 and 10 (**d**); *n* = 10, 5, 5 and 5 (**e**); *n* = 10, 10, 12, 5, 5 and 5 (**f**); *n* = 10, 10 and 10 (**g**). Tests were two-way analysis of variance (ANOVA) with Dunnett’s test (**c**), Kruskal–Wallis with Dunn’s test (**d**,**e**), two-way ANOVA with Tukey’s test (**f**) and Friedman with Dunn’s test (**g**). Exact *P* values: vehicle versus increasing ‘4905 doses of 0.9759, 0.9841, 0.7141 and <0.0001 (**c**); vehicle versus PS75 of 0.0301 and 0.0043 (**d**); vehicle versus ‘3629 of >0.9999, 0.0393 and 0.0164 (**e**); SNI versus SNI + vehicle of 0.7416, SNI + vehicle versus SNI + ‘4905 of 0.0037, SNI + vehicle versus SNI + PS75 of 0.0333, SNI + ‘4905 versus SNI + ‘3629 of 0.0188 (**f**); vehicle versus ‘3629, vehicle versus ‘4905 and ‘4905 versus ‘3629 of 0.0417, <0.0001 and 0.2209, respectively (**g**). *P < 0.05, **P < 0.01, ****P < 0.0001 and not significant (NS). Source data

Notwithstanding the frustrated landscape for ligand affinity or potency and PK improvement, medicinal chemists can often navigate this multi-parameter landscape to improve the properties of lead molecules. Here too, there were analogues that improved in affinity or potency with only a modest decrease in PK. Among these was an analogue of an α_2_A parent, compound ‘4905, where a single methyl group substitution improved potency 52-fold (Fig. 5a,b). In a cryo-EM structure of the ‘4905–α_2_A–G protein complex determined to 2.8 Å resolution (Extended Data Table 5), ‘4905 superposed with the docking prediction for the parent ‘3629 and with the experimental structure of a previously described member of this family, ‘9087. This suggests that the methyl perturbation did not change the orientation of the analogue and that this family may be understood in the same structural context. A slight upwards deflection at high concentrations of ‘4905 in the cAMP dose–response curves may reflect secondary G_s_ coupling, supported by pertussis toxin-treated cAMP assays showing a residual, concentration-dependent increase in cAMP for ‘4905 but not ‘3629 (Supplementary Fig. 9a). Structurally, the added methyl group fills a hydrophobic sub-pocket (Fig. 5a and Supplementary Fig. 9b), helping to explain its improved potency.

Although the improved potency of **‘**4905 came at a cost to its in vitro PK, the effects were modest: compared with its parent, ‘3629, the fraction unbound decreased by 57% and the microsomal half-life was reduced by 41% (Supplementary Tables 2–7). The in vivo PK of **‘**4905 qualitatively reflected the in vitro results, with cerebrospinal fluid concentrations—a proxy for fraction unbound in the brain^50^—reduced by 36%, whereas half-life was reduced from 196 to 15.2 min (Supplementary Table 12). Accordingly, we compared the in vivo analgesia of ‘4905 to that of its parent ‘3629 and to PS75, the most potent analgesic known for this family of α_2_A agonists^51^. Despite the deterioration in PK, in mouse nociception assays including tail flick, neuropathic pain (SNI) and hotplate, **‘**4905 was far more potent than the parent ‘3629 and at least as potent as PS75 (Fig. 5c–g). If ligand optimization is a frustrated landscape, it remains a navigable one^41^. In addition to beginning to provide a random background for such efforts, this study supports a strategy by which that landscape may be reconnoitered^23,24^.

## Discussion

Although ligand optimization is at the heart of drug discovery, quantifying the success of optimization strategies has been difficult owing to a lack of a random background. Four key observations emerge from this study. First, systematic and unbiased small perturbations to 18 scaffolds revealed that 11.3% of the analogues improved tenfold or more in affinity or potency versus the parent (Table 1); ‘magic methyls’^25,52^ were common in this set. These improvements were observed in five of six targets, across ligand physical properties and across five orders of magnitude of parent activity. This begins to establish a background expectation for affinity or potency optimization against which methods and designs may be compared. It may also suggest a simple, unbiased approach for achieving substantial jumps in affinity or potency, improvements that will not always be obvious from structural analyses or simulations (Figs. 4 and 5 and Extended Data Fig. 4) but may complement them. Second, improved affinity or potency typically came at the cost of ligand PK, with plasma fraction unbound, stability to liver microsomes and permeability, among other terms, declining as analogue affinity or potency increased. The individual in vitro PK terms were orthogonal to each other and several were anti-correlated with affinity or potency improvement (Fig. 3). This suggests a frustrated landscape for ligand optimization. Although the qualitative challenges and trade-offs in ligand optimization are known to medicinal chemists^8,40,41^, this study has begun to quantify them across a range of target classes and ligand properties. Third, computational prediction of affinity or potency and PK had some success anticipating the trends that we observed (Fig. 4). Still, their correlations with experiment remained loose enough to preclude replacing systematic exploration and testing of molecules as this background set is expanded, something seen with other potency prediction methods^53^. Fourth, although potency trades off against PK, it is possible to find analogues where it rises sufficiently to overcome drops in exposure and half-life. Thus, the α_2_A analogue ‘4905 is the most potent analgesic in a large series, and is far more potent in vivo than its parent, despite experiencing these trade-offs.

At first glance, this study may recall classical medicinal chemistry where conservative and ligand-based approaches such as Topliss Trees^11^ dominated the field. However, this would be to misremember the classic age of medicinal chemistry on two counts. First, the systematic small perturbations explored here were rarely used then, partly because the building blocks and reactions to support them were lacking. The ability to systematically explore small perturbations across scaffolds reflects advances in synthetic chemistry and the new building-block approaches that have expanded readily available compounds^54,55^. Second, the logic behind Topliss Trees and other quantitative structure–activity approaches^11,12^ was anchored in ligand physical chemistry without reference to receptor structure. For instance, in Topliss Trees the success of methyl and chloro derivatives taught opposite lessons because of their different electronic effects on the ligand, whereas the steric and non-polar properties of the two groups will seem similar from the view ‘inside the receptor’^52,56^. Meanwhile, the role of a random background would have seemed foreign. Thus, although a strategy of systematic small perturbations may smack of the ‘methyl, ethyl, propyl, butyl, futile’ approach with which classic medicinal chemistry has been tarred^57,58^, it has a different basis in theory and reflects new synthetic opportunities.

Certain limitations of this study merit airing. At 257 analogues and six targets, the set remains small and inevitably, if unintentionally, biased. Expanding to additional targets and ligand classes might reveal different trends. We note that the six targets do recognize a range of chemotypes, including large polar co-factors (Mac1), large hydrophobic lipids (CB2), anionic β-lactams (AmpC), cationic neurotransmitters (α_2_A and SERT) and peptides (µOR). Experimentally, we assessed activity changes using a transporter uptake assay (SERT), radioligand competition binding assays (α_2_A and CB2), second messenger assays (α_2_A, CB2 and MOR), and binding and enzyme activity (Mac1 and AmpC) assays. Although ligand competition correlates with *K*_i_, agonist EC_50_ values are affected by receptor expression. Because we compared the relative activities of analogues and parents, controlled for receptor expression (Supplementary Fig. 4), and measured effects at low expression levels (Supplementary Fig. 5), the effects of receptor expression on relative activity should be modest, although they cannot be completely discounted. For these and related reasons, changes of EC_50_ between agonist parent and analogues cannot be read as changes in affinity the way that changes in *K*_i_ can be, although EC_50_ changes remain the relevant metric for agonist activity. Overall, the impact of these effects may be inspected in full concentration–response curves for parent–ligand pairs with more than threefold activity improvement (Supplementary Fig. 2). In the area of in vitro PK, our use of liver microsomes rather than hepatocytes to measure metabolic stability meant that some types of metabolism were missed, including glucuronidation. This might make groups such as phenolic hydroxyls seem less labile than they would be in vivo. More broadly, for all but two molecules, we only measured in vitro not in vivo PK. Whereas the latter are widely used in ligand optimization, their prediction of in vivo behaviour is only approximate.

These limitations should not obscure the main observations of this study. Overall, 11.3% of systematic and unbiased small perturbations improved analogue affinity or potency tenfold or more, beginning to establish a background expectation for the likelihood of substantial ligand affinity or potency improvement and a systematic approach to doing so^23,24^. Balancing this was a concomitant deterioration in ligand PK, which will lower the exposure and half-life of a ligand in vivo, counteracting the improvements in affinity or potency. Navigating this multi-parameter space is at the heart of medicinal chemistry; this study supports the development of quantitative models to do so.

## Methods

### ChEMBL database molecular pairs

From the PostgreSQL version of CHEMBL34 (ref. ^4^) (https://doi.org/10.6019/CHEMBL.database.34), we filtered the database for compounds with activities reported against a single protein, with either *K*_i_, *K*_d_, IC_50_ or EC_50_ as the activity type. To be considered a molecular pair, compounds had to have the same assay ID, the same target ID, the same activity type and the same reference document (publication) ID, in addition to having a parent–analogue relationship as defined in this work, meaning a C–H group replaced by C–OH, C–F, C–Cl, C–Br, C–CH_3_ or an aromatic carbon replaced by a nitrogen. This left us with 191,732 parent–analogue pairs.

### Analogue enumeration and synthesis

Eighteen parent compounds were selected from previously published literature^51^^,59,60^ or datasets (https://asapdiscovery.org/outputs/molecules/#ASAP-SARS-COV-2-NSP3-MAC1) based on their known binding activity, structural relevance or representation of diverse chemical scaffolds. Starting from a parent compound, we used RDKit (http://www.rdkit.org/) to identify all C–H bonds and iteratively replaced the hydrogen atom with a methyl, hydroxyl, fluoro, chloro or bromo group. We also identified aromatic carbons with two heavy-atom neighbours and replaced them with nitrogen. Every analogue generated was represented as a canonical isomeric SMILES string and added to a set to remove duplicates. For each of the 18 parents, the full set of possible analogues that could be synthesized for less than US$400 for 10 mg was ordered.

### Confidence intervals and statistics

When reported, 95% confidence intervals were derived from bootstrap resampling with 10,000 iterations. In cases in which the observed frequency of success is exactly 0, bootstrap will fail to give an upper bound for the interval. In such cases, we used the Clopper–Pearson method to estimate an upper bound based on the sample size^61^. Pearson and Spearman correlations with their associated *P* values were computed using the scipy.stats module from the SciPy package^62^.

### FEP simulations

These were conducted using FEP+ within the Schrödinger software suite (v2025-2) with the OPLS4 force field^63^ and the modified simple point charge water model. The default setting was used for the number of lambda windows selection where it depends on the type of perturbations; charge-changing, core hopping and all other perturbations have 24, 16, and 12 lambda windows, respectively. For alchemical transformations with charge changes, the total charge of the simulation box was kept constant by transmuting a Na^+^ or Cl^−^ ion to water or vice versa. In addition, a 0.15 M concentration of NaCl was added to the simulation box of charge-changing perturbations. For α_2_A, CB2 and SERT, the FEP+ membrane protocol was applied where a POPC membrane was added to the system in simulation. All other settings were kept default except that the simulation time was extended from 5 ns to 10 ns.

The default FEP map generation protocol was used with the parent compound selected as the biased node. In preparing proteins and ligands for FEP+, the Schrödinger protein preparation workflow and LigPrep were used. The initial binding poses of parent compounds were from poses generated by DOCK3.8, and analogues were aligned to the parents with severe steric clashes resolved using the FEP+ Pose Builder workflow.

### Docking

Whereas no structural information was used in the design of analogues from parent compounds, for FEP+ calculations and for post hoc structural analysis, we generated ligand-bound complexes of the parents and relevant ligands using DOCK.3.8 (refs. ^54,64^). Ligands were docked into the receptor-binding site using grids prepared in previous studies^51,59,60,65,66^.

### Assay selection

We used one consistent readout per parent series (no mixing within a series). SERT and AmpC are reported as *K*_i_; Mac1 is reported as IC_50_ from peptide displacement (a scalable functional hydrolysis assay is unavailable); GPCR agonist series are reported as EC_50_ and antagonist series as *K*_i_, such that μOR is EC_50_ only, whereas α_2_A and CB2 include EC_50_ and *K*_i_ depending on the parent series. Representative concentration–response curves and the corresponding *Z* values for each assay are provided in Supplementary Fig. 3.

### Transporter assays for SERT *K*_i_

SERT activity was measured using the Neurotransmitter Transporter Uptake Assay Kit from Molecular Devices (#R8174), following the manufacturer’s protocol with slight modifications as previously described^65^. HEK293T cells (ATCC CRL-3216) stably expressing human SERT were plated in poly-L-lysine-coated 384-well black, clear-bottom plates at a density of 15,000 cells in 40 µl per well, using DMEM supplemented with 1% dialysed FBS. Cells were incubated overnight at 37 °C with 5% CO_2_ to allow adherence and recovery. The following day, the medium was removed by flicking, and cells were incubated with 25 µl per well of test compound solutions prepared in assay buffer (1× HBSS, 20 mM HEPES, pH 7.4, supplemented with 1 mg ml^−1^ BSA) for 30 min at 37 °C. After drug treatment, 25 µl per well of dye solution (as provided in the kit) was added directly to the wells, followed by an additional 30-min incubation at 37 °C. Fluoxetine (10 µM) was used as a positive control for SERT inhibition. Fluorescence was measured using the FlexStation II microplate reader with excitation at 440 nm and emission at 520 nm. Relative fluorescence units were exported and analysed using GraphPad Prism 10.0 to calculate IC_50_ values, from which *K*_i_ values were derived using the Cheng–Prusoff equation.

### CB2 radioligand-binding assay

CB2 receptor-binding assays were performed using membrane preparations from HEK293 cells (ATCC CRL-1573) stably expressing human CB2, following previously published methods^67,68^. Membranes were resuspended in TME buffer containing 0.1% BSA (w/v) and 25 µg of membrane protein was added per well. The assay was conducted using the radioligand [^3^H]CP-55,940 at a final concentration of 0.75 nM, prepared in assay buffer. Nonspecific binding was defined in the presence of 5 µM unlabelled CP-55,940. Test compounds were applied at increasing concentrations to assess competition. Reactions were incubated at 30 °C for 1 h with gentle shaking. After incubation, samples were transferred to Unifilter GF/B 96-well filter plates and filtered using a Packard Filtermate-196 cell harvester (PerkinElmer). Plates were washed four times with ice-cold wash buffer (50 mM Tris-HCl, 5 mM MgCl_2_ and 0.5% BSA, pH 7.4). Radioactivity bound to the filters was quantified via liquid scintillation counting. Specific binding was calculated by subtracting nonspecific binding from total binding. IC_50_ and *K*_i_ values were calculated using nonlinear regression in GraphPad Prism 9 using the Cheng–Prusoff equation.

### α_2_A receptor-binding assay

α_2_AAR binding was performed using membrane preparations from insect cells (Expression Systems, 94-001S) expressing human α_2_A receptors, as previously described^51^. Membranes were incubated with increasing concentrations of test compounds and 5 nM [^3^H]rauwolscine in buffer containing 20 mM HEPES (pH 7.5) and 100 mM NaCl, at room temperature for 2 h. After incubation, samples were filtered onto GF/B filter plates, washed with ice-cold buffer and radioactivity was quantified by liquid scintillation counting. IC_50_ and *K*_i_ values were derived using nonlinear regression in GraphPad Prism.

### GloSensor cAMP assay for α_2_A, CB2 and MOR

The GloSensor cAMP assay was performed following the manufacturer’s instructions (Promega) with slight modifications as previously described^51,69^. In brief, wild-type human α_2_A, CB2 and μOR were cloned into the pcDNA3.1 vector and co-transfected with the 22F cAMP GloSensor plasmid into HEK293T cells (ATCC CRL-3216) cultured in six-well plates. After 24 h, cells were reseeded into 96-well white plates in CO_2_-independent medium and equilibrated with GloSensor cAMP reagent as per the manufacturer’s protocol. Cells were incubated for 1 h at 37 °C followed by 1 h at room temperature. Where applicable, 10 μM forskolin was used to elevate basal cAMP levels for assessing receptor-mediated inhibition. Serially diluted test compounds were added, and luminescence signals were recorded using a PerkinElmer microplate reader. Data were analysed using GraphPad Prism 9.0 to calculate EC_50_ or IC_50_ values.

### AmpC β-lactamase inhibition assay

The AmpC β-lactamase inhibition assay was performed as previously described^60^. The candidate inhibitors were dissolved in DMSO (20 mM stock) and diluted to maintain a constant 1% DMSO (v/v) in 50 mM sodium cacodylate buffer (pH 6.5). Assays were performed in the presence of 0.01% Triton X-100 to reduce aggregation artefacts. AmpC enzymatic activity was monitored spectrophotometrically using CENTA or nitrocefin as substrates. Initial screening was performed at 200 µM, 100 µM and 40 µM compound concentrations. Substrate concentrations were selected based on known *K*_m_ values to achieve defined [*S*]:*K*_m_ ratios: for CENTA ([*S*] = 50 µM, *K*_m_ = 27.6 µM) and nitrocefin ([*S*] = 100 µM or 28 µM, *K*_m_ = 180 µM). Reactions were carried out in 96-well format on a BMG Labtech CLARIOstar plate reader, with substrate and enzyme injected into wells containing the inhibitor, followed by kinetic measurement over 50 s. IC_50_ values were determined by fitting inhibition curves in GraphPad Prism using a fixed Hill coefficient of 1, and *K*_i_ values were calculated using the Cheng–Prusoff equation^70^.

### HTRF assay for Mac1

Binding of the compounds to Mac1 was assessed by the displacement of an ADPr-conjugated biotin peptide from His6-tagged protein using a homogeneous time-resolved fluorescence (HTRF)-based assay, as previously described^71^. The expression sequences used for SARS-CoV-2 Mac1 are listed below. All proteins were expressed and purified as previously described for SARS-CoV-2 Mac1 (ref. ^71^). Compounds were dispensed into ProxiPlate-384 Plus (PerkinElmer) assay plates using an Echo 650 Liquid Handler (Beckman Coulter). Binding assays were conducted in a final volume of 16 μl with 12.5 nM NSP3 Mac1 protein, 200 nM peptide ARTK(Bio)QTARK(Aoa- RADP)S (Cambridge Peptides), 1:20,000 anti-His6-Eu3^+^ cryptate (HTRF donor; PerkinElmer AD0402) and 1:500 streptavidin-XL665 (HTRF acceptor; PerkinElmer 610SAXLB) in assay buffer (25 mM HEPES pH 7.0, 20 mM NaCl, 0.05% bovine serum albumin and 0.05% Tween-20, the latter also to reduce aggregation artefacts). Assay reagents were dispensed manually into plates using an electronic multichannel pipette. Mac1 and peptide were pre-incubated for 30 min at room temperature before HTRF reagents were added. Fluorescence was measured after a 1-h incubation at room temperature using a Perkin Elmer EnVision 2105-0010 Dual Detector Multimode microplate reader with dual emission protocol (A = excitation of 320 nm and emission of 665 nm, and B = excitation of 320 nm and emission of 620 nm). Compounds were tested in triplicate in a 14-point dose response. Raw data were processed to give an HTRF ratio (channel A/B × 10,000), which was used to generate IC_50_ curves using nonlinear regression using GraphPad Prism v10.0.2 (GraphPad Software).

### hERG channel inhibition

hERG channel inhibition was evaluated using a Thallium Flux assay on HEK293 cells (ATCC CRL-1573) stably expressing the hERG potassium channel. Cells were seeded at a density of 8,000 cells per well in 384-well poly-D-lysine-coated plates and incubated for 24 h under standard conditions (37 °C at 5% CO_2_). The next day, a thallium-sensitive dye was added to the cells, followed by a 1-h incubation to ensure dye uptake. Test compounds were added to achieve a final concentration of 30 μM in 0.5% DMSO, and the cells were incubated for an additional 30 min at room temperature. Subsequently, a stimulation buffer containing thallium was added, and fluorescence measurements were taken using a FLIPR Tetra system. Data were collected every 3 s for 3 min (excitation of 470–495 nm and emission of 515–575 nm). Fluorescence intensity over time was analysed to calculate the area under the curve from which percentage inhibition was determined versus haloperidol at 100 μM (positive control) and vehicle (DMSO).

### α_2_A receptor purification and structure determination

Wild-type human α_2_AAR was cloned into a pVL1392 vector with an N-terminal FLAG tag. The construct was expressed in *Spodoptera frugiperda* (Sf9) insect cells using the BestBac system. Cells at a density of 4 × 10^6^ cells per millilitre were infected with virus and incubated for 48 h at 27 °C. The receptor was solubilized and purified by FLAG affinity chromatography and size-exclusion chromatography in the presence of 10 μM compound ‘4905. Monomeric peak fractions were concentrated and used for G protein complex formation. G_αo_G_β1γ2_ heterotrimeric G proteins were expressed in Hi5 insect cells (Expression Systems, 94-002S) and purified using Ni^2+^ affinity following detergent solubilization and dephosphorylation. The final α_2_AAR–G_αo_G_β1γ2_–scFv16 complex^72^ was assembled in the presence of ‘4905 and purified by size-exclusion chromatography. Cryo-EM grids were prepared using UltrAufoil R1.2/1.3 300-mesh grids and vitrified in liquid ethane. Data were collected on a Titan Krios G3 electron microscope equipped with a K3 direct electron detector. Image processing was performed using cryoSPARC, yielding a final reconstruction at approximately 2.8 Å resolution. Model building and refinement were performed with Protein Data Bank (PDB) 7EJ8 as a starting model using ChimeraX^73^, Phenix^74^ and Coot^75^. The final structures have been deposited in the PDB with accession codes 9PLO and 9PLN. Structural representations of protein–ligand complexes were generated using the PyMOL Molecular Graphics System v2.5.5 (Schrödinger).

### Mac1 purification and crystallization

Wild-type Mac1 protein (P4_3_ construct, residues 3–169) was expressed in *Escherichia coli* BL21(DE3) as an N-terminal His_6_-tagged construct and purified by Ni^2+^-affinity chromatography^71^. The His-tag was cleaved with TEV protease, followed by size-exclusion chromatography (Superdex 75) in 20 mM Tris-HCl (pH 7.5), 150 mM NaCl and 1 mM dithiothreitol. Purified protein was concentrated to 40 mg ml^−1^ for crystallization and stored at −80 °C.

Crystals were obtained by sitting-drop vapour diffusion in 28% PEG 3000 and 100 mM CHES (pH 9.5). Compounds (100 mM in DMSO) were added to crystal drops using an Echo 650 acoustic dispenser^76^ to a final concentration of 10 mM. Crystals were incubated at room temperature for 2–4 h and vitrified in liquid nitrogen without additional cryoprotection. X-ray diffraction data were collected at 100 K at the Advanced Light Source (beamline 8.3.1) using an X-ray wavelength of 0.88557 Å, and diffraction data were processed using XDS^77^ and Aimless^78^. Structures were determined to resolutions ranging from 0.97 to 1.02 Å. Ligands with low occupancy or conformational disorder were modelled using PanDDA^79^ and Coot^75^ and refined using phenix.refine^80^ as previously described^81^. The structure of ‘3184 was determined using a racemic preparation of the compound (‘9037). X-ray data collection and refinement statistics are shown in Extended Data Tables 1–4, as are the 32 PDB IDs. Structural representations of protein–ligand complexes were generated using the PyMOL Molecular Graphics System v2.5.5 (Schrödinger).

### Microsomal stability

Microsomal stability of compounds was evaluated using pooled mouse liver microsomes (M3000/lot #2010026, XenoTech) to estimate their metabolic stability and predict hepatic clearance. Each compound was incubated at 2 μM in a reaction mixture containing 0.42 mg ml^−1^ microsomal protein, phosphate buffer (100 mM, pH 7.4), MgCl_2_ (3.3 mM), NADPH (3 mM), glucose-6-phosphate (5.3 mM) and glucose-6-phosphate dehydrogenase (0.67 units per millilitre). Reactions were conducted at 37 °C in 96-well plates with shaking at 100 rpm. Samples were collected at five time points (0, 7, 15, 25 and 40 min), and reactions were quenched by adding five volumes of acetonitrile containing an internal standard. After centrifugation at 5,500 rpm for 5 min, the supernatants were analysed via high-performance liquid chromatography–tandem mass spectrometry (HPLC–MS/MS). The elimination rate constant (kel), half-life (*t*_1/2_) and intrinsic clearance (Clint) were calculated by plotting the natural logarithm of the remaining parent compound versus time. Stability was compared with reference standards such as imipramine and propranolol.

### Plasma protein binding

Plasma protein binding was measured using equilibrium dialysis with a 14-kDa molecular weight cut-off membrane in a 96-well HTD96b dialyser. Mouse plasma containing 1 μM test compound (0.005% DMSO and 1% acetonitrile) was placed in one chamber, and phosphate-buffered saline (PBS, pH 7.4) in the opposing chamber. The assembled plates were incubated at 37 °C with 5% CO_2_ and approximately 95% humidity, shaking at 250 rpm for 5 h to reach equilibrium. After incubation, aliquots from each chamber were mixed with equal volumes of the blank opposite matrix and processed with acetonitrile containing internal standard. Supernatants obtained after centrifugation were analysed by HPLC–MS/MS. The percentage of compound bound to plasma proteins was calculated using the peak area ratio in buffer to plasma compartments. Recovery and stability standards were included to ensure accuracy and reliability. Verapamil served as a reference control. Most compounds showed moderate binding, typically ranging between 75% and 85%.

### Plasma stability

Plasma stability was assessed in non-sterile mouse plasma (Li-heparin treated) at 1 μM concentration (final DMSO content was 0.005%). Incubations were carried out in aliquots of 60 μl each (two per time point) at 37 °C under 5% CO_2_ and high humidity (approximately 95%). The reactions were quenched with 240 µl of 90% acetonitrile containing an internal standard at 0, 20, 40, 60 and 120 min, followed by centrifugation at 6,000 rpm for 5 min. Supernatants were analysed via HPLC–MS/MS to determine the percentage of parent compound remaining at each time point. Data were plotted to calculate compound half-lives (*t*_1/2_). Reference compounds, verapamil and propantheline, were used as high and low stability controls, respectively. This assay is critical for identifying compounds susceptible to degradation by plasma esterases or hydrolytic enzymes, helping inform PK optimization strategies during lead selection.

### Thermodynamic solubility

Aqueous thermodynamic solubility was determined in PBS (pH 7.4) for 247 compounds using a shake-flask method followed by UV absorbance quantification. Dry powder compounds were dissolved in PBS to a theoretical concentration of 4 mM and incubated in duplicate at 25 °C for 4 h and 24 h with shaking. After incubation, samples were filtered using HTS 96-well filter plates. The filtrates were diluted twofold in acetonitrile with 4% DMSO for UV analysis. The incubation samples for charged molecules were additionally diluted tenfold with 50% acetonitrile–PBS with 2% final DMSO. Calibration curves (0–200 μM) were prepared in 50% acetonitrile–PBS (2% final DMSO). Absorbance was measured between 230 and 550 nm using a SpectraMax Plus microplate reader. Compound-specific absorbance maxima were used to calculate concentration using SoftMax Pro and Excel. The assay dynamic range was approximately 2–400 μM (about 20–4,000 μM for charged molecules), with values near the upper limit treated as semi-quantitative. Ondansetron was used as a reference compound. This method reflects equilibrium solubility under physiologically relevant conditions and helps to rank compounds for formulation feasibility.

### PAMPA-BBB

Passive blood–brain barrier (BBB) permeability was estimated using PAMPA (PAMPA-BBB) with a phospholipid-coated membrane simulating the brain endothelium. Test compounds (50 μM in Prisma HT buffer, pH 7.4, with 0.5% DMSO) were added to donor wells, whereas brain sink buffer was added to the acceptor wells. The donor and acceptor chambers were separated by a 0.45-µm filter membrane coated with brain polar lipids. Plates were incubated without agitation at room temperature for 4 h. Post-incubation, samples from both chambers, as well as a standard solution, were diluted with acetonitrile containing an internal standard. Apparent permeability coefficients (log Papp) were calculated based on peak area ratio. Clozapine and chlorpromazine were used as high-permeability controls, whereas ranitidine represented low permeability. The assay provides a high-throughput, non-cell-based method for estimating central nervous system exposure potential.

### Behavioural analyses

Male C57BL/6J mice 7–8 weeks of age were obtained from The Jackson Laboratory (JAX strain #000664) and housed five per cage under a standard 12–12-h light–dark cycle at 20.7 °C (69.3 °F) and 58% humidity. All animal procedures were approved by the University of California, San Francisco Institutional Animal Care and Use Committee (protocol #AN208219). Animals were randomly assigned to treatment and control groups. For behavioural experiments, mice were initially placed together in a cage and allowed to move freely for a few minutes; each mouse was then randomly selected, injected with compound or vehicle, and placed in a separate cylinder before testing. The experimenter assessing behaviour was different from the experimenter administering injections and placing mice into the cylinders; experimenters were therefore blinded to treatment. Behavioural experiments were conducted in two independent cohorts. Mice were habituated individually in Plexiglas enclosures for 1 h before testing. Compounds were administered subcutaneously 30 min before behavioural assessment, and, where applicable, the α_2_AAR antagonist atipamezole (2 mg kg^−1^, intraperitoneally) was given 15 min before compound injection. Tail-flick latency was measured by immersing the distal third of the tail in a 50 °C water bath and recording the withdrawal time. For the neuropathic pain model, SNI was performed under isoflurane anaesthesia by ligating and transecting two of the three branches of the sciatic nerve, sparing the sural nerve. Mechanical thresholds were assessed 7–14 days post-surgery using von Frey filaments and the up–down method, and values were normalized to the baseline of each animal. Thermal nociception was evaluated using a 55 °C hotplate, and the latency to nocifensive behaviour (paw lick or jump) was recorded with a cut-off of 45 s to prevent tissue damage. Behavioural data are summarized as group size (*n*), mean and s.d. (Supplementary Table 13). Statistical analyses were performed in GraphPad Prism. For the SNI experiment (Fig. 5f), we used two-way ANOVA followed by Tukey’s multiple-comparisons test. The hotplate assay (Fig. 5g) was analysed using the Friedman test followed by Dunn’s multiple-comparisons test. For the tail-flick assays, the 4905 dose–response (Fig. 5c) was analysed by two-way ANOVA followed by Dunnett’s multiple-comparisons test against vehicle (control), whereas the PS75 and 3629 dose groups (Fig. 5d,e) were analysed using the Kruskal–Wallis test followed by Dunn’s multiple-comparisons test against vehicle. The Kruskal–Wallis and Friedman tests were nonparametric; Prism calculated two-sided, multiplicity-adjusted *P* values for the Dunn comparisons (Supplementary Table 13). For sample size, we did not perform formal a priori power calculations. Instead, group sizes were guided by our previous experience with these assays and by precedent in the literature. This approach may limit sensitivity to small effects. Raw data for the animal assays underlying Fig. 5c–g are provided in the Source Data file.

### Reporting summary

Further information on research design is available in the Nature Portfolio Reporting Summary linked to this article.

## Online content

Any methods, additional references, Nature Portfolio reporting summaries, source data, extended data, supplementary information, acknowledgements, peer review information; details of author contributions and competing interests; and statements of data and code availability are available at https://doi.org/10.1038/s41586-026-11013-5.

## Supplementary information

Supplementary Information Supplementary Figures 1–9, Supplementary Methods, and titles and legends for Supplementary Tables

Reporting Summary

Supplementary Tables Supplementary Tables 1–13

Peer Review file

## Source data

Source Data Figs. 2, 3, 4, 5 and Source Data Extended Data Figs. 1, 2, 3, 5

## Figures and Tables

**Extended Data Table 1:**
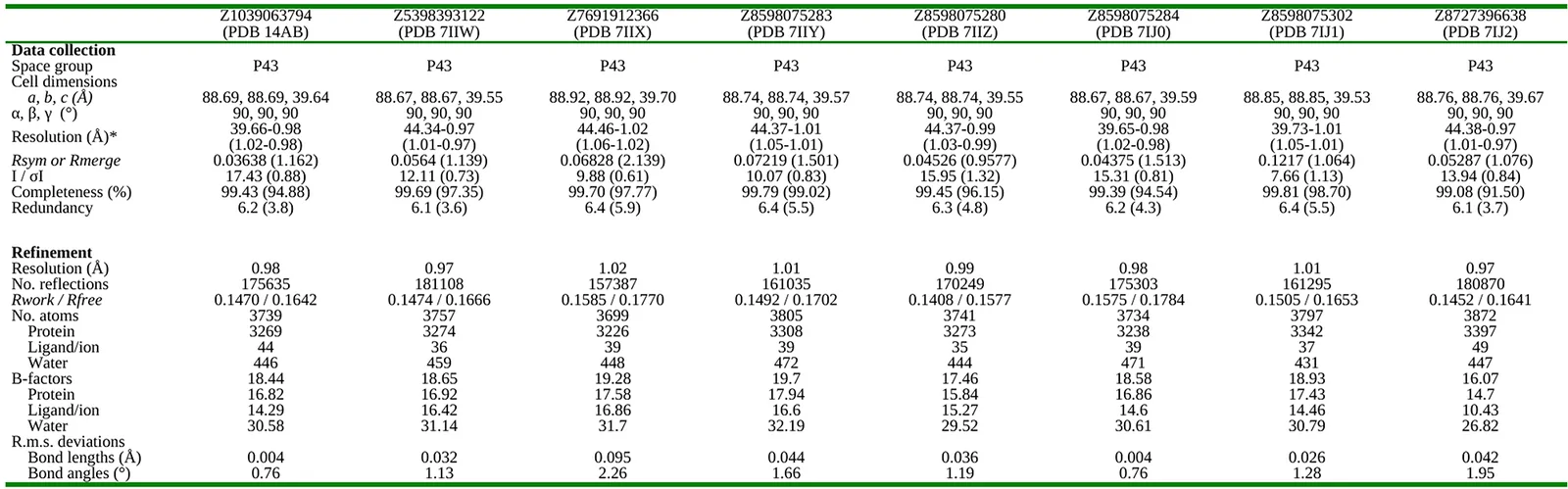
Data collection and refinement statistics (molecular replacement)

*Values in parentheses are for the highest-resolution shell.

**Extended Data Table 2:**
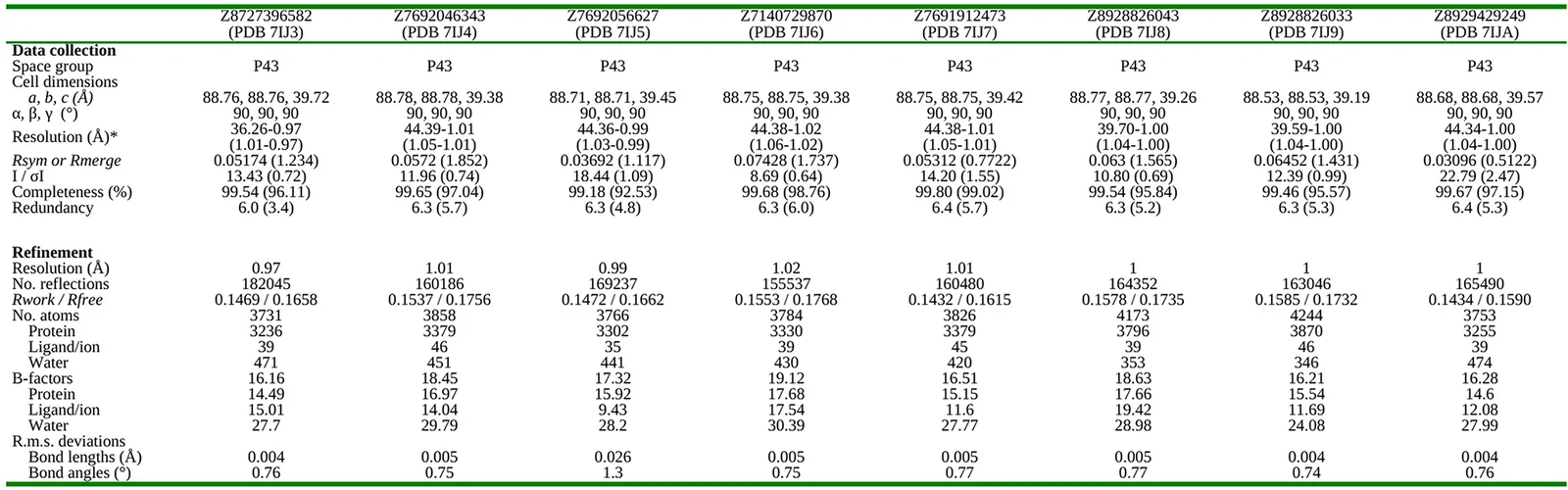
Data collection and refinement statistics (molecular replacement)

*Values in parentheses are for the highest-resolution shell.

**Extended Data Table 3:**
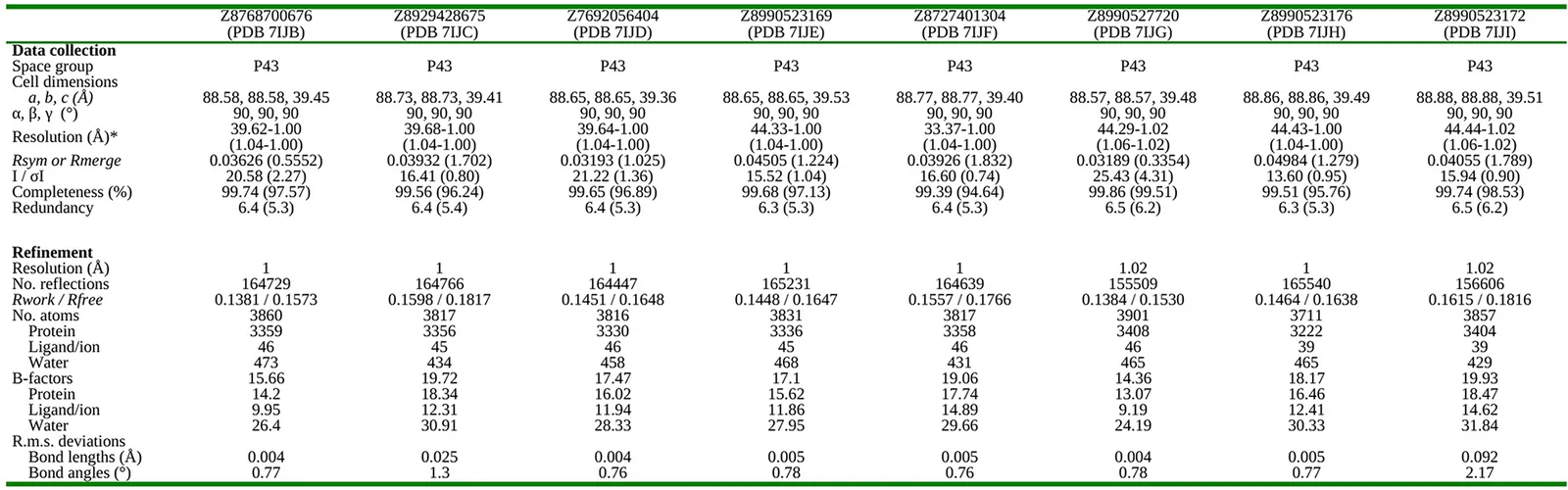
Data collection and refinement statistics (molecular replacement)

*Values in parentheses are for the highest-resolution shell.

**Extended Data Table 4:**
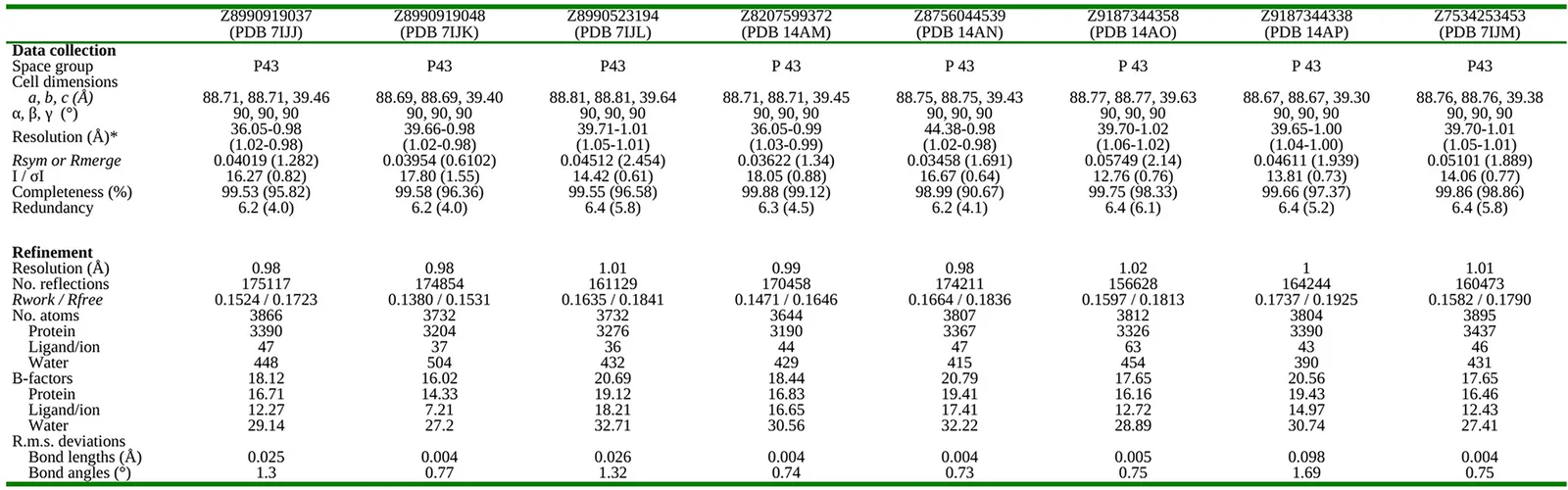
Data collection and refinement statistics (molecular replacement)

*Values in parentheses are for the highest-resolution shell.

**Extended Data Table 5:**
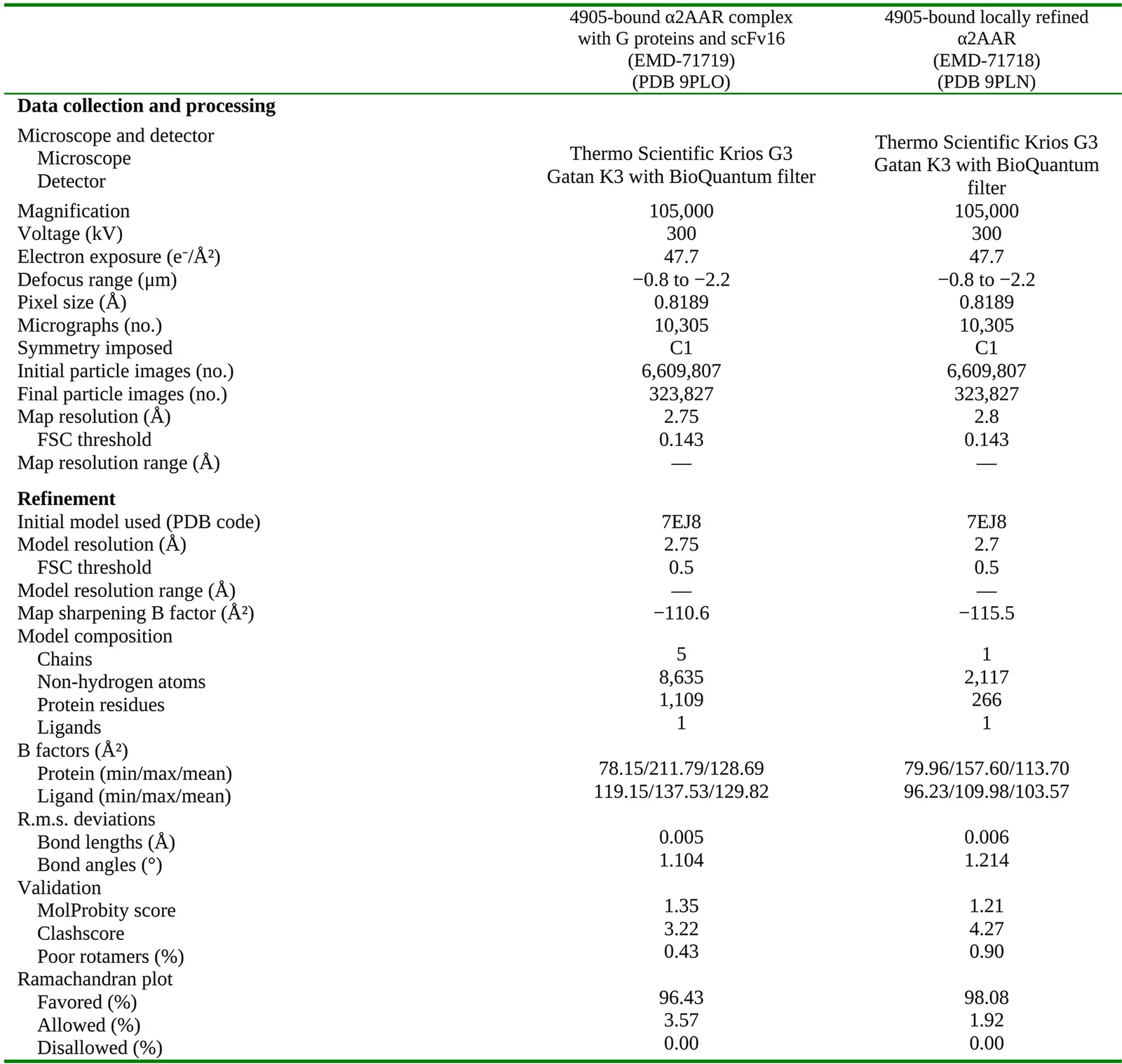
Cryo-EM data collection, refinement and validation statistics

Dashes indicate values that were not reported.

## Data Availability

All primary data are available through this article and its Extended Data and Supplementary Information. The cryo-EM structures and maps have been deposited in the PDB and Electron Microscopy Data Bank, respectively, under the accession codes 9PLO and EMD-71719 (the ‘4905-bound α_2_AAR complex with G proteins and scFv16), and PDB 9PLN and EMD-71718 (the ‘4905-bound locally refined α_2_AAR structure). The Mac1 crystal structures and supporting structure factors have been deposited in the PDB under the accession codes 14AB, 7IIW, 7IIX, 7IIY, 7IIZ, 7IJ0, 7IJ1, 7IJ2, 7IJ3, 7IJ4, 7IJ5, 7IJ6, 7IJ7, 7IJ8, 7IJ9, 7IJA, 7IJB, 7IJC, 7IJD, 7IJE, 7IJF, 7IJG, 7IJH, 7IJI, 7IJJ, 7IJK, 7IJL, 14AM, 14AN, 14AO, 14AP and 7IJM. Source data are provided with this paper.

## Code availability

ZINC tools used in the selection of the small-perturbation analogues are openly available (https://zinc22.docking.org).
