# Supplementary material for: Development of a random background to understand ligand optimization
Source: Nature. 2026 Sep 16;658(8138):1341–9. doi: 10.1038/s41586-026-11013-5 (PMC13716815; doi:10.1038/s41586-026-11013-5)
Supplement: Supplementary file 1 [file 41586_2026_11013_MOESM1_ESM.pdf]

nature portfolio

https://doi.org/10.1038/s41586-026-11013-5

Supplementary information

Development of a random background to understand ligand optimization

In the format provided by the
authors and unedited

Supplementary Information

Development of a Random Background to Understand Ligand Optimization

Xinyu Xu[1†], Olivier Mailhot[1†], Galen J. Correy[2†], Xi-Ping Huang[3†], Joao M. Braz[4], Da Shi[5], Karthik Srinivasan[1], Kara Zielinski[2], Yuliia Holota[6], Yuliia Kuziv[6], Christos Iliopoulos-Tsoutsouvas[7], Nathan D. Levinzon[8], Yagmur U. Doruk[9], Moira M. Rachman[1], Morgan E. Diolaiti[9], Maisie G. V. Stevens[9], Fangyu Liu[1], Katie L. Holland[1], Harald Hübner[10], Jing Wang[3], Yujin Wu[1], Alan Ashworth[9], Alexandros Makriyannis[7], Yuqi Zhang[5], Yurii S. Moroz[11], Peter Gmeiner[10], Robert Abel[5], Aashish Manglik[1], Allan I. Basbaum[4], Bryan L. Roth[3*], James S. Fraser[2*], & Brian K. Shoichet[1*]

† Contributed equally.

* Corresponding authors: bryan_roth@med.unc.edu; jaimefraser@gmail.com; bshoichet@gmail.com

[1]Department of Pharmaceutical Chemistry, University of California, San Francisco, San Francisco, CA 94143, USA.
[2]Department of Bioengineering and Therapeutic Sciences, University of California, San Francisco, San Francisco, CA 94158, USA.
[3]Department of Pharmacology, NIMH Psychoactive Drug Screening Program, School of Medicine, University of North Carolina at Chapel Hill, Chapel Hill, NC 27599, USA.
[4]Department of Anatomy, University of California, San Francisco, San Francisco, CA 94143, USA.
5Schrödinger, Inc., San Diego, CA, USA; Schrödinger, Inc., New York, NY, USA.
6Bienta, Winston Churchill St. 78, 02094 Kyiv, Ukraine.
7Center for Drug Discovery, Department of Pharmaceutical Sciences, Northeastern University, Boston, MA, USA.
[8]Department of Medicinal Chemistry, College of Pharmacy, University of Utah, 2000 East 30 South Skaggs 306, Salt Lake City, Utah 84112, United States.
9Helen Diller Family Comprehensive Cancer Center, University of California, San Francisco, CA 94158, USA.
[10]Department of Chemistry and Pharmacy, Medicinal Chemistry, Friedrich-Alexander-Universität Erlangen-Nürnberg, Erlangen, Germany.
[11]Chemspace LLC, Kyiv 02094, Ukraine; Taras Shevchenko National University of Kyiv, Kyiv 01601, Ukraine; Enamine Ltd., Kyiv 02094, Ukraine.

Contents

Supplementary Fig. 1 | Sample size required to constrain the "random background" success rate. 3
Supplementary Fig. 2 | Dose-response curves for all activity assays 4
Supplementary Fig. 3 | Representative concentration-response curves and the corresponding Z values 12
Supplementary Fig. 4 | Receptor expression levels for α2A, CB2, and MOR (relative and absolute measurements) 13
Supplementary Fig. 5 | cAMP responses at different receptor expression levels 14
Supplementary Fig. 6 | Correlation between parent compound or binding-pocket properties and affinity/potency fold change 15
Supplementary Fig. 7 | Pharmacokinetic profiles of 29 individual analogs with at least tenfold affinity/potency improvement 16
Supplementary Fig. 8 | Distribution and cumulative effects of atom modifications on molecular properties... 17
Supplementary Fig. 9 | PTX-treated cAMP responses and structural rationale for ligand-specific Gs coupling by 4905 18
Supplementary Methods | Chemical synthesis and characterization 19

Supplementary Fig. 1 | Sample size required to constrain the "random background" success rate.

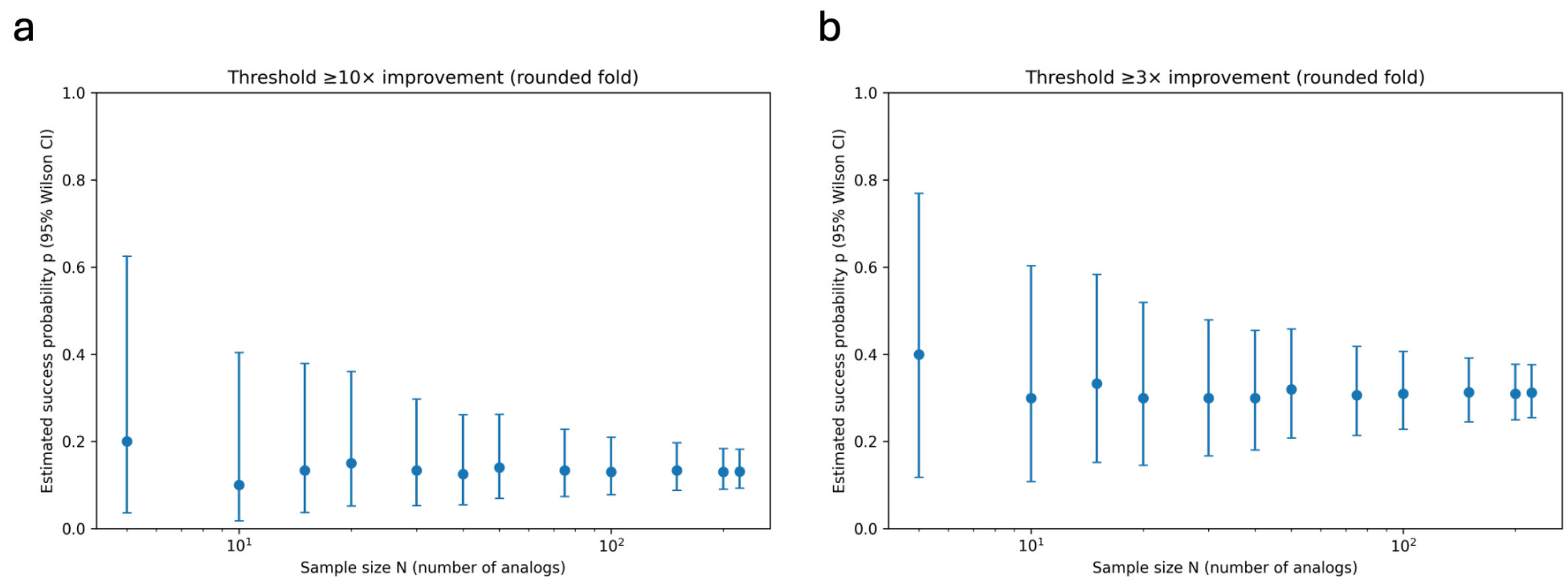

Each analog was treated as a Bernoulli trial for a threshold outcome (success = integer-rounded fold improvement ≥10× in panel a; ≥3× in panel b). Points show the estimated success frequency (p^=k/N) at each sample size N; error bars indicate two-sided 95% binomial confidence intervals computed using the Wilson score method. As N increases, the confidence intervals narrow, showing that pooled analyses with large N provide a well-constrained estimate of the background probability, whereas per-parent sample sizes (typically much smaller) yield wide and often uninformative intervals.

Supplementary Fig. 2 | Dose-response curves for all activity assays

SERT parent1 2686

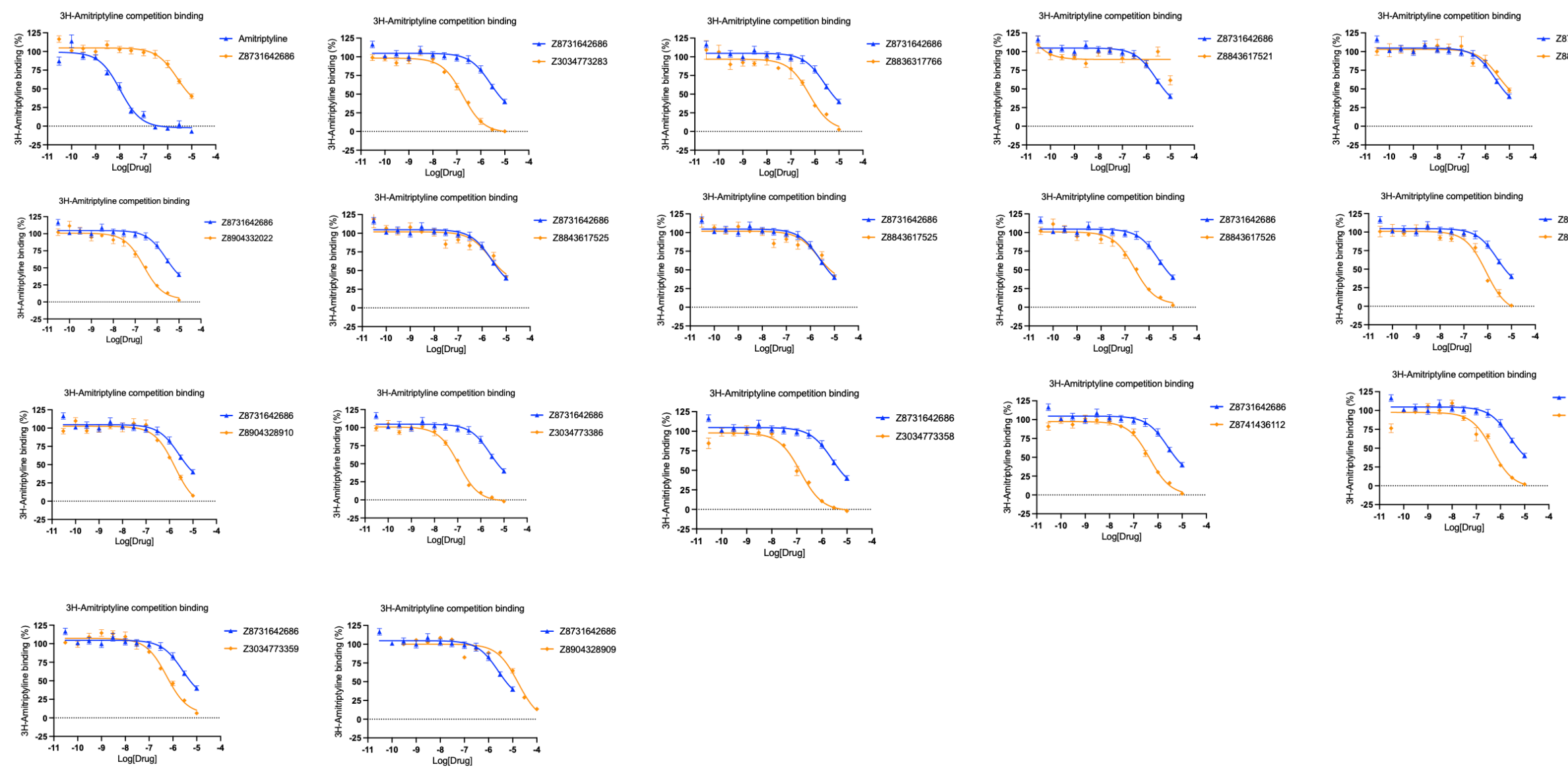

SERT parent2 2480

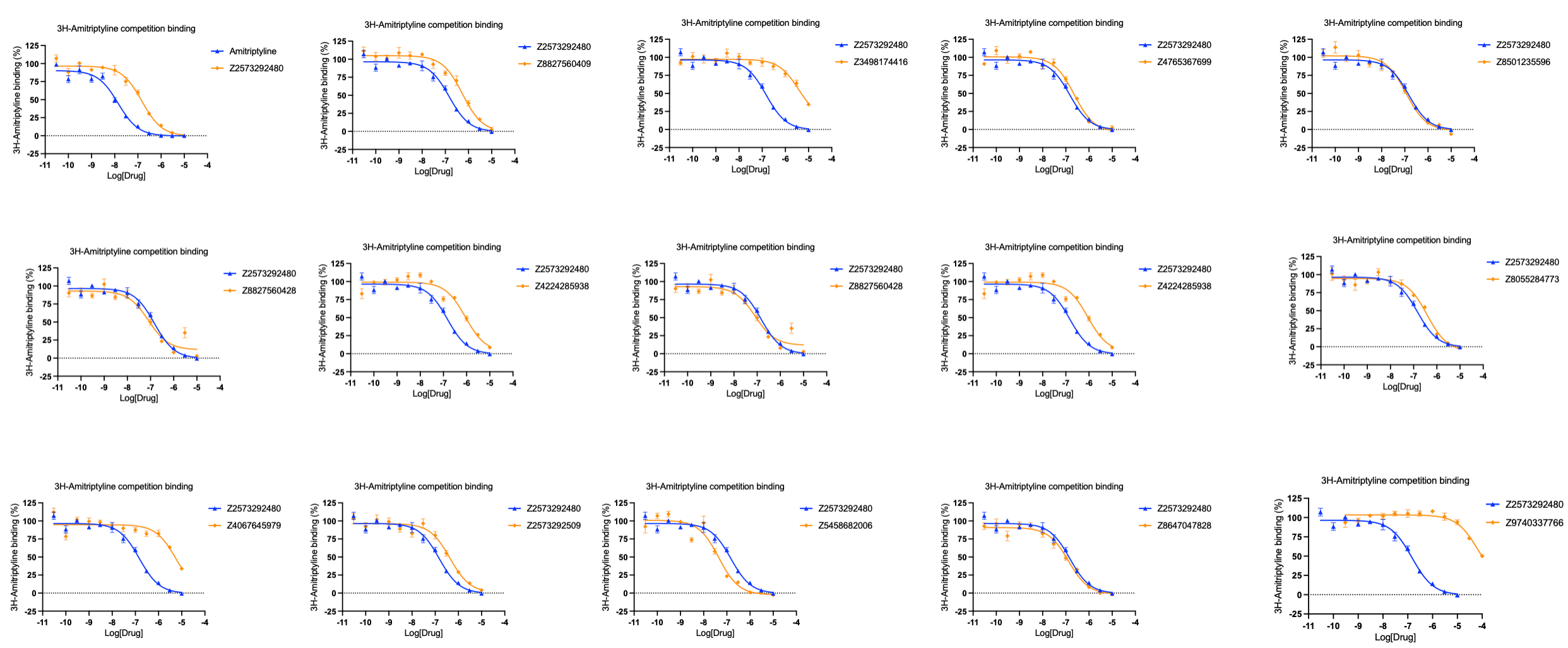

SERT parent3 8218

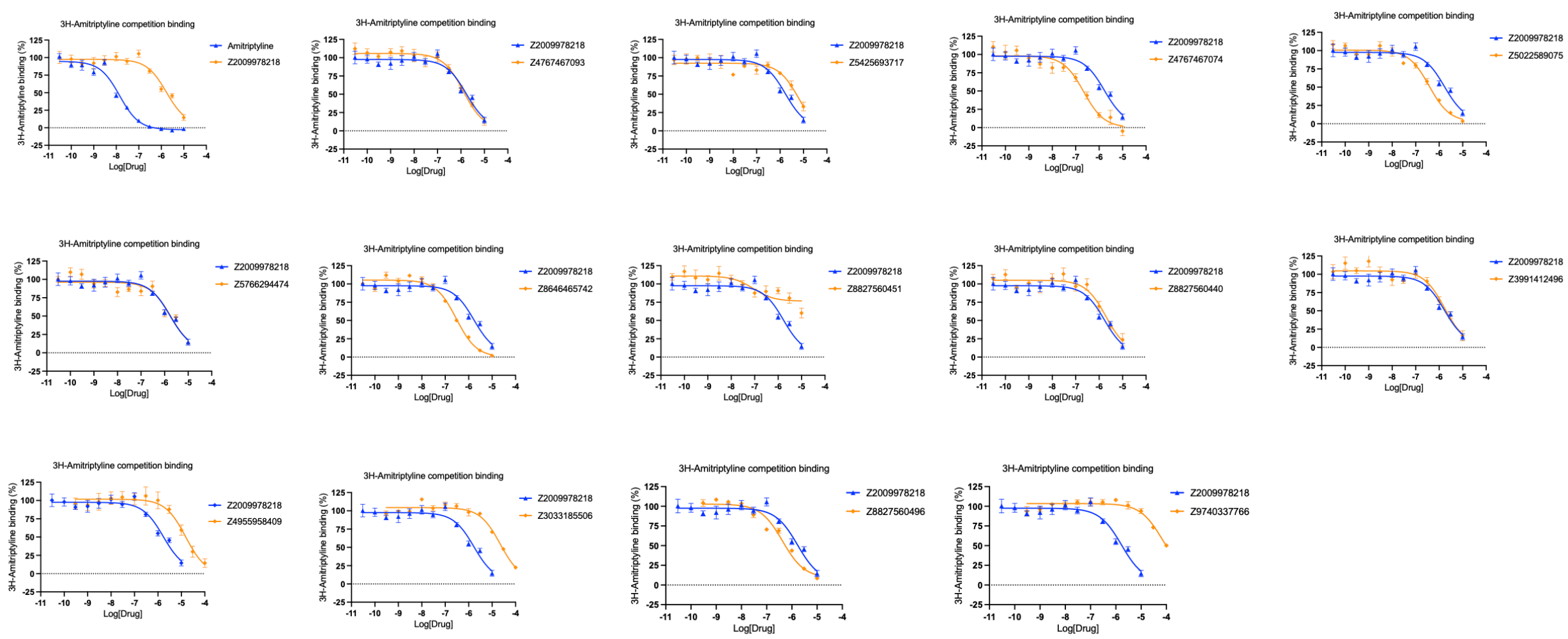

SERT parent4 2509

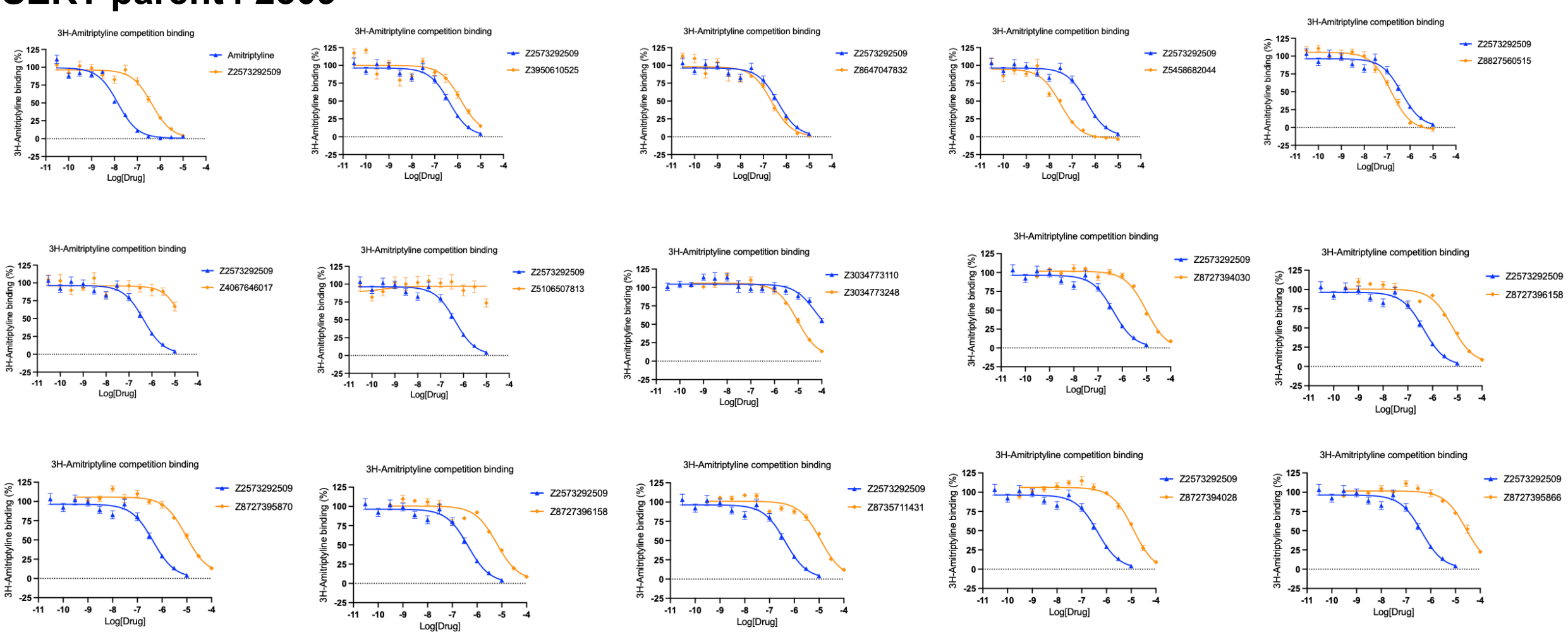

SERT parent5 3110

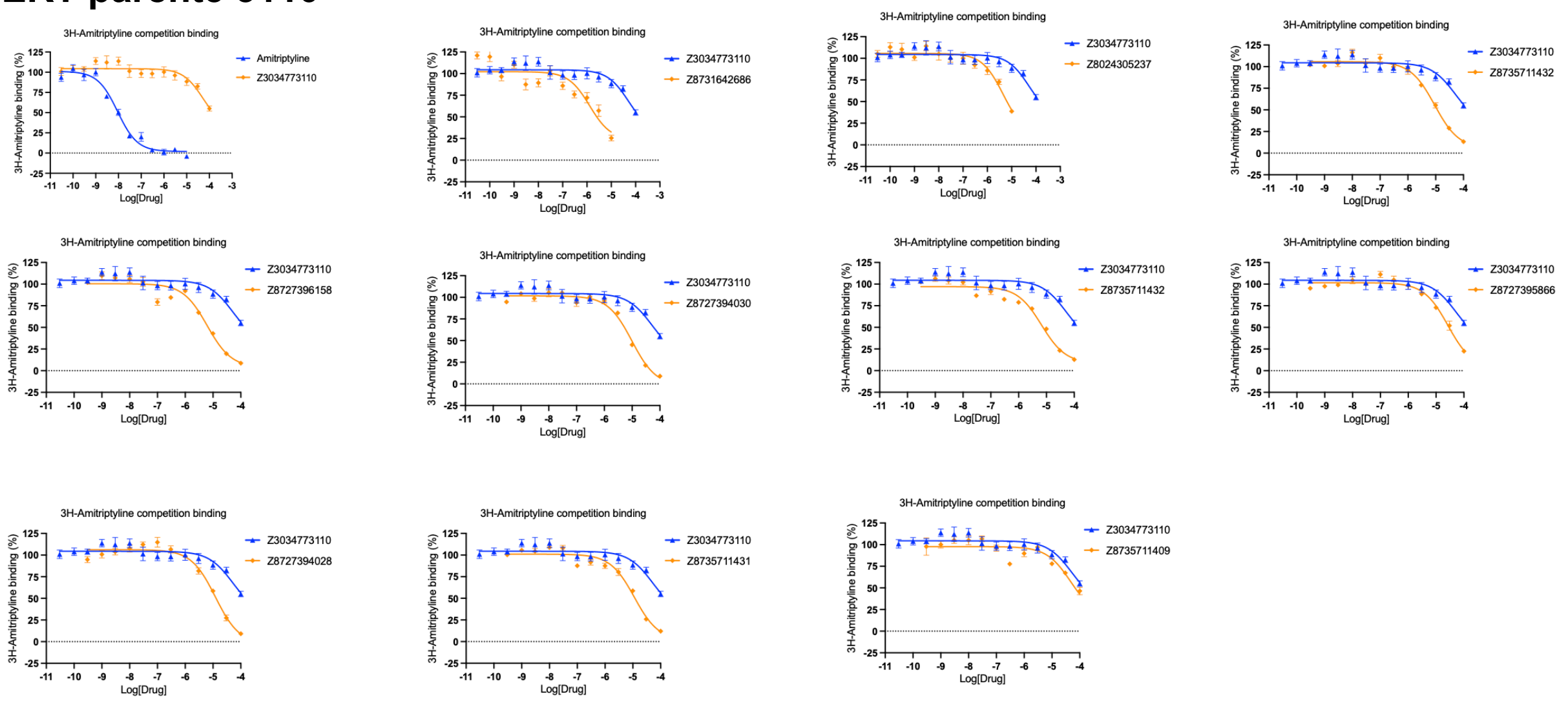

SERT parent6 7399

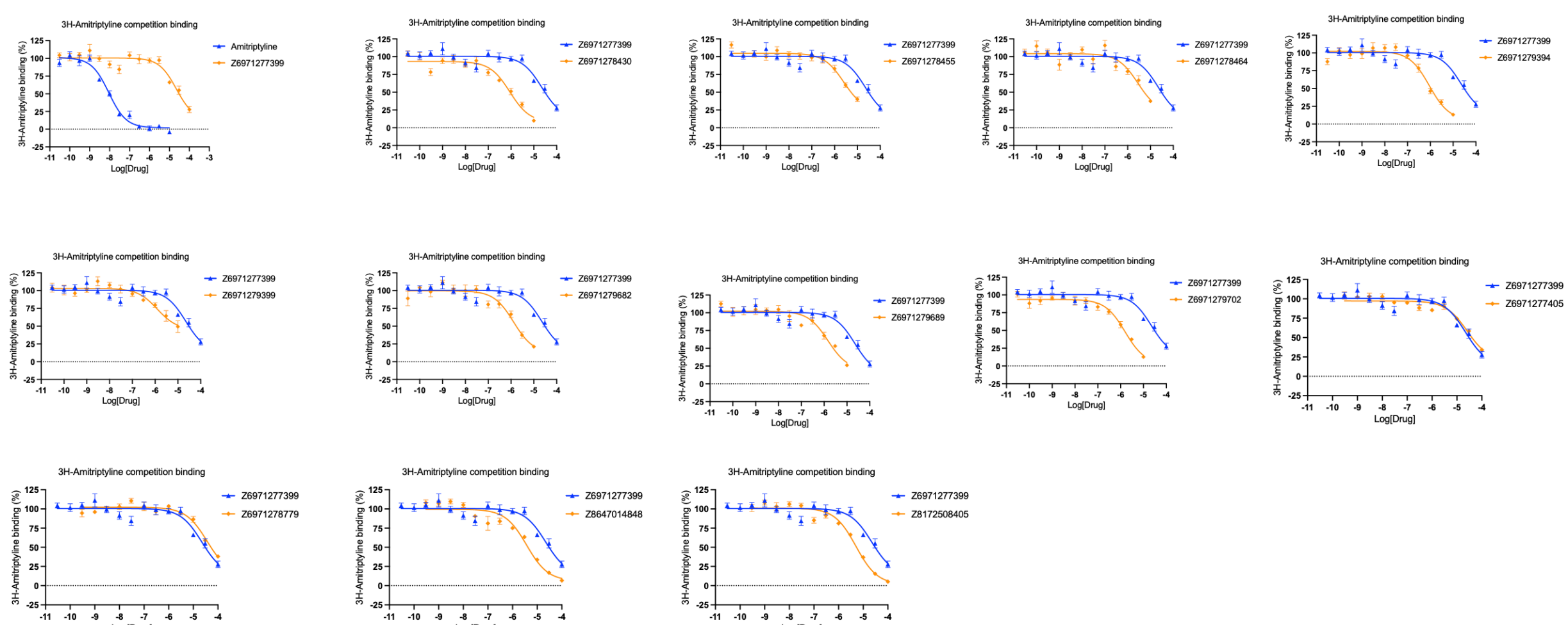

MOR parent 8716

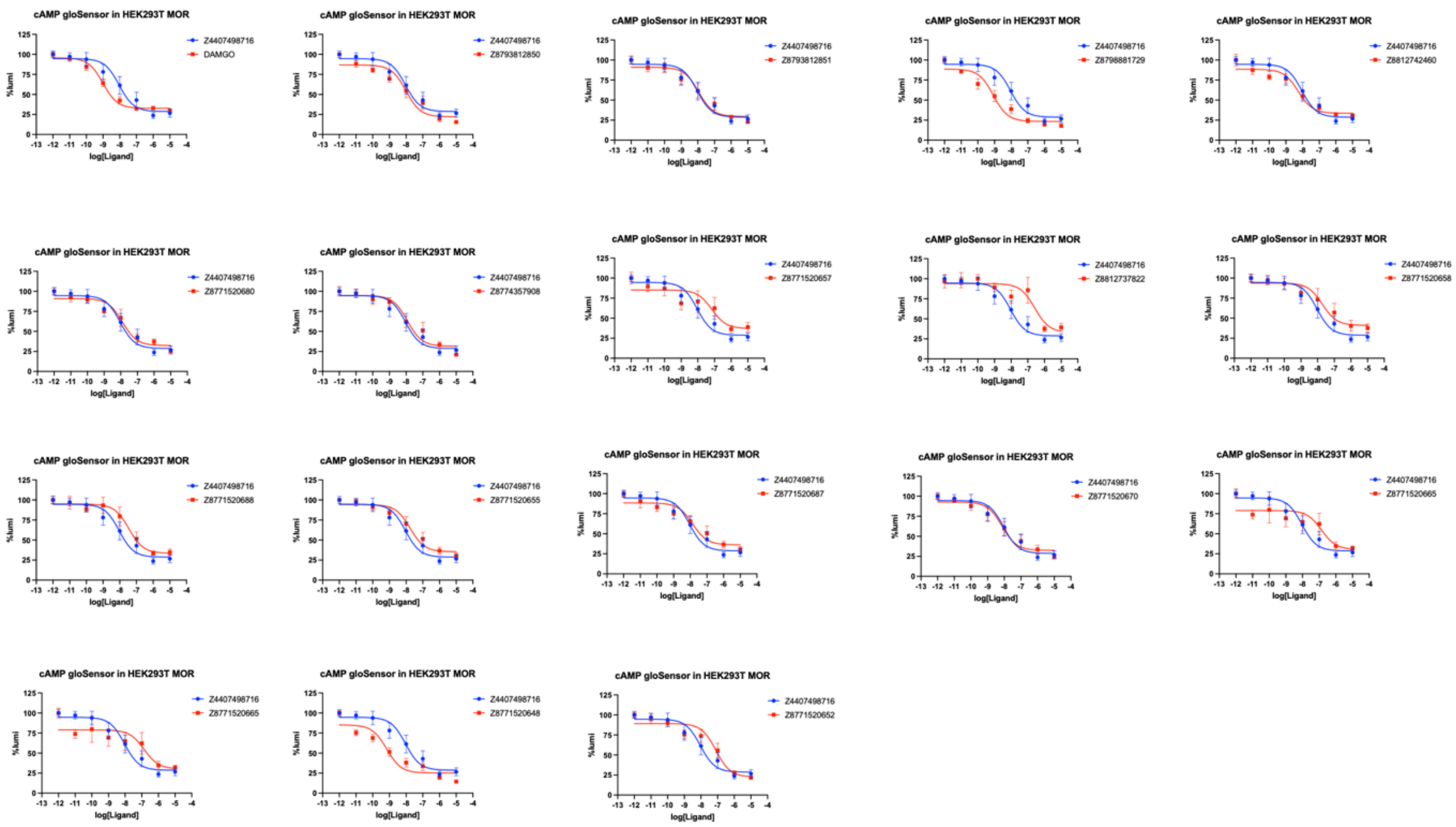

Alpha2 parent1 3629

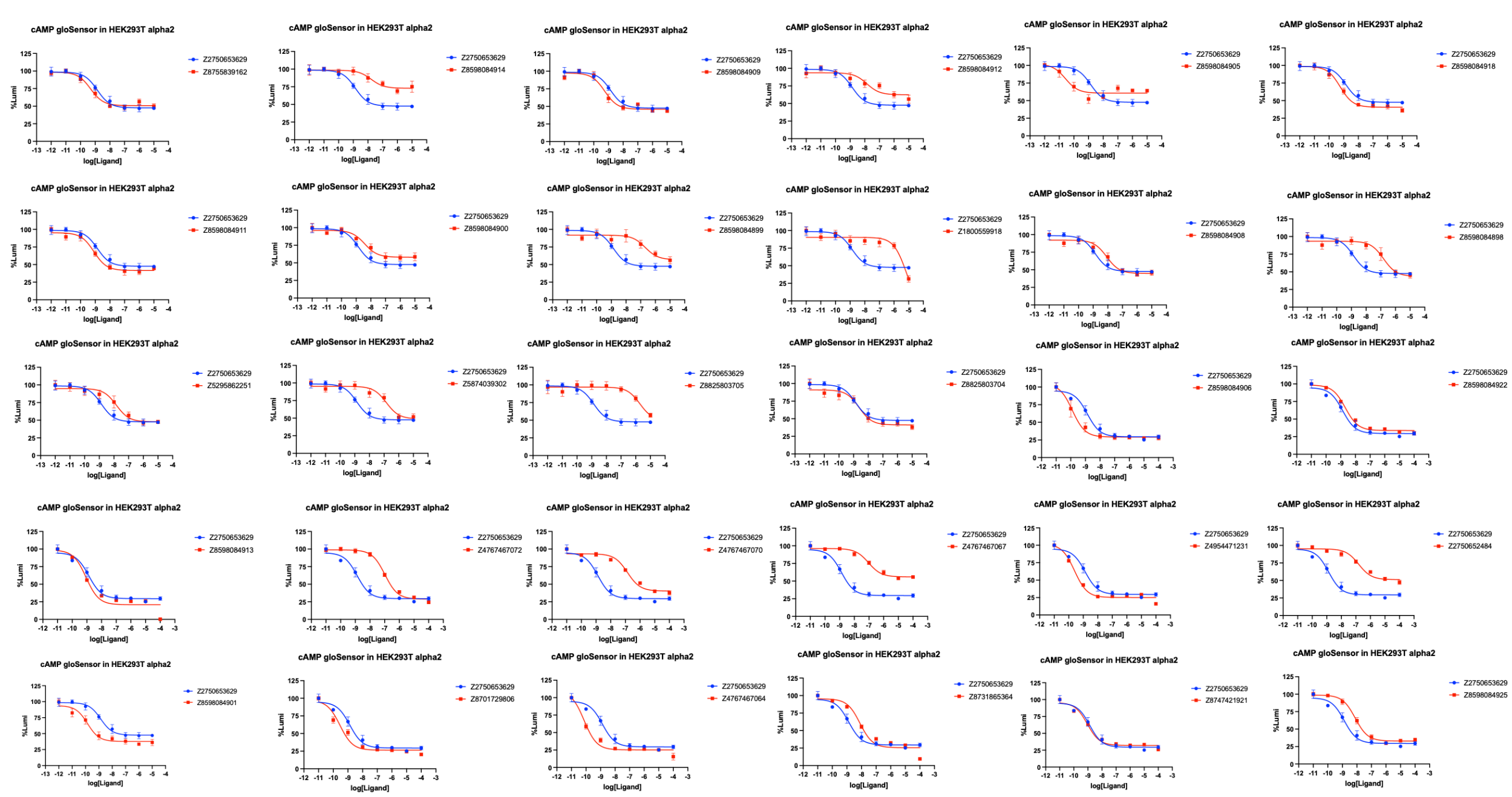

Alpha2 parent2 0014

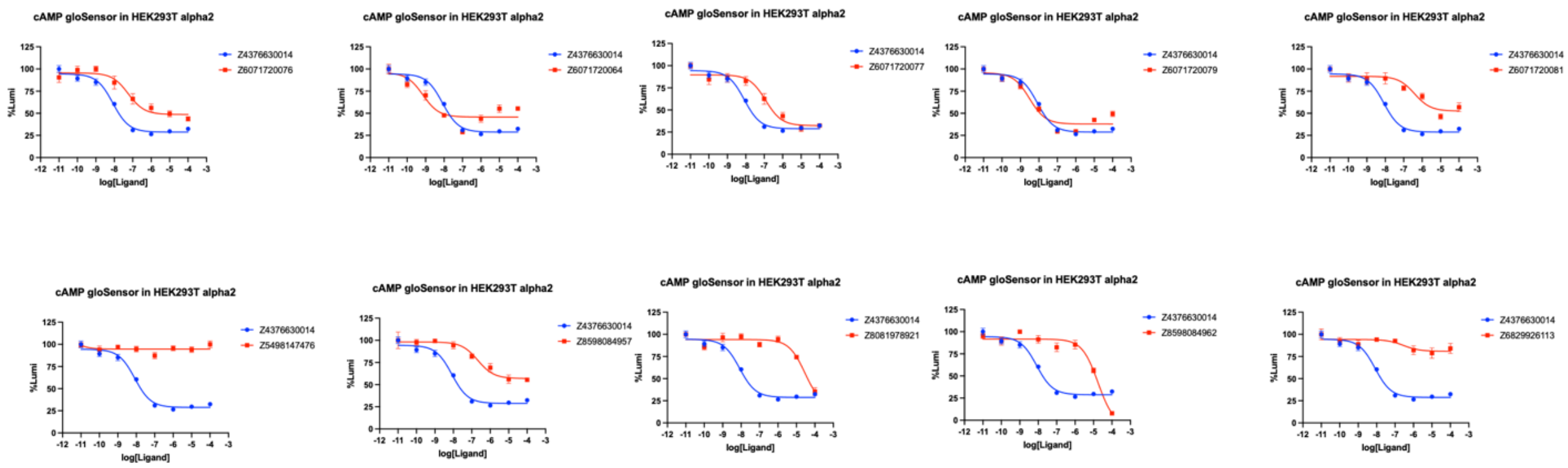

Alpha2 parent3 5870

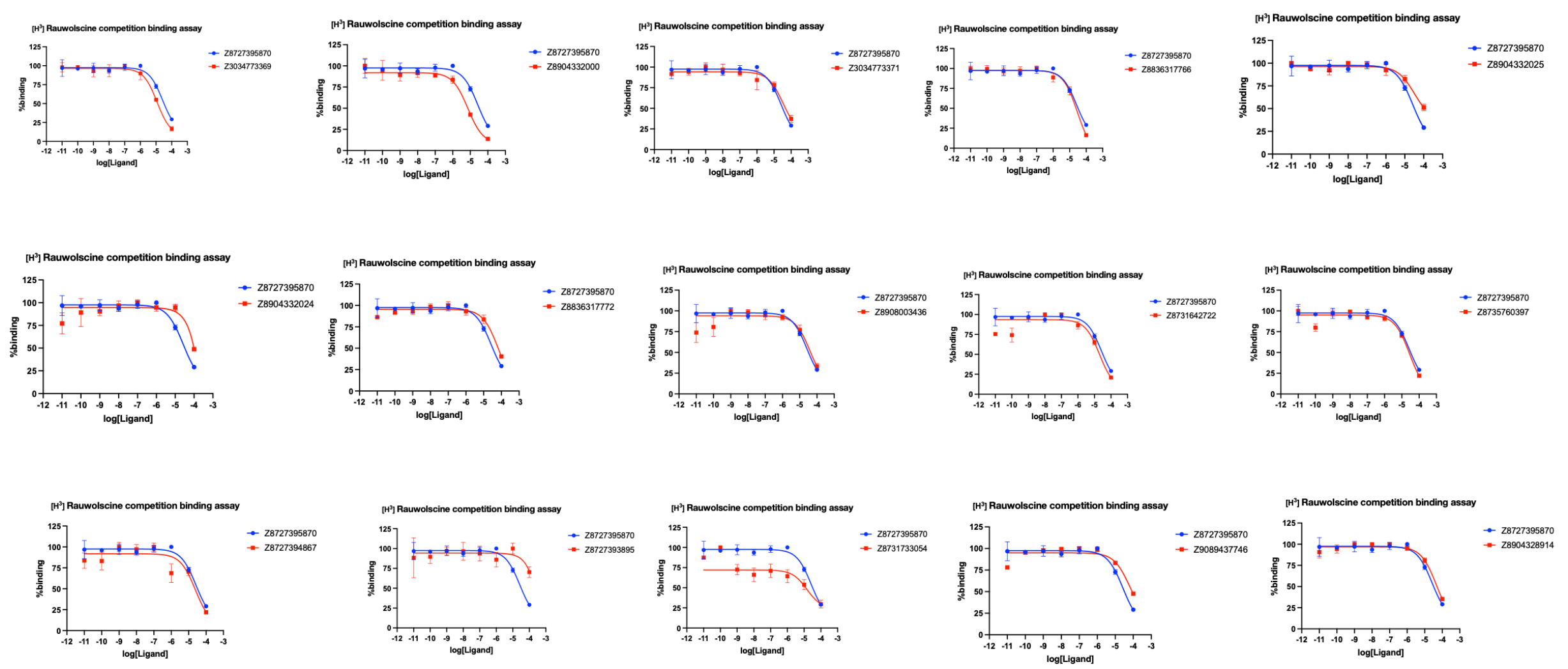

Alpha2 parent4 3248

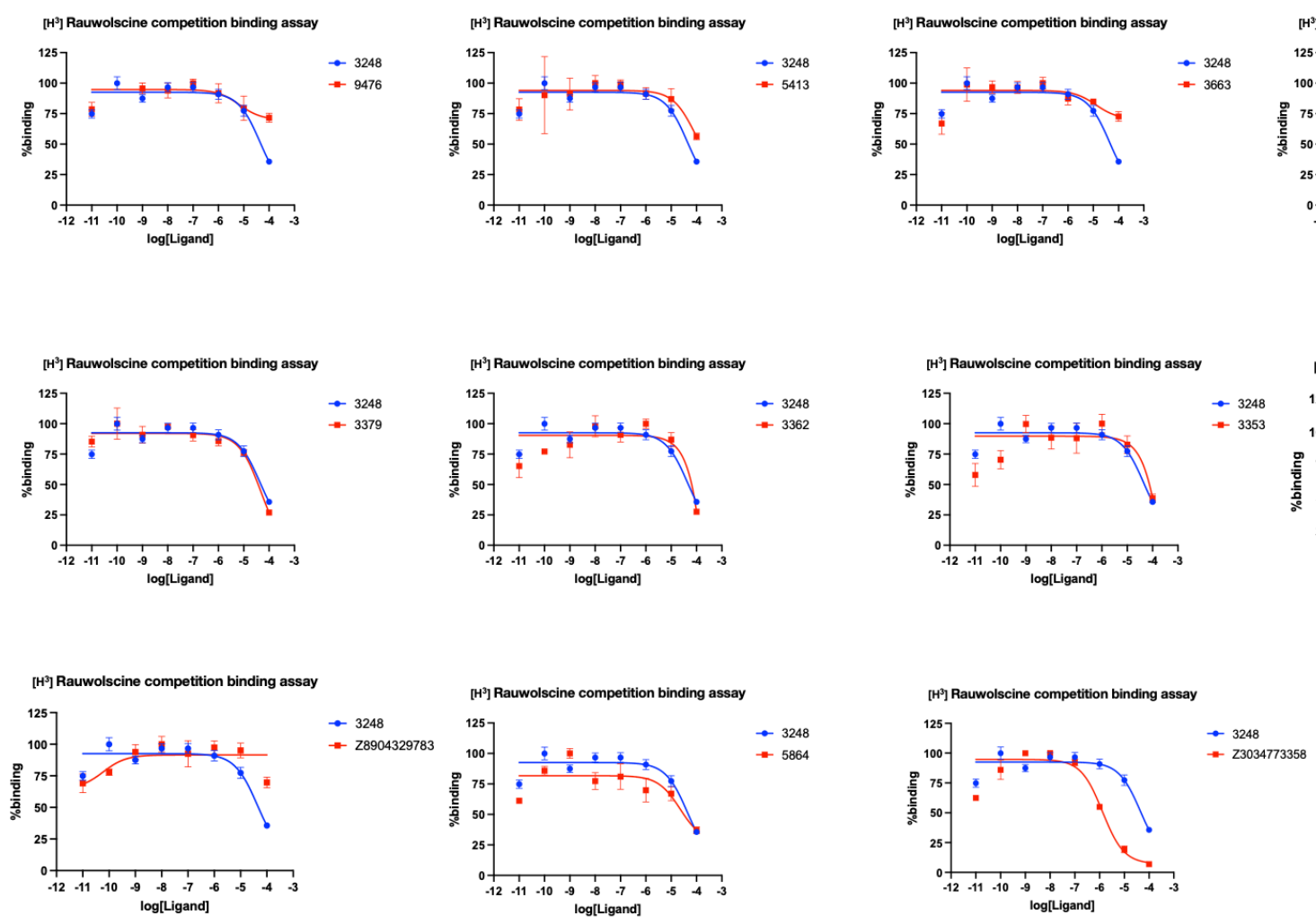

CB2 parent1 5903

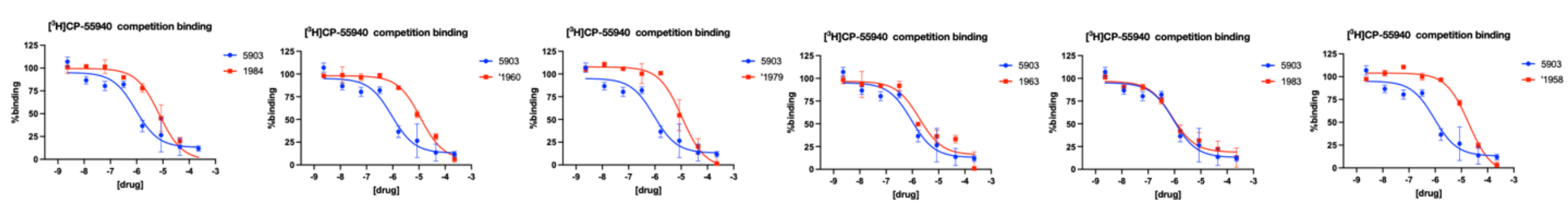

CB2 parent2 6138

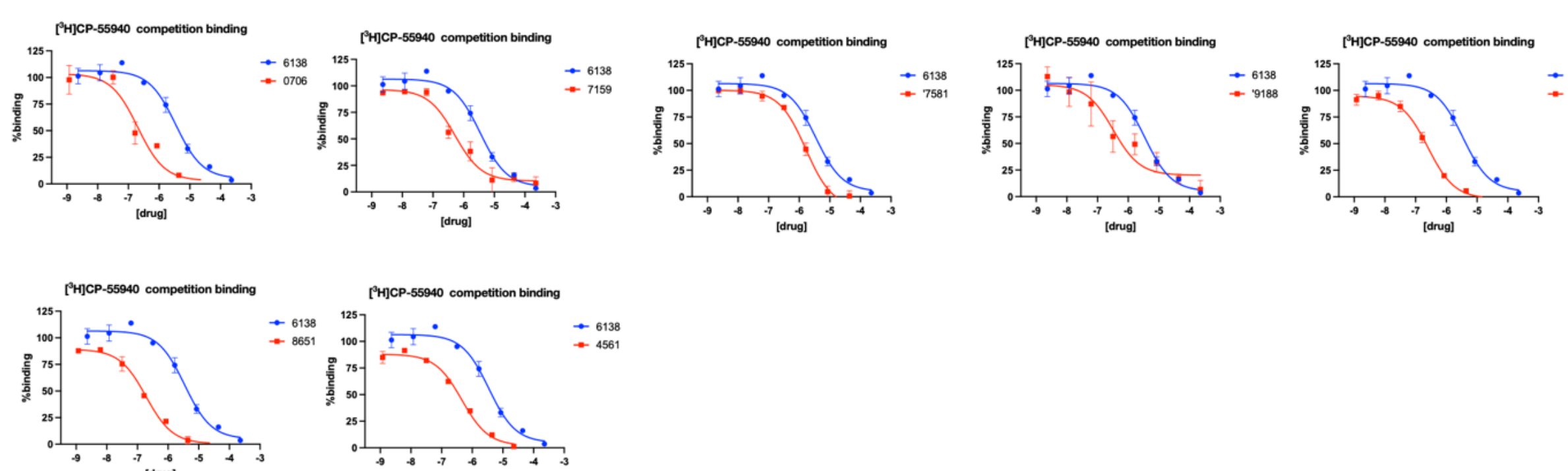

CB2 parent38918

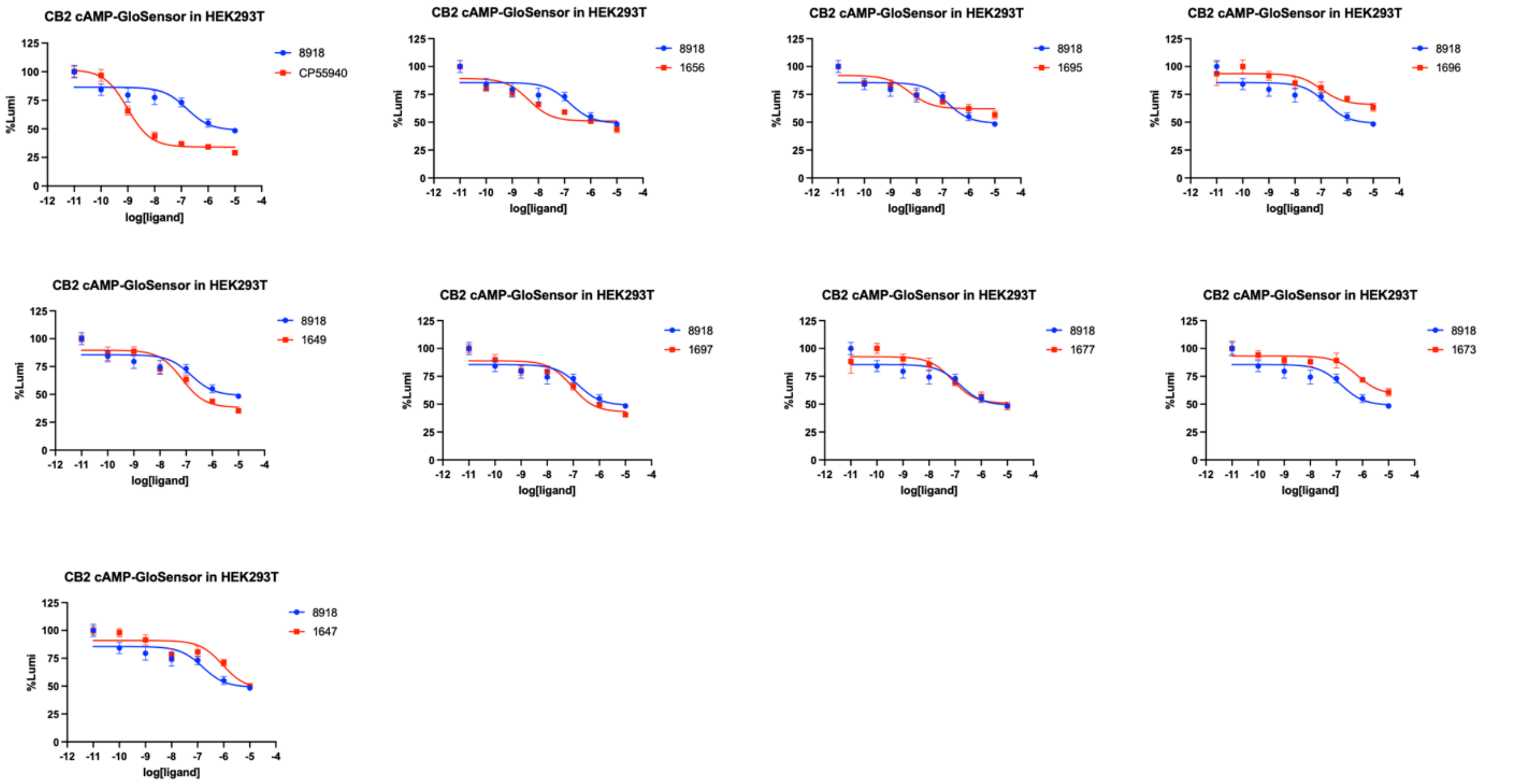

MAC1 parent1 3122

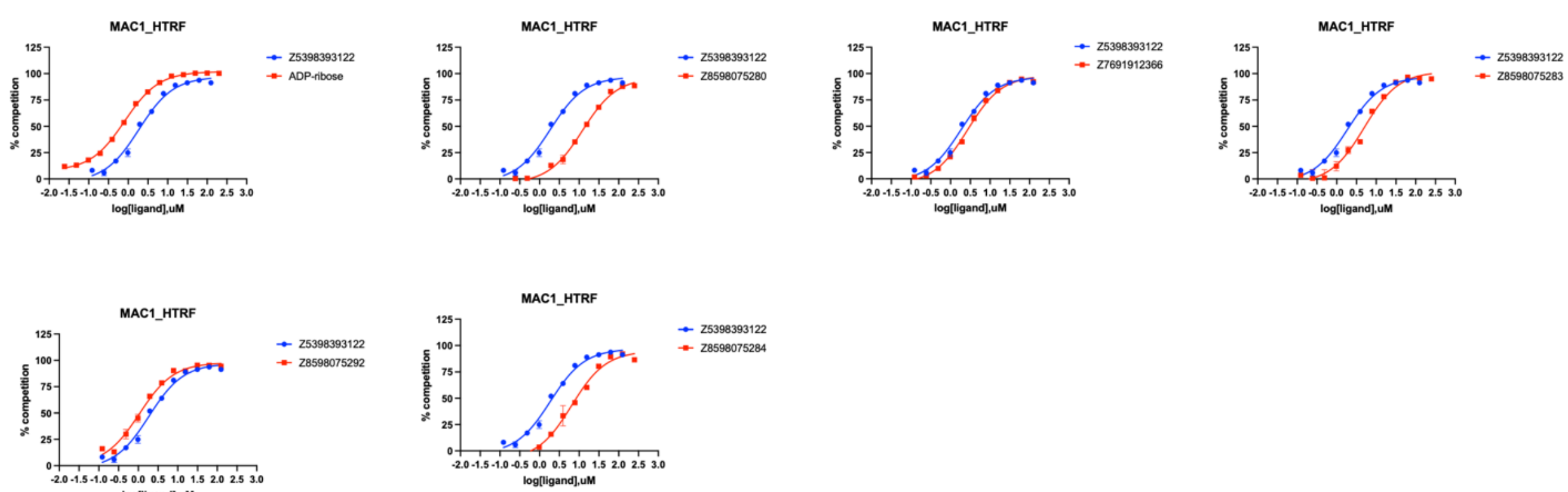

MAC1 parent2 3453

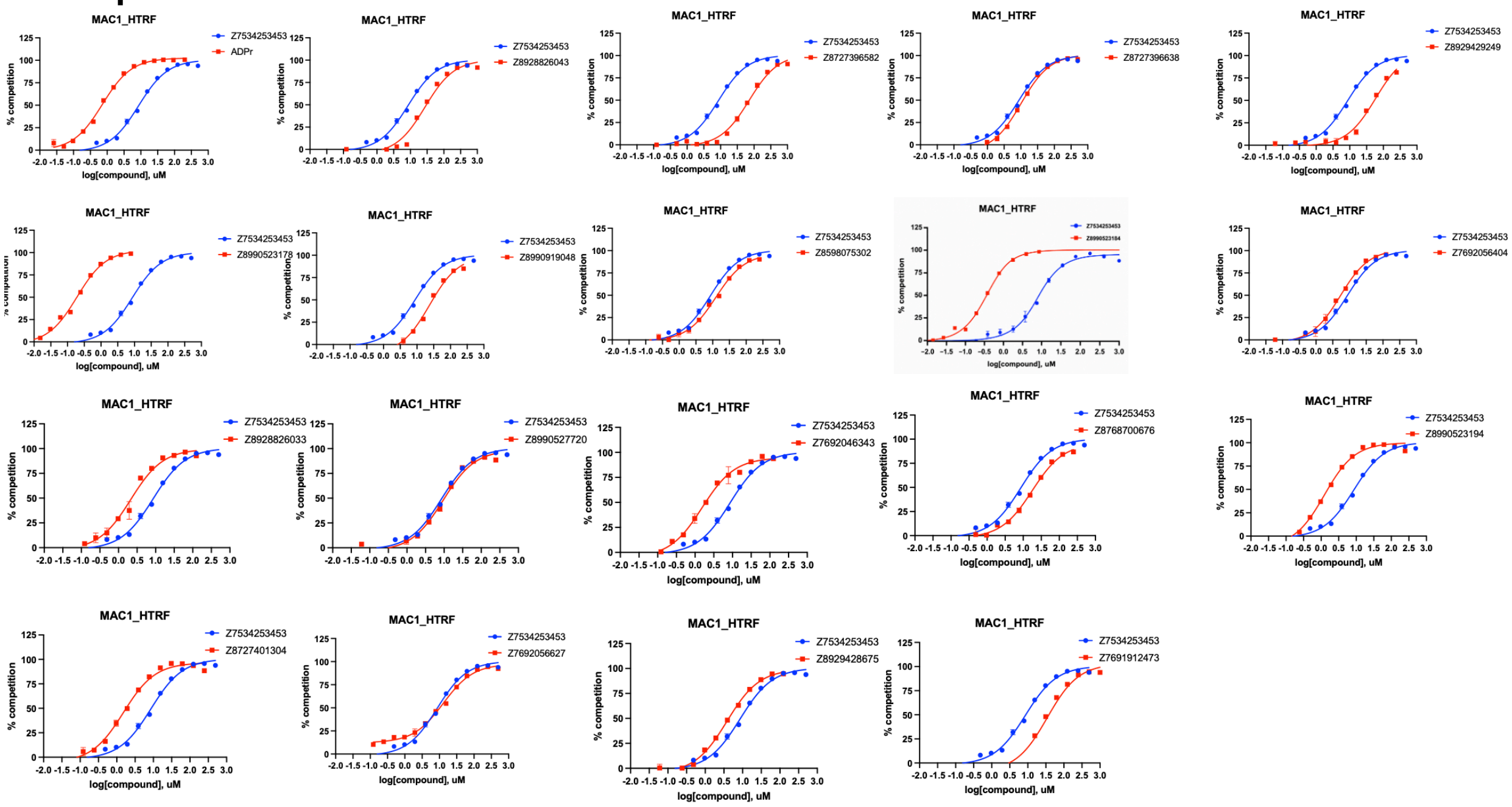

MAC1 parent3 3794

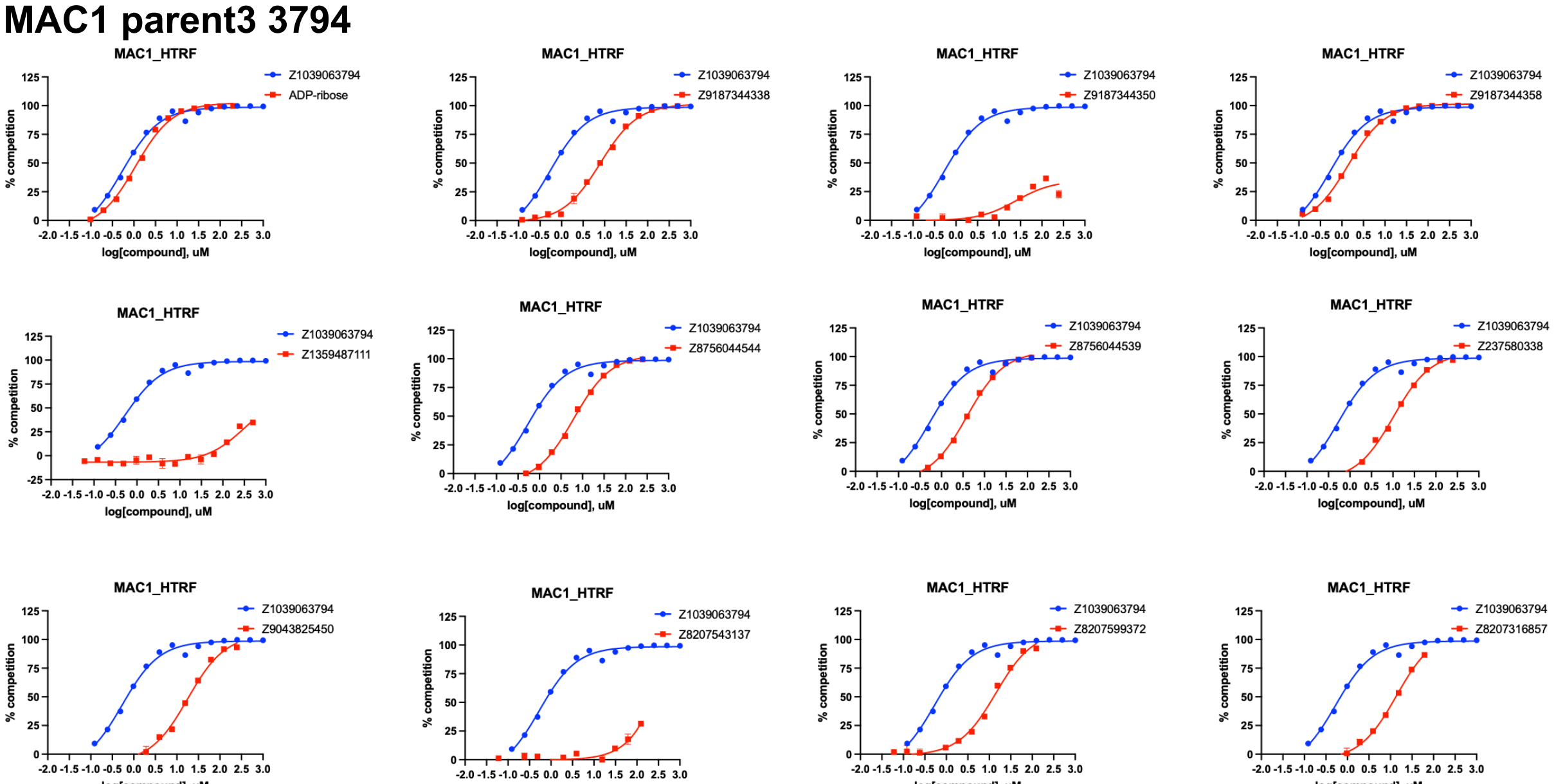

Supplementary Fig. 3 | Representative concentration–response curves and the corresponding Z values

a

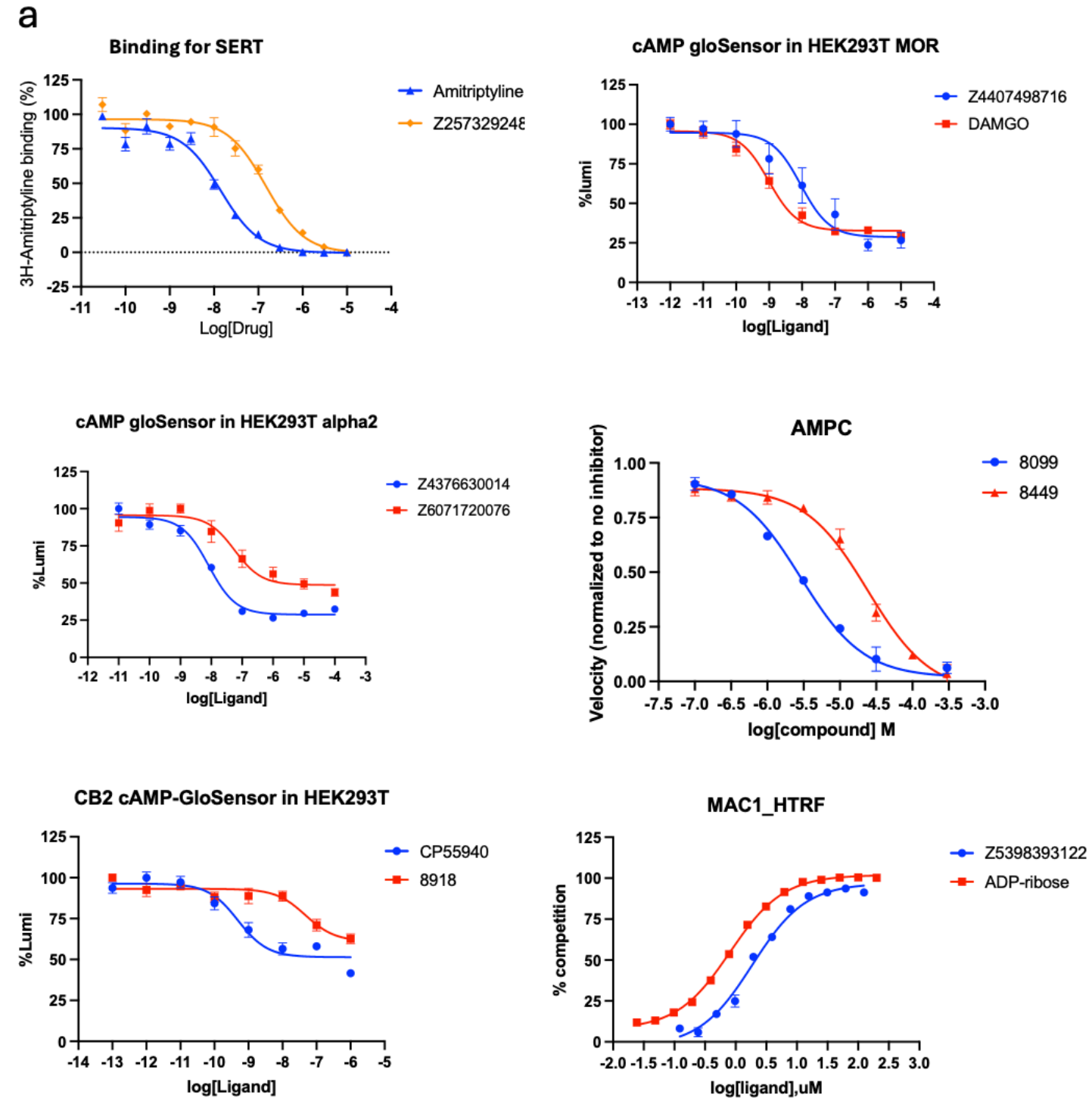

b

Target	Readout	Parent	Z-value
SERT	Ki	Z2573292480, Z2009978218, Z2573292509, Z6971277399, Z8727393896, Z8731642686	0.658
MOR	EC50	Z4407498716	0.5728
alpha2	EC50	Z2750653629, Z4376630014	0.409
alpha2	Ki	Z3034773248, Z8727395870	0.787
AmpC	Ki	Z2610488449	0.493
cb2	Ki	Z52076138, Z6969215903	0.576
cb2	EC50	Z8184698918	0.571
Mac1	IC50	Z5398393122, Z1039063794, Z7534253453	0.880

a. Representative concentration–response curves for the assays used in this study (SERT; MOR, α2, and CB2; AmpC; Mac1).
b. Corresponding Z-values and the parent compounds used for each target/readout.

Supplementary Fig. 4 | Receptor expression levels for α2A, CB2, and MOR (relative and absolute measurements)

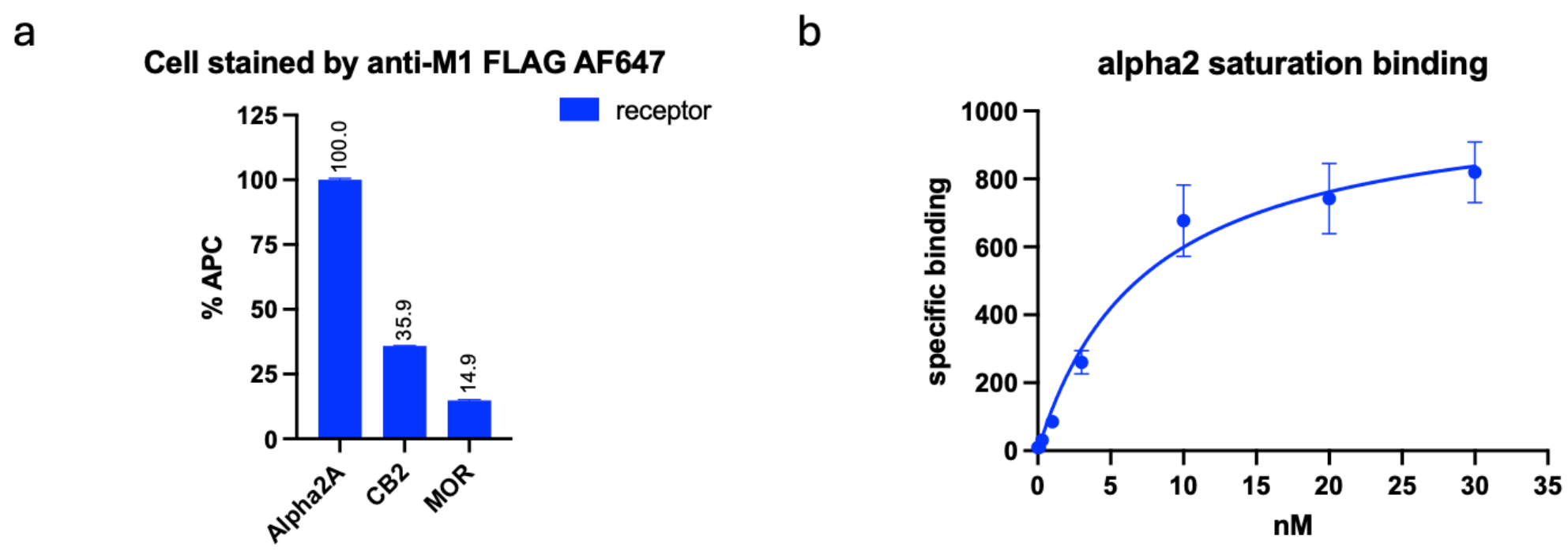

96 plate	Plasmid	Expression
Alpha2A	100ng	100
CB2	300ng	35.9
MOR	600ng	14.9

Best-fit values	
Bmax	1046
Kd	7.466
95% CI (profile likelihood)	
**Bmax**	**870.7 to 1322**
Kd	4.216 to 14.00

a. Relative quantification of surface receptor expression for α2A, CB2, and MOR by flow cytometry using anti-M1 FLAG–AF647 staining (normalized to α2A = 100%).
b. Absolute quantification of α2 receptor expression by radioligand saturation binding, reporting best-fit **Bmax** and **Kd** (with 95% confidence intervals).

Supplementary Fig. 5 | cAMP responses at different receptor expression levels

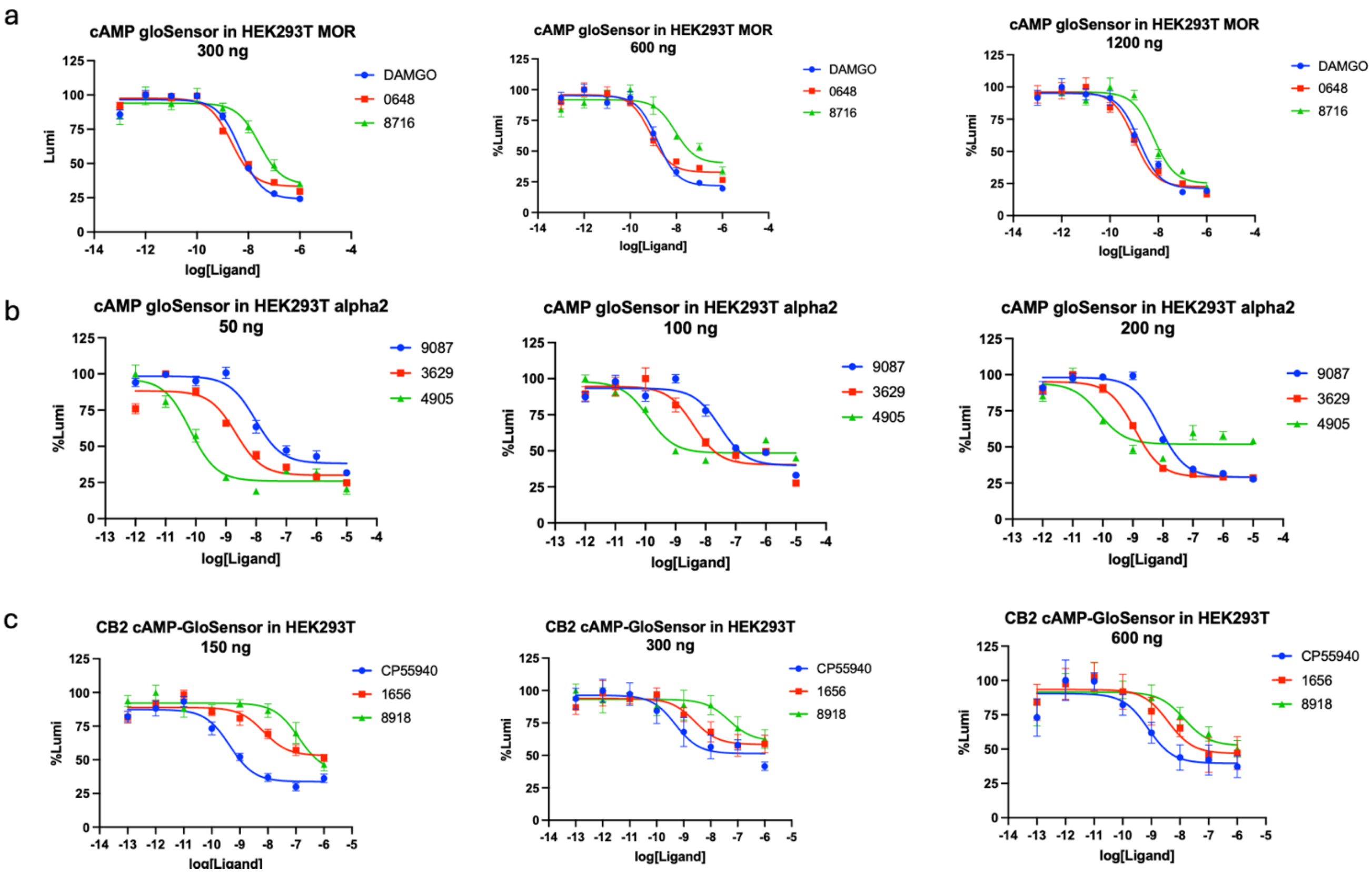

a. MOR cAMP GloSensor concentration–response curves measured in HEK293T cells transfected with increasing amounts of MOR plasmid (300, 600, and 1200 ng), comparing DAMGO and two test ligands.
b. α2AR cAMP GloSensor concentration–response curves measured at increasing receptor plasmid amounts (50, 100, and 200 ng), comparing 9087 and two test ligands.
c. CB2 cAMP GloSensor concentration–response curves measured at increasing receptor plasmid amounts (150, 300, and 600 ng), comparing CP55940 and two test ligands. Points show mean ± s.e.m. with fitted dose–response curves.

Supplementary Fig. 6 | Correlation between parent compound or binding-pocket properties and affinity/potency fold change

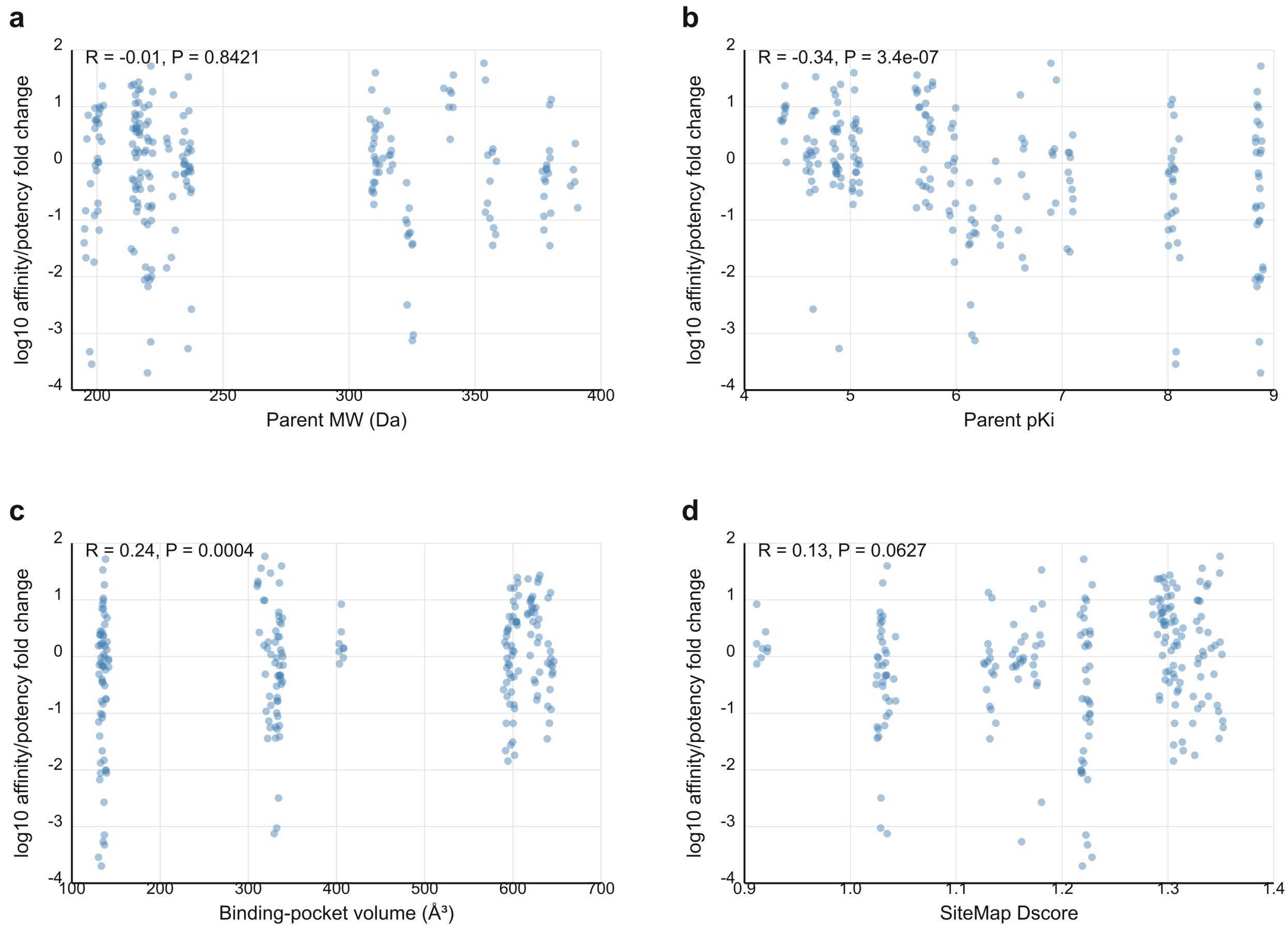

a, Correlation between parent molecular weight (MW) and log10 affinity/potency fold change (R = −0.0135, P = 0.8421). b, Correlation between parent pKi and log10 affinity/potency fold change (R = −0.3356, P = 3.42 × 10−7). c, Correlation between binding-pocket volume and log10 affinity/potency fold change (R = 0.2366, P = 4.01 × 10−4). d, Correlation between SiteMap Dscore and log10 affinity/potency fold change (R = 0.1257, P = 0.06268). Each point represents one analog–parent pair with complete numerical affinity/potency data (n = 220 for each panel). R denotes Pearson’s correlation coefficient; P values are two-sided and unadjusted for multiple comparisons. Horizontal jitter was added for visualization only.

Supplementary Fig. 7 | Pharmacokinetic profiles of 29 individual analogs with at least tenfold affinity/potency improvement

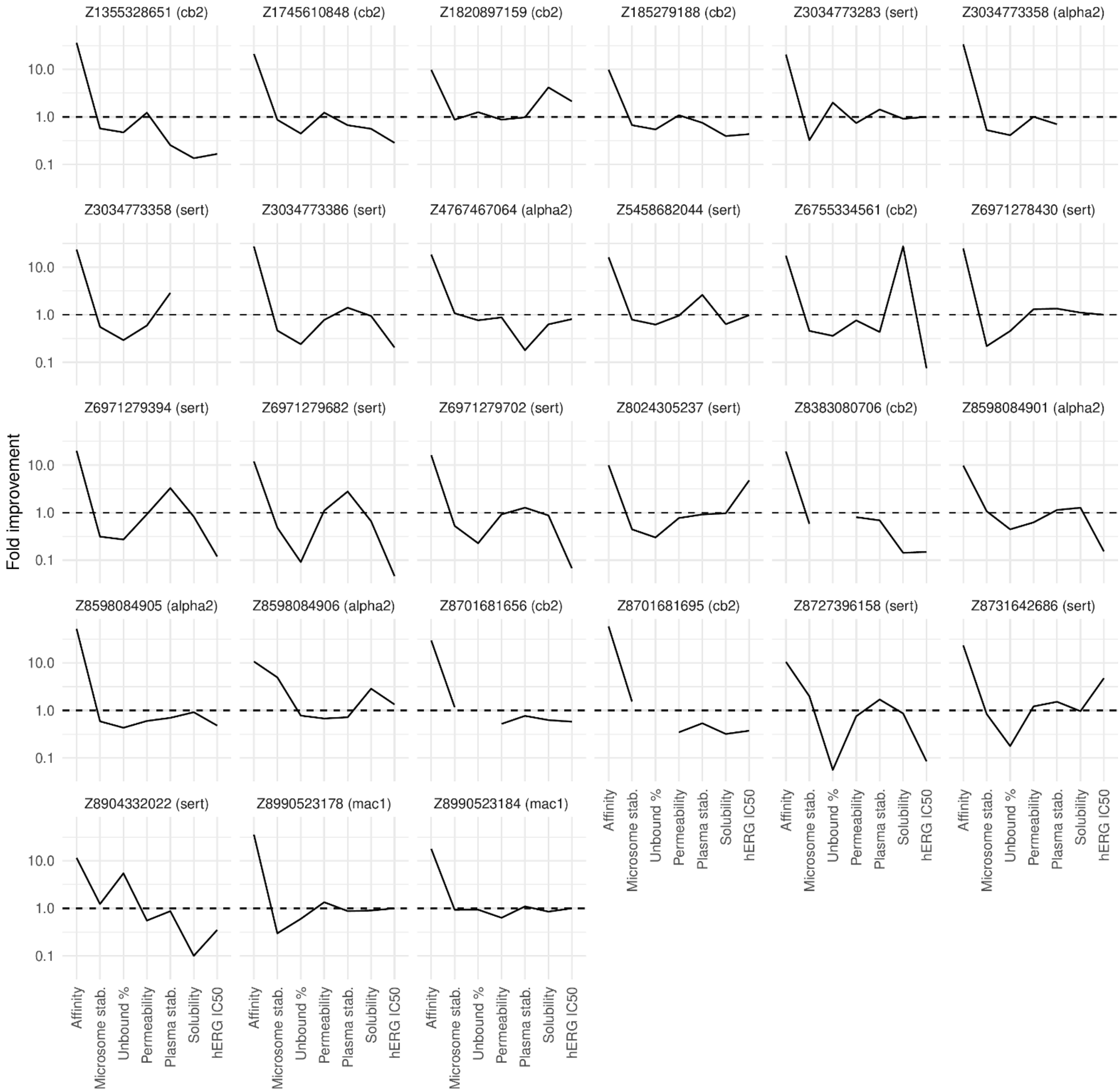

Fold changes in in vitro pharmacokinetic properties, including microsomal stability, plasma fraction unbound, PAMPA permeability, plasma stability, solubility and hERG inhibition, for 29 analogs showing at least tenfold affinity/potency improvement.

Supplementary Fig. 8 | Distribution and cumulative effects of atom modifications on molecular properties

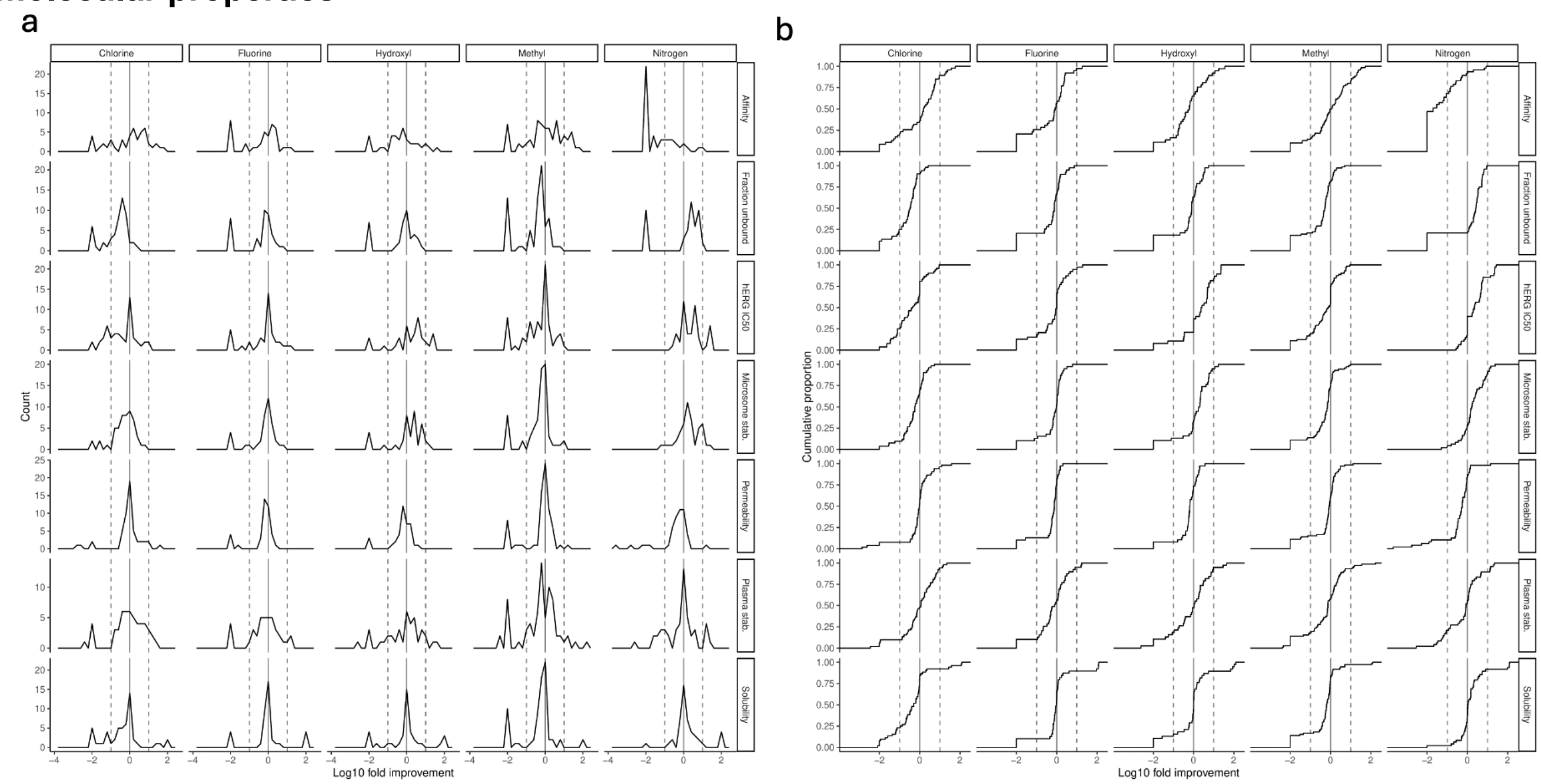

a, Density plots showing the distribution of log10 fold improvement for different properties after single-atom modifications (chlorine, fluorine, hydroxyl, methyl and nitrogen). Each row represents a property and each column represents a modification. Vertical dashed lines indicate no change and approximately threefold improvement or reduction. b, Cumulative distribution plots of the same data, showing the proportion of analogs achieving different levels of improvement or reduction.

Supplementary Fig. 9 | PTX-treated cAMP responses and structural rationale for ligand-specific Gs coupling by 4905

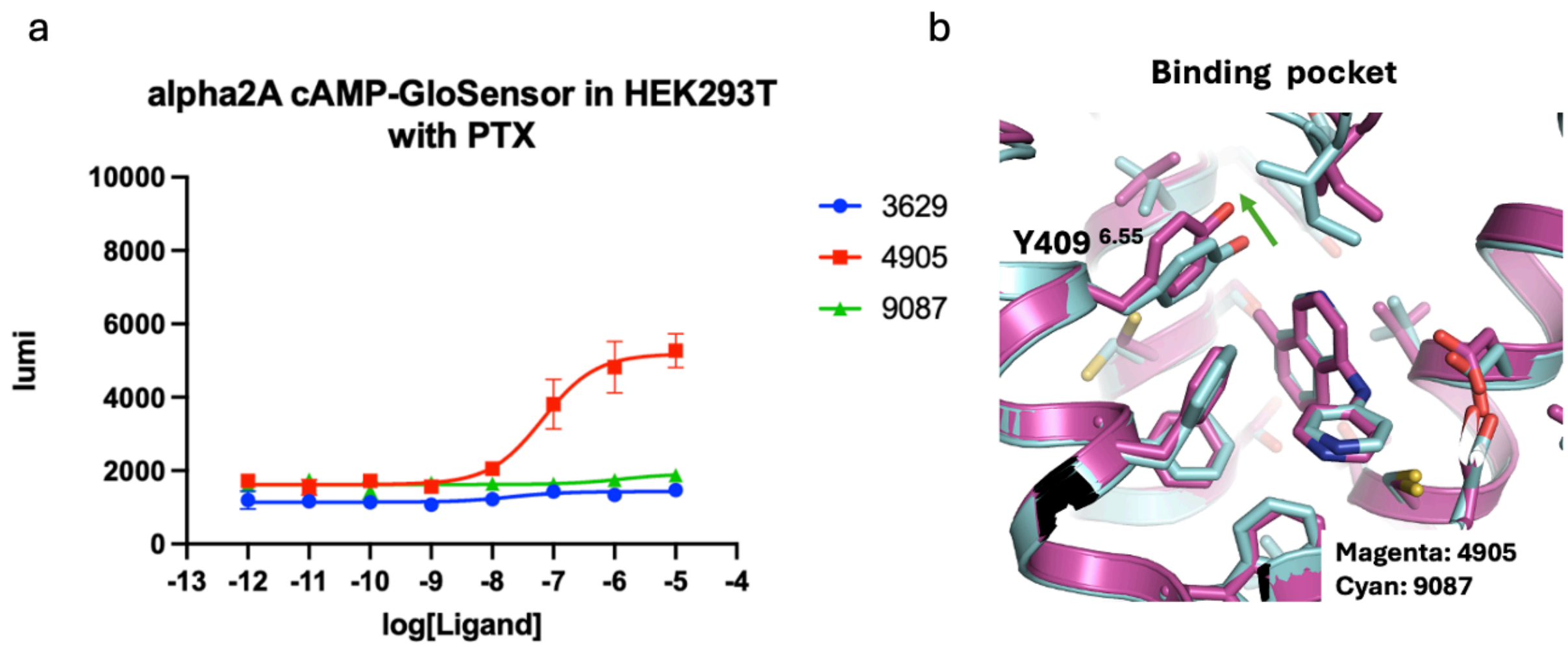

a. Pertussis toxin (PTX)-treated α2A cAMP-GloSensor concentration–response curves in HEK293T cells for 3629, 4905, and 9087. A residual, concentration-dependent increase in cAMP is observed for 4905 at high concentrations, whereas 3629 and 9087 remain flat under PTX treatment.

b. Overlay of the α2A binding pocket comparing 4905 and 9087 complexes (magenta, 4905; cyan, 9087), highlighting an outward shift of Y409^6.55 associated with the additional methyl group in 4905.

Supplementary Methods | Chemical synthesis and characterization

General considerations.

All chemicals for the synthesis of the studied compounds were provided by Enamine Ltd. (www.enamine.net). All solvents were treated according to standard methods. ^{1}H NMR spectra were recorded at 400, 500, or 600 MHz (Varian or Bruker spectrometers). ^{1}H chemical shifts are calibrated using residual non-deuterated solvent DMSO, $CHCl_3$, or methanol: δ = 2.50 ppm, 7.29 ppm, or 3.43 ppm, respectively. Coupling constants are given in Hz. LC/MS analysis was performed utilizing Agilent 1200 Series LC/MSD system with DAD/ELSD (column Zorbax SB-C18 1.8 µm 4.6x15mm; solvent A (water, 0.1% formic acid) and solvent B (acetonitrile, 0.1% formic acid); gradient 0% – 100% solvent B, run time, 1.8 min; flow rate, 3 mL/min) and Agilent LC/MSD SL (G6130A), SL (G6140A) mass-spectrometer (APCI mode). All the LC/MS data were obtained using positive/negative mode switching.

Synthetic procedures.

Method 1. A carboxylic acid (100 mg) was dissolved in 0.5 mL of 10% Hydroxybenzotriazole (HOBt) in DMF, followed by the addition of an amine (1 mol equiv to the carboxylic acid) and 1-ethyl-3-(3-dimethylaminopropyl)carbodiimide (EDC) (1.2 mol equiv to the carboxylic acid). The resulting mixture was shaken for 24 h at room temperature. Then, $CHCl_3$ (2 mL) was added, and the organic phase was washed with water, dried over sodium sulfate, and evaporated under reduced pressure. The crude was dissolved in 0.5 mL of DMSO and further purified by preparative HPLC.

Method 2. An amine (50 mg), DIPEA (1.2 mol equiv to the amine), and DMSO (0.5 mL) were placed into a 4 mL capped glass vial and stirred for 30 min at room temperature. After the addition of an aryl halide (1.2 mol equiv to the amine), the mixture was stirred for 1 h. Then the vial was placed on a thermostat (set to 100°C) for 9 h. After cooling down, the mixture was filtered; the solvent and volatile components were evaporated under reduced pressure. The sample was further purified by preparative HPLC.

Method 3. An aryl halide (100 mg), boronic derivative (1 mol equiv to the aryl halide), sodium carbonate (1.5 mol equiv to the aryl halide), 1,1′- [bis(diphenylphosphino)ferrocene]-dichloropalladium(II) (0.05 mol equiv to the aryl halide), and a water-dioxane mixture 1:3 (1 mL) were placed in a vial. The vial was then placed into a thermostat and stirred for 24 h at 95 °C. After cooling down the mixture, the solvents were evaporated under reduced pressure. The residue was dissolved in chloroform (3 mL) and washed with water (3 × 1 mL). The catalyst was then filtered, and solvents were evaporated under reduced pressure. The sample was dissolved in 0.6 mL of TFA, placed in an ultrasonic bath for 1 h, and then kept overnight at room temperature. TFA was further removed under reduced pressure, and the crude sample was purified by preparative HPLC.

Method 4. An amine (50 mg), DIPEA (1.2 mol equiv to the amine), and bis(2,2,2-trifluoroethyl) carbonate (1.5 mol equiv to the amine) were dissolved in 0.5 mL of acetonitrile in a 4 mL vial. The sealed vial was heated for 2 h at 100°C, then cooled down to room temperature, and the solvent and the unreacted carbonate were removed under reduced pressure. The residual sample was dissolved in 0.5 mL of acetonitrile, and an amino acid *t*-butyl ester (1.1 mol equiv to the aryl amine) and DBU (1.3 mol equiv to the amine) were added. The sealed reaction mixture was kept for 15 h at 100 °C, then cooled down to room temperature, and the solvent was removed under reduced pressure. The sample was dissolved in 0.6 mL of TFA, placed in an ultrasonic bath for 1 h, and then kept overnight at room temperature. TFA was further removed under reduced pressure, and the crude sample was purified by preparative HPLC.

Method 5. A mixture of a nitrile (1 mmol), $NH_2OH·HCl$ (1.5 mmol), and TEA (2 mmol) in ethanol was shaken in a sealed 8 mL vial at room temperature for 6 h. Then, the reaction mixture was heated at 70°C for 16 h. The solvent was removed after the heating, utilizing a rotary evaporator. To the in situ formed amidoxime, a carboxylic acid (1 mmol), EDC (1.5 mmol), and 0.7 mL of 20 wt% % HOAt in DMF were added, and the mixture was shaken at room temperature for 24 h. The cyclodehydration was performed in the presence of TEA (1 mmol) by heating the reaction vial at 100 °C for 3 h. Then, 3 mL of water was added, and the mixture was extracted with CHCl3. The chloroform phase was washed with water three times and evaporated. The sample was dissolved in 0.6 mL of TFA, placed in an ultrasonic bath for 1 h, and then kept overnight at room temperature. TFA was further removed under reduced pressure, and the crude sample was purified by preparative HPLC.

Method 6. An aryl halide (100 mg), boronic derivative (1 mol equiv to the aryl halide), sodium carbonate (1.5 mol equiv to the aryl halide), 1,1′- [bis(diphenylphosphino)ferrocene]-dichloropalladium(II) (0.05 mol equiv to the aryl halide), and a water-dioxane mixture 1:3 (1 mL) were placed in a vial. The vial was then placed into a thermostat and stirred for 24 h at 95 °C. After cooling down the mixture, the solvents were evaporated under reduced pressure. The residue was dissolved in chloroform (3 mL) and washed with water (3 × 1 mL). The catalyst was then filtered, and solvents were evaporated under reduced pressure. The product was further purified by HPLC.

Method 7. An amine (50 mg), DIPEA (1.2 mol equiv to the amine), and bis(2,2,2-trifluoroethyl) carbonate (1.5 mol equiv to the amine) were dissolved in 0.5 mL of acetonitrile in a 4 mL vial. The sealed vial was heated for 2 h at 100°C, then cooled down to room temperature, and the solvent and the unreacted carbonate were removed under reduced pressure. The residual sample was dissolved in 0.5 mL of acetonitrile and an amino acid *t*-butyl ester (1.1 mol equiv to the aryl amine) and DBU (1.3 mol equiv to the amine) were added. The sealed reaction mixture was kept for 15 h at 100°C, then cooled down to room temperature, and the solvent was removed under reduced pressure an,d the crude sample was purified by preparative HPLC.

Method 8. A carboxylic acid (100 mg) was dissolved in 0.5 mL of 10% Hydroxybenzotriazole (HOBt) in DMF, followed by the addition of an amine (1 mol equiv to the carboxylic acid) and 1-ethyl-3-(3-dimethylaminopropyl)carbodiimide (EDC) (1.2 mol equiv to the carboxylic acid). The resulting mixture was shaken for 24 h at room temperature. Then, $CHCl_3$ (2 mL) was added, and the organic phase was washed with water, dried over sodium sulfate, and evaporated under reduced pressure. The sample was dissolved in 0.5 mL of DMSO and subjected to hydrolysis with 0.2 mL of 4M KOH for 12 h at room temperature. Then, 50 uL of formic acid was added, and the reaction vial was shaken for 30 min at room temperature. The solvent was evaporated under reduced pressure, and the crude product was dissolved in 0.5 mL of DMSO. The sample was further purified by preparative HPLC.

Method 9. The molecules were synthesized according to the previously published procedure (https://doi.org/10.1007/s11030-021-10218-2).

Compound characterization data

3-(2-(7-chloro-3,4-dihydrobenzo[b][1,4]thiazepin-5(2H)-yl)-2-oxoethyl)isobenzofuran-1(3H)-one - Z1355328651 (Method 1).

Yield: 43%; purity, >95% (assessed by LC/MS).

^{1}H NMR (500 MHz, $CDCl_3$) δ 8.59 (d, J = 15.4 Hz, 1H), 8.01 (d, J = 7.6 Hz, 1H), 7.60 (d, J = 8.3 Hz, 1H), 7.40 (dt, J = 25.3, 7.5 Hz, 2H), 7.31 (s, 1H), 7.30 - 7.22 (m, 3H), 6.03 (d, J = 15.4 Hz, 1H), 4.96 (d, J = 13.8 Hz, 1H), 2.94 (d, J = 14.2 Hz, 1H), 2.76 (dt, J = 27.0, 12.9 Hz, 2H), 2.41 (q, J = 13.1 Hz, 1H), 2.14 (s, 1H).

LC/MS (APSI) m/z [M+H] calculated for C19H17ClNO3S: 374.1; found: 374.0.

N-(1-(2,3-dihydrobenzo[b][1,4]dioxin-6-yl)-2-methylpropyl)-2-methyl-7H-pyrrolo[2,3-d]pyrimidin-4-amine - Z1359487111 (Method 2).

Yield: 19%; purity, >95% (assessed by LC/MS).

^{1}H NMR (600 MHz, DMSO-*d*$_6$) δ 11.17 (s, 1H), 7.35 (d, J = 9.3 Hz, 1H), 6.94 (dt, J = 3.5, 2.0 Hz, 2H), 6.85 (dd, J = 8.3, 2.0 Hz, 1H), 6.74 (d, J = 8.3 Hz, 1H), 6.59 (s, 1H), 4.94 (s, 1H), 4.22 - 4.14 (m, 3H), 3.29 (s, 2H), 2.32 (s, 2H), 2.10 - 2.05 (m, 1H), 1.00 (d, J = 6.6 Hz, 3H), 0.75 (d, J = 6.6 Hz, 3H).

LC/MS (APSI) m/z [M+H] calculated for C19H23N4O2: 339.2; found: 339.2.

3-(2-(3-methyl-3,4-dihydrobenzo[b][1,4]thiazepin-5(2H)-yl)-2-oxoethyl)isobenzofuran-1(3H)-one - Z1511587581 (Method 1).

Yield: 26%; purity, >95% (assessed by LC/MS).

^{1}H NMR (500 MHz, DMSO-*d*$_6$) δ 7.78 - 7.67 (m, 2H), 7.66 - 7.50 (m, 3H), 7.44 - 7.30 (m, 2H), 7.27 (td, J = 7.3, 1.9 Hz, 1H), 5.92 - 5.83 (m, 1H), 4.41 - 4.33 (m, 1H), 2.96 - 2.81 (m, 2H), 2.43 - 2.33 (m, 2H), 2.21 (s, 1H), 2.06 (dd, J = 16.8, 7.4 Hz, 1H), 0.89 (dd, J = 9.3, 6.6 Hz, 3H).

LC/MS (APSI) m/z [M+H] calculated for C20H20NO3S: 354.1; found: 354.1.

3-(2-(8-methyl-3,4-dihydrobenzo[b][1,4]thiazepin-5(2H)-yl)-2-oxoethyl)isobenzofuran-1(3H)-one - Z1745610848 (Method 1).

Yield: 30%; purity, >95% (assessed by LC/MS).

^{1}H NMR (600 MHz, DMSO-*d*$_6$) δ 7.80 - 7.69 (m, 2H), 7.65 (t, J = 8.0 Hz, 1H), 7.56 (t, J = 7.5 Hz, 1H), 7.41 (dd, J = 13.1, 2.0 Hz, 1H), 7.28 (d, J = 7.9 Hz, 0H), 7.14 (ddd, J = 10.4, 7.8, 2.0 Hz, 1H), 5.95 - 5.87 (m, 1H), 4.55 (ddd, J = 13.5, 8.9, 3.9 Hz, 1H), 3.30 (s, 3H), 2.95 - 2.88 (m, 1H), 2.67 (ddd, J = 20.0, 10.3, 4.9 Hz, 2H), 2.31 - 2.23 (m, 3H), 2.15 - 2.04 (m, 1H), 2.04 - 1.95 (m, 1H).

LC/MS (APSI) m/z [M+H] calculated for C20H20NO3S: 354.1; found: 354.0.

2-chloro-N-(naphthalen-1-yl)pyridin-4-amine - Z1800559918 (Method 2).

Yield: 34%; purity, >95% (assessed by LC/MS).

^{1}H NMR (500 MHz, DMSO-*d*$_6$) δ 9.19 (s, 1H), 7.98 (dt, J = 7.2, 2.5 Hz, 1H), 7.97 - 7.90 (m, 2H), 7.83 (d, J = 8.1 Hz, 1H), 7.59 - 7.50 (m, 3H), 7.47 (d, J = 7.3 Hz, 1H), 6.69 (dt, J = 5.7, 2.5 Hz, 1H), 6.65 - 6.60 (m, 1H).

LC/MS (APSI) m/z [M+H] calculated for C15H12ClN2: 255.1; found: 255.0.

3-(2-(7-fluoro-3,4-dihydrobenzo[b][1,4]thiazepin-5(2H)-yl)-2-oxoethyl)isobenzofuran-1(3H)-one - Z1820897159 (Method 1).

Yield: 46%; purity, 95% (assessed by LC/MS).

^{1}H NMR (500 MHz, DMSO-*d*$_6$) δ 7.90 - 7.56 (m, 3H), 7.54 (t, J = 7.4 Hz, 1H), 7.38 (ddd, J = 16.0, 9.3, 2.8 Hz, 1H), 7.19 - 7.06 (m, 1H), 6.72 - 6.31 (m, 1H), 5.95 - 5.83 (m, 1H), 4.51 (dd, J = 14.1, 4.4 Hz, 1H), 2.94 - 2.79 (m, 2H), 2.75 - 2.60 (m, 2H), 2.33 - 1.93 (m, 2H), 1.95 - 1.86 (m, 1H).

LC/MS (APSI) m/z [M+H] calculated for C19H17FNO3S: 358.1; found: 358.2.

3-(2-(2-methyl-3,4-dihydrobenzo[b][1,4]thiazepin-5(2H)-yl)-2-oxoethyl)isobenzofuran-1(3H)-one - Z185279188 (Method 1).

Yield: 54%; purity, >95% (assessed by LC/MS).

^{1}H NMR (500 MHz, DMSO-*d*$_6$) δ 7.75 (dd, J = 7.8, 5.0 Hz, 1H), 7.75 - 7.67 (m, 1H), 7.67 - 7.47 (m, 3H), 7.41 - 7.32 (m, 2H), 7.27 (dh, J = 8.0, 2.3 Hz, 1H), 5.94 - 5.85 (m, 1H), 4.57 - 4.50 (m, 1H), 2.93 (ddd, J = 21.4, 12.7, 4.8 Hz, 1H), 2.77 - 2.59 (m, 1H), 2.35 - 2.23 (m, 1H), 2.15 - 1.94 (m, 1H), 1.96 (s, 1H), 1.25 (d, J = 6.8 Hz, 2H), 1.09 (t, J = 6.3 Hz, 1H).

LC/MS (APSI) m/z [M+H] calculated for C20H20NO3S: 354.1; found: 354.0.

5-(2,3-dihydro-1H-inden-5-yl)-1,2,3,6-tetrahydropyridine - Z2009978218 (Method 3).
Yield: 20%; purity, >95% (assessed by LC/MS).

^{1}H NMR (500 MHz, DMSO-d_6) δ 7.18 (s, 1H), 7.13 (d, J = 7.9 Hz, 1H), 7.08 (d, J = 7.9 Hz, 1H), 6.09 (d, J = 4.4 Hz, 1H), 2.80 (tt, J = 10.8, 5.6 Hz, 8H), 2.10 (h, J = 3.0 Hz, 2H), 2.07 - 1.92 (m, 3H).

LC/MS (APSI) m/z [M+H] calculated for C14H18N: 200.1; found: 200.2.

N-(1-(2,3-dihydrobenzo[b][1,4]dioxin-6-yl)-2-methylpropyl)-1H-pyrazolo[3,4-d]pyrimidin-4-amine - Z237580338 (Method 2).
Yield: 26%; purity, >95% (assessed by LC/MS).

^{1}H NMR (600 MHz, DMSO-d_6) δ 13.34 (s, 1H), 8.28 (d, J = 9.0 Hz, 1H), 8.22 (s, 1H), 8.15 (s, 1H), 6.92 (d, J = 2.1 Hz, 1H), 6.84 (dd, J = 8.3, 2.1 Hz, 1H), 6.77 (d, J = 8.3 Hz, 1H), 5.01 (t, J = 9.0 Hz, 1H), 4.22 - 4.17 (m, 4H), 2.07 (q, J = 7.2 Hz, 1H), 1.00 (d, J = 6.6 Hz, 3H), 0.77 (d, J = 6.6 Hz, 3H).

LC/MS (APSI) m/z [M+H] calculated for C17H20N5O2: 326.2; found: 326.2.

5-(benzo[b]thiophen-5-yl)-1,2,3,6-tetrahydropyridine - Z2573292480 (Method 3).
Yield: 18%; purity, >95% (assessed by LC/MS).

^{1}H NMR (600 MHz, DMSO-d_6) δ 7.92 (d, J = 8.4 Hz, 1H), 7.83 (s, 1H), 7.73 (d, J = 5.4 Hz, 1H), 7.43 (d, J = 5.6 Hz, 2H), 6.28 (s, 1H), 3.62 (s, 2H), 2.83 (t, J = 5.7 Hz, 2H), 2.18 (d, J = 6.5 Hz, 2H).

LC/MS (APSI) m/z [M+H] calculated for C13H14NS: 216.1; found: 216.1.

5-(benzo[b]thiophen-5-yl)-4-methyl-1,2,3,6-tetrahydropyridine hydrochloride - Z2573292509 (Method 3).
Yield: 18%; purity, >95% (assessed by LC/MS).

^{1}H NMR (600 MHz, DMSO-d_6) δ 8.47 - 8.42 (m, 1H), 8.15 - 8.08 (m, 1H), 7.98 - 7.06 (m, 6H), 3.40 (s, 1H), 2.90 (t, J = 5.8 Hz, 1H), 2.29 (s, 2H), 2.06 (s, 1H), 1.58 (s, 3H).

LC/MS (APSI) m/z [M+H] calculated for C14H16NS: 230.1; found: 230.2.

1-((3-(5-phenylisoxazol-3-yl)ureido)methyl)cyclobutane-1-carboxylic acid - Z2610488449 (Method 4).
Yield: 32%; purity, >95% (assessed by LC/MS).

^{1}H NMR (500 MHz, DMSO-d_6) δ 12.47 (s, 1H), 9.51 (s, 1H), 7.85 - 7.79 (m, 2H), 7.55 - 7.44 (m, 3H), 7.11 (s, 1H), 6.62 (t, J = 6.0 Hz, 1H), 3.50 (d, J = 5.9 Hz, 2H), 2.31 - 2.21 (m, J = 4.1 Hz, 2H), 1.89 (dtd, J = 25.0, 17.7, 17.3, 7.8 Hz, 4H).

LC/MS (APSI) m/z [M+H] calculated for C16H18N3O4: 316.1; found: 316.0.

1-((3-(5-(3-chlorophenyl)22yclobute-3-yl)ureido)methyl)22yclobutene-1-carboxylic acid - Z2610512538 (Method 4).
Yield: 31%; purity, >95% (assessed by LC/MS).

^{1}H NMR (500 MHz, DMSO-d_6) δ 12.47 (s, 1H), 9.54 (s, 1H), 7.91 (d, J = 2.2 Hz, 1H), 7.80 (dt, J = 6.5, 2.0 Hz, 1H), 7.57 – 7.49 (m, 2H), 7.26 (s, 1H), 6.61 (t, J = 5.9 Hz, 1H), 3.50 (d, J = 5.8 Hz, 2H), 2.26 (dp, J = 9.4, 5.1, 4.7 Hz, 2H), 1.98 – 1.79 (m, 4H), 1.85 (s, 1H).

LC/MS (APSI) m/z [M+H] calculated for C16H17ClN3O4: 350.1; found: 350.1.

1-[({[5-(2-fluorophenyl)-1,2-oxazol-3-yl]carbamoyl}amino)methyl]22yclobutene-1-carboxylic acid - Z2610513554 (Method 4).
Yield: 38%; purity, >95% (assessed by LC/MS).

^{1}H NMR (600 MHz, DMSO-d_6) δ 12.48 (s, 1H), 9.59 (s, 1H), 7.90 (td, J = 7.7, 1.7 Hz, 1H), 7.58 (tdd, J = 7.5, 5.2, 1.7 Hz, 1H), 7.44 (dd, J = 11.3, 8.3 Hz, 1H), 7.39 (t, J = 7.6 Hz, 1H), 7.08 (d, J = 3.6 Hz, 1H), 6.64 (t, J = 6.0 Hz, 1H), 3.51 (d, J = 5.9 Hz, 2H), 2.27 (qd, J = 7.1, 3.5 Hz, 2H), 1.99 – 1.82 (m, 4H).

LC/MS (APSI) m/z [M+H] calculated for C16H17FN3O4: 334.0; found: 334.1.

1-[({[5-(3-fluorophenyl)-1,2-oxazol-3-yl]carbamoyl}amino)methyl]cyclobutane-1-carboxylic acid - Z2610513975 (Method 4).
Yield: 29%; purity, >95% (assessed by LC/MS).

^{1}H NMR (500 MHz, Methanol-d_4) δ 7.62 (d, J = 7.8 Hz, 1H), 7.52 (ddt, J = 11.5, 8.0, 4.3 Hz, 2H), 7.22 (td, J = 8.5, 2.5 Hz, 1H), 6.95 (s, 1H), 3.65 (s, 2H), 2.47 - 2.38 (m, 2H), 2.02 (ttt, J = 19.1, 9.8, 4.3 Hz, 3H).

LC/MS (APSI) m/z [M+H] calculated for C16H17FN3O4: 334.0; found: 334.1.

N-(pyridin-4-yl)quinolin-8-amine - Z2750652484 (Method 2).
Yield: 47%; purity, >95% (assessed by LC/MS).
^{1}H NMR (500 MHz, DMSO-d_6) δ 9.15 (s, 1H), 8.90 (dt, J = 4.2, 2.0 Hz, 1H), 8.37 (dt, J = 8.3, 2.1 Hz, 1H), 8.30 - 8.23 (m, 2H), 7.73 (dd, J = 6.6, 2.3 Hz, 1H), 7.61 (dd, J = 8.3, 4.1 Hz, 1H), 7.58 - 7.51 (m, 2H), 7.32 - 7.27 (m, 2H).
LC/MS (APSI) m/z [M+H] calculated for C14H12N3: 222.1; found: 222.1.

N-(naphthalen-1-yl)pyridin-4-amine - Z2750653629 (Method 2).
Yield: 18%; purity, >95% (assessed by LC/MS).
^{1}H NMR (500 MHz, DMSO-d_6) δ 8.83 (s, 1H), 8.14 - 8.05 (m, 2H), 8.01 - 7.93 (m, 2H), 7.76 (d, J = 8.1 Hz, 1H), 7.60 - 7.47 (m, 3H), 7.45 (d, J = 7.3 Hz, 1H), 6.75 - 6.69 (m, 2H).
LC/MS (APSI) m/z [M+H] calculated for C15H13N2: 221.2; found: 221.1.

5-{5H,6H,7H-cyclopenta[b]pyridin-2-yl}-1,2,3,6-tetrahydropyridine - Z3033185506 (Method 3).
Yield: 24%; purity, >95% (assessed by LC/MS).
^{1}H NMR (600 MHz, DMSO-d_6) δ 7.53 (d, J = 7.9 Hz, 1H), 7.19 (d, J = 7.9 Hz, 1H), 6.63 (d, J = 4.3 Hz, 1H), 3.61 (d, J = 3.1 Hz, 2H), 2.86 (t, J = 7.5 Hz, 5H), 2.80 (t, J = 5.7 Hz, 2H), 2.16 (d, J = 5.7 Hz, 2H), 2.03 (p, J = 7.6 Hz, 2H).
LC/MS (APSI) m/z [M+H] calculated for C13H17N2: 201.2; found: 201.1.

3-(2-Azetidinyl)-5-phenyl-1,2,4-oxadiazole - Z8727393896, Z3034773110 (Method 5).
Yield: 27%; purity, >95% (assessed by LC/MS).
^{1}H NMR (500 MHz, DMSO-d_6) δ 9.64 (s, 2H), 8.18 - 8.13 (m, 2H), 7.76 (t, J = 7.4 Hz, 1H), 7.67 (t, J = 7.6 Hz, 2H), 5.77 (t, J = 8.5 Hz, 1H), 4.14 (q, J = 9.2 Hz, 1H), 3.99 (td, J = 9.9, 6.0 Hz, 1H), 2.95 - 2.79 (m, 2H).
LC/MS (APSI) m/z [M+H] calculated for C11H12N3O1: 202.1; found: 202.0.

3-[3-(azetidin-2-yl)-1,2,4-oxadiazol-5-yl]pyridine - Z3034773111 (Method 5).
Yield: 15%; purity, >95% (assessed by LC/MS).
LC/MS (APSI) m/z [M+H] calculated for C10H11N4O: 203.0; found: 203.1.

4-[3-(azetidin-2-yl)-1,2,4-oxadiazol-5-yl]pyridine - Z3034773112 (Method 5).
Yield: 14%; purity, >95% (assessed by LC/MS).
^{1}H NMR (500 MHz, DMSO-d_6) δ 8.89 - 8.82 (m, 2H), 8.05 - 7.98 (m, 2H), 4.99 (t, J = 7.7 Hz, 1H), 3.61 (q, J = 7.8 Hz, 1H), 3.37 (q, J = 6.7 Hz, 1H), 2.58 (td, J = 8.5, 7.8, 6.4 Hz, 2H).
LC/MS (APSI) m/z [M+H] calculated for C10H11N4O: 203.0; found: 203.1.

3-(2-Azetidinyl)-5-(o-tolyl)-1,2,4-oxadiazole - Z8731642686, Z3034773188 (Method 5).
Yield: 17%; purity, >95% (assessed by LC/MS).
^{1}H NMR (500 MHz, DMSO-d_6) δ 9.60 (s, 2H), 8.06 (d, J = 7.7 Hz, 1H), 7.61 (t, J = 7.5 Hz, 1H), 7.50 (d, J = 7.7 Hz, 1H), 7.46 (t, J = 7.6 Hz, 1H), 5.76 (t, J = 8.4 Hz, 1H), 4.13 (q, J = 9.2 Hz, 1H), 4.00 (td, J = 9.7, 6.2 Hz, 1H), 2.88 (qt, J = 8.8, 4.7 Hz, 2H), 2.66 (s, 3H).
LC/MS (APSI) m/z [M+H] calculated for C12H14N3O1: 216.1; found: 216.0.

3-(2-Azetidinyl)-5-(3-methyl-2-pyridyl)-1,2,4-oxadiazole - Z8890819186, Z3034773192 (Method 5).
Yield: 15%; purity, >95% (assessed by LC/MS).
^{1}H NMR (600 MHz, DMSO-d_6) δ 9.56 (s, 2H), 8.68 (td, J = 7.0, 5.8, 2.6 Hz, 1H), 8.00 - 7.91 (m, 1H), 7.66 (dd, J = 7.8, 4.6 Hz, 1H), 5.82 (t, J = 8.4 Hz, 1H), 4.16 (q, J = 9.2 Hz, 1H), 4.03 (td, J = 9.9, 6.2 Hz, 1H), 2.96 - 2.85 (m, 2H), 2.69 (d, J = 10.9 Hz, 3H).
LC/MS (APSI) m/z [M+H] calculated for C11H13N4O1: 217.1; found: 217.0.

3-(2-Azetidinyl)-5-(2-methyl-3-pyridyl)-1,2,4-oxadiazole - Z8735711383, Z3034773194 (Method 5).
Yield: 24%; purity, >95% (assessed by LC/MS).
^{1}H NMR (500 MHz, DMSO-d_6) δ 9.77 (s, 2H), 8.74 (dd, J = 4.7, 1.8 Hz, 1H), 8.43 (dd, J = 8.1, 1.8 Hz, 1H), 7.52 (dd, J = 7.9, 4.8 Hz, 1H), 5.80 (t, J = 8.4 Hz, 1H), 4.15 (q, J = 9.2 Hz, 1H), 4.02 (td, J = 9.7, 6.3 Hz, 1H), 2.86 (s, 3H), 2.96 - 2.72 (m, 2H).
LC/MS (APSI) m/z [M+H] calculated for C11H13N4O1: 217.1; found: 217.0.

3-(2-Azetidinyl)-5-(o-fluorophenyl)-1,2,4-oxadiazole - Z8735711427, Z3034773205 (Method 5).
Yield: 52%; purity, 94% (assessed by LC/MS).
^{1}H NMR (500 MHz, DMSO-d_6) δ 9.59 (s, 2H), 8.17 (td, J = 7.6, 1.9 Hz, 1H), 7.82 (dtt, J = 7.6, 5.2, 2.0 Hz, 1H), 7.56 (dd, J = 11.1, 8.4 Hz, 1H), 7.50 (t, J = 7.6 Hz, 1H), 5.79 (t, J = 8.5 Hz, 1H), 4.14 (q, J = 9.2 Hz, 1H), 3.99 (td, J = 9.9, 5.9 Hz, 1H), 2.96 - 2.79 (m, 2H).
LC/MS (APSI) m/z [M+H] calculated for C11F1H11N3O1: 220.1; found: 220.1.

3-(2-Azetidinyl)-5-(p-fluorophenyl)-1,2,4-oxadiazole - Z8727395866, Z3034773206 (Method 5).
Yield: 20%; purity, >95% (assessed by LC/MS).
^{1}H NMR (500 MHz, DMSO-d_6) δ 10.16 - 8.44 (m, 1H), 8.40 - 7.91 (m, 2H), 7.71 - 7.25 (m, 2H), 5.77 (t, J = 8.5 Hz, 1H), 4.31 - 3.88 (m, 1H), 3.74 - 3.43 (m, 2H), 3.06 - 2.72 (m, 1H), 2.24 - 1.84 (m, 1H).
LC/MS (APSI) m/z [M+H] calculated for C11F1H11N3O1: 220.1; found: 220.0.

3-(2-Azetidinyl)-5-(o-chlorophenyl)-1,2,4-oxadiazole - Z8727395870, Z3034773247 (Method 5).
Yield: 20%; purity, >95% (assessed by LC/MS).
^{1}H NMR (500 MHz, DMSO-d_6) δ 9.60 (s, 2H), 8.13 (dd, J = 7.8, 1.6 Hz, 1H), 7.76 (dtd, J = 15.3, 8.1, 1.6 Hz, 2H), 7.63 (td, J = 7.5, 1.5 Hz, 1H), 5.80 (t, J = 8.5 Hz, 1H), 4.14 (q, J = 9.1 Hz, 1H), 3.99 (td, J = 9.8, 5.9 Hz, 1H), 2.96 - 2.80 (m, 2H).
LC/MS (APSI) m/z [M+H] calculated for C11Cl1H11N3O1: 236.1; found: 236.0.

3-(azetidin-2-yl)-5-(3-chlorophenyl)-1,2,4-oxadiazole - Z3034773248 (Method 5).
Yield: 34%; purity, >95% (assessed by LC/MS).
^{1}H NMR (500 MHz, DMSO-d_6) δ 9.54 (s, 2H), 8.18 - 8.06 (m, 2H), 7.87 - 7.82 (m, 1H), 7.71 (td, J = 7.9, 2.3 Hz, 1H), 5.77 (t, J = 8.5 Hz, 1H), 4.13 (q, J = 9.3 Hz, 1H), 4.00 (td, J = 9.7, 6.3 Hz, 1H), 2.94 - 2.79 (m, 2H).
LC/MS (APSI) m/z [M+H] calculated for C11H11ClN3O: 236.0; found: 236.1.

3-(2-Azetidinyl)-5-(p-chlorophenyl)-1,2,4-oxadiazole - Z8735711431, Z3034773249 (Method 5).
Yield: 27%; purity, >95% (assessed by LC/MS).
^{1}H NMR (600 MHz, DMSO-d_6) δ 9.52 (s, 2H), 8.21 - 8.13 (m, 2H), 7.79 - 7.74 (m, 2H), 5.78 (t, J = 8.5 Hz, 1H), 4.15 (q, J = 9.0 Hz, 1H), 4.00 (td, J = 9.8, 6.0 Hz, 1H), 2.94 - 2.81 (m, 2H).
LC/MS (APSI) m/z [M+H] calculated for C11Cl1H11N3O1: 236.1; found: 236.0.

3-(2-Azetidinyl)-5-(3-chloro-4-pyridyl)-1,2,4-oxadiazole - Z8727393894, Z3034773251 (Method 5).
Yield: 39%; purity, >95% (assessed by LC/MS).
^{1}H NMR (500 MHz, DMSO-d_6) δ 9.66 (s, 2H), 9.01 (s, 1H), 8.83 (d, J = 5.0 Hz, 1H), 8.12 (d, J = 5.0 Hz, 1H), 5.84 (t, J = 8.5 Hz, 1H), 4.15 (q, J = 9.2 Hz, 1H), 4.00 (td, J = 9.8, 6.1 Hz, 1H), 2.88 (dddt, J = 12.0, 9.1, 5.9, 3.2 Hz, 2H).
LC/MS (APSI) m/z [M+H] calculated for C10Cl1H10N4O1: 237.1; found: 237.0.

3-(2-Azetidinyl)-5-(p-bromophenyl)-1,2,4-oxadiazole - Z8727396158, Z3034773263 (Method 5).
Yield: 24%; purity, >95% (assessed by LC/MS).
^{1}H NMR (500 MHz, DMSO-d_6) δ 9.49 (s, 2H), 8.11 - 8.06 (m, 2H), 7.92 - 7.82 (m, 2H), 5.76 (t, J = 8.5 Hz, 1H), 4.13 (q, J = 9.2 Hz, 1H), 3.98 (td, J = 9.8, 6.1 Hz, 1H), 3.01 - 2.74 (m, 2H).
LC/MS (APSI) m/z [M+H] calculated for C11Br1H11N3O1: 282.0; found: 282.0.

3-(azetidin-2-yl)-5-(2-bromophenyl)-1,2,4-oxadiazole - Z8024305237, Z3034773264 (Method 8).
Yield: 38%; purity, >95% (assessed by LC/MS).
^{1}H NMR (500 MHz, DMSO-d_6) δ 9.53 (s, 2H), 8.06 (dd, J = 6.9, 2.6 Hz, 1H), 7.97 - 7.90 (m, 1H), 7.65 (ddd, J = 7.4, 5.0, 2.1 Hz, 2H), 5.80 (t, J = 8.6 Hz, 1H), 4.13 (q, J = 9.3 Hz, 1H), 3.99 (td, J = 9.8, 6.1 Hz, 1H), 2.94 - 2.80 (m, 2H).
LC/MS (APSI) m/z [M+H] calculated for C11H11BrN3O: 282.0; found: 282.0.

3-(2-Azetidinyl)-5-(m-bromophenyl)-1,2,4-oxadiazole - Z8727394030, Z3034773265 (Method 5).
Yield: 27%; purity, >95% (assessed by LC/MS).
^{1}H NMR (600 MHz, DMSO-d_6) δ 9.49 (s, 2H), 8.31 (t, J = 1.8 Hz, 1H), 8.20 - 8.12 (m, 1H), 7.99 (dd, J = 8.0, 2.1 Hz, 1H), 7.66 (t, J = 7.9 Hz, 1H), 5.79 (t, J = 8.5 Hz, 1H), 4.15 (q, J = 9.1 Hz, 1H), 4.02 (td, J = 9.8, 6.1 Hz, 1H), 2.94 - 2.82 (m, 2H).

LC/MS (APSI) m/z [M+H] calculated for C11Br1H11N3O1: 280.0; found: 280.0.

3-(azetidin-2-yl)-5-(2,6-dimethylphenyl)-1,2,4-oxadiazole - Z3034773283 (Method 5).
Yield: 48%; purity, >95% (assessed by LC/MS).

^{1}H NMR (500 MHz, DMSO-d_6) δ 9.69 (s, 2H), 7.45 (t, J = 7.7 Hz, 1H), 7.27 (d, J = 7.6 Hz, 2H), 5.80 (t, J = 8.5 Hz, 1H), 4.14 (q, J = 9.2 Hz, 1H), 4.00 (td, J = 9.9, 5.9 Hz, 1H), 3.00 - 2.79 (m, 2H), 2.24 (s, 6H).

LC/MS (APSI) m/z [M+H] calculated for C13H16N3O: 230.2; found: 230.1.

3-(2-Azetidinyl)-5-(2,4-xylyl)-1,2,4-oxadiazole - Z8891248505, Z3034773287 (Method 5).
Yield: 42%; purity, >95% (assessed by LC/MS).

^{1}H NMR (500 MHz, DMSO-d_6) δ 9.48 (s, 1H), 7.97 (d, J = 8.0 Hz, 1H), 7.32 (s, 1H), 7.28 (d, J = 8.1 Hz, 1H), 5.75 (t, J = 8.4 Hz, 1H), 4.13 (q, J = 9.1 Hz, 1H), 3.99 (td, J = 9.8, 6.1 Hz, 1H), 2.87 (qd, J = 10.5, 8.8, 4.8 Hz, 2H), 2.63 (s, 3H), 2.37 (s, 3H).

LC/MS (APSI) m/z [M+H] calculated for C13H16N3O1: 230.1; found: 230.2.

3-(azetidin-2-yl)-5-(2,5-dimethylphenyl)-1,2,4-oxadiazole - Z3034773290 (Method 5).
Yield: 35%; purity, >95% (assessed by LC/MS).

^{1}H NMR (500 MHz, DMSO-d_6) δ 9.65 (s, 2H), 7.88 (d, J = 1.9 Hz, 1H), 7.42 (dd, J = 7.8, 1.9 Hz, 1H), 7.38 (d, J = 7.9 Hz, 1H), 5.77 (t, J = 8.4 Hz, 1H), 4.14 (q, J = 9.2 Hz, 1H), 4.01 (td, J = 9.8, 6.2 Hz, 1H), 2.88 (qd, J = 10.6, 8.9, 4.8 Hz, 2H), 2.61 (s, 3H), 2.36 (s, 3H), 2.35 (s, 0H).

LC/MS (APSI) m/z [M+H] calculated for C13H16N3O: 230.0; found: 230.1.

3-(2-Azetidinyl)-5-(2,3-xylyl)-1,2,4-oxadiazole - Z8915636241, Z3034773292 (Method 5).
Yield: 62%; purity, >95% (assessed by LC/MS).

^{1}H NMR (500 MHz, DMSO-d_6) δ 9.50 (s, 2H), 7.79 (d, J = 7.9 Hz, 1H), 7.51 (d, J = 7.5 Hz, 1H), 7.34 (t, J = 7.7 Hz, 1H), 5.77 (t, J = 8.5 Hz, 1H), 4.13 (q, J = 9.2 Hz, 1H), 3.99 (td, J = 9.8, 5.9 Hz, 1H), 2.95 - 2.82 (m, 1H), 2.53 (s, 3H), 2.36 (s, 3H).

LC/MS (APSI) m/z [M+H] calculated for C13H16N3O1: 230.1; found: 230.2.

3-(2-Azetidinyl)-5-(5-fluoro-2-tolyl)-1,2,4-oxadiazole - Z8741436112, Z3034773309 (Method 5).
Yield: 25%; purity, >95% (assessed by LC/MS).

^{1}H NMR (500 MHz, DMSO-d_6) δ 9.55 (s, 2H), 7.85 (td, J = 12.0, 10.7, 5.7 Hz, 1H), 7.57 (dd, J = 8.6, 5.6 Hz, 1H), 7.50 (td, J = 8.5, 2.9 Hz, 1H), 5.78 (t, J = 8.4 Hz, 1H), 4.13 (q, J = 9.2 Hz, 1H), 4.01 (td, J = 9.6, 6.4 Hz, 1H), 2.87 (dtt, J = 12.5, 6.4, 3.7 Hz, 2H), 2.63 (d, J = 7.3 Hz, 3H).

LC/MS (APSI) m/z [M+H] calculated for C12F1H13N3O1: 234.1; found: 234.2.

3-(2-Azetidinyl)-5-(2-chloro-4-tolyl)-1,2,4-oxadiazole - Z8904328914, Z3034773352 (Method 5).
Yield: 8%; purity, 93% (assessed by LC/MS).

^{1}H NMR (600 MHz, DMSO-d_6) δ 9.50 (s, 2H), 8.06 (d, J = 8.0 Hz, 1H), 7.64 (s, 1H), 7.48 - 7.44 (m, 1H), 5.79 (t, J = 8.4 Hz, 1H), 4.14 (q, J = 9.2 Hz, 1H), 3.99 (td, J = 9.9, 5.9 Hz, 1H), 2.96 - 2.88 (m, 1H), 2.86 (ddd, J = 12.1, 7.3, 2.9 Hz, 1H), 2.43 (s, 3H).

LC/MS (APSI) m/z [M+H] calculated for C12Cl1H13N3O1: 250.1; found: 250.0.

3-(azetidin-2-yl)-5-(3-chloro-5-methylphenyl)-1,2,4-oxadiazole - Z3034773353 (Method 5).
Yield: 18%; purity, >95% (assessed by LC/MS).

^{1}H NMR (600 MHz, DMSO-d_6) δ 7.97 (d, J = 5.8 Hz, 2H), 7.71 (s, 1H), 5.77 (t, J = 8.4 Hz, 1H), 4.14 (q, J = 9.2 Hz, 1H), 4.04 - 3.97 (m, 1H), 2.92 - 2.84 (m, 1H), 2.45 (s, 3H), 1.24 (s, 1H).

LC/MS (APSI) m/z [M+H] calculated for C12H13ClN3O: 250.0; found: 250.1.

3-(azetidin-2-yl)-5-(3-chloro-2-methylphenyl)-1,2,4-oxadiazole - Z3034773358 (Method 5).
Yield: 13%; purity, >95% (assessed by LC/MS).

^{1}H NMR (600 MHz, DMSO-d_6) δ 7.97 (d, J = 5.8 Hz, 2H), 7.71 (s, 1H), 5.77 (t, J = 8.4 Hz, 1H), 4.14 (q, J = 9.3 Hz, 1H), 4.04 - 3.97 (m, 1H), 2.92 - 2.84 (m, 1H), 2.45 (s, 3H), 1.24 (s, 1H).

LC/MS (APSI) m/z [M+H] calculated for C12H13ClN3O: 250.2; found: 250.1.

3-(azetidin-2-yl)-5-(4-chloro-2-methylphenyl)-1,2,4-oxadiazole - Z3034773359 (Method 5).
Yield: 24%; purity, >95% (assessed by LC/MS).

^{1}H NMR (500 MHz, DMSO-d_6) δ 9.53 (s, 1H), 8.08 (d, J = 8.4 Hz, 1H), 7.66 (d, J = 2.2 Hz, 1H), 7.55 (dd, J = 8.5, 2.3 Hz, 1H), 7.37 - 7.29 (m, 1H), 5.77 (t, J = 8.4 Hz, 1H), 4.12 (q, J = 9.2 Hz, 1H), 3.99 (td, J = 9.7, 6.4 Hz, 1H), 2.92 - 2.81 (m, 2H), 2.67 (s, 3H).

LC/MS (APSI) m/z [M+H] calculated for C12H13ClN3O: 250.0; found: 250.1.

3-(2-Azetidinyl)-5-(2-chloro-3-tolyl)-1,2,4-oxadiazole - Z8731733054, Z3034773361 (Method 5).
Yield: 26%; purity, >95% (assessed by LC/MS).

^{1}H NMR (500 MHz, DMSO-d_6) δ 9.53 (s, 2H), 7.92 (d, J = 7.7 Hz, 1H), 7.73 (d, J = 7.6 Hz, 1H), 7.52 (t, J = 7.7 Hz, 1H), 5.80 (t, J = 8.5 Hz, 1H), 4.13 (q, J = 9.2 Hz, 1H), 3.98 (td, J = 9.9, 5.9 Hz, 1H), 2.96 - 2.79 (m, 2H), 2.45 (s, 3H).

LC/MS (APSI) m/z [M+H] calculated for C12Cl1H13N3O1: 250.1; found: 250.0.

3-(azetidin-2-yl)-5-(3-chloro-4-methylphenyl)-1,2,4-oxadiazole - Z3034773362 (Method 5).
Yield: 50%; purity, >95% (assessed by LC/MS).

^{1}H NMR (500 MHz, DMSO-d_6) δ 9.60 (s, 2H), 8.14 (t, J = 2.4 Hz, 1H), 8.03 (dd, J = 7.8, 2.0 Hz, 1H), 7.66 (d, J = 7.9 Hz, 1H), 5.76 (t, J = 8.5 Hz, 1H), 4.13 (q, J = 9.2 Hz, 1H), 4.00 (td, J = 9.8, 6.2 Hz, 1H), 2.94 - 2.79 (m, 2H), 2.45 (d, J = 3.0 Hz, 3H).

LC/MS (APSI) m/z [M+H] calculated for C12H13ClN3O: 250.0; found: 250.1.

3-(2-Azetidinyl)-5-(3-chloro-4-fluorophenyl)-1,2,4-oxadiazole - Z8904328913, Z3034773368 (Method 5).
Yield: 32%; purity, >95% (assessed by LC/MS).

^{1}H NMR (600 MHz, DMSO-d_6) δ 9.55 (s, 2H), 8.38 (dd, J = 6.9, 2.2 Hz, 1H), 8.21 (ddd, J = 8.7, 4.6, 2.2 Hz, 1H), 7.75 (t, J = 8.9 Hz, 1H), 5.78 (t, J = 8.4 Hz, 1H), 4.15 (q, J = 9.1 Hz, 1H), 4.01 (td, J = 9.8, 6.3 Hz, 1H), 2.94 - 2.82 (m, 2H).

LC/MS (APSI) m/z [M+H] calculated for C11Cl1F1H10N3O1: 254.1; found: 254.0.

3-(azetidin-2-yl)-5-(2-chloro-3-fluorophenyl)-1,2,4-oxadiazole - Z3034773369 (Method 5).
Yield: 29%; purity, 92% (assessed by LC/MS).

^{1}H NMR (600 MHz, DMSO-d_6) δ 9.57 (s, 2H), 8.03 (dd, J = 8.0, 1.4 Hz, 1H), 7.84 (td, J = 8.9, 1.5 Hz, 1H), 7.70 (td, J = 8.1, 5.2 Hz, 1H), 5.83 (t, J = 8.5 Hz, 1H), 4.16 (q, J = 9.1 Hz, 1H), 4.01 (td, J = 9.9, 6.0 Hz, 1H), 2.97 - 2.89 (m, 1H), 2.91 - 2.83 (m, 1H).

LC/MS (APSI) m/z [M+H] calculated for C11H10ClFN3O: 254.0; found: 254.0.

3-(2-Azetidinyl)-5-(3-chloro-2-fluorophenyl)-1,2,4-oxadiazole - Z8741436115, Z3034773370 (Method 5).
Yield: 38%; purity, >95% (assessed by LC/MS).

^{1}H NMR (500 MHz, DMSO-d_6) δ 9.59 - 9.55 (m, 2H), 8.14 (t, J = 7.1 Hz, 1H), 8.01 (t, J = 7.5 Hz, 1H), 7.52 (t, J = 8.0 Hz, 1H), 5.80 (t, J = 8.5 Hz, 1H), 4.14 (q, J = 9.2 Hz, 1H), 3.98 (td, J = 9.8, 6.0 Hz, 1H), 2.95 - 2.83 (m, 2H).

LC/MS (APSI) m/z [M+H] calculated for C11Cl1F1H10N3O1: 254.0; found: 254.0.

3-(azetidin-2-yl)-5-(2-chloro-6-fluorophenyl)-1,2,4-oxadiazole - Z3034773371 (Method 5).
Yield: 20%; purity, >95% (assessed by LC/MS).

^{1}H NMR (500 MHz, DMSO-d_6) δ 9.64 (s, 2H), 7.82 (td, J = 8.3, 6.0 Hz, 1H), 7.67 (d, J = 8.1 Hz, 1H), 7.58 (t, J = 9.1 Hz, 1H), 5.85 (t, J = 8.5 Hz, 1H), 4.15 (q, J = 9.2 Hz, 1H), 3.97 (td, J = 9.9, 5.7 Hz, 1H), 2.97 - 2.80 (m, 2H).

LC/MS (APSI) m/z [M+H] calculated for C11H10ClFN3O: 254.2; found: 254.0.

3-(2-Azetidinyl)-5-(2-chloro-4-fluorophenyl)-1,2,4-oxadiazole - Z8731733014, Z3034773372 (Method 5).
Yield: 30%; purity, >95% (assessed by LC/MS).

^{1}H NMR (500 MHz, DMSO-d_6) δ 9.46 (s, 2H), 8.22 (dd, J = 8.8, 6.0 Hz, 1H), 7.85 (dd, J = 8.9, 2.6 Hz, 1H), 7.54 (td, J = 8.4, 2.7 Hz, 1H), 5.78 (t, J = 8.5 Hz, 1H), 4.12 (q, J = 9.2 Hz, 1H), 3.98 (td, J = 9.8, 6.0 Hz, 1H), 2.87 (dddt, J = 11.9, 8.9, 6.1, 3.1 Hz, 2H).

LC/MS (APSI) m/z [M+H] calculated for C11Cl1F1H10N3O1: 254.0; found: 254.0.

3-(2-Azetidinyl)-5-(5-chloro-2-fluorophenyl)-1,2,4-oxadiazole - Z8727396152, Z3034773374 (Method 5).
Yield: 17%; purity, >95% (assessed by LC/MS).

^{1}H NMR (500 MHz, DMSO-d_6) δ 9.65 (s, 2H), 8.18 (dd, J = 6.1, 2.8 Hz, 1H), 7.90 (dt, J = 9.3, 3.4 Hz, 1H), 7.64 (t, J = 9.6 Hz, 1H), 5.80 (t, J = 8.4 Hz, 1H), 4.14 (q, J = 9.2 Hz, 1H), 4.01 (td, J = 9.7, 6.2 Hz, 1H), 2.95 - 2.80 (m, 2H).

LC/MS (APSI) m/z [M+H] calculated for C11Cl1F1H10N3O1: 254.1; found: 254.0.

3-(2-Azetidinyl)-5-(2-chloro-5-fluorophenyl)-1,2,4-oxadiazole - Z8731642722, Z3034773375 (Method 5).
Yield: 39%; purity, >95% (assessed by LC/MS).

^{1}H NMR (500 MHz, DMSO-d_6) δ 9.61 (s, 2H), 7.99 (dd, J = 8.7, 3.1 Hz, 1H), 7.85 (dd, J = 9.0, 4.9 Hz, 1H), 7.67 (td, J = 8.4, 3.1 Hz, 1H), 5.80 (t, J = 8.4 Hz, 1H), 4.14 (q, J = 9.2 Hz, 1H), 4.00 (td, J = 9.7, 6.1 Hz, 1H), 2.95 - 2.86 (m, 1H), 2.89 - 2.81 (m, 1H).

LC/MS (APSI) m/z [M+H] calculated for C11Cl1F1H10N3O1: 254.0; found: 254.1.

3-(2-Azetidinyl)-5-(2,5-dichlorophenyl)-1,2,4-oxadiazole - Z8741436242, Z3034773378 (Method 5).
Yield: 19%; purity, >95% (assessed by LC/MS).

^{1}H NMR (500 MHz, DMSO-d_6) δ 9.56 (s, 2H), 8.17 (d, J = 2.3 Hz, 1H), 7.83 (d, J = 3.4 Hz, 2H), 5.80 (t, J = 8.5 Hz, 1H), 4.13 (q, J = 9.2 Hz, 1H), 4.00 (td, J = 9.7, 6.2 Hz, 1H), 2.87 (qd, J = 10.5, 9.4, 3.6 Hz, 2H).

LC/MS (APSI) m/z [M+H] calculated for C11Cl2H10N3O1: 270.0; found: 270.0.

3-(azetidin-2-yl)-5-(3,4-dichlorophenyl)-1,2,4-oxadiazole - Z3034773379 (Method 5).
Yield: 22%; purity, >95% (assessed by LC/MS).

^{1}H NMR (600 MHz, DMSO-d_6) δ 9.53 (s, 2H), 8.38 (d, J = 2.0 Hz, 1H), 8.14 (dd, J = 8.4, 2.1 Hz, 1H), 7.98 (d, J = 8.4 Hz, 1H), 5.79 (t, J = 8.4 Hz, 1H), 4.14 (q, J = 9.1 Hz, 1H), 4.01 (td, J = 9.7, 6.2 Hz, 1H), 2.94 - 2.82 (m, 2H).

LC/MS (APSI) m/z [M+H] calculated for C11H10Cl2N3O: 270.0; found: 270.0.

3-(2-Azetidinyl)-5-(2,4-dichlorophenyl)-1,2,4-oxadiazole - Z8727394867, Z3034773380 (Method 5).
Yield: 33%; purity, 92% (assessed by LC/MS).

^{1}H NMR (500 MHz, DMSO-d_6) δ 9.51 (s, 2H), 8.16 (d, J = 8.5 Hz, 1H), 8.01 (d, J = 2.1 Hz, 1H), 7.74 (dd, J = 8.5, 2.1 Hz, 1H), 5.79 (t, J = 8.5 Hz, 1H), 4.13 (q, J = 9.2 Hz, 1H), 3.98 (td, J = 9.8, 6.1 Hz, 1H), 2.87 (qd, J = 10.7, 9.1, 4.7 Hz, 2H).

LC/MS (APSI) m/z [M+H] calculated for C11Cl2H10N3O1: 272.0; found: 272.0.

3-(azetidin-2-yl)-5-(3,5-dichlorophenyl)-1,2,4-oxadiazole - Z3034773381 (Method 5).
Yield: 26%; purity, 91% (assessed by LC/MS).

^{1}H NMR (500 MHz, DMSO-d_6) δ 9.53 (s, 2H), 8.16 (d, J = 2.1 Hz, 1H), 8.13 - 8.04 (m, 1H), 7.83 (dd, J = 12.9, 2.0 Hz, 0H), 5.78 (t, J = 8.4 Hz, 1H), 4.13 (q, J = 9.1 Hz, 1H), 4.01 (td, J = 9.5, 6.5 Hz, 1H), 2.86 (dqd, J = 9.6, 6.8, 6.1, 3.3 Hz, 2H), 2.76 (s, 1H).

LC/MS (APSI) m/z [M+H] calculated for C11H10Cl2N3O: 270.0; found: 270.0.

3-(2-Azetidinyl)-5-(2,6-dichlorophenyl)-1,2,4-oxadiazole - Z9089437746, Z3034773382 (Method 5).
Yield: 42%; purity, 91% (assessed by LC/MS).

^{1}H NMR (500 MHz, DMSO-d_6) δ 9.68 (s, 2H), 7.83 - 7.73 (m, 3H), 5.88 (t, J = 8.6 Hz, 1H), 4.16 (q, J = 9.2 Hz, 1H), 3.97 (td, J = 9.7, 6.0 Hz, 1H), 2.95 - 2.80 (m, 2H).

LC/MS (APSI) m/z [M+H] calculated for C11Cl2H10N3O1: 270.0; found: 270.0.

3-(2-Azetidinyl)-5-(2,3-dichlorophenyl)-1,2,4-oxadiazole - Z8904332000, Z3034773383 (Method 5).
Yield: 22%; purity, >95% (assessed by LC/MS).

^{1}H NMR (600 MHz, DMSO-d_6) δ 9.55 (s, 2H), 8.11 (dd, J = 7.8, 1.5 Hz, 1H), 8.05 (dd, J = 8.1, 1.5 Hz, 1H), 7.67 (t, J = 8.0 Hz, 1H), 5.82 (t, J = 8.5 Hz, 1H), 4.15 (q, J = 9.1 Hz, 1H), 4.00 (td, J = 9.9, 5.9 Hz, 1H), 2.96 - 2.83 (m, 2H).

LC/MS (APSI) m/z [M+H] calculated for C11Cl2H10N3O1: 270.0; found: 270.0.

3-(azetidin-2-yl)-5-(3-bromo-2-methylphenyl)-1,2,4-oxadiazole - Z3034773386 (Method 5).
Yield: 53%; purity, >95% (assessed by LC/MS).

^{1}H NMR (500 MHz, DMSO-d_6) δ 9.51 (s, 2H), 7.99 (t, J = 8.2 Hz, 2H), 7.41 (t, J = 7.8 Hz, 1H), 5.78 (t, J = 8.4 Hz, 1H), 4.13 (q, J = 9.1 Hz, 1H), 3.99 (td, J = 9.8, 6.1 Hz, 1H), 2.87 (dtd, J = 17.9, 8.7, 4.2 Hz, 2H), 2.71 (s, 3H).

LC/MS (APSI) m/z [M+H] calculated for C12H13BrN3O: 296.0; found: 296.0.

3-(2-Azetidinyl)-5-(4-bromo-2-tolyl)-1,2,4-oxadiazole - Z8727393902, Z3034773392 (Method 5).
Yield: 31%; purity, >95% (assessed by LC/MS).

^{1}H NMR (500 MHz, DMSO-d_6) δ 9.52 (s, 2H), 8.00 (d, J = 8.4 Hz, 1H), 7.80 (s, 1H), 7.69 (dd, J = 8.4, 2.1 Hz, 1H), 5.77 (t, J = 8.4 Hz, 1H), 4.12 (q, J = 9.2 Hz, 1H), 3.99 (td, J = 9.6, 6.3 Hz, 1H), 2.92 - 2.81 (m, 2H), 2.66 (s, 3H).

LC/MS (APSI) m/z [M+H] calculated for C12Br1H13N3O1: 294.0; found: 294.0.

3-[3-(azetidin-2-yl)-1,2,4-oxadiazol-5-yl]-5-chloropyridine - Z3034773663 (Method 5).
Yield: 15%; purity, >95% (assessed by LC/MS).
LC/MS (APSI) m/z [M+H] calculated for C10H10ClN4O: 237.2; found: 237.0.

3-(2-Azetidinyl)-5-(3-fluoro-2-tolyl)-1,2,4-oxadiazole - Z8904328910, Z3034773685 (Method 5).
Yield: 9%; purity, >95% (assessed by LC/MS).
^{1}H NMR (500 MHz, DMSO-d_6) δ 9.60 (s, 2H), 7.90 (d, J = 7.8 Hz, 1H), 7.60 - 7.48 (m, 2H), 5.79 (t, J = 8.4 Hz, 1H), 4.14 (q, J = 9.2 Hz, 1H), 4.01 (td, J = 9.8, 6.2 Hz, 1H), 2.95 - 2.80 (m, 2H), 2.57 (d, J = 2.3 Hz, 3H).
LC/MS (APSI) m/z [M+H] calculated for C12F1H13N3O1: 234.1; found: 234.2.

3-(2-Azetidinyl)-5-(4-methyl-3-pyridyl)-1,2,4-oxadiazole - Z8904328909, Z3034773810 (Method 5).
Yield: 34%; purity, >95% (assessed by LC/MS).
^{1}H NMR (500 MHz, DMSO-d_6) δ 9.77 (s, 1H), 8.71 (s, 1H), 8.35 (s, 3H), 7.57 (s, 1H), 2.97 (s, 4H), 2.68 (d, J = 6.1 Hz, 3H).
LC/MS (APSI) m/z [M+H] calculated for C11H13N4O1: 217.1; found: 217.2.

3-(2-Azetidinyl)-5-(3-chloro-2-pyridyl)-1,2,4-oxadiazole - Z8727393895, Z3034773859 (Method 5).
Yield: 35%; purity, >95% (assessed by LC/MS).
^{1}H NMR (500 MHz, DMSO-d_6) δ 9.46 (s, 2H), 8.80 (td, J = 5.7, 5.1, 2.4 Hz, 1H), 8.33 - 8.26 (m, 1H), 7.79 (dt, J = 8.5, 4.3 Hz, 1H), 3.99 (td, J = 9.9, 6.0 Hz, 1H), 3.62 - 3.47 (m, 1H), 2.98 - 2.81 (m, 2H), 2.14 (qd, J = 6.4, 2.6 Hz, 1H).
LC/MS (APSI) m/z [M+H] calculated for C10Cl1H10N4O1: 237.1; found: 237.0.

3-(azetidin-2-yl)-5-(3-chloro-5-fluorophenyl)-1,2,4-oxadiazole - Z3305295413 (Method 5).
Yield: 26%; purity, >95% (assessed by LC/MS).
^{1}H NMR (500 MHz, DMSO-d_6) δ 9.56 (s, 2H), 8.05 (q, J = 2.3, 1.8 Hz, 1H), 8.00 (dt, J = 8.5, 1.9 Hz, 1H), 7.93 (dt, J = 8.6, 2.2 Hz, 1H), 5.78 (t, J = 8.4 Hz, 1H), 4.13 (q, J = 9.0 Hz, 1H), 4.00 (td, J = 9.7, 6.3 Hz, 1H), 2.93 - 2.79 (m, 2H).
LC/MS (APSI) m/z [M+H] calculated for C11H10ClFN3O: 254.0; found: 254.0.

3-(2-Azetidinyl)-5-(2-chloro-5-tolyl)-1,2,4-oxadiazole - Z8735760397, Z3335760412 (Method 5).
Yield: 39%; purity, >95% (assessed by LC/MS).
^{1}H NMR (500 MHz, DMSO-d_6) δ 9.59 (s, 2H), 7.95 (d, J = 2.3 Hz, 1H), 7.65 (d, J = 8.1 Hz, 1H), 7.55 (dd, J = 8.3, 2.3 Hz, 1H), 5.79 (t, J = 8.4 Hz, 1H), 4.14 (q, J = 9.2 Hz, 1H), 4.00 (td, J = 9.9, 6.0 Hz, 1H), 2.96 - 2.80 (m, 2H), 2.39 (s, 3H), 0.93 (s, 0H).
LC/MS (APSI) m/z [M+H] calculated for C12Cl1H13N3O1: 250.1; found: 250.0.

5-(1,2,5,6-tetrahydropyridin-3-yl)-1,3-benzothiazole - Z3498174416 (Method 3).
Yield: 20%; purity, >95% (assessed by LC/MS).
^{1}H NMR (600 MHz, DMSO-d_6) δ 9.52 - 9.31 (m, 2H), 8.22 - 8.06 (m, 2H), 8.02 (d, J = 1.8 Hz, 1H), 7.56 (dd, J = 8.4, 1.7 Hz, 1H), 6.38 (t, J = 4.1 Hz, 1H), 3.72 (q, J = 2.4 Hz, 2H), 2.90 (t, J = 5.7 Hz, 2H), 2.24 (td, J = 6.0, 3.0 Hz, 2H).
LC/MS (APSI) m/z [M+H] calculated for C12H13N2S: 217.0; found: 217.1.

5-(1,2,5,6-tetrahydropyridin-3-yl)-1,2-benzothiazole - Z8055284773, Z3950610504 (Method 3).
Yield: 45%; purity, >95% (assessed by LC/MS).
^{1}H NMR (500 MHz, DMSO-d_6) δ 9.16 (s, 2H), 9.11 (s, 1H), 8.25 - 8.19 (m, 2H), 7.76 (dd, J = 8.6, 1.8 Hz, 1H), 6.55 - 6.50 (m, 1H), 4.10 (s, 2H), 3.26 (t, J = 6.1 Hz, 2H), 2.52 (d, J = 3.6 Hz, 1H).
LC/MS (APSI) m/z [M+H] calculated for C12H13N2S: 217.0; found: 217.1.

5-(4-methyl-1,2,5,6-tetrahydropyridin-3-yl)-1,2-benzothiazole - Z3950610525 (Method 3).
Yield: 27%; purity, >95% (assessed by LC/MS).
^{1}H NMR (600 MHz, DMSO-d_6) δ 9.39 (d, J = 15.6 Hz, 1H), 8.19 - 8.07 (m, 2H), 8.02 (d, J = 1.8 Hz, 1H), 7.56 (dd, J = 8.5, 1.8 Hz, 1H), 3.72 (q, J = 2.4 Hz, 2H), 2.90 (t, J = 5.7 Hz, 2H), 2.24 (dq, J = 6.1, 3.0 Hz, 2H).
LC/MS (APSI) m/z [M+H] calculated for C13H15N2S: 231.1; found: 231.1.

5-(4-fluoro-2,3-dihydro-1H-inden-5-yl)-1,2,3,6-tetrahydropyridine - Z3991412496 (Method 3).
Yield: 27%; purity, >95% (assessed by LC/MS).
^{1}H NMR (600 MHz, DMSO-d_6) δ 7.07 - 6.94 (m, 2H), 6.03 - 5.90 (m, 1H), 3.44 (s, 2H), 2.99 - 2.78 (m, 7H), 2.21 - 1.99 (m, 4H).
LC/MS (APSI) m/z [M+H] calculated for C14H17FN: 218.2; found: 218.1.

5-{thieno[2,3-b]pyridin-5-yl}-1,2,3,6-tetrahydropyridine - Z4067645979 (Method 3).
Yield: 39%; purity, >95% (assessed by LC/MS).
^{1}H NMR (500 MHz, DMSO-d_6) δ 8.61 (d, J = 2.2 Hz, 1H), 8.19 (d, J = 2.2 Hz, 1H), 7.84 (d, J = 6.0 Hz, 1H), 7.40 (d, J = 6.0 Hz, 1H), 6.35 (d, J = 3.9 Hz, 1H), 3.61 (d, J = 2.5 Hz, 2H), 2.82 (t, J = 5.7 Hz, 2H), 2.17 (dh, J = 5.7, 2.9 Hz, 2H).
LC/MS (APSI) m/z [M+H] calculated for C12H13N2S: 217.1; found: 217.1.

4-methyl-5-{thieno[2,3-b]pyridin-5-yl}-1,2,3,6-tetrahydropyridine - Z4067646017 (Method 3).
Yield: 30%; purity, >95% (assessed by LC/MS).
LC/MS (APSI) m/z [M+H] calculated for C13H15N2S: 231.0; found: 231.1.

4-[3-(2-Azetidinyl)-1,2,4-oxadiazol-5-yl]-2-chlorophenol - Z8908003437, Z4209159476 (Method 5).
Yield: 13%; purity, >95% (assessed by LC/MS).
^{1}H NMR (500 MHz, DMSO-d_6) δ 11.59 (s, 1H), 9.50 (s, 2H), 8.10 (d, J = 2.3 Hz, 1H), 7.96 (dd, J = 8.6, 2.2 Hz, 1H), 7.20 (d, J = 8.5 Hz, 1H), 5.72 (t, J = 8.5 Hz, 1H), 4.12 (q, J = 9.1 Hz, 1H), 4.03 - 3.94 (m, 1H), 2.89 - 2.81 (m, 2H).
LC/MS (APSI) m/z [M+H] calculated for C11Cl1H11N3O2: 252.1; found: 252.0.

m-[3-(2-Azetidinyl)-1,2,4-oxadiazol-5-yl]phenol - Z8727394028, Z4209159788 (Method 5).
Yield: 18%; purity, 93% (assessed by LC/MS).
^{1}H NMR (600 MHz, DMSO-d_6) δ 10.18 (s, 1H), 9.49 (s, 2H), 7.61 - 7.57 (m, 1H), 7.14 (dd, J = 8.1, 2.6 Hz, 1H), 5.76 (t, J = 8.5 Hz, 1H), 4.14 (q, J = 9.1 Hz, 1H), 3.99 (td, J = 9.9, 6.0 Hz, 1H), 2.94 - 2.80 (m, 2H).
LC/MS (APSI) m/z [M+H] calculated for C11H12N3O2: 218.1; found: 218.2.

2-[3-(2-Azetidinyl)-1,2,4-oxadiazol-5-yl]-6-chlorophenol - Z4209160323 (Method 5).
Yield: 25%; purity, >95% (assessed by LC/MS).
^{1}H NMR (500 MHz, DMSO-d_6) δ 10.64 (s, 1H), 9.51 (s, 2H), 7.96 (dd, J = 7.9, 1.6 Hz, 1H), 7.80 (dd, J = 8.0, 1.6 Hz, 1H), 7.13 (t, J = 7.9 Hz, 1H), 5.81 (t, J = 8.5 Hz, 1H), 4.13 (q, J = 9.4 Hz, 1H), 4.01 (q, J = 9.1 Hz, 1H), 2.86 (dtt, J = 12.4, 6.4, 3.6 Hz, 2H).
LC/MS (APSI) m/z [M+H] calculated for C11Cl1H11N3O2: 252.1; found: 252.0.

2-[3-(2-Azetidinyl)-1,2,4-oxadiazol-5-yl]-4-chlorophenol - Z8727395864, Z4209160405 (Method 5).
Yield: 18%; purity, >95% (assessed by LC/MS).
^{1}H NMR (500 MHz, DMSO-d_6) δ 11.17 (s, 1H), 9.52 (s, 1H), 7.94 (d, J = 2.8 Hz, 1H), 7.59 (dd, J = 8.9, 2.8 Hz, 1H), 7.15 (d, J = 8.9 Hz, 1H), 5.76 (t, J = 8.5 Hz, 1H), 4.13 (q, J = 9.2 Hz, 1H), 4.00 (td, J = 9.8, 6.1 Hz, 1H), 2.93 - 2.78 (m, 2H).
LC/MS (APSI) m/z [M+H] calculated for C11Cl1H11N3O2: 252.1; found: 252.0.

o-[3-(2-Azetidinyl)-1,2,4-oxadiazol-5-yl]phenol - Z8735711409, Z4209160875 (Method 5).
Yield: 15%; purity, >95% (assessed by LC/MS).
^{1}H NMR (600 MHz, DMSO-d_6) δ 10.73 (s, 1H), 9.48 (s, 2H), 7.97 (dd, J = 7.9, 1.7 Hz, 1H), 7.58 - 7.53 (m, 1H), 7.14 (d, J = 8.3 Hz, 1H), 7.05 (t, J = 7.5 Hz, 1H), 5.77 (t, J = 8.5 Hz, 1H), 4.14 (q, J = 9.2 Hz, 1H), 4.00 (td, J = 9.9, 5.9 Hz, 1H), 2.95 - 2.80 (m, 2H).
LC/MS (APSI) m/z [M+H] calculated for C11H12N3O2: 218.1; found: 218.0.

3-(2-Azetidinyl)-5-(2-fluoro-6-tolyl)-1,2,4-oxadiazole - Z8890819769, Z4209171692 (Method 5).
Yield: 25%; purity, >95% (assessed by LC/MS).
^{1}H NMR (600 MHz, DMSO-d_6) δ 9.59 (s, 2H), 7.67 (td, J = 8.0, 5.7 Hz, 1H), 7.36 (t, J = 9.0 Hz, 2H), 5.83 (t, J = 8.5 Hz, 1H), 4.23 - 3.95 (m, 2H), 3.05 - 2.75 (m, 2H), 2.48 - 2.40 (m, 1H).
LC/MS (APSI) m/z [M+H] calculated for C12F1H13N3O1: 234.1; found: 234.2.

2-[3-(azetidin-2-yl)-1,2,4-oxadiazol-5-yl]-6-chloropyridine - Z4209177826 (Method 3).
Yield: 17%; purity, >95% (assessed by LC/MS).
LC/MS (APSI) m/z [M+H] calculated for C10H10ClN4O: 237.0; found: 237.0.

4-[3-(azetidin-2-yl)-1,2,4-oxadiazol-5-yl]-2-chloropyridine - Z4209179703 (Method 3).
Yield: 29%; purity, >95% (assessed by LC/MS).
^{1}H NMR (600 MHz, DMSO-*d*$_6$) δ 9.54 (s, 2H), 8.78 (d, J = 5.1 Hz, 1H), 8.20 (s, 1H), 8.11 (dd, J = 5.1, 1.4 Hz, 1H), 5.82 (t, J = 8.5 Hz, 1H), 4.15 (q, J = 9.2 Hz, 1H), 4.01 (td, J = 9.7, 6.4 Hz, 1H), 2.95 - 2.83 (m, 2H).
LC/MS (APSI) m/z [M+H] calculated for C10H10ClN4O: 237.0; found: 237.0.

N-[1-(2,3-dihydro-1,4-benzodioxin-6-yl)-2-methylpropyl]-9H-purin-6-amine - Z4223060659 (Method 2).
Yield: 18%; purity, >95% (assessed by LC/MS).
^{1}H NMR (500 MHz, DMSO-*d*$_6$) δ 13.01 - 12.69 (m, 1H), 8.27 - 7.86 (m, 2H), 7.13 - 6.67 (m, 2H), 4.94 (d, J = 82.6 Hz, 1H), 4.16 (d, J = 6.7 Hz, 3H), 3.29 (s, 3H), 2.11 (d, J = 69.9 Hz, 1H), 0.97 (d, J = 6.4 Hz, 2H), 0.69 (d, J = 6.6 Hz, 2H).
LC/MS (APSI) m/z [M+H] calculated for C17H20N5O: 326.2; found: 326.2.

5-{thieno[3,2-b]pyridin-5-yl}-1,2,3,6-tetrahydropyridine - Z4224285938 (Method 3).
Yield: 44%; purity, >95% (assessed by LC/MS).
^{1}H NMR (500 MHz, DMSO-*d*$_6$) δ 8.37 (d, J = 8.5 Hz, 1H), 8.07 (d, J = 5.5 Hz, 1H), 7.55 (d, J = 8.6 Hz, 1H), 7.49 (d, J = 5.5 Hz, 1H), 6.76 (d, J = 4.3 Hz, 1H), 3.74 - 3.70 (m, 2H), 2.82 (t, J = 5.7 Hz, 2H), 2.19 (hept, J = 2.7 Hz, 2H).
LC/MS (APSI) m/z [M+H] calculated for C12H13N2S: 217.1; found: 217.1.

5-(1,2,5,6-tetrahydropyridin-3-yl)-2,3-dihydro-1H-inden-1-ol - Z4224292450 (Method 3).
Yield: 14%; purity, >95% (assessed by LC/MS).
^{1}H NMR (500 MHz, DMSO-*d*$_6$) δ 7.37 - 7.06 (m, 3H), 6.24 - 6.01 (m, 1H), 5.15 (d, J = 6.2 Hz, 1H), 4.99 (q, J = 6.4 Hz, 1H), 3.53 (q, J = 2.5 Hz, 2H), 2.96 - 2.75 (m, 3H), 2.66 (dt, J = 16.9, 8.4 Hz, 1H), 2.29 (ddd, J = 12.1, 7.8, 3.6 Hz, 1H), 2.13 (s, 2H), 1.74 (dq, J = 14.7, 7.9 Hz, 1H).
LC/MS (APSI) m/z [M+H] calculated for C14H18NO: 216.2; found: 216.1.

2-(1-phenylethenyl)pyridin-4-amine - Z4376630014 (Method 6).
Yield: 15%; purity, >95% (assessed by LC/MS).
^{1}H NMR (500 MHz, DMSO-*d*$_6$) δ 8.00 - 7.95 (m, 1H), 7.39 - 7.24 (m, 5H), 6.38 (d, J = 4.4 Hz, 2H), 5.96 (d, J = 5.6 Hz, 1H), 5.82 (d, J = 2.1 Hz, 1H), 5.39 (d, J = 2.1 Hz, 1H), 2.52 (s, 1H).
LC/MS (APSI) m/z [M+H] calculated for C13H13N2: 197.2; found: 197.1.

1-(1-cyclobutanecarbonylpiperidin-3-yl)-3-[2-(dimethylamino)-2-(thiophen-3-yl)ethyl]urea - Z4407498716 (Method 7).
Yield: 53%; purity, >95% (assessed by LC/MS).
^{1}H NMR (500 MHz, DMSO-*d*$_6$) δ 7.47 (dt, J = 5.3, 2.6 Hz, 1H), 7.30 (d, J = 2.9 Hz, 1H), 7.02 (d, J = 4.9 Hz, 1H), 6.25 - 5.90 (m, 1H), 5.86 - 5.57 (m, 1H), 4.06 - 3.82 (m, 1H), 3.56 (qd, J = 8.5, 7.4, 4.9 Hz, 1H), 3.43 - 3.37 (m, 1H), 3.28 (s, 2H), 3.20 (d, J = 8.6 Hz, 1H), 2.96 - 2.86 (m, 1H), 2.13 (ddt, J = 38.2, 19.5, 9.1 Hz, 2H), 2.03 (s, 6H), 2.02 (s, 1H), 1.93 (dtt, J = 11.5, 7.5, 3.7 Hz, 1H), 1.84 (dddd, J = 10.8, 9.0, 6.3, 3.2 Hz, 1H), 1.70 (s, 2H), 1.56 (d, J = 10.3 Hz, 1H), 1.35 - 1.23 (m, 2H).
LC/MS (APSI) m/z [M+H] calculated for C19H31N4O2S: 379.2; found: 379.2.

3-[3-(2-Azetidinyl)-1,2,4-oxadiazol-5-yl]-2-chlorophenol - Z8904332024, Z4445120248 (Method 5).
Yield: 24%; purity, >95% (assessed by LC/MS).
LC/MS (APSI) m/z [M+H] calculated for C11Cl1H11N3O2: 252.1; found: 252.0.

5-(7-methyl-1-benzothiophen-5-yl)-1,2,3,6-tetrahydropyridine - Z4765367699 (Method 3).
Yield: 17%; purity, >95% (assessed by LC/MS).
^{1}H NMR (600 MHz, DMSO-*d*$_6$) δ 7.72 (d, J = 5.4 Hz, 3H), 7.52 - 7.42 (m, 1H), 7.27 (s, 1H), 6.37 - 6.22 (m, 1H), 3.62 (q, J = 2.4 Hz, 2H), 2.84 (t, J = 5.7 Hz, 2H), 2.68 - 2.56 (m, 1H), 2.17 (tt, J = 5.8, 2.8 Hz, 2H).
LC/MS (APSI) m/z [M+H] calculated for C14H16NS: 230.0; found: 230.1.

4-methyl-5-(7-methyl-1-benzothiophen-5-yl)-1,2,3,6-tetrahydropyridine - Z4765367717 (Method 3).
Yield: 35%; purity, >95% (assessed by LC/MS).
^{1}H NMR (500 MHz, DMSO-d_6) δ 7.79 - 7.66 (m, 1H), 7.53 - 7.46 (m, 1H), 7.43 (d, J = 5.4 Hz, 1H), 6.98 (s, 1H), 3.34 (s, 4H), 2.85 (t, J = 5.8 Hz, 2H), 2.01 (s, 2H), 1.55 (s, 3H).
LC/MS (APSI) m/z [M+H] calculated for C15H18NS: 244.2; found: 244.1.

N-(7-methylnaphthalen-1-yl)pyridin-4-amine - Z4954471231, Z4767467063 (Method 2).
Yield: 34%; purity, >95% (assessed by LC/MS).
^{1}H NMR (600 MHz, DMSO-d_6) δ 8.89 (s, 1H), 8.17 - 8.11 (m, 2H), 7.87 (d, J = 8.3 Hz, 1H), 7.79 (s, 1H), 7.73 (dd, J = 7.6, 1.7 Hz, 1H), 7.49 - 7.37 (m, 3H), 6.78 - 6.73 (m, 2H), 2.46 (s, 3H).
LC/MS (APSI) m/z [M+H] calculated for C16H15N2: 235.2; found: 235.1.

N-(4-fluoronaphthalen-1-yl)pyridin-4-amine - Z4767467064 (Method 2).
Yield: 54%; purity, >95% (assessed by LC/MS).
^{1}H NMR (500 MHz, DMSO-d_6) δ 8.77 (s, 1H), 8.12 - 8.05 (m, 3H), 7.98 (d, J = 8.4 Hz, 1H), 7.70 - 7.59 (m, 2H), 7.41 (dd, J = 8.2, 4.9 Hz, 1H), 7.34 (dd, J = 10.5, 8.2 Hz, 1H), 6.65 - 6.60 (m, 2H).
LC/MS (APSI) m/z [M+H] calculated for C15H12FN2: 239.1; found: 239.1.

N-(pyridin-4-yl)isoquinolin-5-amine - Z4767467067 (Method 2).
Yield: 18%; purity, >95% (assessed by LC/MS).
^{1}H NMR (500 MHz, DMSO-d_6) δ 9.33 (s, 1H), 9.02 (s, 1H), 8.51 (d, J = 5.9 Hz, 1H), 8.17 (d, J = 5.8 Hz, 2H), 7.92 (d, J = 7.9 Hz, 1H), 7.84 (d, J = 5.9 Hz, 1H), 7.76 - 7.63 (m, 2H), 6.84 - 6.79 (m, 2H).
LC/MS (APSI) m/z [M+H] calculated for C14H12N3: 222.2; found: 222.2.

N-(pyridin-4-yl)isoquinolin-1-amine - Z4767467070 (Method 2).
Yield: 47%; purity, >95% (assessed by LC/MS).
^{1}H NMR (500 MHz, DMSO-d_6) δ 9.54 (s, 1H), 8.53 (d, J = 8.5 Hz, 1H), 8.38 - 8.33 (m, 2H), 8.12 (d, J = 5.7 Hz, 1H), 7.93 - 7.86 (m, 3H), 7.75 (t, J = 7.5 Hz, 1H), 7.67 (t, J = 7.7 Hz, 1H), 7.35 (d, J = 5.7 Hz, 1H).
LC/MS (APSI) m/z [M+H] calculated for C14H12N3: 222.2; found: 222.2.

N-(pyridin-4-yl)quinolin-4-amine - Z4767467072 (Method 2).
Yield: 34%; purity, >95% (assessed by LC/MS).
^{1}H NMR (500 MHz, DMSO-d_6) δ 9.34 (s, 1H), 8.65 (d, J = 5.1 Hz, 1H), 8.39 - 8.34 (m, 2H), 8.28 (dd, J = 8.5, 1.3 Hz, 1H), 7.94 (d, J = 8.4 Hz, 1H), 7.74 (ddd, J = 8.3, 6.7, 1.4 Hz, 1H), 7.58 (ddd, J = 8.4, 6.6, 1.3 Hz, 1H), 7.36 (d, J = 5.1 Hz, 1H), 7.26 - 7.22 (m, 2H).
LC/MS (APSI) m/z [M+H] calculated for C14H12N3: 222.2; found: 222.2.

5-(4-chloro-2,3-dihydro-1H-inden-5-yl)-1,2,3,6-tetrahydropyridine - Z4767467074 (Method 6).
Yield: 33%; purity, >95% (assessed by LC/MS).
^{1}H NMR (600 MHz, DMSO-d_6) δ 9.00 - 8.97 (m, 2H), 7.21 (d, J = 7.6 Hz, 1H), 7.07 (d, J = 7.5 Hz, 1H), 5.90 (q, J = 2.8, 1.9 Hz, 1H), 3.80 (d, J = 2.5 Hz, 2H), 3.25 (d, J = 12.3 Hz, 1H), 2.96 (t, J = 7.5 Hz, 2H), 2.91 (t, J = 7.5 Hz, 2H), 2.44 (p, J = 3.1 Hz, 2H), 2.06 (p, J = 7.5 Hz, 2H).
LC/MS (APSI) m/z [M+H] calculated for C14H17ClN: 234.2; found: 234.1.

5-(2,3-dihydro-1H-inden-5-yl)-3-methyl-1,2,3,6-tetrahydropyridine - Z4767467093 (Method 3).
Yield: 23%; purity, >95% (assessed by LC/MS).
^{1}H NMR (600 MHz, DMSO-d_6) δ 8.51 (dd, J = 160.8, 2.1 Hz, 1H), 7.97 - 7.49 (m, 1H), 7.29 - 7.02 (m, 2H), 3.05 - 2.74 (m, 9H), 2.12 - 1.92 (m, 5H), 1.10 - 0.74 (m, 2H).
LC/MS (APSI) m/z [M+H] calculated for C15H20N: 214.2; found: 214.2.

5-{5H,6H,7H-cyclopenta[c]pyridin-3-yl}-1,2,3,6-tetrahydropyridine - Z4955958409 (Method 3).
Yield: 15%; purity, >95% (assessed by LC/MS).
^{1}H NMR (500 MHz, DMSO-d_6) δ 8.48 - 8.20 (m, 1H), 7.66 - 7.32 (m, 1H), 6.64 (d, J = 5.0 Hz, 1H), 3.73 - 3.62 (m, 1H), 3.04 - 2.75 (m, 8H), 2.20 (s, 1H), 2.03 (dp, J = 22.1, 7.4 Hz, 3H).
LC/MS (APSI) m/z [M+H] calculated for C13H17N2: 201.2; found: 201.1.

5-{5H,6H,7H-cyclopenta[b]pyridin-3-yl}-1,2,3,6-tetrahydropyridine - Z4957966902 (Method 3).
Yield: 23%; purity, >95% (assessed by LC/MS).
^{1}H NMR (600 MHz, DMSO-d_6) δ 8.39 - 8.17 (m, 1H), 7.71 - 7.50 (m, 1H), 6.37 - 6.15 (m, 1H), 3.68 - 3.47 (m, 2H), 3.07 - 2.76 (m, 7H), 2.22 - 1.89 (m, 4H).
LC/MS (APSI) m/z [M+H] calculated for C13H17N2: 201.2; found: 201.1.

5-(7-methyl-2,3-dihydro-1H-inden-5-yl)-1,2,3,6-tetrahydropyridine - Z5022589075 (Method 3).
Yield: 24%; purity, >95% (assessed by LC/MS).
^{1}H NMR (500 MHz, Methanol-d_4) δ 7.00 (s, 1H), 6.91 (s, 1H), 6.10 (tt, J = 4.0, 1.8 Hz, 1H), 3.63 (q, J = 2.4 Hz, 2H), 2.95 (t, J = 5.8 Hz, 2H), 2.87 (t, J = 7.5 Hz, 2H), 2.79 (t, J = 7.4 Hz, 2H), 2.34 - 2.19 (m, 3H), 2.22 (s, 3H), 2.10 - 2.00 (m, 2H), 2.01 (s, 1H).
LC/MS (APSI) m/z [M+H] calculated for C15H20N: 214.2; found: 214.2.

5-(4-methyl-1,2,5,6-tetrahydropyridin-3-yl)-1,3-benzothiazole - Z5106507813 (Method 3).
Yield: 25%; purity, >95% (assessed by LC/MS).
^{1}H NMR (600 MHz, DMSO-d_6) δ 9.49 - 9.35 (m, 1H), 8.23 - 8.01 (m, 1H), 7.97 - 7.79 (m, 1H), 7.48 - 7.23 (m, 1H), 3.52 - 3.40 (m, 1H), 2.93 (t, J = 5.8 Hz, 1H), 2.31 (s, 1H), 2.24 - 2.04 (m, 1H), 1.61 (d, J = 18.7 Hz, 4H).
LC/MS (APSI) m/z [M+H] calculated for C13H15N2S: 231.2; found: 231.1.

3-[2-oxo-2-(2,3,4,5-tetrahydro-1,5-benzothiazepin-5-yl)ethyl]-1,3-dihydro-2-benzofuran-1-one - Z52076138 (Method 1).
Yield: 74%; purity, >95% (assessed by LC/MS).
^{1}H NMR (500 MHz, DMSO-d_6) δ 7.75 (dd, J = 7.7, 5.2 Hz, 1H), 7.71 (q, J = 7.8 Hz, 1H), 7.63 (t, J = 8.2 Hz, 1H), 7.62 - 7.50 (m, 2H), 7.38 (ddd, J = 19.1, 6.6, 2.5 Hz, 1H), 7.34 (dd, J = 8.5, 1.8 Hz, 1H), 7.30 - 7.21 (m, 1H), 5.94 - 5.85 (m, 1H), 4.54 (ddd, J = 12.5, 8.3, 3.9 Hz, 1H), 2.95 - 2.87 (m, 1H), 2.68 (ddt, J = 16.2, 11.9, 7.8 Hz, 3H), 2.26 (dd, J = 16.4, 5.8 Hz, 1H), 2.18 - 2.01 (m, 1H), 2.04 - 1.97 (m, 1H).
LC/MS (APSI) m/z [M+H] calculated for C19H18NO3S: 340.0; found: 340.1.

3-methyl-N-(naphthalen-1-yl)pyridin-4-amine - Z5290600172 (Method 2).
Yield: 56%; purity, >95% (assessed by LC/MS).
^{1}H NMR (500 MHz, DMSO-d_6) δ 8.09 (d, J = 3.4 Hz, 1H), 8.00 - 7.94 (m, 2H), 7.84 (dd, J = 10.4, 7.1 Hz, 3H), 7.58 - 7.44 (m, 3H), 7.37 (t, J = 5.3 Hz, 1H), 6.07 (t, J = 5.3 Hz, 1H), 2.29 (d, J = 3.4 Hz, 3H).
LC/MS (APSI) m/z [M+H] calculated for C16H15N2: 235.2; found: 235.1.

N-(pyridin-4-yl)isoquinolin-4-amine - Z5295862251 (Method 2).
Yield: 12%; purity, >95% (assessed by LC/MS).
^{1}H NMR (600 MHz, DMSO-d_6) δ 9.18 (s, 1H), 8.94 (s, 1H), 8.50 (s, 1H), 8.19 (d, J = 8.2 Hz, 1H), 8.18 - 8.14 (m, 2H), 7.98 (d, J = 8.4 Hz, 1H), 7.82 (dd, J = 8.4, 6.9 Hz, 1H), 7.74 (t, J = 7.5 Hz, 1H), 6.78 - 6.73 (m, 2H).
LC/MS (APSI) m/z [M+H] calculated for C14H12N3: 222.2; found: 222.1.

3-(4-bromophenyl)-3-({7H-pyrrolo[2,3-d]pyrimidin-4-yl}formamido)propanoic acid - Z5398393122 (Method 8).
Yield: 57%; purity, >95% (assessed by LC/MS).
^{1}H NMR (500 MHz, DMSO-d_6) δ 12.36 (s, 1H), 9.57 (d, J = 8.7 Hz, 1H), 8.85 (s, 1H), 7.71 (t, J = 2.9 Hz, 1H), 7.50 (d, J = 8.1 Hz, 2H), 7.41 (d, J = 8.2 Hz, 2H), 6.98 (d, J = 3.2 Hz, 1H), 5.42 (q, J = 7.6 Hz, 1H), 3.05 (dd, J = 16.2, 8.0 Hz, 1H), 2.87 (dd, J = 16.2, 6.1 Hz, 1H).
LC/MS (APSI) m/z [M+H] calculated for C16H14BrN4O3: 389.0; found: 389.0.

5-(6-chloro-2,3-dihydro-1H-inden-5-yl)-1,2,3,6-tetrahydropyridine - Z5425693717 (Method 3).
Yield: 36%; purity, 91% (assessed by LC/MS).
LC/MS (APSI) m/z [M+H] calculated for C14H16ClN: 234.0; found: 234.1.

5-(3-methyl-1-benzothiophen-5-yl)-1,2,3,6-tetrahydropyridine - Z5458682006 (Method 3).
Yield: 14%; purity, >95% (assessed by LC/MS).
^{1}H NMR (500 MHz, DMSO-d_6) δ 7.86 (d, J = 8.4 Hz, 1H), 7.65 (d, J = 1.8 Hz, 1H), 7.39 (dd, J = 8.5, 1.8 Hz, 1H), 7.35 (d, J = 1.4 Hz, 1H), 6.26 (tt, J = 4.1, 1.8 Hz, 1H), 3.62 (q, J = 2.4 Hz, 2H), 2.81 (t, J = 5.7 Hz, 2H), 2.52 (s, 1H), 2.38 (d, J = 1.2 Hz, 3H), 2.16 (dp, J = 6.0, 2.9 Hz, 2H).

LC/MS (APSI) m/z [M+H] calculated for C14H16NS: 230.2; found: 230.1.

4-methyl-5-(3-methyl-1-benzothiophen-5-yl)-1,2,3,6-tetrahydropyridine - Z5458682044 (Method 3).
Yield: 23%; purity, >95% (assessed by LC/MS).

^{1}H NMR (500 MHz, DMSO-d_6) δ 8.43 (q, J = 2.7 Hz, 1H), 8.14 - 7.68 (m, 1H), 7.53 - 7.42 (m, 1H), 7.42 - 7.29 (m, 1H), 7.14 (dd, J = 8.2, 1.7 Hz, 1H), 3.38 (t, J = 2.6 Hz, 1H), 2.88 (t, J = 5.8 Hz, 1H), 2.45 - 2.15 (m, 5H), 2.11 - 1.79 (m, 1H), 1.56 (s, 2H).

LC/MS (APSI) m/z [M+H] calculated for C15H18NS: 244.2; found: 244.1.

2-methyl-6-(1-phenylethenyl)pyridin-4-amine - Z5498147476 (Method 6).
Yield: 35%; purity, >95% (assessed by LC/MS).

^{1}H NMR (500 MHz, DMSO-d_6) δ 7.35 (ddd, J = 7.6, 6.0, 1.6 Hz, 2H), 7.34 - 7.26 (m, 2H), 7.29 - 7.24 (m, 1H), 6.25 (d, J = 2.1 Hz, 1H), 6.18 (d, J = 2.1 Hz, 1H), 5.85 (s, 2H), 5.80 (d, J = 2.2 Hz, 1H), 5.36 (d, J = 2.2 Hz, 1H), 2.23 (d, J = 2.4 Hz, 3H).

LC/MS (APSI) m/z [M+H] calculated for C14H15N2: 211.2; found: 211.1.

5-(7-fluoro-2,3-dihydro-1H-inden-5-yl)-1,2,3,6-tetrahydropyridine - Z5766294474 (Method 3).
Yield: 23%; purity, >95% (assessed by LC/MS).

^{1}H NMR (500 MHz, DMSO-d_6) δ 9.33 (s, 2H), 7.14 (s, 1H), 7.04 (d, J = 10.7 Hz, 1H), 6.39 - 6.34 (m, 1H), 3.92 (s, 2H), 3.17 (q, J = 5.9 Hz, 2H), 2.87 (dt, J = 15.5, 7.4 Hz, 4H), 2.43 (d, J = 6.2 Hz, 2H), 2.05 (p, J = 7.5 Hz, 2H).

LC/MS (APSI) m/z [M+H] calculated for C14H17FN: 218.0; found: 218.1.

N-(pyridin-4-yl)isoquinolin-8-amine - Z5874039302 (Method 2).
Yield: 15%; purity, >95% (assessed by LC/MS).

^{1}H NMR (500 MHz, DMSO-d_6) δ 9.44 (d, J = 3.3 Hz, 1H), 9.18 - 9.14 (m, 1H), 8.51 (dd, J = 5.8, 3.2 Hz, 1H), 8.21 (d, J = 4.8 Hz, 2H), 7.85 - 7.79 (m, 1H), 7.72 (dq, J = 10.1, 6.6, 4.9 Hz, 2H), 7.64 (s, 1H), 7.58 (dd, J = 7.5, 3.3 Hz, 1H), 6.91 (d, J = 4.8 Hz, 2H).

LC/MS (APSI) m/z [M+H] calculated for C14H11N3: 222.2; found: 222.1.

2-[1-(3-methylphenyl)ethenyl]pyridin-4-amine - Z6071720064 (Method 6).
Yield: 36%; purity, >95% (assessed by LC/MS).

LC/MS (APSI) m/z [M+H] calculated for C14H15N2: 211.0; found: 211.1.

2-[1-(2-chlorophenyl)ethenyl]pyridin-4-amine - Z6071720076 (Method 6).
Yield: 46%; purity, >95% (assessed by LC/MS).

^{1}H NMR (500 MHz, DMSO-d_6) δ 7.95 (d, J = 5.5 Hz, 1H), 7.46 (dt, J = 7.6, 3.6 Hz, 1H), 7.38 (dt, J = 5.9, 3.3 Hz, 2H), 7.31 (dt, J = 5.7, 3.4 Hz, 1H), 6.34 (dt, J = 5.5, 2.5 Hz, 1H), 6.27 (t, J = 2.5 Hz, 1H), 6.14 (d, J = 2.3 Hz, 1H), 5.93 (s, 2H), 5.22 (d, J = 2.3 Hz, 1H).

LC/MS (APSI) m/z [M+H] calculated for C13H12ClN2: 231.0; found: 231.1.

2-[1-(4-fluorophenyl)ethenyl]pyridin-4-amine - Z6071720077 (Method 6).
Yield: 15%; purity, >95% (assessed by LC/MS).

^{1}H NMR (600 MHz, DMSO-d_6) δ 7.99 (d, J = 5.4 Hz, 1H), 7.36 - 7.30 (m, 2H), 7.22 - 7.15 (m, 2H), 6.41 (d, J = 5.0 Hz, 2H), 5.99 (d, J = 6.7 Hz, 2H), 5.81 (d, J = 1.8 Hz, 1H), 5.41 (d, J = 1.8 Hz, 1H).

LC/MS (APSI) m/z [M+H] calculated for C13H12FN2: 215.2; found: 215.1.

2-[1-(3-fluorophenyl)ethenyl]pyridin-4-amine - Z6071720079 (Method 6).
Yield: 30%; purity, >95% (assessed by LC/MS).

^{1}H NMR (500 MHz, DMSO-d_6) δ 7.98 (d, J = 5.4 Hz, 1H), 7.40 (td, J = 8.0, 6.2 Hz, 1H), 7.19 - 7.06 (m, 3H), 6.44 - 6.38 (m, 2H), 6.07 (s, 2H), 5.84 (d, J = 1.6 Hz, 1H), 5.50 (d, J = 1.7 Hz, 1H).

LC/MS (APSI) m/z [M+H] calculated for C13H12FN2: 215.2; found: 215.1.

2-[1-(4-chlorophenyl)ethenyl]pyridin-4-amine - Z6071720081 (Method 6).
Yield: 14%; purity, >95% (assessed by LC/MS).

LC/MS (APSI) m/z [M+H] calculated for C13H12ClN2: 231.0; found: 231.1.

3-[2-(8-chloro-2,3,4,5-tetrahydro-1,5-benzothiazepin-5-yl)-2-oxoethyl]-1,3-dihydro-2-benzofuran-1-one - Z6755334561 (Method 1).

Yield: 40%; purity, >95% (assessed by LC/MS).

^{1}H NMR (500 MHz, DMSO-d_6) δ 7.76 (t, J = 6.6 Hz, 1H), 7.71 (q, J = 7.3 Hz, 1H), 7.66 - 7.60 (m, 2H), 7.54 (t, J = 7.5 Hz, 1H), 7.46 - 7.37 (m, 2H), 5.90 (dt, J = 10.5, 5.4 Hz, 1H), 4.52 (dd, J = 13.3, 8.9 Hz, 1H), 3.02 - 2.62 (m, 4H), 2.34 - 1.83 (m, 3H).

LC/MS (APSI) m/z [M+H] calculated for C19H17ClNO3S: 374.1; found: 374.1.

3-methyl-2-(1-phenylethenyl)pyridin-4-amine - Z6829926113 (Method 6).

Yield: 30%; purity, >95% (assessed by LC/MS).

^{1}H NMR (600 MHz, DMSO-d_6) δ 7.87 (d, J = 5.4 Hz, 1H), 7.30 (dd, J = 8.1, 6.5 Hz, 2H), 7.28 - 7.21 (m, 3H), 6.50 (d, J = 5.4 Hz, 1H), 5.84 (d, J = 1.3 Hz, 1H), 5.77 (s, 2H), 5.13 (d, J = 1.3 Hz, 1H), 1.83 (s, 3H).

LC/MS (APSI) m/z [M+H] calculated for C14H15N2: 211.0; found: 211.1.

3-[1-(3-chlorophenyl)-3-[(thiophen-2-yl)methyl]-1H-1,2,4-triazol-5-yl]-4-methyl-1,2,5-oxadiazole - Z6969215903 (Method 9).

Yield: 11%; purity, >95% (assessed by LC/MS).

^{1}H NMR (500 MHz, DMSO-d_6) δ 7.76 (q, J = 1.3 Hz, 1H), 7.65 - 7.59 (m, 1H), 7.57 (s, 1H), 7.59 - 7.53 (m, 1H), 7.42 - 7.37 (m, 1H), 7.05 (d, J = 3.4 Hz, 1H), 6.96 (dd, J = 5.1, 3.4 Hz, 1H), 4.39 (s, 2H), 2.62 (s, 3H).

LC/MS (APSI) m/z [M+H] calculated for C16H13ClN5OS: 357.9; found: 358.0.

5-phenyl-3-[(2R)-pyrrolidin-2-yl]-1,2,4-oxadiazole - Z6971277399 (Method 5).

Yield: 16%; purity, 95% (assessed by LC/MS).

^{1}H NMR (600 MHz, DMSO-d_6) δ 8.12 - 8.07 (m, 2H), 7.73 - 7.67 (m, 1H), 7.63 (dd, J = 8.4, 7.0 Hz, 2H), 4.33 (dd, J = 8.0, 5.8 Hz, 1H), 2.97 (dt, J = 9.8, 6.6 Hz, 1H), 2.90 (ddd, J = 9.8, 7.6, 5.6 Hz, 1H), 2.16 - 2.06 (m, 1H), 1.97 - 1.82 (m, 2H), 1.80 - 1.70 (m, 1H).

LC/MS (APSI) m/z [M+H] calculated for C12H14N3O: 216.2; found: 216.1.

3-{3-[(2R)-pyrrolidin-2-yl]-1,2,4-oxadiazol-5-yl}pyridine - Z6971277405 (Method 5).

Yield: 11%; purity, >95% (assessed by LC/MS).

^{1}H NMR (500 MHz, DMSO-d_6) δ 9.36 - 8.69 (m, 3H), 8.52 - 8.07 (m, 2H), 7.78 - 7.22 (m, 2H), 4.71 - 4.13 (m, 1H), 3.06 - 2.78 (m, 1H), 2.18 - 1.55 (m, 3H).

LC/MS (APSI) m/z [M+H] calculated for C11H13N4O: 217.2; found: 217.1.

4-{3-[(2R)-pyrrolidin-2-yl]-1,2,4-oxadiazol-5-yl}pyridine - Z6971277409 (Method 5).

Yield: 13%; purity, >95% (assessed by LC/MS).

^{1}H NMR (600 MHz, DMSO-d_6) δ 8.92 - 8.85 (m, 2H), 8.05 - 7.99 (m, 2H), 4.38 (dd, J = 8.0, 5.8 Hz, 1H), 2.94 (tdd, J = 17.4, 10.0, 6.1 Hz, 2H), 2.14 (dtd, J = 16.4, 8.2, 5.5 Hz, 1H), 2.04 - 1.82 (m, 2H), 1.76 (tdd, J = 12.3, 8.0, 6.3 Hz, 1H).

LC/MS (APSI) m/z [M+H] calculated for C11H13N4O: 217.2; found: 217.1.

2-{3-[(2R)-pyrrolidin-2-yl]-1,2,4-oxadiazol-5-yl}pyridine - Z6971277415 (Method 5).

Yield: 23%; purity, >95% (assessed by LC/MS).

^{1}H NMR (500 MHz, Methanol-d_4) δ 8.81 (dt, J = 4.8, 1.4 Hz, 1H), 8.37 - 8.32 (m, 1H), 8.12 (td, J = 7.8, 1.7 Hz, 1H), 7.72 (ddd, J = 7.7, 4.8, 1.3 Hz, 1H), 5.05 (t, J = 7.8 Hz, 1H), 3.62 - 3.47 (m, 2H), 2.62 (dtd, J = 13.1, 7.9, 5.3 Hz, 1H), 2.41 (dq, J = 13.2, 7.9 Hz, 1H), 2.36 - 2.18 (m, 2H).

LC/MS (APSI) m/z [M+H] calculated for C11H13N4O: 217.2; found: 217.1.

5-(2-methylphenyl)-3-[(2R)-pyrrolidin-2-yl]-1,2,4-oxadiazole - Z6971278430 (Method 5).

Yield: 69%; purity, >95% (assessed by LC/MS).

^{1}H NMR (600 MHz, DMSO-d_6) δ 9.87 (s, 1H), 9.38 (s, 1H), 8.07 (dd, J = 7.9, 1.4 Hz, 1H), 7.62 (td, J = 7.6, 1.4 Hz, 1H), 7.52 (d, J = 7.7 Hz, 1H), 7.47 (t, J = 7.6 Hz, 1H), 5.03 (t, J = 7.7 Hz, 1H), 3.45 - 3.34 (m, 2H), 2.67 (s, 3H), 2.25 (dq, J = 12.8, 7.8 Hz, 1H), 2.20 - 2.03 (m, 2H).

LC/MS (APSI) m/z [M+H] calculated for C13H16N3O: 230.2; found: 230.1.

5-(4-methylphenyl)-3-[(2R)-pyrrolidin-2-yl]-1,2,4-oxadiazole - Z6971278455 (Method 5).

Yield: 13%; purity, >95% (assessed by LC/MS).

LC/MS (APSI) m/z [M+H] calculated for C13H16N3O: 230.0; found: 230.1.

5-(3-methylphenyl)-3-[(2R)-pyrrolidin-2-yl]-1,2,4-oxadiazole - Z6971278464 (Method 5).
Yield: 9%; purity, >95% (assessed by LC/MS).

^{1}H NMR (500 MHz, DMSO-*d*$_6$) δ 7.92 - 7.84 (m, 2H), 7.49 (d, J = 4.7 Hz, 2H), 4.30 (dd, J = 8.0, 5.6 Hz, 1H), 2.99 - 2.88 (m, 1H), 2.91 - 2.84 (m, 1H), 2.40 (s, 3H), 2.14 - 2.06 (m, 1H), 1.95 - 1.82 (m, 2H), 1.73 (s, 1H).

LC/MS (APSI) m/z [M+H] calculated for C13H16N3O: 230.0; found: 230.1.

5-(2-fluorophenyl)-3-[(2R)-pyrrolidin-2-yl]-1,2,4-oxadiazole - Z6971278779 (Method 5).
Yield: 24%; purity, >95% (assessed by LC/MS).

^{1}H NMR (500 MHz, DMSO-*d*$_6$) δ 8.19 - 7.93 (m, 1H), 7.74 (td, J = 8.0, 4.5 Hz, 1H), 7.45 (dtd, J = 28.3, 15.8, 13.5, 8.1 Hz, 3H), 7.11 - 6.75 (m, 1H), 4.34 (ddd, J = 12.1, 7.8, 5.5 Hz, 1H), 3.00 - 2.84 (m, 2H), 2.20 - 2.06 (m, 1H), 1.99 - 1.87 (m, 1H), 1.85 (td, J = 13.4, 12.7, 6.2 Hz, 1H), 1.74 (dp, J = 12.8, 6.8, 6.4 Hz, 1H).

LC/MS (APSI) m/z [M+H] calculated for C12H13FN3O: 234.0; found: 234.1.

5-(4-fluorophenyl)-3-[(2R)-pyrrolidin-2-yl]-1,2,4-oxadiazole - Z6971278787 (Method 5).
Yield: 10%; purity, >95% (assessed by LC/MS).

^{1}H NMR (500 MHz, DMSO-*d*$_6$) δ 8.14 (ddd, J = 8.6, 5.4, 2.6 Hz, 2H), 7.45 (t, J = 8.8 Hz, 2H), 4.30 (dd, J = 7.9, 5.6 Hz, 1H), 2.98 - 2.84 (m, 2H), 2.10 (dtd, J = 15.7, 8.1, 4.9 Hz, 1H), 1.95 - 1.79 (m, 2H), 1.78 - 1.67 (m, 1H).

LC/MS (APSI) m/z [M+H] calculated for C12H13FN3O: 234.0; found: 234.1.

5-(2-chlorophenyl)-3-[(2R)-pyrrolidin-2-yl]-1,2,4-oxadiazole - Z6971279383 (Method 5).
Yield: 12%; purity, >95% (assessed by LC/MS).

^{1}H NMR (500 MHz, DMSO-*d*$_6$) δ 8.05 (dd, J = 7.8, 1.7 Hz, 1H), 7.75 - 7.64 (m, 2H), 7.57 (td, J = 7.5, 1.4 Hz, 1H), 4.35 (dd, J = 8.0, 5.6 Hz, 1H), 2.98 - 2.85 (m, 2H), 2.11 (dtd, J = 11.2, 8.2, 5.4 Hz, 1H), 1.97 - 1.80 (m, 2H), 1.73 (dddd, J = 14.8, 12.2, 8.0, 6.0 Hz, 1H).

LC/MS (APSI) m/z [M+H] calculated for C12H13ClN3O: 250.2; found: 250.1.

5-(3-chlorophenyl)-3-[(2R)-pyrrolidin-2-yl]-1,2,4-oxadiazole - Z6971279394 (Method 5).
Yield: 19%; purity, >95% (assessed by LC/MS).

^{1}H NMR (500 MHz, DMSO-*d*$_6$) δ 8.10 - 8.01 (m, 2H), 7.82 - 7.74 (m, 1H), 7.65 (t, J = 7.9 Hz, 1H), 4.32 (dd, J = 8.0, 5.6 Hz, 1H), 2.98 - 2.85 (m, 2H), 2.09 (ddd, J = 12.7, 8.4, 4.7 Hz, 1H), 1.88 (dtt, J = 32.0, 13.7, 6.5 Hz, 2H), 1.73 (dt, J = 12.8, 7.0 Hz, 1H).

LC/MS (APSI) m/z [M+H] calculated for C12H13ClN3O: 250.0; found: 250.1.

5-(4-chlorophenyl)-3-[(2R)-pyrrolidin-2-yl]-1,2,4-oxadiazole - Z6971279399 (Method 5).
Yield: 11%; purity, >95% (assessed by LC/MS).

^{1}H NMR (500 MHz, DMSO-*d*$_6$) δ 8.12 - 8.06 (m, 2H), 7.71 - 7.66 (m, 2H), 4.31 (dd, J = 8.0, 5.6 Hz, 1H), 2.98 - 2.84 (m, 2H), 2.10 (dtd, J = 11.2, 8.0, 7.6, 5.0 Hz, 1H), 1.95 - 1.79 (m, 2H), 1.79 - 1.67 (m, 1H).

LC/MS (APSI) m/z [M+H] calculated for C12H13ClN3O: 250.0; found: 250.1.

5-(4-bromophenyl)-3-[(2R)-pyrrolidin-2-yl]-1,2,4-oxadiazole - Z6971279682 (Method 5).
Yield: 10%; purity, >95% (assessed by LC/MS).

LC/MS (APSI) m/z [M+H] calculated for C12H12BrN3O: 296.0; found: 296.0.

5-(2-bromophenyl)-3-[(2R)-pyrrolidin-2-yl]-1,2,4-oxadiazole - Z6971279689 (Method 5).
Yield: 8%; purity, 94% (assessed by LC/MS).

^{1}H NMR (600 MHz, DMSO-*d*$_6$) δ 8.00 (dd, J = 7.3, 2.2 Hz, 1H), 7.91 (dd, J = 7.4, 1.8 Hz, 1H), 7.66 - 7.58 (m, 3H), 7.50 (s, 0H), 4.44 (s, 1H), 3.08 - 2.92 (m, 2H), 2.16 (d, J = 6.1 Hz, 0H), 1.97 (dd, J = 13.8, 6.9 Hz, 1H), 1.93 - 1.88 (m, 1H), 1.80 (t, J = 9.2 Hz, 1H).

LC/MS (APSI) m/z [M+H] calculated for C12H12BrN3O: 296.1; found: 296.0.

5-(3-bromophenyl)-3-[(2R)-pyrrolidin-2-yl]-1,2,4-oxadiazole - Z6971279702 (Method 5).
Yield: 13%; purity, >95% (assessed by LC/MS).

^{1}H NMR (500 MHz, DMSO-*d*$_6$) δ 8.19 (t, J = 1.7 Hz, 1H), 8.08 (d, J = 8.0 Hz, 1H), 7.93 - 7.87 (m, 1H), 7.58 (t, J = 7.9 Hz, 1H), 4.32 (dd, J = 7.9, 5.6 Hz, 1H), 2.98 - 2.84 (m, 2H), 2.15 - 2.05 (m, 1H), 1.95 - 1.79 (m, 2H), 1.77 - 1.67 (m, 1H).

LC/MS (APSI) m/z [M+H] calculated for C12H12BrN3O: 294.0; found: 294.0.

(3S)-3-(4-methylphenyl)-3-({7H-pyrrolo[2,3-d]pyrimidin-4-yl}formamido)propanoic acid - Z7140729870 (Method 8).

Yield: 37%; purity, >95% (assessed by LC/MS).

^{1}H NMR (500 MHz, DMSO-d_6) δ 12.36 (s, 1H), 9.47 (d, J = 8.8 Hz, 1H), 8.84 (s, 1H), 7.70 (t, J = 2.8 Hz, 1H), 7.32 (d, J = 7.8 Hz, 2H), 7.10 (d, J = 7.7 Hz, 2H), 6.99 (d, J = 3.4 Hz, 1H), 5.46 - 5.38 (m, 1H), 3.01 (dd, J = 16.0, 7.8 Hz, 1H), 2.83 (dd, J = 16.0, 6.1 Hz, 1H), 2.24 (s, 3H).

LC/MS (APSI) m/z [M+H] calculated for C17H17N4O3: 325.2; found: 325.1.

N-[(1S)-1-(2,3-dihydro-1,4-benzodioxin-6-yl)-2-methylpropyl]-7H-pyrrolo[2,3-d]pyrimidin-4-amine - Z1039063794, Z7311094256 (Method 2).

Yield: 21%; purity, >95% (assessed by LC/MS).

^{1}H NMR (500 MHz, DMSO-d_6) δ 11.40 (s, 1H), 8.00 (s, 1H), 7.46 (d, J = 9.2 Hz, 1H), 7.02 (t, J = 2.9 Hz, 1H), 6.92 (d, J = 2.1 Hz, 1H), 6.83 (dd, J = 8.3, 2.1 Hz, 1H), 6.73 (d, J = 8.2 Hz, 1H), 6.65 (s, 1H), 4.93 (s, 1H), 4.16 (q, J = 4.1, 3.0 Hz, 4H), 2.09 - 2.01 (m, 1H), 0.98 (d, J = 6.5 Hz, 3H), 0.74 (d, J = 6.7 Hz, 3H).

LC/MS (APSI) m/z [M+H] calculated for C18H21N4O2: 325.2; found: 325.2.

(3S)-3-phenyl-3-({7H-pyrrolo[2,3-d]pyrimidin-4-yl}formamido)propanoic acid - Z7534253453 (Method 8).

Yield: 22%; purity, >95% (assessed by LC/MS).

^{1}H NMR (600 MHz, DMSO-d_6) δ 9.80 (d, J = 8.7 Hz, 1H), 8.86 (s, 1H), 7.71 (d, J = 3.4 Hz, 1H), 7.47 - 7.42 (m, 2H), 7.30 (t, J = 7.6 Hz, 2H), 7.22 (t, J = 7.4 Hz, 1H), 7.01 (d, J = 3.4 Hz, 1H), 5.42 (q, J = 7.3, 6.8 Hz, 1H), 2.94 (dd, J = 15.9, 7.2 Hz, 1H), 2.80 (dd, J = 15.9, 5.8 Hz, 1H).

LC/MS (APSI) m/z [M+H] calculated for C16H15N4O3: 311.2; found: 311.1.

3-(4-bromophenyl)-3-({2-methyl-7H-pyrrolo[2,3-d]pyrimidin-4-yl}formamido)propanoic acid - Z7691912366 (Method 8).

Yield: 23%; purity, >95% (assessed by LC/MS).

^{1}H NMR (500 MHz, DMSO-d_6) δ 12.35 (s, 1H), 12.08 (s, 1H), 9.37 (d, J = 8.8 Hz, 1H), 7.58 (t, J = 2.9 Hz, 1H), 7.52 (dd, J = 8.3, 2.0 Hz, 2H), 7.43 - 7.37 (m, 2H), 6.89 (dd, J = 3.6, 1.9 Hz, 1H), 5.43 (q, J = 7.5 Hz, 1H), 3.10 - 3.01 (m, 1H), 2.94 - 2.86 (m, 1H), 2.71 (d, J = 2.0 Hz, 3H).

LC/MS (APSI) m/z [M+H] calculated for C17H16BrN4O3: 403.0; found: 403.0.

3-(pyridin-4-yl)-3-({7H-pyrrolo[2,3-d]pyrimidin-4-yl}formamido)propanoic acid - Z7691912473 (Method 8).

Yield: 21%; purity, >95% (assessed by LC/MS).

^{1}H NMR (500 MHz, DMSO-d_6) δ 12.38 (s, 1H), 9.68 (d, J = 8.7 Hz, 1H), 8.87 (s, 1H), 8.51 - 8.46 (m, 2H), 7.71 (d, J = 3.6 Hz, 1H), 7.45 - 7.40 (m, 2H), 6.98 (d, J = 3.5 Hz, 1H), 5.43 (td, J = 8.2, 5.5 Hz, 1H), 3.06 (dd, J = 16.4, 7.9 Hz, 1H), 2.89 (dd, J = 16.4, 5.6 Hz, 1H).

LC/MS (APSI) m/z [M+H] calculated for C15H14N5O3: 312.2; found: 312.1.

(3S)-3-(4-chlorophenyl)-3-({7H-pyrrolo[2,3-d]pyrimidin-4-yl}formamido)propanoic acid - Z7692046343 (Method 8).

Yield: 39%; purity, >95% (assessed by LC/MS).

^{1}H NMR (600 MHz, DMSO-d_6) δ 12.37 (s, 1H), 9.58 (d, J = 8.7 Hz, 1H), 8.87 (s, 1H), 7.72 (t, J = 2.9 Hz, 1H), 7.51 - 7.46 (m, 2H), 7.41 - 7.36 (m, 2H), 7.00 (dd, J = 3.5, 1.4 Hz, 1H), 5.46 (td, J = 8.3, 6.1 Hz, 1H), 3.07 (dd, J = 16.2, 8.0 Hz, 1H), 2.89 (dd, J = 16.2, 6.1 Hz, 1H).

LC/MS (APSI) m/z [M+H] calculated for C16H14ClN4O3: 345.0; found: 345.1.

(3S)-3-(4-fluorophenyl)-3-({7H-pyrrolo[2,3-d]pyrimidin-4-yl}formamido)propanoic acid - Z7692056404 (Method 8).

Yield: 43%; purity, >95% (assessed by LC/MS).

^{1}H NMR (500 MHz, DMSO-d_6) δ 12.36 (s, 1H), 9.54 (d, J = 8.8 Hz, 1H), 8.85 (s, 1H), 7.73 - 7.68 (m, 1H), 7.52 - 7.46 (m, 2H), 7.13 (t, J = 8.8 Hz, 2H), 6.99 (dd, J = 3.5, 1.6 Hz, 1H), 5.46 (td, J = 8.3, 6.0 Hz, 1H), 3.05 (dd, J = 16.1, 8.0 Hz, 1H), 2.87 (dd, J = 16.1, 6.1 Hz, 1H).

LC/MS (APSI) m/z [M+H] calculated for C16H14FN4O3: 329.0; found: 329.1.

(3S)-3-(pyridin-2-yl)-3-({7H-pyrrolo[2,3-d]pyrimidin-4-yl}formamido)propanoic acid - Z7692056627 (Method 8).

Yield: 33%; purity, >95% (assessed by LC/MS).

^{1}H NMR (600 MHz, DMSO-d_6) δ 12.40 (s, 1H), 9.48 (d, J = 8.8 Hz, 1H), 8.88 (s, 1H), 8.56 (dd, J = 5.0, 1.7 Hz, 1H), 7.76 (ddd, J = 14.9, 7.5, 2.6 Hz, 2H), 7.44 (d, J = 7.9 Hz, 1H), 7.30 (dd, J = 7.5, 4.8 Hz, 1H), 7.04 (d, J = 3.4 Hz, 1H), 5.55 (dt, J = 8.7, 6.5 Hz, 1H), 3.05 (dd, J = 16.1, 6.9 Hz, 1H), 2.98 (dd, J = 16.1, 6.0 Hz, 1H).

LC/MS (APSI) m/z [M+H] calculated for C15H14N5O3: 312.0; found: 312.1.

[(1,4-Dioxa-5-aza-2,3-dihydro-7-naphthyl)methyl]-1,5,7-triaza-1H-inden-4-ylamine - Z9187344350, Z7983219665 (Method 2).

Yield: 11%; purity, >95% (assessed by LC/MS).

^{1}H NMR (600 MHz, DMSO-d_6) δ 11.50 (s, 1H), 8.09 (s, 1H), 7.85 (t, J = 6.0 Hz, 1H), 7.72 (d, J = 2.0 Hz, 1H), 7.25 (dd, J = 13.2, 2.1 Hz, 1H), 7.06 (dd, J = 3.4, 2.3 Hz, 1H), 6.52 (dd, J = 3.4, 1.9 Hz, 1H), 4.58 (d, J = 6.0 Hz, 2H), 4.38 - 4.31 (m, 3H), 4.26 - 4.16 (m, 3H), 0.89 (d, J = 6.7 Hz, 1H), 0.69 (d, J = 6.7 Hz, 1H).

LC/MS (APSI) m/z [M+H] calculated for C14H14N5O2: 284.1; found: 284.2.

[(S)-1-(1,4-Dioxa-6-aza-2,3-dihydro-7-naphthyl)-2-methylpropyl]-1,5,7-triaza-1H-inden-4-ylamine - Z9187344338, Z7983219668 (Method 2).

Yield: 16%; purity, >95% (assessed by LC/MS).

^{1}H NMR (500 MHz, DMSO-d_6) δ 11.43 (s, 1H), 8.02 (dd, J = 6.6, 1.7 Hz, 2H), 7.42 (d, J = 9.1 Hz, 1H), 7.03 (dt, J = 3.7, 2.0 Hz, 1H), 6.95 (d, J = 1.7 Hz, 1H), 6.72 (s, 1H), 5.09 (s, 1H), 4.29 (d, J = 4.5 Hz, 2H), 4.26 - 4.21 (m, 2H), 2.25 (s, 1H), 0.96 (dd, J = 6.7, 1.7 Hz, 3H), 0.75 (dd, J = 6.7, 1.6 Hz, 3H).

LC/MS (APSI) m/z [M+H] calculated for C17H20N5O2: 326.2; found: 326.2.

[(S)-1-(1,4-Dioxa-5-aza-2,3-dihydro-6-naphthyl)-2-methylpropyl]-1,5,7-triaza-1H-inden-4-ylamine - Z9187344358, Z7983219673 (Method 2).

Yield: 17%; purity, >95% (assessed by LC/MS).

^{1}H NMR (600 MHz, DMSO-d_6) δ 11.41 (s, 1H), 8.00 (s, 1H), 7.43 (d, J = 9.0 Hz, 1H), 7.18 (d, J = 7.9 Hz, 1H), 7.02 (dd, J = 3.4, 2.2 Hz, 1H), 6.95 (d, J = 8.0 Hz, 1H), 6.74 (s, 1H), 5.03 (s, 1H), 4.35 (q, J = 3.6 Hz, 2H), 4.22 - 4.15 (m, 2H), 2.24 (q, J = 7.4 Hz, 1H), 0.96 (d, J = 6.8 Hz, 3H), 0.75 (d, J = 6.7 Hz, 3H).

LC/MS (APSI) m/z [M+H] calculated for C17H20N5O2: 326.2; found: 326.2.

3-chloro-2-(1-phenylethenyl)pyridin-4-amine - Z8081978921 (Method 6).

Yield: 17%; purity, 93% (assessed by LC/MS).

LC/MS (APSI) m/z [M+H] calculated for C13H12ClN2: 231.0; found: 231.1.

3-{3-[(2R)-pyrrolidin-2-yl]-1,2,4-oxadiazol-5-yl}phenol - Z8172508405 (Method 5).

Yield: 12%; purity, >95% (assessed by LC/MS).

^{1}H NMR (600 MHz, DMSO-d_6) δ 10.04 (s, 1H), 7.51 (d, J = 7.6 Hz, 1H), 7.45 (t, J = 2.0 Hz, 1H), 7.42 (t, J = 7.9 Hz, 1H), 7.07 (dd, J = 8.1, 2.5 Hz, 1H), 4.33 (dd, J = 8.0, 5.8 Hz, 1H), 2.97 (dt, J = 9.9, 6.7 Hz, 1H), 2.91 (ddd, J = 9.9, 7.6, 5.5 Hz, 1H), 2.16 - 2.07 (m, 1H), 1.89 (dddd, J = 30.9, 15.7, 8.5, 4.4 Hz, 2H), 1.75 (dddd, J = 14.5, 12.0, 8.0, 6.2 Hz, 1H).

LC/MS (APSI) m/z [M+H] calculated for C12H14N3O2: 232.0; found: 232.1.

ethyl (1S,3aR,6aS)-2-[3-(trifluoromethyl)benzoyl]-octahydrocyclopenta[c]pyrrole-1-carboxylate - Z8184698918 (Method 1).

Yield: 65%; purity, >95% (assessed by LC/MS).

^{1}H NMR (500 MHz, DMSO-d_6) δ 7.86 (d, J = 7.9 Hz, 1H), 7.81 (d, J = 7.7 Hz, 1H), 7.76 - 7.65 (m, 2H), 4.32 (d, J = 3.2 Hz, 1H), 4.14 (p, J = 8.1, 6.8 Hz, 2H), 4.02 - 3.62 (m, 1H), 2.65 (s, 2H), 1.92 (dd, J = 13.3, 7.2 Hz, 1H), 1.72 (h, J = 6.7, 6.2 Hz, 2H), 1.64 (s, 1H), 1.58 - 1.51 (m, 2H), 1.37 - 1.32 (m, 1H), 1.21 (t, J = 7.1 Hz, 2H), 0.97 (t, J = 7.0 Hz, 1H).

LC/MS (APSI) m/z [M+H] calculated for C18H21F3NO3: 356.2; found: 356.1.

6-chloro-N-[1-(2,3-dihydro-1,4-benzodioxin-6-yl)-2-methylpropyl]-7H-pyrrolo[2,3-d]pyrimidin-4-amine - Z8207316857 (Method 2).

Yield: 14%; purity, >95% (assessed by LC/MS).

LC/MS (APSI) m/z [M+H] calculated for C18H20ClN4O2: 359.2; found: 359.1.

2-chloro-N-[1-(2,3-dihydro-1,4-benzodioxin-6-yl)-2-methylpropyl]-7H-pyrrolo[2,3-d]pyrimidin-4-amine - Z8207543137 (Method 2).

Yield: 27%; purity, >95% (assessed by LC/MS).

^{1}H NMR (600 MHz, DMSO-d_6) δ 11.61 (s, 1H), 7.96 (d, J = 9.0 Hz, 1H), 7.06 (dd, J = 3.5, 2.3 Hz, 1H), 6.92 (s, 1H), 6.84 (d, J = 8.5 Hz, 1H), 6.77 (d, J = 8.3 Hz, 1H), 6.68 (s, 1H), 4.79 (t, J = 9.3 Hz, 1H), 4.19 (dq, J = 6.1, 3.7, 2.6 Hz, 4H), 2.09 (s, 1H), 1.00 (d, J = 6.5 Hz, 3H), 0.75 (d, J = 6.7 Hz, 3H).

LC/MS (APSI) m/z [M+H] calculated for C18H20ClN4O2: 359.2; found: 359.1.

5-chloro-N-[1-(2,3-dihydro-1,4-benzodioxin-6-yl)-2-methylpropyl]-7H-pyrrolo[2,3-d]pyrimidin-4-amine - Z8207599372 (Method 2).

Yield: 24%; purity, >95% (assessed by LC/MS).

^{1}H NMR (500 MHz, DMSO-d_6) δ 11.90 (s, 1H), 8.07 (s, 1H), 7.31 (d, J = 2.9 Hz, 1H), 6.87 (d, J = 2.1 Hz, 1H), 6.81 (dd, J = 8.3, 2.1 Hz, 1H), 6.76 (d, J = 8.3 Hz, 1H), 6.31 (d, J = 8.8 Hz, 1H), 5.02 (t, J = 8.2 Hz, 1H), 4.18 (s, 4H), 2.18 (q, J = 6.8 Hz, 1H), 0.92 (d, J = 6.7 Hz, 3H), 0.82 (d, J = 6.7 Hz, 3H).

LC/MS (APSI) m/z [M+H] calculated for C18H20ClN4O2: 359.2; found: 359.1.

3-[2-(7-methyl-2,3,4,5-tetrahydro-1,5-benzothiazepin-5-yl)-2-oxoethyl]-1,3-dihydro-2-benzofuran-1-one - Z8383080706 (Method 1).

Yield: 48%; purity, >95% (assessed by LC/MS).

^{1}H NMR (500 MHz, DMSO-d_6) δ 7.79 - 7.60 (m, 2H), 7.54 (td, J = 7.5, 4.2 Hz, 1H), 7.45 (dd, J = 16.1, 7.9 Hz, 1H), 7.20 (dd, J = 35.4, 2.0 Hz, 1H), 7.07 (ddd, J = 7.9, 5.4, 1.9 Hz, 1H), 5.90 (dt, J = 17.7, 6.2 Hz, 1H), 4.53 (t, J = 13.4 Hz, 1H), 2.94 - 2.85 (m, 1H), 2.76 - 2.56 (m, 2H), 2.32 - 2.25 (m, 1H), 2.25 (d, J = 7.4 Hz, 3H), 2.14 - 2.07 (m, 1H), 2.05 (s, 1H), 2.01 (s, 1H), 1.96 (d, J = 15.9 Hz, 1H).

LC/MS (APSI) m/z [M+H] calculated for C20H20NO3S: 354.1; found: 354.1.

(3-Chloro-4-pyridyl)-1-naphthylamine - Z8825803705, Z8465161943 (Method 2).

Yield: 24%; purity, >95% (assessed by LC/MS).

^{1}H NMR (500 MHz, DMSO-d_6) δ 10.09 (s, 1H), 8.76 (s, 1H), 8.06 (t, J = 7.9 Hz, 3H), 7.77 (d, J = 8.2 Hz, 1H), 7.69 - 7.58 (m, 2H), 7.61 - 7.54 (m, 2H), 6.33 (dd, J = 6.8, 2.7 Hz, 1H).

LC/MS (APSI) m/z [M+H] calculated for C15Cl1H12N2: 255.1; found: 255.1.

5-(5-Methyl-1,2,5,6-tetrahydro-3-pyridyl)-1-benzothiophene - Z8501235596 (Method 3).

Yield: 24%; purity, >95% (assessed by LC/MS).

^{1}H NMR (600 MHz, DMSO-d_6) δ 7.92 (d, J = 8.4 Hz, 1H), 7.84 (d, J = 1.9 Hz, 1H), 7.73 (d, J = 5.4 Hz, 1H), 7.48 - 7.40 (m, 2H), 6.13 (d, J = 2.5 Hz, 1H), 3.59 (q, J = 2.3 Hz, 2H), 3.03 - 2.96 (m, 1H), 2.38 - 2.30 (m, 2H), 1.01 (d, J = 6.4 Hz, 3H).

LC/MS (APSI) m/z [M+H] calculated for C14H16N1S1: 230.1; found: 230.0.

1-{[3-(5-Phenyl-1,2,4-oxadiazol-3-yl)ureido]methyl}cyclobutanecarboxylic acid - Z8584748063 (Method 4).

Yield: 39%; purity, >95% (assessed by LC/MS).

^{1}H NMR (500 MHz, DMSO-d_6) δ 12.54 (s, 1H), 10.09 (s, 1H), 8.07 - 8.02 (m, 2H), 7.70 (t, J = 7.4 Hz, 1H), 7.62 (t, J = 7.7 Hz, 2H), 7.25 (t, J = 5.9 Hz, 1H), 3.53 (d, J = 5.4 Hz, 2H), 2.27 (dd, J = 11.1, 6.8 Hz, 2H), 1.99 - 1.82 (m, 4H).

LC/MS (APSI) m/z [M+H] calculated for C15H17N4O4: 317.1; found: 317.0.

1-({3-[5-(3-Pyridyl)-3-isoxazolyl]ureido}methyl)cyclobutanecarboxylic acid - Z8584748090 (Method 4).

Yield: 31%; purity, >95% (assessed by LC/MS).

^{1}H NMR (500 MHz, DMSO-d_6) δ 10.27 (s, 1H), 8.99 (d, J = 2.2 Hz, 1H), 8.65 (dd, J = 4.8, 1.7 Hz, 1H), 8.19 (dt, J = 8.0, 2.0 Hz, 1H), 7.50 (dd, J = 8.0, 4.8 Hz, 1H), 6.55 (d, J = 13.1 Hz, 2H), 3.49 (d, J = 5.8 Hz, 2H), 2.26 (dt, J = 11.3, 7.2 Hz, 2H), 1.88 (tdd, J = 21.0, 12.0, 6.9 Hz, 3H), 1.87 (s, 2H).

LC/MS (APSI) m/z [M+H] calculated for C15H17N4O4: 317.1; found: 317.2.

1-({3-[5-(4-Pyridyl)-3-isoxazolyl]ureido}methyl)cyclobutanecarboxylic acid - Z8584748099 (Method 4).

Yield: 55%; purity, >95% (assessed by LC/MS).

^{1}H NMR (500 MHz, DMSO-d_6) δ 10.47 (s, 1H), 8.70 - 8.65 (m, 2H), 7.80 - 7.75 (m, 2H), 6.71 (s, 1H), 6.56 (s, 1H), 3.48 (d, J = 5.7 Hz, 2H), 2.30 - 2.21 (m, 2H), 1.96 - 1.79 (m, 4H), 1.07 (t, J = 7.2 Hz, 1H).

LC/MS (APSI) m/z [M+H] calculated for C15H17N4O4: 317.1; found: 317.0.

1-{[3-(4-Methyl-5-phenyl-3-isoxazolyl)ureido]methyl}cyclobutanecarboxylic acid - Z8584748114 (Method 4).
Yield: 10%; purity, >95% (assessed by LC/MS).

^{1}H NMR (600 MHz, DMSO-d_6) δ 12.48 (s, 1H), 9.05 (s, 1H), 7.73 - 7.68 (m, 2H), 7.59 - 7.49 (m, 3H), 7.27 (d, J = 6.4 Hz, 1H), 3.55 (d, J = 5.8 Hz, 2H), 2.28 (ddd, J = 10.9, 7.7, 3.7 Hz, 2H), 2.11 (s, 3H), 1.95 (ddt, J = 20.7, 13.3, 6.4 Hz, 3H), 1.91 - 1.83 (m, 1H).

LC/MS (APSI) m/z [M+H] calculated for C17H20N3O4: 330.1; found: 330.2.

1-({3-[5-(p-Fluorophenyl)-3-isoxazolyl]ureido}methyl)cyclobutanecarboxylic acid - Z8584748244 (Method 4).
Yield: 29%; purity, >95% (assessed by LC/MS).

^{1}H NMR (600 MHz, DMSO-d_6) δ 12.47 (s, 1H), 9.52 (s, 1H), 7.94 - 7.88 (m, 2H), 7.36 (t, J = 8.8 Hz, 2H), 7.13 (s, 1H), 6.63 (t, J = 6.0 Hz, 1H), 3.51 (d, J = 5.8 Hz, 2H), 2.28 (tq, J = 10.2, 6.1, 4.5 Hz, 2H), 1.99 - 1.82 (m, 4H).

LC/MS (APSI) m/z [M+H] calculated for C16F1H17N3O4: 334.1; found: 334.0.

1-({3-[5-(o-Chlorophenyl)-3-isoxazolyl]ureido}methyl)cyclobutanecarboxylic acid - Z8584748322 (Method 4).
Yield: 20%; purity, >95% (assessed by LC/MS).

^{1}H NMR (600 MHz, DMSO-d_6) δ 12.49 (s, 1H), 9.60 (s, 1H), 7.89 (dd, J = 7.1, 2.3 Hz, 1H), 7.67 (dd, J = 7.3, 1.9 Hz, 1H), 7.58 - 7.49 (m, 2H), 7.29 (s, 1H), 6.64 (t, J = 6.0 Hz, 1H), 3.51 (d, J = 5.9 Hz, 2H), 2.28 (qd, J = 7.3, 6.8, 3.5 Hz, 2H), 1.99 - 1.82 (m, 4H).

LC/MS (APSI) m/z [M+H] calculated for C16Cl1H17N3O4: 350.1; found: 350.1.

1-({3-[5-(p-Chlorophenyl)-3-isoxazolyl]ureido}methyl)cyclobutanecarboxylic acid - Z8584748346 (Method 4).
Yield: 44%; purity, >95% (assessed by LC/MS).

^{1}H NMR (500 MHz, DMSO-d_6) δ 12.47 (s, 1H), 9.53 (s, 1H), 7.85 (d, J = 8.5 Hz, 2H), 7.56 (d, J = 8.5 Hz, 2H), 7.18 (s, 1H), 6.61 (t, J = 6.0 Hz, 1H), 3.49 (d, J = 5.8 Hz, 2H), 2.25 (dq, J = 9.4, 4.9, 2.9 Hz, 2H), 1.88 (dddd, J = 24.4, 18.2, 11.0, 5.9 Hz, 4H).

LC/MS (APSI) m/z [M+H] calculated for C16Cl1H17N3O4: 350.1; found: 350.1.

3-(p-Bromophenyl)-3-(6-purinylcarbonylamino)propionic acid - Z8598075280 (Method 8).
Yield: 43%; purity, >95% (assessed by LC/MS).

^{1}H NMR (500 MHz, DMSO-d_6) δ 9.80 (d, J = 8.5 Hz, 1H), 9.06 (s, 1H), 8.72 (d, J = 2.3 Hz, 1H), 7.54 - 7.48 (m, 2H), 7.45 - 7.39 (m, 2H), 5.45 (td, J = 8.2, 5.8 Hz, 1H), 3.08 (dd, J = 16.2, 8.2 Hz, 1H), 2.88 (dd, J = 16.2, 6.0 Hz, 1H).

LC/MS (APSI) m/z [M+H] calculated for C15Br1H13N5O3: 390.0; found: 390.0.

3-(p-Bromophenyl)-3-(3-methyl-1,5,7-triaza-1H-inden-4-ylcarbonylamino)propionic acid - Z8598075283 (Method 8).
Yield: 49%; purity, >95% (assessed by LC/MS).

^{1}H NMR (500 MHz, DMSO-d_6) δ 12.36 (s, 1H), 12.04 (s, 1H), 9.37 (d, J = 8.6 Hz, 1H), 8.74 (s, 1H), 7.53 (d, J = 8.4 Hz, 2H), 7.40 (td, J = 6.3, 5.7, 2.1 Hz, 3H), 5.42 (td, J = 8.4, 6.1 Hz, 1H), 2.94 (dd, J = 16.1, 8.2 Hz, 1H), 2.81 (dd, J = 16.1, 6.1 Hz, 1H), 2.24 (s, 3H).

LC/MS (APSI) m/z [M+H] calculated for C17Br1H16N4O3: 405.0; found: 405.0.

3-(4-Bromo-2-tolyl)-3-(1,5,7-triaza-1H-inden-4-ylcarbonylamino)propionic acid - Z8598075284 (Method 8).
Yield: 37%; purity, >95% (assessed by LC/MS).

^{1}H NMR (600 MHz, DMSO-d_6) δ 12.36 (s, 1H), 9.59 (d, J = 8.5 Hz, 1H), 8.86 (s, 1H), 7.71 (t, J = 2.8 Hz, 1H), 7.50 (d, J = 8.3 Hz, 1H), 7.40 - 7.34 (m, 2H), 6.98 (dd, J = 3.6, 1.5 Hz, 1H), 5.62 (td, J = 8.3, 5.9 Hz, 1H), 3.00 (dd, J = 16.1, 8.3 Hz, 1H), 2.81 (dd, J = 16.2, 6.0 Hz, 1H), 2.47 (s, 3H).

LC/MS (APSI) m/z [M+H] calculated for C17Br1H16N4O3: 403.0; found: 403.0.

3-(p-Bromophenyl)-3-(6-chloro-1,5,7-triaza-1H-inden-4-ylcarbonylamino)propionic acid - Z8598075292 (Method 8).
Yield: 42%; purity, >95% (assessed by LC/MS).

^{1}H NMR (500 MHz, DMSO-d_6) δ 12.57 (s, 1H), 9.43 (d, J = 8.6 Hz, 1H), 7.74 (d, J = 3.6 Hz, 1H), 7.54 - 7.49 (m, 2H), 7.40 (d, J = 8.0 Hz, 2H), 6.97 (d, J = 3.4 Hz, 1H), 5.42 (q, J = 7.5 Hz, 1H), 3.08 (dd, J = 16.3, 8.1 Hz, 1H), 2.88 (dd, J = 16.4, 6.0 Hz, 1H).

LC/MS (APSI) m/z [M+H] calculated for C16Br1Cl1H13N4O3: 425.0; found: 425.0.

3-(p-Hydroxyphenyl)-3-(1,5,7-triaza-1H-inden-4-ylcarbonylamino)propionic acid - Z8598075302 (Method 8).
Yield: 11%; purity, >95% (assessed by LC/MS).

^{1}H NMR (500 MHz, DMSO-*d*6) δ 12.34 (s, 1H), 9.40 (d, J = 8.9 Hz, 1H), 9.30 (s, 1H), 8.83 (s, 1H), 7.70 (d, J = 3.5 Hz, 1H), 7.23 (d, J = 8.1 Hz, 2H), 7.00 (d, J = 3.4 Hz, 1H), 6.67 (d, J = 8.1 Hz, 2H), 5.36 (q, J = 7.5 Hz, 1H), 2.97 (dd, J = 15.9, 7.7 Hz, 1H), 2.79 (dd, J = 15.9, 6.2 Hz, 1H).

LC/MS (APSI) m/z [M+H] calculated for C16H15N4O4: 327.0; found: 327.0.

N-4-Pyridyl-5-quinolylamine - Z8598084898 (Method 2).
Yield: 20%; purity, >95% (assessed by LC/MS).

^{1}H NMR (500 MHz, DMSO-*d*6) δ 9.04 (s, 1H), 8.92 (dd, J = 4.1, 1.6 Hz, 1H), 8.41 (d, J = 8.6 Hz, 1H), 8.16 (d, J = 5.6 Hz, 2H), 7.84 (d, J = 8.5 Hz, 1H), 7.78 - 7.68 (m, 1H), 7.54 (dd, J = 8.3, 3.8 Hz, 2H), 6.80 (t, J = 5.1 Hz, 2H).

LC/MS (APSI) m/z [M+H] calculated for C14H12N3: 222.1; found: 222.0.

(2-Methyl-4-pyridyl)-1-naphthylamine - Z8598084899 (Method 2).
Yield: 50%; purity, >95% (assessed by LC/MS).

^{1}H NMR (500 MHz, DMSO-*d*6) δ 8.73 (s, 1H), 8.01 - 7.97 (m, 2H), 7.97 - 7.91 (m, 1H), 7.74 (d, J = 8.1 Hz, 1H), 7.57 - 7.46 (m, 3H), 7.43 (d, J = 7.3 Hz, 1H), 6.59 (t, J = 2.1 Hz, 1H), 6.55 (dt, J = 5.6, 2.6 Hz, 1H), 2.47 (s, 0H), 2.25 (d, J = 3.0 Hz, 3H).

LC/MS (APSI) m/z [M+H] calculated for C16H15N2: 235.1; found: 235.0.

N-4-Pyridyl(3-methyl-1-naphthyl)amine - Z8598084900 (Method 2).
Yield: 45%; purity, >95% (assessed by LC/MS).

^{1}H NMR (500 MHz, DMSO-*d*6) δ 8.79 (s, 1H), 8.11 (d, J = 5.5 Hz, 2H), 7.91 (d, J = 8.4 Hz, 1H), 7.84 (d, J = 8.2 Hz, 1H), 7.53 (s, 1H), 7.51 - 7.45 (m, 1H), 7.45 - 7.39 (m, 1H), 7.29 (d, J = 1.7 Hz, 1H), 6.75 - 6.69 (m, 2H), 2.45 (s, 3H).

LC/MS (APSI) m/z [M+H] calculated for C16H15N2: 235.1; found: 235.0.

N-4-Pyridyl(4-methyl-1-naphthyl)amine - Z8598084901 (Method 2).
Yield: 31%; purity, 90% (assessed by LC/MS).

^{1}H NMR (600 MHz, DMSO-*d*6) δ 8.75 (s, 1H), 8.11 - 8.07 (m, 2H), 8.05 (d, J = 8.4 Hz, 1H), 8.01 - 7.93 (m, 1H), 7.60 (ddd, J = 8.2, 6.7, 1.3 Hz, 1H), 7.54 (ddd, J = 7.4, 6.1, 2.2 Hz, 1H), 7.38 (d, J = 7.5 Hz, 1H), 7.34 (d, J = 7.4 Hz, 1H), 6.67 - 6.62 (m, 2H), 2.66 (s, 3H).

LC/MS (APSI) m/z [M+H] calculated for C16H15N2: 235.1; found: 235.2.

N-4-Pyridyl(2-methyl-1-naphthyl)amine - Z8825803704, Z8598084904 (Method 2).
Yield: 15%; purity, >95% (assessed by LC/MS).

^{1}H NMR (600 MHz, DMSO-*d*6) δ 13.48 (s, 1H), 10.46 (s, 1H), 8.35 (s, 1H), 8.09 (s, 1H), 8.05 - 8.00 (m, 1H), 7.98 (d, J = 8.4 Hz, 1H), 7.77 - 7.73 (m, 1H), 7.61 - 7.53 (m, 3H), 7.34 (s, 1H), 6.01 (s, 1H), 2.33 (s, 3H).

LC/MS (APSI) m/z [M+H] calculated for C16H15N2: 235.1; found: 235.0.

N-4-Pyridyl(5-methyl-1-naphthyl)amine - Z8598084905 (Method 2).
Yield: 12%; purity, >95% (assessed by LC/MS).

^{1}H NMR (500 MHz, DMSO-*d*6) δ 8.84 (s, 1H), 8.12 - 8.08 (m, 2H), 7.86 (t, J = 8.4 Hz, 2H), 7.55 (t, J = 7.9 Hz, 1H), 7.46 (d, J = 7.3 Hz, 1H), 7.39 (q, J = 3.7 Hz, 2H), 6.69 (td, J = 4.8, 1.7 Hz, 2H), 2.66 (s, 3H).

LC/MS (APSI) m/z [M+H] calculated for C16H15N2: 235.1; found: 235.2.

N-4-Pyridyl(8-methyl-1-naphthyl)amine - Z8598084906 (Method 2).
Yield: 60%; purity, >95% (assessed by LC/MS).

^{1}H NMR (500 MHz, DMSO-*d*6) δ 8.56 (s, 1H), 8.06 - 8.01 (m, 2H), 7.86 (dd, J = 8.2, 1.4 Hz, 1H), 7.81 (d, J = 8.1 Hz, 1H), 7.50 (t, J = 7.7 Hz, 1H), 7.37 (t, J = 7.6 Hz, 2H), 7.25 (d, J = 7.0 Hz, 1H), 6.41 - 6.36 (m, 2H), 2.67 (s, 3H).

LC/MS (APSI) m/z [M+H] calculated for C16H15N2: 235.1; found: 235.2.

N-4-Pyridyl(6-fluoro-1-naphthyl)amine - Z8598084908 (Method 2).
Yield: 24%; purity, >95% (assessed by LC/MS).

^{1}H NMR (600 MHz, DMSO-d_6) δ 8.90 (s, 1H), 8.18 - 8.13 (m, 2H), 8.08 (dd, J = 9.3, 5.6 Hz, 1H), 7.79 - 7.73 (m, 2H), 7.56 (t, J = 7.8 Hz, 1H), 7.53 - 7.42 (m, 2H), 6.80 - 6.73 (m, 2H).

LC/MS (APSI) m/z [M+H] calculated for C15F1H12N2: 239.1; found: 239.2.

N-4-Pyridyl(7-fluoro-1-naphthyl)amine - Z8598084909 (Method 2).
Yield: 20%; purity, >95% (assessed by LC/MS).

^{1}H NMR (600 MHz, DMSO-d_6) δ 8.80 (s, 1H), 8.16 (d, J = 5.4 Hz, 2H), 8.08 (dd, J = 9.0, 5.9 Hz, 1H), 7.82 (dd, J = 7.3, 2.0 Hz, 1H), 7.70 (dd, J = 11.2, 2.6 Hz, 1H), 7.55 - 7.45 (m, 3H), 6.79 - 6.74 (m, 2H).

LC/MS (APSI) m/z [M+H] calculated for C15F1H12N2: 239.1; found: 239.2.

N-4-Pyridyl(8-fluoro-1-naphthyl)amine - Z8598084911 (Method 2).
Yield: 18%; purity, >95% (assessed by LC/MS).

^{1}H NMR (500 MHz, DMSO-d_6) δ 8.78 (s, 1H), 8.09 (d, J = 5.5 Hz, 2H), 7.84 (dd, J = 8.0, 2.2 Hz, 1H), 7.80 (d, J = 8.2 Hz, 1H), 7.57 (t, J = 7.8 Hz, 1H), 7.53 - 7.44 (m, 2H), 7.25 (dd, J = 13.8, 7.6 Hz, 1H), 6.72 - 6.63 (m, 2H).

LC/MS (APSI) m/z [M+H] calculated for C15F1H12N2: 239.1; found: 239.0.

N-4-Pyridyl(3-chloro-1-naphthyl)amine - Z8598084912 (Method 2).
Yield: 38%; purity, >95% (assessed by LC/MS).

^{1}H NMR (500 MHz, DMSO-d_6) δ 9.01 (s, 1H), 8.19 (d, J = 5.5 Hz, 2H), 8.02 (d, J = 8.4 Hz, 1H), 7.93 (d, J = 8.1 Hz, 1H), 7.84 (d, J = 2.1 Hz, 1H), 7.60 (t, J = 7.8 Hz, 1H), 7.58 - 7.51 (m, 1H), 7.41 (d, J = 2.1 Hz, 1H), 6.87 - 6.82 (m, 2H).

LC/MS (APSI) m/z [M+H] calculated for C15Cl1H12N2: 255.1; found: 255.0.

N-4-Pyridyl(4-chloro-1-naphthyl)amine - Z8598084913 (Method 2).
Yield: 29%; purity, >95% (assessed by LC/MS).

^{1}H NMR (500 MHz, DMSO-d_6) δ 8.93 (s, 1H), 8.20 (d, J = 8.4 Hz, 1H), 8.17 - 8.12 (m, 2H), 8.09 (d, J = 8.4 Hz, 1H), 7.73 (dd, J = 8.3, 6.9 Hz, 1H), 7.70 - 7.61 (m, 2H), 7.45 (d, J = 8.0 Hz, 1H), 6.80 - 6.73 (m, 2H).

LC/MS (APSI) m/z [M+H] calculated for C15Cl1H12N2: 255.1; found: 255.1.

N-4-Pyridyl(6-chloro-1-naphthyl)amine - Z8598084914 (Method 2).
Yield: 28%; purity, >95% (assessed by LC/MS).

^{1}H NMR (500 MHz, DMSO-d_6) δ 8.90 (s, 1H), 8.16 - 8.11 (m, 2H), 8.08 (d, J = 2.2 Hz, 1H), 8.01 (d, J = 9.0 Hz, 1H), 7.74 (d, J = 8.2 Hz, 1H), 7.59 - 7.50 (m, 2H), 7.47 (d, J = 7.3 Hz, 1H), 6.80 - 6.71 (m, 2H).

LC/MS (APSI) m/z [M+H] calculated for C15Cl1H12N2: 255.1; found: 255.0.

N-4-Pyridyl(8-chloro-1-naphthyl)amine - Z8598084918 (Method 2).
Yield: 44%; purity, >95% (assessed by LC/MS).

^{1}H NMR (500 MHz, DMSO-d_6) δ 8.61 (s, 1H), 8.05 (d, J = 5.5 Hz, 2H), 7.96 (dd, J = 16.1, 8.2 Hz, 2H), 7.64 - 7.56 (m, 2H), 7.52 - 7.44 (m, 2H), 6.45 (d, J = 5.5 Hz, 2H).

LC/MS (APSI) m/z [M+H] calculated for C15Cl1H12N2: 255.1; found: 255.0.

4-(4-Pyridylamino)-2-naphthol - Z8747421921, Z8598084919 (Method 2).
Yield: 12%; purity, >95% (assessed by LC/MS).

^{1}H NMR (500 MHz, DMSO-d_6) δ 10.15 (s, 1H), 8.77 (d, J = 6.1 Hz, 2H), 8.05 (d, J = 8.3 Hz, 1H), 8.01 (d, J = 6.1 Hz, 2H), 7.60 (d, J = 8.6 Hz, 1H), 7.36 (t, J = 7.7 Hz, 1H), 7.23 (t, J = 7.6 Hz, 1H), 6.51 (s, 1H).

LC/MS (APSI) m/z [M+H] calculated for C15H13N2O1: 237.1; found: 237.1.

8-(4-Pyridylamino)-2-naphthol - Z8598084922 (Method 2).
Yield: 35%; purity, >95% (assessed by LC/MS).

^{1}H NMR (600 MHz, DMSO-d_6) δ 9.78 (s, 1H), 8.66 (s, 1H), 8.11 - 8.07 (m, 2H), 7.82 (d, J = 8.8 Hz, 1H), 7.68 (d, J = 8.1 Hz, 1H), 7.33 (d, J = 7.2 Hz, 1H), 7.28 (t, J = 7.7 Hz, 1H), 7.19 (d, J = 2.4 Hz, 1H), 7.10 (dd, J = 8.8, 2.4 Hz, 1H), 6.63 - 6.59 (m, 2H).

LC/MS (APSI) m/z [M+H] calculated for C15H13N2O1: 237.1; found: 237.2.

5-(4-Pyridylamino)-1-naphthol - Z8701729806, Z8598084923 (Method 2).
Yield: 55%; purity, >95% (assessed by LC/MS).

^{1}H NMR (500 MHz, DMSO-d_6) δ 13.55 (s, 1H), 10.59 (s, 1H), 10.46 (s, 1H), 8.24 - 8.16 (m, 3H), 7.57 - 7.50 (m, 2H), 7.37 (t, J = 8.0 Hz, 1H), 7.27 (d, J = 8.5 Hz, 1H), 6.96 (d, J = 7.5 Hz, 1H), 6.66 (s, 1H).

LC/MS (APSI) m/z [M+H] calculated for C15H13N2O1: 237.1; found: 237.1.

8-(4-Pyridylamino)-1-naphthol - Z8731865364, Z8598084924 (Method 2).
Yield: 13%; purity, >95% (assessed by LC/MS).

^{1}H NMR (600 MHz, DMSO-d_6) δ 8.20 (d, J = 5.5 Hz, 2H), 7.49 (d, J = 8.0 Hz, 1H), 7.39 (t, J = 7.8 Hz, 1H), 7.36 - 7.31 (m, 2H), 7.28 (t, J = 7.8 Hz, 1H), 7.01 (d, J = 5.8 Hz, 2H), 6.83 (d, J = 7.5 Hz, 1H).

LC/MS (APSI) m/z [M+H] calculated for C15H13N2O1: 237.1; found: 237.0.

4-(1-Naphthylamino)-3-pyridinol - Z8598084925 (Method 2).
Yield: 11%; purity, >95% (assessed by LC/MS).

^{1}H NMR (500 MHz, DMSO-d_6) δ 8.25 (s, 1H), 7.96 (d, J = 8.0 Hz, 1H), 7.92 - 7.83 (m, 2H), 7.80 (d, J = 8.2 Hz, 1H), 7.64 (d, J = 5.5 Hz, 1H), 7.60 (s, 1H), 7.52 (dtd, J = 14.9, 8.1, 7.5, 5.0 Hz, 3H), 7.41 (d, J = 7.2 Hz, 1H), 6.29 (d, J = 5.6 Hz, 1H).

LC/MS (APSI) m/z [M+H] calculated for C15H13N2O1: 237.1; found: 237.2.

5-Methyl-2-(1-phenylethenyl)-4-pyridylamine - Z8598084957 (Method 6).
Yield: 18%; purity, 93% (assessed by LC/MS).

^{1}H NMR (500 MHz, DMSO-d_6) δ 7.89 (s, 1H), 7.33 (ddd, J = 13.9, 7.6, 5.9 Hz, 3H), 7.30 - 7.25 (m, 2H), 6.42 (s, 1H), 5.79 - 5.72 (m, 3H), 5.35 (d, J = 2.0 Hz, 1H), 1.99 (s, 3H).

LC/MS (APSI) m/z [M+H] calculated for C14H15N2: 211.1; found: 211.2.

5-Chloro-2-(1-phenylethenyl)-4-pyridylamine - Z8598084962 (Method 6).
Yield: 25%; purity, >95% (assessed by LC/MS).

^{1}H NMR (600 MHz, DMSO-d_6) δ 8.14 (s, 1H), 7.41 - 7.28 (m, 6H), 6.61 (s, 1H), 6.32 (s, 2H), 5.86 (d, J = 1.8 Hz, 1H), 5.45 (d, J = 1.8 Hz, 1H).

LC/MS (APSI) m/z [M+H] calculated for C13Cl1H12N2: 231.1; found: 231.1.

5-(7-Chloro-5-indanyl)-1,2,3,6-tetrahydropyridine - Z8646465742 (Method 3).
Yield: 32%; purity, >95% (assessed by LC/MS).

^{1}H NMR (500 MHz, DMSO-d_6) δ 8.70 (dd, J = 155.4, 3.4 Hz, 1H), 7.55 (s, 1H), 7.23 - 7.07 (m, 2H), 6.22 (s, 1H), 3.59 (s, 2H), 2.18 (s, 2H), 2.15 - 1.90 (m, 7H).

LC/MS (APSI) m/z [M+H] calculated for C14Cl1H17N1: 234.1; found: 234.1.

2-Methyl-5-(1,2,5,6-tetrahydro-3-pyridyl)-1-benzothiophene - Z8647047828 (Method 3).
Yield: 28%; purity, 93% (assessed by LC/MS).

^{1}H NMR (500 MHz, DMSO-d_6) δ 7.75 (d, J = 8.4 Hz, 1H), 7.64 (s, 1H), 7.29 (d, J = 8.4 Hz, 1H), 7.07 (s, 1H), 6.22 (d, J = 4.4 Hz, 1H), 3.57 (d, J = 2.9 Hz, 2H), 2.80 (t, J = 5.7 Hz, 2H), 2.53 (s, 3H), 2.14 (tt, J = 5.9, 3.0 Hz, 2H).

LC/MS (APSI) m/z [M+H] calculated for C14H16N1S1: 230.1; found: 230.1.

2-Methyl-5-(4-methyl-1,2,5,6-tetrahydro-3-pyridyl)-1-benzothiophene - Z8647047832 (Method 3).
Yield: 23%; purity, >95% (assessed by LC/MS).

^{1}H NMR (500 MHz, DMSO-d_6) δ 8.58 - 7.85 (m, 1H), 7.77 (d, J = 8.2 Hz, 1H), 7.45 (s, 1H), 7.15 - 6.97 (m, 2H), 2.86 (t, J = 5.8 Hz, 2H), 2.63 - 2.56 (m, 1H), 2.53 (s, 3H), 2.13 - 1.97 (m, 2H), 1.54 (s, 4H).

LC/MS (APSI) m/z [M+H] calculated for C15H18N1S1: 244.1; found: 244.1.

Ethyl (1S,2S,5R)-3-{[4-(trifluoromethyl)-2-pyridyl]carbonyl}-3-azabicyclo[3.3.0]octane-2-carboxylate - Z8701681644 (Method 1).
Yield: 54%; purity, >95% (assessed by LC/MS).

^{1}H NMR (500 MHz, DMSO-d_6) δ 8.85 (dd, J = 69.1, 5.1 Hz, 1H), 8.04 (d, J = 43.0 Hz, 1H), 7.91 (ddd, J = 18.4, 5.3, 2.0 Hz, 1H), 4.56 (dd, J = 213.2, 3.4 Hz, 1H), 4.17 - 3.94 (m, 2H), 3.86 - 3.54 (m, 2H), 3.28 (s, 2H), 2.68 (dddt, J = 35.5, 15.9, 8.2, 3.6 Hz, 2H), 2.04 - 1.33 (m, 4H), 1.14 (dtd, J = 58.8, 7.1, 3.0 Hz, 3H).

LC/MS (APSI) m/z [M+H] calculated for C17F3H20N2O3: 357.1; found: 357.2.

Ethyl (1S,2S,5R)-3-[5-(trifluoromethyl)-nicotinoyl]-3-azabicyclo[3.3.0]octane-2-carboxylate - Z8701681645 (Method 1).

Yield: 56%; purity, >95% (assessed by LC/MS).

^{1}H NMR (500 MHz, DMSO-d_6) δ 9.07 (d, J = 22.1 Hz, 1H), 9.03 - 8.84 (m, 1H), 8.23 (d, J = 45.8 Hz, 1H), 4.45 - 4.21 (m, 1H), 4.18 - 3.46 (m, 3H), 3.28 (s, 1H), 2.80 - 2.59 (m, 2H), 2.02 - 1.28 (m, 6H), 1.08 (dt, J = 125.1, 7.0 Hz, 3H).

LC/MS (APSI) m/z [M+H] calculated for C17F3H20N2O3: 357.1; found: 357.2.

Ethyl (1S,2S,5R)-3-[2-(trifluoromethyl)-isonicotinoyl]-3-azabicyclo[3.3.0]octane-2-carboxylate - Z8701681646 (Method 1).

Yield: 68%; purity, >95% (assessed by LC/MS).

^{1}H NMR (500 MHz, DMSO-d_6) δ 8.86 (dd, J = 22.8, 4.9 Hz, 1H), 7.97 - 7.62 (m, 2H), 4.42 - 3.81 (m, 3H), 3.79 - 3.44 (m, 1H), 3.28 (s, 1H), 3.24 (dd, J = 10.7, 3.5 Hz, 1H), 2.84 - 2.58 (m, 3H), 2.07 - 1.30 (m, 4H), 1.09 (dt, J = 115.1, 7.1 Hz, 3H).

LC/MS (APSI) m/z [M+H] calculated for C17F3H20N2O3: 357.1; found: 357.0.

Ethyl (1S,2S,5R)-3-{[6-(trifluoromethyl)-2-pyridyl]carbonyl}-3-azabicyclo[3.3.0]octane-2-carboxylate - Z8701681647 (Method 1).

Yield: 62%; purity, >95% (assessed by LC/MS).

^{1}H NMR (500 MHz, DMSO-d_6) δ 8.23 (q, J = 7.9 Hz, 1H), 8.19 - 7.91 (m, 2H), 4.58 (dd, J = 246.5, 3.4 Hz, 1H), 4.24 - 3.83 (m, 2H), 3.83 - 3.52 (m, 2H), 3.28 (s, 1H), 2.81 - 2.56 (m, 2H), 2.09 - 1.29 (m, 4H), 1.13 (dt, J = 66.8, 7.1 Hz, 3H).

LC/MS (APSI) m/z [M+H] calculated for C17F3H20N2O3: 357.1; found: 357.2.

Ethyl (1S,2S,5R)-3-[5-(trifluoromethyl)-2-toluoyl]-3-azabicyclo[3.3.0]octane-2-carboxylate - Z8701681648 (Method 1).

Yield: 58%; purity, >95% (assessed by LC/MS).

^{1}H NMR (500 MHz, DMSO-d_6) δ 7.78 - 7.23 (m, 3H), 4.40 - 3.37 (m, 4H), 2.90 (dd, J = 10.8, 3.3 Hz, 1H), 2.64 (pq, J = 6.7, 3.9 Hz, 2H), 2.30 (d, J = 33.3 Hz, 3H), 2.09 - 1.27 (m, 6H), 1.27 - 0.91 (m, 3H).

LC/MS (APSI) m/z [M+H] calculated for C19F3H23N1O3: 370.2; found: 370.2.

Ethyl (1S,2S,5R)-3-[3-(trifluoromethyl)-2-toluoyl]-3-azabicyclo[3.3.0]octane-2-carboxylate - Z8701681649 (Method 1).

Yield: 54%; purity, >95% (assessed by LC/MS).

^{1}H NMR (500 MHz, DMSO-d_6) δ 7.93 - 7.66 (m, 1H), 7.48 (d, J = 5.3 Hz, 2H), 4.45 - 3.39 (m, 4H), 3.28 (s, 1H), 2.99 - 2.54 (m, 3H), 2.32 (d, J = 48.2 Hz, 3H), 2.01 - 1.27 (m, 5H), 1.21 (td, J = 7.2, 6.4, 2.8 Hz, 3H).

LC/MS (APSI) m/z [M+H] calculated for C19F3H23N1O3: 370.2; found: 370.2.

Ethyl (1S,2S,5R)-3-[5-(trifluoromethyl)-3-toluoyl]-3-azabicyclo[3.3.0]octane-2-carboxylate - Z8184698918, Z8701681656 (Method 1).

Yield: 56%; purity, >95% (assessed by LC/MS).

^{1}H NMR (500 MHz, DMSO-d_6) δ 7.75 - 7.26 (m, 3H), 4.42 - 3.42 (m, 4H), 3.28 (s, 1H), 3.24 (dd, J = 10.7, 3.3 Hz, 1H), 2.80 - 2.56 (m, 2H), 2.41 (d, J = 19.2 Hz, 3H), 2.04 - 1.26 (m, 4H), 1.10 (dt, J = 103.0, 7.0 Hz, 3H).

LC/MS (APSI) m/z [M+H] calculated for C19F3H23N1O3: 370.2; found: 370.2.

Ethyl (1S,2S,5R)-3-[3-(trifluoromethyl)-4-toluoyl]-3-azabicyclo[3.3.0]octane-2-carboxylate - Z8701681660 (Method 1).

Yield: 66%; purity, 93% (assessed by LC/MS).

^{1}H NMR (500 MHz, DMSO-d_6) δ 7.81 - 7.33 (m, 3H), 4.51 - 3.46 (m, 4H), 3.27 (d, J = 11.2 Hz, 2H), 2.63 (d, J = 15.4 Hz, 2H), 2.45 (s, 2H), 2.05 - 1.26 (m, 4H), 1.25 - 0.91 (m, 3H).

LC/MS (APSI) m/z [M+H] calculated for C19F3H23N1O3: 370.2; found: 370.2.

Ethyl (1S,2S,5R)-3-[2-hydroxy-5-(trifluoromethyl)benzoyl]-3-azabicyclo[3.3.0]octane-2-carboxylate - Z8701681662 (Method 1).

Yield: 45%; purity, >95% (assessed by LC/MS).

^{1}H NMR (500 MHz, DMSO-d_6) δ 10.93 (s, 1H), 7.58 (ddd, J = 18.3, 8.7, 2.6 Hz, 1H), 7.28 (dd, J = 59.2, 2.6 Hz, 1H), 7.03 (dd, J = 17.1, 8.6 Hz, 1H), 4.46 - 3.38 (m, 4H), 3.12 (dd, J = 10.9, 2.7 Hz, 2H), 2.75 - 2.56 (m, 2H), 2.05 - 1.31 (m, 6H), 1.08 (dtd, J = 124.2, 7.1, 2.9 Hz, 3H).

LC/MS (APSI) m/z [M+H] calculated for C18F3H21N1O4: 372.1; found: 372.2.

Ethyl (1S,2S,5R)-3-[4-hydroxy-3-(trifluoromethyl)benzoyl]-3-azabicyclo[3.3.0]octane-2-carboxylate - Z8701681669 (Method 1).

Yield: 44%; purity, >95% (assessed by LC/MS).

^{1}H NMR (500 MHz, DMSO-d_6) δ 10.41 (s, 1H), 7.36 - 6.83 (m, 3H), 4.40 - 3.43 (m, 5H), 3.28 (s, 1H), 2.91 (dd, J = 10.9, 3.0 Hz, 1H), 2.80 - 2.53 (m, 2H), 2.02 - 1.25 (m, 4H), 1.22 - 1.02 (m, 3H).

LC/MS (APSI) m/z [M+H] calculated for C18F3H21N1O4: 372.1; found: 372.2.

Ethyl (1S,2S,5R)-3-[3-hydroxy-5-(trifluoromethyl)benzoyl]-3-azabicyclo[3.3.0]octane-2-carboxylate - Z8701681670 (Method 1).

Yield: 54%; purity, >95% (assessed by LC/MS).

^{1}H NMR (500 MHz, DMSO-d_6) δ 10.47 (s, 1H), 7.27 - 6.74 (m, 3H), 4.43 - 3.46 (m, 4H), 3.23 (dd, J = 10.7, 3.1 Hz, 1H), 2.63 (d, J = 12.3 Hz, 2H), 2.05 - 1.25 (m, 6H), 1.11 (dt, J = 88.8, 7.1 Hz, 3H).

LC/MS (APSI) m/z [M+H] calculated for C18F3H21N1O4: 372.1; found: 372.2.

Ethyl (1S,2S,5R)-3-[2-hydroxy-3-(trifluoromethyl)benzoyl]-3-azabicyclo[3.3.0]octane-2-carboxylate - Z8701681673 (Method 1).

Yield: 18%; purity, 90% (assessed by LC/MS).

^{1}H NMR (500 MHz, DMSO-d_6) δ 10.72 (d, J = 209.8 Hz, 1H), 7.89 - 6.82 (m, 3H), 4.46 - 3.55 (m, 4H), 2.73 - 2.62 (m, 1H), 2.02 - 1.32 (m, 7H), 1.29 - 0.81 (m, 3H).

LC/MS (APSI) m/z [M+H] calculated for C18F3H21N1O4: 372.1; found: 372.2.

Ethyl (1S,2S,5R)-3-[4-fluoro-3-(trifluoromethyl)benzoyl]-3-azabicyclo[3.3.0]octane-2-carboxylate - Z8701681675 (Method 1).

Yield: 52%; purity, >95% (assessed by LC/MS).

^{1}H NMR (500 MHz, DMSO-d_6) δ 8.06 - 7.36 (m, 3H), 4.46 - 3.49 (m, 4H), 3.28 (s, 1H), 2.78 - 2.59 (m, 2H), 2.05 - 1.29 (m, 6H), 1.27 - 0.85 (m, 3H).

LC/MS (APSI) m/z [M+H] calculated for C18F4H20N1O3: 374.1; found: 374.0.

Ethyl (1S,2S,5R)-3-[2-fluoro-3-(trifluoromethyl)benzoyl]-3-azabicyclo[3.3.0]octane-2-carboxylate - Z8701681677 (Method 1).

Yield: 71%; purity, >95% (assessed by LC/MS).

^{1}H NMR (500 MHz, DMSO-d_6) δ 7.88 (dt, J = 15.8, 7.3 Hz, 1H), 7.71 (dt, J = 34.2, 7.1 Hz, 1H), 7.48 (dt, J = 23.6, 7.8 Hz, 1H), 4.46 - 3.40 (m, 4H), 3.28 (s, 1H), 3.07 (dd, J = 10.8, 2.8 Hz, 1H), 2.68 (qt, J = 10.7, 5.6 Hz, 2H), 2.03 - 1.25 (m, 4H), 1.11 (dt, J = 101.1, 7.1 Hz, 3H).

LC/MS (APSI) m/z [M+H] calculated for C18F4H20N1O3: 374.1; found: 374.2.

Ethyl (1S,2S,5R)-3-[2-fluoro-5-(trifluoromethyl)benzoyl]-3-azabicyclo[3.3.0]octane-2-carboxylate - Z8701681679 (Method 1).

Yield: 75%; purity, >95% (assessed by LC/MS).

^{1}H NMR (500 MHz, DMSO-d_6) δ 7.93 (td, J = 9.1, 8.6, 4.6 Hz, 1H), 7.82 - 7.62 (m, 1H), 7.57 (dt, J = 16.9, 8.9 Hz, 1H), 4.43 - 3.44 (m, 4H), 3.09 (dd, J = 10.7, 2.8 Hz, 1H), 2.85 - 2.56 (m, 1H), 2.02 - 1.26 (m, 6H), 1.10 (dt, J = 104.7, 7.1 Hz, 3H).

LC/MS (APSI) m/z [M+H] calculated for C18F4H20N1O3: 374.1; found: 374.2.

Ethyl (1S,2S,5R)-3-[3-fluoro-5-(trifluoromethyl)benzoyl]-3-azabicyclo[3.3.0]octane-2-carboxylate - Z8701681686 (Method 1).

Yield: 57%; purity, >95% (assessed by LC/MS).

^{1}H NMR (500 MHz, DMSO-d_6) δ 7.96 - 7.39 (m, 3H), 4.44 - 3.46 (m, 4H), 3.27 (d, J = 12.7 Hz, 1H), 2.76 - 2.57 (m, 2H), 2.02 - 1.29 (m, 6H), 1.27 - 0.84 (m, 3H).

LC/MS (APSI) m/z [M+H] calculated for C18F4H20N1O3: 374.1; found: 374.0.

Ethyl (1S,2S,5R)-3-[2-chloro-5-(trifluoromethyl)benzoyl]-3-azabicyclo[3.3.0]octane-2-carboxylate - Z8701681688 (Method 1).

Yield: 49%; purity, >95% (assessed by LC/MS).

^{1}H NMR (500 MHz, DMSO-d_6) δ 7.96 - 7.35 (m, 3H), 4.45 - 3.43 (m, 5H), 2.94 (dd, J = 11.1, 2.8 Hz, 1H), 2.79 - 2.57 (m, 2H), 2.02 - 1.31 (m, 5H), 1.12 (dt, J = 97.5, 7.1 Hz, 3H).

LC/MS (APSI) m/z [M+H] calculated for C18Cl1F3H20N1O3: 390.1; found: 390.2.

Ethyl (1S,2S,5R)-3-[3-chloro-5-(trifluoromethyl)benzoyl]-3-azabicyclo[3.3.0]octane-2-carboxylate - Z8701681695 (Method 1).

Yield: 51%; purity, >95% (assessed by LC/MS).

^{1}H NMR (500 MHz, DMSO-d_6) δ 8.19 - 7.53 (m, 3H), 4.47 - 3.42 (m, 4H), 3.28 - 3.23 (m, 2H), 2.80 - 2.56 (m, 2H), 2.07 - 1.28 (m, 5H), 1.10 (dt, J = 102.8, 7.1 Hz, 3H).

LC/MS (APSI) m/z [M+H] calculated for C18Cl1F3H20N1O3: 390.1; found: 390.0.

Ethyl (1S,2S,5R)-3-[4-chloro-3-(trifluoromethyl)benzoyl]-3-azabicyclo[3.3.0]octane-2-carboxylate - Z8701681696 (Method 1).

Yield: 66%; purity, >95% (assessed by LC/MS).

^{1}H NMR (500 MHz, DMSO-d_6) δ 7.97 - 7.59 (m, 3H), 4.43 - 3.42 (m, 4H), 2.64 (d, J = 17.9 Hz, 2H), 2.01 - 1.27 (m, 7H), 1.27 - 0.91 (m, 3H).

LC/MS (APSI) m/z [M+H] calculated for C18Cl1F3H20N1O3: 390.1; found: 390.0.

Ethyl (1S,2S,5R)-3-[2-chloro-3-(trifluoromethyl)benzoyl]-3-azabicyclo[3.3.0]octane-2-carboxylate - Z8701681697 (Method 1).

Yield: 49%; purity, >95% (assessed by LC/MS).

^{1}H NMR (500 MHz, DMSO-d_6) δ 8.12 - 7.27 (m, 3H), 4.42 - 3.43 (m, 4H), 3.09 - 2.57 (m, 3H), 2.04 - 1.29 (m, 6H), 1.28 - 0.91 (m, 3H).

LC/MS (APSI) m/z [M+H] calculated for C18Cl1F3H20N1O3: 390.1; found: 390.0.

1-(3-Chloro-2-tolyl)-5-(4-methyl-3-furazanyl)-3-thenyl-1H-1,2,4-triazole - Z8701681958 (Method 9).

Yield: 6%; purity, >95% (assessed by LC/MS).

^{1}H NMR (500 MHz, DMSO-d_6) δ 7.69 (d, J = 8.0 Hz, 1H), 7.47 - 7.42 (m, 1H), 7.42 - 7.35 (m, 2H), 7.04 (d, J = 3.5 Hz, 1H), 6.97 (dd, J = 5.2, 3.4 Hz, 1H), 4.41 (s, 2H), 2.69 - 2.61 (m, 3H), 1.99 (s, 3H).

LC/MS (APSI) m/z [M+H] calculated for C17Cl1H15N5O1S1: 372.1; found: 372.0.

1-(5-Chloro-2-tolyl)-5-(4-methyl-3-furazanyl)-3-thenyl-1H-1,2,4-triazole - Z8701681960 (Method 9).

Yield: 8%; purity, >95% (assessed by LC/MS).

^{1}H NMR (500 MHz, Methanol-d_4) δ 7.50 (dd, J = 8.3, 2.3 Hz, 1H), 7.46 - 7.38 (m, 2H), 7.26 (dd, J = 5.2, 1.3 Hz, 1H), 7.04 - 6.99 (m, 1H), 6.95 (dd, J = 5.2, 3.5 Hz, 1H), 4.43 - 4.39 (m, 2H), 2.68 (s, 3H), 2.00 (s, 3H).

LC/MS (APSI) m/z [M+H] calculated for C17Cl1H15N5O1S1: 372.1; found: 372.0.

1-(3-Chloro-4-tolyl)-5-(4-methyl-3-furazanyl)-3-thenyl-1H-1,2,4-triazole - Z8701681963 (Method 9).

Yield: 9%; purity, >95% (assessed by LC/MS).

^{1}H NMR (600 MHz, DMSO-d_6) δ 7.73 (d, J = 2.1 Hz, 1H), 7.53 (d, J = 8.2 Hz, 1H), 7.48 (dd, J = 8.1, 2.1 Hz, 1H), 7.41 (dd, J = 5.2, 1.3 Hz, 1H), 7.06 (dd, J = 3.3, 1.3 Hz, 1H), 6.98 (dd, J = 5.1, 3.4 Hz, 1H), 4.40 (s, 2H), 2.63 (s, 3H), 2.41 (s, 3H).

LC/MS (APSI) m/z [M+H] calculated for C17Cl1H15N5O1S1: 372.1; found: 372.0.

1-(3-Chloro-5-fluorophenyl)-5-(4-methyl-3-furazanyl)-3-thenyl-1H-1,2,4-triazole - Z8701681979 (Method 9).

Yield: 8%; purity, 93% (assessed by LC/MS).

LC/MS (APSI) m/z [M+H] calculated for C16Cl1F1H12N5O1S1: 376.0; found: 376.0.

1-(3,4-Dichlorophenyl)-5-(4-methyl-3-furazanyl)-3-thenyl-1H-1,2,4-triazole - Z8701681983 (Method 9).

Yield: 12%; purity, >95% (assessed by LC/MS).

^{1}H NMR (600 MHz, DMSO-d_6) δ 8.03 (d, J = 2.4 Hz, 1H), 7.85 (d, J = 8.6 Hz, 1H), 7.65 (dd, J = 8.6, 2.5 Hz, 1H), 7.41 (dd, J = 5.1, 1.3 Hz, 1H), 7.07 (dd, J = 3.3, 1.3 Hz, 1H), 6.98 (dd, J = 5.1, 3.5 Hz, 1H), 4.41 (s, 2H), 2.65 (s, 3H).

LC/MS (APSI) m/z [M+H] calculated for C16Cl2H12N5O1S1: 392.0; found: 392.0.

1-(3,5-Dichlorophenyl)-5-(4-methyl-3-furazanyl)-3-thenyl-1H-1,2,4-triazole - Z8701681984 (Method 9).
Yield: 8%; purity, >95% (assessed by LC/MS).

^{1}H NMR (600 MHz, DMSO-d_6) δ 7.87 (t, J = 1.9 Hz, 1H), 7.82 (d, J = 1.9 Hz, 2H), 7.41 (dd, J = 5.1, 1.3 Hz, 1H), 7.07 (dd, J = 3.5, 1.3 Hz, 1H), 6.98 (dd, J = 5.1, 3.4 Hz, 1H), 4.41 (s, 2H), 2.66 (s, 3H).

LC/MS (APSI) m/z [M+H] calculated for C16Cl2H12N5O1S1: 392.0; found: 392.0.

3-(m-Tolyl)-3-(1,5,7-triaza-1H-inden-4-ylcarbonylamino)propionic acid - Z8727396582 (Method 8).
Yield: 29%; purity, >95% (assessed by LC/MS).

^{1}H NMR (500 MHz, DMSO-d_6) δ 12.35 (s, 1H), 9.49 (d, J = 8.9 Hz, 1H), 8.85 (s, 1H), 7.70 (d, J = 3.5 Hz, 1H), 7.24 (d, J = 9.2 Hz, 2H), 7.18 (t, J = 7.5 Hz, 1H), 7.03 (d, J = 7.4 Hz, 1H), 6.99 (d, J = 3.4 Hz, 1H), 5.42 (td, J = 8.2, 5.8 Hz, 1H), 3.02 (dd, J = 16.0, 7.8 Hz, 1H), 2.83 (dd, J = 16.0, 6.0 Hz, 1H), 2.26 (s, 3H).

LC/MS (APSI) m/z [M+H] calculated for C17H17N4O3: 325.1; found: 325.0.

2-Methyl-3-phenyl-3-(1,5,7-triaza-1H-inden-4-ylcarbonylamino)propionic acid - Z8727396638 (Method 8).
Yield: 55%; purity, >95% (assessed by LC/MS).

^{1}H NMR (500 MHz, DMSO-d_6) δ 12.37 (s, 1H), 9.59 (d, J = 9.3 Hz, 1H), 8.87 (s, 1H), 7.71 (t, J = 2.5 Hz, 1H), 7.44 (d, J = 7.6 Hz, 2H), 7.31 (t, J = 7.5 Hz, 2H), 7.23 (t, J = 7.3 Hz, 1H), 6.98 (d, J = 3.5 Hz, 1H), 5.22 - 5.15 (m, 1H), 3.14 (p, J = 7.1 Hz, 1H), 1.04 (d, J = 7.1 Hz, 3H).

LC/MS (APSI) m/z [M+H] calculated for C17H17N4O3: 325.1; found: 325.2.

(S)-3-(m-Chlorophenyl)-3-(1,5,7-triaza-1H-inden-4-ylcarbonylamino)propionic acid - Z8727401304 (Method 8).
Yield: 39%; purity, >95% (assessed by LC/MS).

^{1}H NMR (500 MHz, DMSO-d_6) δ 12.37 (s, 1H), 9.60 (d, J = 8.8 Hz, 1H), 8.86 (s, 1H), 7.74 - 7.69 (m, 1H), 7.53 (t, J = 2.0 Hz, 1H), 7.41 (d, J = 7.6 Hz, 1H), 7.34 (t, J = 7.8 Hz, 1H), 7.29 (dt, J = 7.9, 1.5 Hz, 1H), 6.99 (dd, J = 3.5, 1.6 Hz, 1H), 5.45 (td, J = 8.3, 5.9 Hz, 1H), 3.07 (dd, J = 16.2, 8.1 Hz, 1H), 2.89 (dd, J = 16.2, 6.0 Hz, 1H).

LC/MS (APSI) m/z [M+H] calculated for C16Cl1H14N4O3: 345.1; found: 345.0.

4-(4-Pyridylamino)-1-naphthol - Z8755839162 (Method 2).
Yield: 27%; purity, >95% (assessed by LC/MS).

^{1}H NMR (600 MHz, DMSO-d_6) δ 13.53 (s, 1H), 10.62 (s, 1H), 10.52 (s, 1H), 8.24 (dd, J = 7.3, 2.1 Hz, 1H), 8.18 (s, 2H), 7.75 - 7.70 (m, 1H), 7.55 (ddt, J = 10.7, 6.8, 3.5 Hz, 2H), 7.35 (d, J = 7.9 Hz, 1H), 6.98 (d, J = 8.0 Hz, 1H).

LC/MS (APSI) m/z [M+H] calculated for C15H13N2O1: 237.1; found: 237.2.

[1-(2,3-Dihydro-1,4-benzodioxin-6-yl)-2-methylpropyl](3-methyl-1,5,7-triaza-1H-inden-4-yl)amine - Z8756044539 (Method 2).
Yield: 13%; purity, >95% (assessed by LC/MS).

^{1}H NMR (600 MHz, DMSO-d_6) δ 11.18 (s, 1H), 7.98 (s, 1H), 6.96 (d, J = 2.0 Hz, 1H), 6.87 (dd, J = 8.3, 2.1 Hz, 1H), 6.83 (t, J = 1.8 Hz, 1H), 6.75 (d, J = 8.3 Hz, 1H), 5.97 (d, J = 8.9 Hz, 1H), 4.97 (t, J = 8.6 Hz, 1H), 4.19 (q, J = 2.6 Hz, 4H), 2.45 (d, J = 1.1 Hz, 3H), 2.26 - 2.17 (m, 1H), 0.97 (d, J = 6.6 Hz, 3H), 0.79 (d, J = 6.7 Hz, 3H).

LC/MS (APSI) m/z [M+H] calculated for C19H23N4O2: 339.2; found: 339.2.

[1-(2,3-Dihydro-1,4-benzodioxin-6-yl)-2-methylpropyl](2-methyl-1,5,7-triaza-1H-inden-4-yl)amine - Z8756044544 (Method 2).
Yield: 23%; purity, >95% (assessed by LC/MS).

^{1}H NMR (600 MHz, DMSO-d_6) δ 11.24 (s, 1H), 7.94 (s, 1H), 7.24 (d, J = 9.3 Hz, 1H), 6.92 (d, J = 2.0 Hz, 1H), 6.83 (dd, J = 8.3, 2.0 Hz, 1H), 6.73 (d, J = 8.2 Hz, 1H), 6.33 (s, 1H), 4.91 (s, 1H), 4.18 (dp, J = 9.3, 2.8 Hz, 4H), 2.29 (s, 3H), 2.09 - 2.00 (m, 1H), 0.98 (d, J = 6.5 Hz, 3H), 0.74 (d, J = 6.6 Hz, 3H).

LC/MS (APSI) m/z [M+H] calculated for C19H23N4O2: 339.2; found: 339.2.

4-[1-(2,3-Dihydro-1,4-benzodioxin-6-yl)-2-methylpropylamino]-1,5,7-triaza-1H-inden-2-ol - Z9043825450, Z8756044564 (Method 2).
Yield: 13%; purity, >95% (assessed by LC/MS).

^{1}H NMR (600 MHz, DMSO-d_6) δ 10.82 (s, 1H), 8.18 (d, J = 86.7 Hz, 1H), 7.14 (dd, J = 51.2, 13.6 Hz, 1H), 6.93 - 6.64 (m, 3H), 4.76 (s, 1H), 4.19 (h, J = 4.6 Hz, 6H), 2.00 (d, J = 8.1 Hz, 1H), 1.12 - 0.88 (m, 3H), 0.85 - 0.61 (m, 3H).

LC/MS (APSI) m/z [M+H] calculated for C18H21N4O3: 341.2; found: 341.2.

p-[3-(2-Azetidinyl)-1,2,4-oxadiazol-5-yl]phenol - Z8766147193 (Method 5).

Yield: 17%; purity, >95% (assessed by LC/MS).

^{1}H NMR (600 MHz, DMSO-d_6) δ 10.74 (s, 1H), 9.70 (s, 2H), 8.03 - 7.96 (m, 2H), 7.04 - 7.00 (m, 2H), 5.70 (t, J = 8.4 Hz, 1H), 4.12 (q, J = 9.2 Hz, 1H), 3.98 (td, J = 9.9, 6.0 Hz, 1H), 2.93 - 2.85 (m, 1H), 2.83 (ddd, J = 12.0, 7.3, 2.9 Hz, 1H).

LC/MS (APSI) m/z [M+H] calculated for C11H12N3O2: 218.1; found: 218.2.

(S)-3-(o-Chlorophenyl)-3-(1,5,7-triaza-1H-inden-4-ylcarbonylamino)propionic acid - Z8768700676 (Method 8).

Yield: 35%; purity, >95% (assessed by LC/MS).

^{1}H NMR (500 MHz, DMSO-d_6) δ 12.37 (s, 1H), 9.67 (d, J = 8.5 Hz, 1H), 8.88 (s, 1H), 7.71 (t, J = 2.8 Hz, 1H), 7.59 (dd, J = 7.6, 2.0 Hz, 1H), 7.43 (dd, J = 7.6, 1.6 Hz, 1H), 7.28 (dtd, J = 17.2, 7.4, 1.7 Hz, 2H), 6.99 - 6.94 (m, 1H), 5.82 (td, J = 8.9, 4.3 Hz, 1H), 3.02 (dd, J = 16.3, 9.2 Hz, 1H), 2.75 (dd, J = 16.3, 4.3 Hz, 1H).

LC/MS (APSI) m/z [M+H] calculated for C16Cl1H14N4O3: 345.1; found: 345.2.

Cyclobutyl(5-{3-[2-(dimethylamino)-2-(3-thienyl)ethyl]ureido}-2-methyl-1-piperidyl)methanone - Z8771520648 (Method 1).

Yield: 13%; purity, >95% (assessed by LC/MS).

^{1}H NMR (600 MHz, DMSO-d_6) δ 7.49 (s, 1H), 7.31 (d, J = 12.8 Hz, 1H), 7.16 - 6.91 (m, 1H), 6.22 (dd, J = 141.4, 9.4 Hz, 1H), 5.98 - 5.51 (m, 1H), 4.67 (s, 1H), 3.85 - 3.49 (m, 2H), 3.17 (dd, J = 77.7, 11.2 Hz, 1H), 2.22 (t, J = 12.0 Hz, 1H), 2.05 (d, J = 6.3 Hz, 6H), 1.89 - 1.31 (m, 4H), 1.23 - 0.90 (m, 3H).

LC/MS (APSI) m/z [M+H] calculated for C20H33N4O2S1: 393.2; found: 393.4.

Cyclobutyl(3-{3-[2-(dimethylamino)-2-(3-thienyl)ethyl]ureido}-2-methyl-1-piperidyl)methanone - Z8771520652 (Method 1).

Yield: 13%; purity, >95% (assessed by LC/MS).

^{1}H NMR (500 MHz, DMSO-d_6) δ 7.83 (s, 1H), 7.72 (s, 1H), 7.29 (s, 1H), 4.53 (s, 1H), 3.51 (s, 3H), 2.80 - 2.52 (m, 8H), 2.31 - 1.27 (m, 3H), 1.20 - 0.75 (m, 1H).

LC/MS (APSI) m/z [M+H] calculated for C20H33N4O2S1: 393.2; found: 393.2.

(3-{3-[2-(Dimethylamino)-2-(3-thienyl)ethyl]ureido}-1-piperidyl)(2-methylcyclobutyl)methanone - Z8771520655 (Method 1).

Yield: 62%; purity, >95% (assessed by LC/MS).

^{1}H NMR (500 MHz, DMSO-d_6) δ 7.50 - 7.45 (m, 1H), 7.30 (s, 1H), 7.02 (d, J = 4.5 Hz, 1H), 6.36 - 5.94 (m, 1H), 5.70 (d, J = 33.2 Hz, 1H), 4.38 - 3.76 (m, 1H), 3.71 - 3.35 (m, 7H), 3.00 (d, J = 40.5 Hz, 1H), 2.88 - 2.59 (m, 1H), 2.04 (s, 7H), 1.88 (s, 1H), 1.74 (s, 1H), 1.57 (s, 1H), 1.30 (s, 3H), 1.13 - 0.75 (m, 4H).

LC/MS (APSI) m/z [M+H] calculated for C20H33N4O2S1: 393.2; found: 393.2.

(3-{3-[2-(Dimethylamino)-2-(3-thienyl)ethyl]ureido}-1-piperidyl)(3-methylcyclobutyl)methanone - Z8771520657 (Method 1).

Yield: 58%; purity, >95% (assessed by LC/MS).

^{1}H NMR (500 MHz, DMSO-d_6) δ 7.48 (dd, J = 5.0, 2.9 Hz, 1H), 7.29 (d, J = 3.2 Hz, 1H), 7.02 (d, J = 5.0 Hz, 1H), 6.31 - 5.99 (m, 1H), 5.76 - 5.64 (m, 1H), 3.56 (dq, J = 13.4, 7.1, 6.5 Hz, 1H), 3.47 - 3.35 (m, 2H), 3.09 - 2.66 (m, 2H), 2.04 (s, 11H), 1.84 - 1.43 (m, 4H), 1.44 - 1.14 (m, 3H), 1.01 (ddd, J = 66.4, 6.6, 2.2 Hz, 3H).

LC/MS (APSI) m/z [M+H] calculated for C20H33N4O2S1: 393.2; found: 393.0.

(3-{3-[2-(Dimethylamino)-2-(3-thienyl)ethyl]ureido}-1-piperidyl)(1-methylcyclobutyl)methanone - Z8771520658 (Method 1).

Yield: 58%; purity, >95% (assessed by LC/MS).

^{1}H NMR (500 MHz, DMSO-d_6) δ 7.47 (dd, J = 4.9, 2.9 Hz, 1H), 7.29 (d, J = 3.0 Hz, 1H), 7.02 (d, J = 4.9 Hz, 1H), 6.15 (d, J = 60.5 Hz, 1H), 5.70 (s, 1H), 3.88 (d, J = 116.6 Hz, 1H), 3.56 (s, 1H), 3.51 (d, J = 14.5 Hz, 1H), 3.38 (d, J

= 15.3 Hz, 2H), 2.89 (s, 1H), 2.67 (s, 1H), 2.50 (s, 1H), 2.36 (dd, J = 18.0, 8.9 Hz, 2H), 2.03 (s, 6H), 1.88 (d, J = 8.2 Hz, 1H), 1.73 (s, 1H), 1.58 (s, 3H), 1.32 (d, J = 8.3 Hz, 5H).

LC/MS (APSI) m/z [M+H] calculated for C20H33N4O2S1: 393.2; found: 393.2.

Cyclobutyl(3-{3-[2-(dimethylamino)-2-(3-thienyl)ethyl]ureido}-5-hydroxy-1-piperidyl)methanone - Z8771520665 (Method 1).

Yield: 15%; purity, >95% (assessed by LC/MS).

^{1}H NMR (600 MHz, DMSO-d_6) δ 7.49 (dd, J = 5.0, 3.0 Hz, 1H), 7.31 (q, J = 2.9, 2.2 Hz, 1H), 7.09 - 6.98 (m, 1H), 6.41 - 6.06 (m, 1H), 5.87 - 5.63 (m, 1H), 5.20 - 4.82 (m, 1H), 4.30 - 4.10 (m, 1H), 3.83 - 3.38 (m, 2H), 3.29 - 3.13 (m, 2H), 2.67 - 2.53 (m, 1H), 2.43 - 2.32 (m, 1H), 2.28 - 1.61 (m, 11H), 1.38 - 0.98 (m, 1H).

LC/MS (APSI) m/z [M+H] calculated for C19H31N4O3S1: 395.2; found: 395.2.

(3-{3-[2-(Dimethylamino)-2-(3-thienyl)ethyl]ureido}-1-piperidyl)(1-hydroxycyclobutyl)methanone - Z8771520670 (Method 1).

Yield: 51%; purity, >95% (assessed by LC/MS).

^{1}H NMR (500 MHz, DMSO-d_6) δ 7.47 (dd, J = 5.0, 2.9 Hz, 1H), 7.29 (s, 1H), 7.02 (d, J = 5.2 Hz, 1H), 6.11 (dd, J = 32.9, 7.9 Hz, 1H), 5.81 (d, J = 26.9 Hz, 1H), 5.68 - 5.63 (m, 1H), 4.26 - 3.70 (m, 2H), 3.57 (s, 1H), 3.38 (ddd, J = 12.5, 7.9, 4.0 Hz, 1H), 3.28 - 3.12 (m, 2H), 3.06 - 2.76 (m, 1H), 2.58 - 2.49 (m, 1H), 2.03 (s, 6H), 2.04 - 1.91 (m, 2H), 1.85 - 1.13 (m, 5H).

LC/MS (APSI) m/z [M+H] calculated for C19H31N4O3S1: 395.2; found: 395.1.

(3-{3-[2-(Dimethylamino)-2-(3-thienyl)ethyl]ureido}-1-piperidyl)(3-fluorocyclobutyl)methanone - Z8771520680 (Method 1).

Yield: 56%; purity, 93% (assessed by LC/MS).

^{1}H NMR (500 MHz, DMSO-d_6) δ 7.47 (dd, J = 5.0, 2.7 Hz, 1H), 7.31 - 7.27 (m, 1H), 7.02 (d, J = 4.9 Hz, 1H), 6.33 - 6.00 (m, 1H), 5.70 (d, J = 34.9 Hz, 1H), 5.28 - 4.81 (m, 1H), 4.10 - 3.49 (m, 2H), 3.45 - 3.36 (m, 4H), 3.28 - 3.23 (m, 1H), 2.97 (dd, J = 12.8, 6.4 Hz, 1H), 2.78 - 2.55 (m, 1H), 2.46 - 2.16 (m, 1H), 2.03 (s, 6H), 1.72 (d, J = 11.3 Hz, 1H), 1.56 (s, 1H), 1.31 (d, J = 47.7 Hz, 3H).

LC/MS (APSI) m/z [M+H] calculated for C19F1H30N4O2S1: 397.2; found: 397.2.

(3-{3-[2-(Dimethylamino)-2-(3-thienyl)ethyl]ureido}-1-piperidyl)(2-fluorocyclobutyl)methanone - Z8771520687 (Method 1).

Yield: 31%; purity, >95% (assessed by LC/MS).

^{1}H NMR (500 MHz, DMSO-d_6) δ 7.47 (dt, J = 5.0, 2.6 Hz, 1H), 7.29 (d, J = 3.0 Hz, 1H), 7.13 - 6.96 (m, 1H), 6.40 - 6.02 (m, 1H), 5.70 (d, J = 39.4 Hz, 1H), 5.06 (ddd, J = 54.9, 13.4, 6.9 Hz, 1H), 3.95 (dd, J = 27.7, 12.7 Hz, 1H), 3.76 - 3.35 (m, 6H), 3.18 - 2.58 (m, 1H), 2.28 - 1.06 (m, 15H).

LC/MS (APSI) m/z [M+H] calculated for C19F1H30N4O2S1: 397.2; found: 397.2.

(3-{3-[2-(Dimethylamino)-2-(3-thienyl)ethyl]ureido}-1-piperidyl)(1-fluorocyclobutyl)methanone - Z8771520688 (Method 1).

Yield: 55%; purity, >95% (assessed by LC/MS).

^{1}H NMR (500 MHz, DMSO-d_6) δ 7.47 (dd, J = 5.0, 2.9 Hz, 1H), 7.29 (d, J = 4.0 Hz, 1H), 7.02 (d, J = 4.6 Hz, 1H), 6.31 - 6.04 (m, 1H), 5.82 - 5.58 (m, 1H), 4.16 - 3.34 (m, 4H), 3.17 - 2.54 (m, 4H), 2.40 - 1.11 (m, 15H).

LC/MS (APSI) m/z [M+H] calculated for C19F1H30N4O2S1: 397.2; found: 397.0.

(3-{3-[2-(Dimethylamino)-2-(3-thienyl)ethyl]ureido}-1-piperidyl)[(1r,3r)-3-hydroxycyclobutyl]methanone - Z8774357908 (Method 1).

Yield: 40%; purity, >95% (assessed by LC/MS).

^{1}H NMR (500 MHz, DMSO-d_6) δ 7.48 (dd, J = 5.0, 2.8 Hz, 1H), 7.29 (d, J = 3.0 Hz, 1H), 7.02 (d, J = 4.9 Hz, 1H), 6.36 - 6.02 (m, 1H), 5.85 - 5.57 (m, 1H), 5.02 (s, 1H), 4.23 - 3.87 (m, 1H), 3.66 - 3.33 (m, 4H), 3.17 - 2.56 (m, 3H), 2.41 - 0.92 (m, 15H).

LC/MS (APSI) m/z [M+H] calculated for C19H31N4O3S1: 395.2; found: 395.2.

5-(4-Methyl-1,2,5,6-tetrahydro-3-pyridyl)-1-thia-4-azaindene - Z8781161134 (Method 3).

Yield: 18%; purity, >95% (assessed by LC/MS).

^{1}H NMR (600 MHz, DMSO-d_6) δ 8.44 - 8.38 (m, 1H), 8.12 (d, J = 5.5 Hz, 1H), 7.51 (d, J = 5.5 Hz, 1H), 7.25 (d, J = 8.4 Hz, 1H), 3.57 (q, J = 2.3 Hz, 2H), 2.92 (t, J = 5.8 Hz, 2H), 2.11 (d, J = 6.2 Hz, 2H), 1.69 (s, 3H).

LC/MS (APSI) m/z [M+H] calculated for C13H15N2S1: 231.1; found: 231.2.

Cyclobutyl(3-{3-[2-(dimethylamino)-2-(3-thienyl)ethyl]ureido}-3-methyl-1-piperidyl)methanone - Z8793812850 (Method 1).

Yield: 17%; purity, >95% (assessed by LC/MS).

^{1}H NMR (600 MHz, DMSO-d_6) δ 9.94 - 9.22 (m, 1H), 7.94 - 7.78 (m, 1H), 7.73 (dt, J = 5.0, 2.4 Hz, 1H), 7.31 (ddd, J = 19.0, 8.8, 5.0 Hz, 1H), 6.34 - 5.92 (m, 1H), 5.92 - 5.67 (m, 1H), 4.72 - 3.59 (m, 4H), 3.14 - 3.02 (m, 1H), 2.90 - 2.56 (m, 7H), 2.32 - 1.36 (m, 11H), 1.34 - 1.17 (m, 3H).

LC/MS (APSI) m/z [M+H] calculated for C20H33N4O2S1: 393.2; found: 393.2.

Cyclobutyl(3-{3-[2-(dimethylamino)-2-(3-thienyl)ethyl]ureido}-4-methyl-1-piperidyl)methanone - Z8793812851 (Method 1).

Yield: 30%; purity, >95% (assessed by LC/MS).

^{1}H NMR (500 MHz, DMSO-d_6) δ 9.64 (d, J = 135.2 Hz, 1H), 8.11 - 7.77 (m, 1H), 7.72 (dt, J = 5.7, 2.9 Hz, 1H), 7.40 - 7.06 (m, 1H), 6.43 - 5.70 (m, 2H), 4.89 - 3.98 (m, 2H), 3.36 - 2.71 (m, 2H), 2.65 (dt, J = 73.1, 4.7 Hz, 6H), 2.20 - 0.98 (m, 9H), 0.91 - 0.50 (m, 3H).

LC/MS (APSI) m/z [M+H] calculated for C20H33N4O2S1: 393.2; found: 393.2.

Cyclobutyl(3-{3-[2-(dimethylamino)-2-(3-thienyl)ethyl]ureido}-5-methyl-1-piperidyl)methanone - Z8798881729 (Method 1).

Yield: 13%; purity, >95% (assessed by LC/MS).

^{1}H NMR (600 MHz, DMSO-d_6) δ 9.65 (d, J = 114.4 Hz, 1H), 8.00 - 7.79 (m, 1H), 7.73 (dt, J = 5.5, 2.9 Hz, 1H), 7.31 (tt, J = 10.2, 4.7 Hz, 1H), 6.35 - 5.87 (m, 2H), 4.90 - 3.60 (m, 4H), 3.14 - 2.57 (m, 10H), 2.39 - 0.63 (m, 13H).

LC/MS (APSI) m/z [M+H] calculated for C20H33N4O2S1: 393.2; found: 393.2.

(3-Chlorocyclobutyl)(3-{3-[2-(dimethylamino)-2-(3-thienyl)ethyl]ureido}-1-piperidyl)methanone - Z8812737822 (Method 1).

Yield: 43%; purity, >95% (assessed by LC/MS).

^{1}H NMR (500 MHz, DMSO-d_6) δ 9.75 (d, J = 40.5 Hz, 1H), 7.84 (d, J = 8.3 Hz, 1H), 7.77 - 7.52 (m, 1H), 7.29 (d, J = 5.3 Hz, 1H), 6.44 - 6.15 (m, 1H), 6.18 - 5.83 (m, 1H), 4.51 (d, J = 40.4 Hz, 2H), 4.08 - 3.66 (m, 1H), 3.49 (ddt, J = 53.5, 8.6, 4.7 Hz, 2H), 3.17 - 2.56 (m, 11H), 1.90 - 1.10 (m, 5H).

LC/MS (APSI) m/z [M+H] calculated for C19Cl1H30N4O2S1: 413.2; found: 413.1.

(1-Chlorocyclobutyl)(3-{3-[2-(dimethylamino)-2-(3-thienyl)ethyl]ureido}-1-piperidyl)methanone - Z8812742460 (Method 1).

Yield: 62%; purity, >95% (assessed by LC/MS).

^{1}H NMR (500 MHz, DMSO-d_6) δ 9.75 (s, 1H), 7.97 - 7.80 (m, 1H), 7.70 (dd, J = 5.1, 2.8 Hz, 1H), 7.29 (dd, J = 10.4, 5.0 Hz, 1H), 6.28 (ddd, J = 29.2, 13.6, 7.8 Hz, 1H), 6.17 - 5.75 (m, 1H), 4.56 (s, 1H), 4.22 - 3.60 (m, 3H), 3.17 - 2.52 (m, 11H), 2.43 - 1.24 (m, 8H).

LC/MS (APSI) m/z [M+H] calculated for C19Cl1H30N4O2S1: 413.2; found: 413.2.

5-(1,2,5,6-Tetrahydro-3-pyridyl)-1-benzothiophen-4-ol - Z8827560409 (Method 6).

Yield: 11%; purity, >95% (assessed by LC/MS).

^{1}H NMR (600 MHz, DMSO-d_6) δ 7.61 (d, J = 5.4 Hz, 1H), 7.57 (d, J = 5.5 Hz, 1H), 7.39 (d, J = 8.2 Hz, 1H), 7.06 (d, J = 8.2 Hz, 1H), 5.85 (d, J = 3.9 Hz, 1H), 3.62 (s, 1H), 2.96 (t, J = 5.9 Hz, 2H), 2.54 (s, 1H), 2.24 (s, 2H), 1.08 - 1.01 (m, 1H), 0.99 (d, J = 6.9 Hz, 1H).

LC/MS (APSI) m/z [M+H] calculated for C13H14N1O1S1: 232.1; found: 232.2.

4-Chloro-5-(1,2,5,6-tetrahydro-3-pyridyl)-1-benzothiophene - Z8827560428 (Method 3).

Yield: 26%; purity, >95% (assessed by LC/MS).

^{1}H NMR (500 MHz, DMSO-d_6) δ 9.50 (s, 2H), 8.02 (d, J = 8.2 Hz, 1H), 7.96 (d, J = 5.5 Hz, 1H), 7.51 (d, J = 5.5 Hz, 1H), 7.28 (d, J = 8.3 Hz, 1H), 5.98 (td, J = 4.0, 2.1 Hz, 1H), 3.85 - 3.81 (m, 2H), 3.24 (d, J = 12.3 Hz, 1H).

LC/MS (APSI) m/z [M+H] calculated for C13Cl1H13N1S1: 250.0; found: 250.0.

5-(4-Methyl-5-indanyl)-1,2,3,6-tetrahydropyridine - Z8827560439 (Method 3).

Yield: 35%; purity, >95% (assessed by LC/MS).

^{1}H NMR (600 MHz, DMSO-d_6) δ 6.97 (d, J = 7.5 Hz, 1H), 6.81 (d, J = 7.6 Hz, 1H), 5.51 (s, 1H), 2.86 - 2.80 (m, 4H), 2.77 (t, J = 7.4 Hz, 2H), 2.20 (s, 1H), 2.14 (s, 3H), 2.09 (s, 2H), 1.99 (p, J = 7.4 Hz, 2H).

LC/MS (APSI) m/z [M+H] calculated for C15H20N1: 214.2; found: 214.2.

5-(2-Methyl-5-indanyl)-1,2,3,6-tetrahydropyridine - Z8827560440 (Method 3).
Yield: 23%; purity, >95% (assessed by LC/MS).

^{1}H NMR (600 MHz, DMSO-d_6) δ 7.17 (s, 1H), 7.16 - 7.07 (m, 2H), 6.14 - 6.09 (m, 1H), 3.53 (d, J = 2.6 Hz, 2H), 2.98 (dt, J = 15.0, 7.2 Hz, 2H), 2.81 (t, J = 5.7 Hz, 2H), 2.47 - 2.41 (m, 2H), 2.14 (dp, J = 6.1, 3.0 Hz, 2H), 1.14 - 1.03 (m, 5H).

LC/MS (APSI) m/z [M+H] calculated for C15H20N1: 214.2; found: 214.2.

6-(1,2,5,6-Tetrahydro-3-pyridyl)-1-indanol - Z8827560451 (Method 6).
Yield: 14%; purity, >95% (assessed by LC/MS).

^{1}H NMR (500 MHz, DMSO-d_6) δ 7.28 (s, 1H), 7.17 (dd, J = 7.8, 1.8 Hz, 1H), 7.13 (d, J = 7.9 Hz, 1H), 6.11 (td, J = 4.2, 2.1 Hz, 1H), 5.17 (s, 1H), 4.99 (t, J = 6.7 Hz, 1H), 3.53 - 3.48 (m, 2H), 2.85 (ddd, J = 15.8, 8.6, 3.8 Hz, 1H), 2.78 (t, J = 5.7 Hz, 2H), 2.65 (dt, J = 16.0, 8.0 Hz, 1H), 2.35 - 2.25 (m, 1H), 2.11 (tq, J = 5.8, 2.9 Hz, 2H), 1.74 (dtd, J = 12.4, 8.2, 6.3 Hz, 1H).

LC/MS (APSI) m/z [M+H] calculated for C14H18N1O1: 216.1; found: 216.2.

5-(4-Methyl-1,2,5,6-tetrahydro-3-pyridyl)-1-benzothiophen-4-ol - Z8827560496 (Method 6).
Yield: 10%; purity, >95% (assessed by LC/MS).

^{1}H NMR (600 MHz, DMSO-d_6) δ 7.57 (t, J = 3.9 Hz, 2H), 7.39 (d, J = 8.1 Hz, 1H), 6.94 (d, J = 8.1 Hz, 1H), 2.91 (s, 2H), 2.54 (s, 1H), 2.03 (s, 2H), 1.43 (s, 3H).

LC/MS (APSI) m/z [M+H] calculated for C14H16N1O1S1: 246.1; found: 246.0.

4-Chloro-5-(4-methyl-1,2,5,6-tetrahydro-3-pyridyl)-1-benzothiophene - Z8827560515 (Method 3).
Yield: 29%; purity, 93% (assessed by LC/MS).

^{1}H NMR (600 MHz, DMSO-d_6) δ 8.55 - 8.31 (m, 1H), 8.15 - 8.00 (m, 1H), 7.98 - 7.89 (m, 1H), 7.61 - 7.47 (m, 1H), 7.42 - 7.32 (m, 1H), 7.17 (d, J = 8.2 Hz, 1H), 3.05 - 2.75 (m, 1H), 2.17 - 1.80 (m, 2H), 1.53 - 1.19 (m, 3H).

LC/MS (APSI) m/z [M+H] calculated for C14Cl1H15N1S1: 264.1; found: 264.0.

3-(2-Azetidinyl)-5-(2-chloro-6-tolyl)-1,2,4-oxadiazole - Z8836317766 (Method 5).
Yield: 55%; purity, >95% (assessed by LC/MS).

^{1}H NMR (500 MHz, DMSO-d_6) δ 9.67 (s, 2H), 7.61 (t, J = 7.8 Hz, 1H), 7.56 (d, J = 7.8 Hz, 1H), 7.46 (d, J = 7.5 Hz, 1H), 5.84 (t, J = 8.5 Hz, 1H), 4.15 (q, J = 9.2 Hz, 1H), 3.99 (td, J = 9.8, 5.8 Hz, 1H), 2.96 - 2.80 (m, 2H), 2.26 (s, 3H).

LC/MS (APSI) m/z [M+H] calculated for C12Cl1H13N3O1: 250.1; found: 250.1.

2-[3-(2-Azetidinyl)-1,2,4-oxadiazol-5-yl]-3-chlorophenol - Z8908003436, Z8836317769 (Method 5).
Yield: 8%; purity, >95% (assessed by LC/MS).

^{1}H NMR (600 MHz, DMSO-d_6) δ 9.57 (s, 2H), 7.51 (t, J = 8.3 Hz, 1H), 7.14 (d, J = 8.0 Hz, 1H), 7.06 (d, J = 8.4 Hz, 1H), 5.84 (t, J = 8.6 Hz, 1H), 4.16 (q, J = 9.2 Hz, 1H), 3.96 (td, J = 9.8, 5.7 Hz, 1H), 2.94 - 2.81 (m, 2H).

LC/MS (APSI) m/z [M+H] calculated for C11Cl1H11N3O2: 252.1; found: 252.0.

4-[3-(2-Azetidinyl)-1,2,4-oxadiazol-5-yl]-3-chlorophenol - Z8904332025, Z8836317771 (Method 5).
Yield: 30%; purity, >95% (assessed by LC/MS).

^{1}H NMR (500 MHz, DMSO-d_6) δ 11.17 (s, 1H), 9.59 (s, 2H), 8.02 (d, J = 8.9 Hz, 1H), 7.08 (d, J = 2.4 Hz, 1H), 6.98 (dd, J = 8.7, 2.4 Hz, 1H), 5.75 (t, J = 8.4 Hz, 1H), 4.12 (q, J = 9.1 Hz, 1H), 3.98 (td, J = 9.8, 6.0 Hz, 1H), 2.94 - 2.78 (m, 2H).

LC/MS (APSI) m/z [M+H] calculated for C11Cl1H11N3O2: 252.1; found: 252.0.

3-[3-(2-Azetidinyl)-1,2,4-oxadiazol-5-yl]-4-chlorophenol - Z8836317772 (Method 5).
Yield: 37%; purity, >95% (assessed by LC/MS).

^{1}H NMR (500 MHz, DMSO-d_6) δ 10.46 (s, 1H), 9.60 (s, 2H), 7.54 (dd, J = 8.8, 3.2 Hz, 1H), 7.48 (d, J = 3.2 Hz, 1H), 7.11 (dt, J = 8.9, 3.3 Hz, 1H), 5.79 (td, J = 8.5, 3.4 Hz, 1H), 4.13 (q, J = 9.3 Hz, 1H), 3.98 (td, J = 9.9, 5.9 Hz, 1H), 2.87 (dqt, J = 15.5, 6.0, 3.2 Hz, 2H).

LC/MS (APSI) m/z [M+H] calculated for C11Cl1H11N3O2: 252.1; found: 252.0.

3-(2-Azetidinyl)-5-(4-chloro-2-pyridyl)-1,2,4-oxadiazole - Z8904329783, Z8836317795 (Method 5).
Yield: 5%; purity, 95% (assessed by LC/MS).
LC/MS (APSI) m/z [M+H] calculated for C10Cl1H10N4O1: 237.1; found: 237.0.

3-[3-(2-Azetidinyl)-1,2,4-oxadiazol-5-yl]-5-chlorophenol - Z8836317802 (Method 5).
Yield: 45%; purity, >95% (assessed by LC/MS).
^{1}H NMR (500 MHz, DMSO-d_6) δ 10.80 (s, 1H), 9.55 (s, 2H), 7.57 (t, J = 1.8 Hz, 1H), 7.49 - 7.42 (m, 1H), 7.18 (t, J = 2.2 Hz, 1H), 5.76 (t, J = 8.4 Hz, 1H), 4.12 (q, J = 9.1 Hz, 1H), 3.99 (td, J = 9.8, 6.2 Hz, 1H), 2.85 (qd, J = 8.8, 5.9 Hz, 2H).
LC/MS (APSI) m/z [M+H] calculated for C11Cl1H11N3O2: 252.1; found: 252.0.

4-[3-(2-Azetidinyl)-1,2,4-oxadiazol-5-yl]-3-cresol - Z8843617521 (Method 5).
Yield: 59%; purity, >95% (assessed by LC/MS).
^{1}H NMR (500 MHz, DMSO-d_6) δ 10.55 (s, 1H), 9.60 (s, 2H), 7.94 (d, J = 8.4 Hz, 1H), 6.82 (d, J = 8.3 Hz, 2H), 5.72 (t, J = 8.5 Hz, 1H), 4.12 (q, J = 9.3 Hz, 1H), 3.99 (td, J = 9.8, 6.4 Hz, 1H), 2.92 - 2.79 (m, 2H), 2.59 (d, J = 2.8 Hz, 3H).
LC/MS (APSI) m/z [M+H] calculated for C12H14N3O2: 232.1; found: 232.2.

3-[3-(2-Azetidinyl)-1,2,4-oxadiazol-5-yl]-2-cresol - Z8843617522 (Method 5).
Yield: 34%; purity, >95% (assessed by LC/MS).
^{1}H NMR (500 MHz, DMSO-d_6) δ 10.07 (s, 1H), 9.60 (s, 2H), 7.44 (d, J = 7.8 Hz, 1H), 7.25 (t, J = 7.9 Hz, 1H), 7.13 (d, J = 8.0 Hz, 1H), 5.77 (t, J = 8.5 Hz, 1H), 4.13 (q, J = 9.2 Hz, 1H), 4.00 (td, J = 9.9, 6.1 Hz, 1H), 2.95 - 2.80 (m, 2H), 2.45 (s, 3H).
LC/MS (APSI) m/z [M+H] calculated for C12H14N3O2: 232.1; found: 232.0.

2-[3-(2-Azetidinyl)-1,2,4-oxadiazol-5-yl]-3-cresol - Z8843617525 (Method 5).
Yield: 22%; purity, 95% (assessed by LC/MS).
^{1}H NMR (600 MHz, DMSO-d_6) δ 10.43 (s, 1H), 9.54 (s, 2H), 7.36 (t, J = 7.9 Hz, 1H), 6.88 (dd, J = 15.4, 7.9 Hz, 2H), 5.80 (t, J = 8.5 Hz, 1H), 4.15 (q, J = 9.1 Hz, 1H), 3.99 (td, J = 9.9, 5.8 Hz, 1H), 2.95 - 2.81 (m, 2H), 2.29 (s, 3H).
LC/MS (APSI) m/z [M+H] calculated for C12H14N3O2: 232.1; found: 232.2.

3-[3-(2-Azetidinyl)-1,2,4-oxadiazol-5-yl]-4-cresol - Z8904332022, Z8843617526 (Method 5).
Yield: 12%; purity, 92% (assessed by LC/MS).
^{1}H NMR (600 MHz, DMSO-d_6) δ 9.86 (d, J = 10.0 Hz, 1H), 9.53 (s, 1H), 7.50 - 7.40 (m, 1H), 7.30 (t, J = 7.8 Hz, 1H), 7.05 - 6.97 (m, 1H), 5.78 (t, J = 8.4 Hz, 1H), 4.15 (q, J = 9.2 Hz, 1H), 4.01 (td, J = 9.8, 6.1 Hz, 1H), 2.95 - 2.82 (m, 1H), 2.55 (d, J = 7.6 Hz, 3H).
LC/MS (APSI) m/z [M+H] calculated for C12H14N3O2: 232.1; found: 232.2.

3-(m-Fluorophenyl)-3-(1,5,7-triaza-1H-inden-4-ylcarbonylamino)propionic acid - Z8928826033 (Method 8).
Yield: 57%; purity, >95% (assessed by LC/MS).
^{1}H NMR (500 MHz, DMSO-d_6) δ 12.36 (s, 1H), 9.57 (d, J = 8.8 Hz, 1H), 8.86 (s, 1H), 7.71 (t, J = 2.9 Hz, 1H), 7.35 (td, J = 8.0, 5.9 Hz, 1H), 7.32 - 7.26 (m, 2H), 7.05 (td, J = 8.6, 2.7 Hz, 1H), 6.99 (dd, J = 3.4, 1.6 Hz, 1H), 5.47 (td, J = 8.6, 4.2 Hz, 1H), 3.06 (dd, J = 16.1, 8.2 Hz, 1H), 2.88 (dd, J = 16.2, 5.9 Hz, 1H).
LC/MS (APSI) m/z [M+H] calculated for C16F1H14N4O3: 329.1; found: 329.0.

3-Phenyl-3-(1,5,7-triaza-1H-inden-4-ylcarbonylamino)butyric acid - Z8928826043, Z8928826043 (Method 8).
Yield: 39%; purity, >95% (assessed by LC/MS).
^{1}H NMR (600 MHz, DMSO-d_6) δ 12.38 (s, 1H), 9.42 (s, 1H), 8.87 (s, 1H), 7.71 (t, J = 2.9 Hz, 1H), 7.44 - 7.40 (m, 2H), 7.32 (t, J = 7.7 Hz, 2H), 7.22 (t, J = 7.3 Hz, 1H), 6.96 (dd, J = 3.4, 1.6 Hz, 1H), 3.11 (d, J = 14.9 Hz, 1H), 2.95 (d, J = 14.9 Hz, 1H), 1.91 (s, 3H).
LC/MS (APSI) m/z [M+H] calculated for C17H17N4O3: 325.1; found: 325.2.

(S)-3-(3-Pyridyl)-3-(1,5,7-triaza-1H-inden-4-ylcarbonylamino)propionic acid - Z8929428675 (Method 8).
Yield: 31%; purity, 93% (assessed by LC/MS).

^{1}H NMR (600 MHz, DMSO-d_6) δ 12.37 (s, 1H), 9.65 (d, J = 8.7 Hz, 1H), 8.87 (s, 1H), 8.66 (d, J = 2.3 Hz, 1H), 8.45 (dd, J = 4.7, 1.6 Hz, 1H), 7.89 (dt, J = 8.0, 2.0 Hz, 1H), 7.74 - 7.70 (m, 1H), 7.36 (dd, J = 8.0, 4.7 Hz, 1H), 7.00 (d, J = 3.4 Hz, 1H), 5.50 (td, J = 8.3, 6.2 Hz, 1H), 3.16 - 3.06 (m, 1H), 2.99 - 2.89 (m, 1H), 2.54 (s, 1H).

LC/MS (APSI) m/z [M+H] calculated for C15H14N5O3: 312.1; found: 312.2.

(S)-3-(o-Tolyl)-3-(1,5,7-triaza-1H-inden-4-ylcarbonylamino)propionic acid - Z8929429249 (Method 8).
Yield: 39%; purity, >95% (assessed by LC/MS).

^{1}H NMR (500 MHz, DMSO-d_6) δ 12.35 (s, 1H), 9.49 (d, J = 8.6 Hz, 1H), 8.85 (s, 1H), 7.72 - 7.67 (m, 1H), 7.53 (d, J = 7.3 Hz, 1H), 7.13 (qd, J = 6.7, 4.8 Hz, 3H), 6.98 (dd, J = 3.5, 1.6 Hz, 1H), 5.68 (td, J = 8.5, 5.8 Hz, 1H), 2.99 (dd, J = 16.0, 8.4 Hz, 1H), 2.79 (dd, J = 16.0, 5.8 Hz, 1H), 2.45 (s, 3H).

LC/MS (APSI) m/z [M+H] calculated for C17H17N4O3: 325.1; found: 325.2.

(S)-3-Phenyl-3-(6-purinylcarbonylamino)propionic acid - Z8990523169 (Method 8).
Yield: 35%; purity, >95% (assessed by LC/MS).

^{1}H NMR (600 MHz, DMSO-d_6) δ 12.30 (s, 1H), 9.74 (d, J = 8.7 Hz, 1H), 9.08 (s, 1H), 8.75 (s, 1H), 7.50 - 7.46 (m, 2H), 7.33 (t, J = 7.6 Hz, 2H), 7.24 (t, J = 7.4 Hz, 1H), 5.52 (td, J = 8.4, 5.9 Hz, 1H), 3.12 (dd, J = 16.2, 8.3 Hz, 1H), 2.91 (dd, J = 16.2, 5.9 Hz, 1H).

LC/MS (APSI) m/z [M+H] calculated for C15H14N5O3: 312.1; found: 312.0.

(S)-3-(6-Methyl-1,5,7-triaza-1H-inden-4-ylcarbonylamino)-3-phenylpropionic acid - Z8990523172 (Method 8).
Yield: 53%; purity, >95% (assessed by LC/MS).

^{1}H NMR (500 MHz, DMSO-d_6) δ 12.31 (s, 1H), 12.07 (s, 1H), 9.32 (d, J = 8.9 Hz, 1H), 7.58 (t, J = 2.9 Hz, 1H), 7.44 (d, J = 7.3 Hz, 2H), 7.32 (t, J = 7.6 Hz, 2H), 7.23 (t, J = 7.2 Hz, 1H), 6.91 (dd, J = 3.5, 1.7 Hz, 1H), 5.47 (td, J = 8.1, 5.9 Hz, 1H), 3.06 (dd, J = 16.1, 7.8 Hz, 1H), 2.90 (dd, J = 16.1, 6.1 Hz, 1H), 2.71 (s, 3H).

LC/MS (APSI) m/z [M+H] calculated for C17H17N4O3: 325.1; found: 325.2.

(S)-3-(3-Methyl-1,5,7-triaza-1H-inden-4-ylcarbonylamino)-3-phenylpropionic acid - Z8990523176 (Method 8).
Yield: 57%; purity, >95% (assessed by LC/MS).

^{1}H NMR (500 MHz, DMSO-d_6) δ 12.25 (s, 1H), 12.04 (s, 1H), 9.30 (d, J = 8.7 Hz, 1H), 8.74 (s, 1H), 7.47 - 7.39 (m, 3H), 7.33 (t, J = 7.5 Hz, 2H), 7.24 (t, J = 7.3 Hz, 1H), 5.47 (td, J = 8.4, 5.9 Hz, 1H), 2.94 (dd, J = 16.0, 8.2 Hz, 1H), 2.82 (dd, J = 16.0, 6.1 Hz, 1H), 2.24 (s, 3H).

LC/MS (APSI) m/z [M+H] calculated for C17H17N4O3: 325.1; found: 325.0.

(S)-3-(6-Chloro-1,5,7-triaza-1H-inden-4-ylcarbonylamino)-3-phenylpropionic acid - Z8990523194 (Method 8).
Yield: 52%; purity, >95% (assessed by LC/MS).

^{1}H NMR (500 MHz, DMSO-d_6) δ 12.57 (s, 1H), 12.33 (s, 1H), 9.38 (d, J = 8.7 Hz, 1H), 7.74 (d, J = 3.6 Hz, 1H), 7.44 (d, J = 7.3 Hz, 2H), 7.31 (t, J = 7.6 Hz, 2H), 7.23 (t, J = 7.3 Hz, 1H), 6.98 (d, J = 3.5 Hz, 1H), 5.47 (td, J = 8.3, 5.7 Hz, 1H), 3.08 (dd, J = 16.1, 8.1 Hz, 1H), 2.88 (dd, J = 16.2, 5.9 Hz, 1H).

LC/MS (APSI) m/z [M+H] calculated for C16Cl1H14N4O3: 345.1; found: 345.2.

3-(o-Fluorophenyl)-3-(1,5,7-triaza-1H-inden-4-ylcarbonylamino)propionic acid - Z8990527720 (Method 8).
Yield: 18%; purity, >95% (assessed by LC/MS).

^{1}H NMR (600 MHz, DMSO-d_6) δ 12.38 (s, 1H), 9.57 (d, J = 8.7 Hz, 1H), 8.88 (s, 1H), 7.72 (d, J = 3.5 Hz, 1H), 7.59 - 7.54 (m, 1H), 7.33 - 7.27 (m, 1H), 7.21 - 7.14 (m, 2H), 6.99 (d, J = 3.4 Hz, 1H), 5.75 (td, J = 8.4, 5.5 Hz, 1H), 3.04 (dd, J = 16.1, 8.4 Hz, 1H), 2.83 (dd, J = 16.1, 5.5 Hz, 1H).

LC/MS (APSI) m/z [M+H] calculated for C16F1H14N4O3: 329.1; found: 329.0.

3-(m-Hydroxyphenyl)-3-(1,5,7-triaza-1H-inden-4-ylcarbonylamino)propionic acid - Z8990523184, Z8990919037 (Method 8).
Yield: 37%; purity, >95% (assessed by LC/MS).

^{1}H NMR (500 MHz, DMSO-d_6) δ 12.36 (s, 1H), 12.27 (s, 1H), 9.45 (d, J = 8.9 Hz, 1H), 9.33 (s, 1H), 8.85 (s, 1H), 7.71 (d, J = 3.1 Hz, 1H), 7.09 (t, J = 7.8 Hz, 1H), 7.00 (d, J = 3.4 Hz, 1H), 6.88 - 6.80 (m, 2H), 6.60 (dd, J = 8.1, 2.4 Hz, 1H), 5.37 (t, J = 7.7 Hz, 1H), 3.00 (dd, J = 16.1, 7.8 Hz, 1H), 2.82 (dd, J = 16.0, 5.9 Hz, 1H).

LC/MS (APSI) m/z [M+H] calculated for C16H15N4O4: 327.1; found: 327.2.

(2R,3S)-2-Hydroxy-3-phenyl-3-(1,5,7-triaza-1H-inden-4-ylcarbonylamino)propionic acid - Z8990919048 (Method 8).

Yield: 52%; purity, >95% (assessed by LC/MS).

^{1}H NMR (500 MHz, DMSO-d_6) δ 12.40 (s, 1H), 9.27 (d, J = 9.1 Hz, 1H), 8.90 (s, 1H), 7.72 (t, J = 2.9 Hz, 1H), 7.39 (d, J = 7.7 Hz, 2H), 7.32 (t, J = 7.5 Hz, 2H), 7.24 (t, J = 7.3 Hz, 1H), 6.97 (d, J = 3.4 Hz, 1H), 5.47 (dd, J = 9.1, 3.1 Hz, 1H), 4.41 (d, J = 3.0 Hz, 1H).

LC/MS (APSI) m/z [M+H] calculated for C16H15N4O4: 327.1; found: 327.2.

3-(o-Hydroxyphenyl)-3-(1,5,7-triaza-1H-inden-4-ylcarbonylamino)propionic acid - Z8990523178, Z8991052050 (Method 8).

Yield: 47%; purity, >95% (assessed by LC/MS).

^{1}H NMR (500 MHz, DMSO-d_6) δ 12.37 (s, 1H), 12.20 (s, 1H), 9.87 (s, 1H), 9.43 (d, J = 9.3 Hz, 1H), 8.85 (s, 1H), 7.73 - 7.69 (m, 1H), 7.21 (dd, J = 7.6, 1.8 Hz, 1H), 7.06 (td, J = 7.7, 1.8 Hz, 1H), 7.01 (dd, J = 3.5, 1.7 Hz, 1H), 6.82 (d, J = 8.0 Hz, 1H), 6.72 (t, J = 7.6 Hz, 1H), 5.66 - 5.57 (m, 1H), 2.92 (dd, J = 15.7, 7.8 Hz, 1H), 2.81 (dd, J = 15.7, 5.8 Hz, 1H).

LC/MS (APSI) m/z [M+H] calculated for C16H15N4O4: 327.1; found: 327.2.
