# Supplementary material for: Development of a random background to understand ligand optimization
Source: Nature. 2026 Sep 16;658(8138):1341–9. doi: 10.1038/s41586-026-11013-5 (PMC13716815; doi:10.1038/s41586-026-11013-5)
Supplement: Supplementary file 2 [file 41586_2026_11013_MOESM2_ESM.pdf]

nature portfolio

Double-anonymous peer review submissions: write DAPR and your manuscript number here

Corresponding author(s): Bryan L. Roth; James S. Fraser; Brian K. Shoichet

Last updated by author(s): 2026-07-23

Reporting Summary

Nature Portfolio wishes to improve the reproducibility of the work that we publish. This form provides structure for consistency and transparency in reporting. For further information on Nature Portfolio policies, see our Editorial Policies and the Editorial Policy Checklist.

Statistics

For all statistical analyses, confirm that the following items are present in the figure legend, table legend, main text, or Methods section.

n/a	Confirmed	
☐	☒	The exact sample size (*n*) for each experimental group/condition, given as a discrete number and unit of measurement
☐	☒	A statement on whether measurements were taken from distinct samples or whether the same sample was measured repeatedly
☐	☒	The statistical test(s) used AND whether they are one- or two-sided<br>*Only common tests should be described solely by name; describe more complex techniques in the Methods section.*
☒	☐	A description of all covariates tested
☐	☒	A description of any assumptions or corrections, such as tests of normality and adjustment for multiple comparisons
☐	☒	A full description of the statistical parameters including central tendency (e.g. means) or other basic estimates (e.g. regression coefficient) AND variation (e.g. standard deviation) or associated estimates of uncertainty (e.g. confidence intervals)
☐	☒	For null hypothesis testing, the test statistic (e.g. *F*, *t*, *r*) with confidence intervals, effect sizes, degrees of freedom and *P* value noted<br>*Give P values as exact values whenever suitable.*
☒	☐	For Bayesian analysis, information on the choice of priors and Markov chain Monte Carlo settings
☒	☐	For hierarchical and complex designs, identification of the appropriate level for tests and full reporting of outcomes
☐	☒	Estimates of effect sizes (e.g. Cohen's *d*, Pearson's *r*), indicating how they were calculated

Our web collection on statistics for biologists contains articles on many of the points above.

Software and code

Policy information about availability of computer code

Data collection	ALS beamline 8.3.1: Blu-Ice 5.
Data analysis	Confirmed software and version details are listed on appended page 6.

For manuscripts utilizing custom algorithms or software that are central to the research but not yet described in published literature, software must be made available to editors and reviewers. We strongly encourage code deposition in a community repository (e.g. GitHub). See the Nature Portfolio guidelines for submitting code & software for further information.

Data

Policy information about availability of data

All manuscripts must include a data availability statement. This statement should provide the following information, where applicable:

- Accession codes, unique identifiers, or web links for publicly available datasets
- A description of any restrictions on data availability
- For clinical datasets or third party data, please ensure that the statement adheres to our policy

All primary data are available through the manuscript, Extended Data, Supplementary Information and Source Data. Cryo-EM structures/maps: PDB 9PLO/EMD-71719 and PDB 9PLN/EMD-71718. Mac1 structures and structure factors: PDB 14AB, 7IIW, 7IIX, 7IIY, 7IIZ, 7IJ0, 7IJ1, 7IJ2, 7IJ3, 7IJ4, 7IJ5, 7IJ6, 7IJ7, 7IJ8, 7IJ9, 7IJA, 7IJB, 7IJC, 7IJD, 7IJE, 7IJF, 7IJG, 7IJH, 7IJI, 7IJJ, 7IJK, 7IJL, 14AM, 14AN, 14AO, 14AP and 7IJM. ChEMBL database version 34 is available at https://doi.org/10.6019/CHEMBL.database.34. Compounds and catalogue identifiers are listed in Supplementary Table 1.

Research involving human participants, their data, or biological material

Policy information about studies with human participants or human data. See also policy information about sex, gender (identity/presentation), and sexual orientation and race, ethnicity and racism.

Reporting on sex and gender	N/A
Reporting on race, ethnicity, or other socially relevant groupings	N/A
Population characteristics	N/A
Recruitment	N/A
Ethics oversight	N/A

Note that full information on the approval of the study protocol must also be provided in the manuscript.

Field-specific reporting

Please select the one below that is the best fit for your research. If you are not sure, read the appropriate sections before making your selection.

☒ Life sciences ☐ Behavioural & social sciences ☐ Ecological, evolutionary & environmental sciences

For a reference copy of the document with all sections, see nature.com/documents/nr-reporting-summary-flat.pdf

Life sciences study design

All studies must disclose on these points even when the disclosure is negative.

Sample size	We synthesized 257 analogs of 18 parent compounds. Sample sizes were not predetermined by statistical methods but were driven by compound availability and feasibility. For mouse studies, no formal a priori power calculation was performed; group sizes followed prior assay experience and literature precedent.
Data exclusions	No data were excluded.
Replication	Fig. 5b comprises three independent experiments, each with two technical replicates. Mouse behavioral experiments were performed in two independent cohorts.
Randomization	Mice were randomly assigned to treatment and control groups as described in the Methods.
Blinding	For mouse experiments, behavioral assessment was performed by an experimenter different from the injector; behavioral investigators were therefore blinded to treatment.

Reporting for specific materials, systems and methods

We require information from authors about some types of materials, experimental systems and methods used in many studies. Here, indicate whether each material, system or method listed is relevant to your study. If you are not sure if a list item applies to your research, read the appropriate section before selecting a response.

Materials & experimental systems

n/a	Involved in the study	
☐	☒	Antibodies
☐	☒	Eukaryotic cell lines
☒	☐	Palaeontology and archaeology
☐	☒	Animals and other organisms
☒	☐	Clinical data
☒	☐	Dual use research of concern
☒	☐	Plants

Methods

n/a	Involved in the study	
☒	☐	ChIP-seq
☒	☐	Flow cytometry
☒	☐	MRI-based neuroimaging

Eukaryotic cell lines

Policy information about cell lines and Sex and Gender in Research

Cell line source(s)	Sf9: Expression Systems 94-001S; Hi5: Expression Systems 94-002S; HEK293: ATCC CRL-1573; HEK293T: ATCC CRL-3216.
Authentication	All cell lines were authenticated by their respective suppliers.
Mycoplasma contamination	The cell lines were not tested for mycoplasma contamination by the authors.
Commonly misidentified lines (See ICLAC register)	no commonly misidentified cell lines were used.

Plants

Seed stocks	*Report on the source of all seed stocks or other plant material used. If applicable, state the seed stock centre and catalogue number. If plant specimens were collected from the field, describe the collection location, date and sampling procedures.*
Novel plant genotypes	*Describe the methods by which all novel plant genotypes were produced. This includes those generated by transgenic approaches, gene editing, chemical/radiation-based mutagenesis and hybridization. For transgenic lines, describe the transformation method, the number of independent lines analyzed and the generation upon which experiments were performed. For gene-edited lines, describe the editor used, the endogenous sequence targeted for editing, the targeting guide RNA sequence (if applicable) and how the editor was applied.*
Authentication	*Describe any authentication procedures for each seed stock used or novel genotype generated. Describe any experiments used to assess the effect of a mutation and, where applicable, how potential secondary effects (e.g. second site T-DNA insertions, mosiacism, off-target gene editing) were examined.*

Animals and other organisms

Policy information about studies involving animals and other organisms

Laboratory animals	Male C57BL/6J mice, 7-8 weeks old, obtained from The Jackson Laboratory (JAX strain #000664).
Housing conditions	Five mice per cage; standard 12:12-hour light-dark cycle; 20.7 C (69.3 F); 58% humidity.
Ethics oversight	University of California, San Francisco Institutional Animal Care and Use Committee (IACUC), protocol #AN208219.
Randomization	Animals were randomly assigned to treatment and control groups.
Blinding	The experimenter assessing behavior was different from the experimenter administering injections and was blinded to treatment.
Replication	Behavioral experiments were conducted in two independent cohorts.
Wild animals	Not applicable.
Field-collected samples	Not applicable.

The corresponding information has also been added to the manuscript Methods and Fig. 5 legend.

Antibodies

Policy information about antibodies used in the study

Antibody/reagent	Anti-His6-Eu3+ cryptate, used as the HTRF donor reagent.
Supplier	PerkinElmer.
Catalogue number	AD0402.
Application	Mac1 HTRF displacement assay for detection of His6-tagged protein.
Dilution	1:20,000.
Validation	Validated by the manufacturer for HTRF detection of His6-tagged proteins.

Supplier, catalogue number, application, dilution and manufacturer validation are also stated in the author response.

Software and code details

Confirmed software and instrument-software versions

X-ray data collection	Blu-Ice 5 at ALS beamline 8.3.1.
X-ray processing	XDS, dated 19 Jan 2025, BUILT=20250430; Aimless 0.8.2.
Density/model analysis	PanDDA 0.2.14; COOT 0.8.9.2; phenix.refine 1.21.2_5419.
Structural visualization	PyMOL 3.1.8; ChimeraX 1.8.
Cryo-EM processing	cryoSPARC 4.5.3.
Mac1 instruments	Echo 650 Liquid Handler 3.1.0; EnVision 2105-0010 software 1.14.3049.1193.
Plate-reader analysis	SoftMax Pro 7.
Statistical/data analysis	GraphPad Prism 9.0, 10.0 and 10.0.2; Microsoft Excel 16.99.2.
FEP calculations	Schrodinger FEP+ 2025-2 with the OPLS4 force field.
Cheminformatics/analysis	Corina 3.60; Omega 3.0.1; ChemAxon JChem 21.13; RDKit 2020.09.1; Python 2.7 and 3.7; R 4.2.0 (ggplot2).
