# Supplementary material for: Development of a random background to understand ligand optimization
Source: Nature. 2026 Sep 16;658(8138):1341–9. doi: 10.1038/s41586-026-11013-5 (PMC13716815; doi:10.1038/s41586-026-11013-5)
Supplement: Supplementary file 3 [file 41586_2026_11013_MOESM4_ESM.pdf]

nature portfolio

Peer Review File

Development of a Random Background to Understand Ligand Optimization

Corresponding Author: Professor Brian Shoichet

Any redactions in this file are there to maintain patient confidentiality, the confidentiality of unpublished data, or to remove third-party material.

This file contains all reviewer reports in order by version, followed by all author rebuttals in order by version.

Version 0:

Reviewer comments:

Referee #1

(Remarks to the Author)
I enjoyed this paper a great deal! Right up front, I want to note that I believe that your reference 45 should be more properly cited to Angew. Chem. (https://doi.org/10.1002/anie.201303207). And I believe that the Chiodi et al. "Magic Chloro" paper really needs to be cited as well (https://pubs.acs.org/doi/abs/10.1021/acs.jmedchem.2c02015). I would also consider adding a citation to the Cramer et al. paper on hydroxy substitution in medicinal chemistry (https://pubs.acs.org/doi/abs/10.1021/acs.jmedchem.9b00179). A similar paper on adding fluorines to med-chem scaffolds is Gillis et al. (https://pubs.acs.org/doi/abs/10.1021/acs.jmedchem.9b00179).

It will be extremely interesting for those of us inside drug discovery organizations to look at our own data on the compounds made during specific compound optimization campaigns to see how well we have been doing compared to this paper's random-background estimate!

Here are some specific comments on the main text. The ones to lines 115, 250, and 407 could be actionable with regards to the text of the manuscript. I think that addressing these could add to the strength of the manuscript, but I will say that it is quite strong as it stands.

Line 41: The “thousands” figure shows up a lot, particularly in the popular press, but I have only been involved in one project in the past 35 years that synthesized that many analogs. However, that shows it is possible, so this statement (which reappears in line 64) can stand.

Lines 115-116: It’s true that these are applied without design, but there is a bias towards synthetic tractability. Indeed, this seems to have led to the great majority of possible analogs here not being synthesized at all. That may seem like a nit-picking point, but it’s one of the systematic biases in the synthetic chemistry literature that has made machine-learning approaches across it very difficult. Other related ones include the commercial availability and cost of starting materials and reagents, the operational difficulty of setting up particular experiments versus alternatives, faddish popularity of certain reactions, reagents, and catalysts at various times, etc.

Line 155: I was waiting and hoping to see how many of these reduced potency! This is about the ratio of harm/help that I would have predicted. After all, you’re starting with molecules that are already (given the total number of possibilities) very much at the tip of the spear, so you’d figure that any changes have a greater chance of being for the worse.

Line 166: The “magic methyl effect” gets some more empirical confirmation!

Line 170: Interesting comparison with F. Most of the time, though, medicinal chemists are using fluorine for metabolic stability, and these smaller changes in potency are in line with my own experience, for what that’s worth.

Line 178: If these questions hadn’t been raised explicitly in the paper, I was going to insist on them myself! Glad to see this analysis.

Line 231: I am absolutely certain that this point is correct, and I'm glad to see it made.

Lines 250-257: Were there any analogs that showed less favorable PK? The biggest possibility I can see would be adding a phenol to an aromatic core.

Lines 259 et seq: It's good to see this laid out so openly - medicinal chemists generally discover this, but for some reason people seem reluctant to say it.

Lines 306 et seq: I would sum this up as "FEP calculations are often most accurate in the situations where you don't need them as much". But that may just be my own biases!

Lines 330 et seq: This seems particularly relevant given the current FDA leadership's stated desires to do more through calculation/AI/ML and less by experiment, which personally I think is extremely ill-advised.

Line 407: To me, the two halves of this sentence don't quite line up - the following sentence mitigates this appropriately, but I'd like to see this reworded to stop people from reading the first one and walking away, as it were. Perhaps "implying that can can in theory eventually guide compound selection".

Line 411: If it weren't possible, the drug industry wouldn't exist (and folks like me wouldn't have been able to make a career out of it!).

Line 433: I've come around to wearing it like a badge of honor for when we can overcome the problem!

Referee #2

(Remarks to the Author)
The study addresses a compelling and timely research question, offering insights that could significantly advance understanding in the field. Its approach—the establishment of a baseline for ligand optimization—demonstrates a creative effort to tackle a complex issue.

A major limitation significantly affects the study's reliability and interpretability: the interchangeable use of the terms affinity and potency throughout the manuscript. These are distinct pharmacological concepts. Affinity specifically refers to the strength of the interaction between a drug and its target, whereas potency is a composite measure that reflects both affinity and efficacy, and is highly system-dependent. This conflation appears to stem from the study design, where the authors used different activity measures depending on the biological target. According to the Methods and Results (lines 149–154), potency was assessed for mOR and AmpC; both potency and affinity for a2A and CB2; and affinity alone for SERT and Mac1. As a result, the manuscript frequently shifts between discussing affinity and potency—sometimes within the same sentence or paragraph—without clear distinction. This is problematic, as these parameters are not interchangeable and should not be pooled as a single global measure of "activity." Affinity does not always correlate directly with potency. In systems with high target expression, changes in potency may reflect changes in affinity, efficacy, or both—but without additional mechanistic data, it is impossible to determine the relative contribution of each. Only in systems with low target expression can shifts in potency (affinity) and efficacy (maximal response) be more reliably distinguished in functional assays. However, the manuscript does not clearly report the expression levels of the various biological targets, which further complicates interpretation.

This ambiguity limits the robustness of the conclusions and weakens the generalizability of the findings. For example, depending on the system, a single chemical modification could alter affinity without affecting potency (only affecting maximal response), or vice versa. Has the team observed cases where a compound affected efficacy (as reflected by a change in maximal response) without altering potency? Contrastingly, have there been instances where a potency shift was misinterpreted as a change in affinity, when in fact it was driven by efficacy and system-specific factors (e.g., receptor overexpression)? Has any well-known strong agonist used in parallel to support the robustness of the study? When both potency and affinity have be quantified, which one is reported and used for interpretation? These considerations are essential for accurate interpretation and structure–activity relationship (SAR) analysis, and in my view are unclear at this stage.

This misuse of affinity vs potency is found throughout the whole manuscript. Here is listed a sample of the places where affinity and potency have been used (beyond the narrative throughout the manuscript):
- Table 1: only EC50 are mentioned—why not affinity? and if measuring both, why not presenting both?
- Fig. 2a: only affinity is mentioned (i.e, "affinity improvement with 95% CI), why not potency?
- Fig. 3: when stating "structural basis of affinity changes…" is this only for the biological targets for which affinity estimates where quantified?
- Fig. 4 legends: this time the term potency is used; was this only for the biological targets where potency where measured?

While the study makes a valuable contribution by opening a new line of inquiry, without further clarifications, potentially additional assays to equally quantify affinity (or potency) to all the biological target as a single parameter, it is unclear whether this manuscript is worthy of publication.

Another major limitation of the study is the lack a clarity in Fig. 6, and in particular Fig. 6c-e. It is unspecified if the authors

performed power calculation and determined the number of animals for their study. There is no associated table that present the data as mean +/- error and animal number in the manuscript. Also, what type of statistical analysis and post-hoc analysis was performed to present significance? Without the summarising table of data, it is unclear how dosing might be significantly different from vehicle. In particular, 4905 at 5 mg/kg, is pretty much at the same level as vehicle, but yet significantly higher? Similar comment for PS75 at 2.5 and 5 mg/kg? These outcomes seem relatively unlikely, but without the table summarising the data, it is impossible to assess. Some group for dosing only had 5 animals; is that sufficient? Have the authors been reusing the animals from one assay to the other after a wash-out period? In figure 6d, the y-axis stipulates “mechanical threshold (/baseline). It is unclear how the baseline can be at 100% then?

In addition to these primary concerns noted above, the study has several minor limitations that, while not undermining its core contributions, should be acknowledged and addressed.

1. How were the 18 parent molecules selected? Why these and not others? What was the rationale for selecting 3 class A GPCRs (no class B, or C or others?), 1 transporter, and 2 enzymes? Why selecting only 1 parent for mOR, whilst in contrast the authors selected 4 parents for a2A, or 6 for sert? Can these bias in some way the “randomness” of the background method intended?

2. Another major concern is the “unbiased” nature of the study. I appreciate the selection of possible single chemical modifications present in Fig. 1a (middle panel), but is there a rationale for not including other simple single atom changes such as N-->C, N-->O, N-->S, O-->S? Would this influence the nature of the random background?

3. Why a mix of binding and/or functional assays for the selected biological targets? There is no obvious rationale for why using only a functional assay for mOR, AmpC, only binding assay for sert and Mac1, and both binding and functional assays for a2A and CB2. Nowhere in the manuscript the affinity estimates appear to be reported (only values are EC50).

4. Table 1 needs to clearly present which parameters (potency or affinity or both) in the column titles.

5. Ext. Data Table 1 is apparently missing 67 analogs (15 for a2A, 2 for ampC, 14 for CB2, 3 for mac1 and 33 for sert). There are only 190 compounds presented. Why are the total 257 analogs not included? What are all the parameters presented in column titles, i.e., exp_dg, fep_dg, fep_unc, fep_ddg, fep_dg_err_abs and fep_ddg_err_abs? As well, only EC50 are reported, not affinity, but for at least 2 targets, only affinity estimated were measured (refer to major comment and point 2). Additionally, it is unclear which atom was modified in ED Table 1. Although there is information about the replacement atom type (Cl, N, F, …), the position of the atom itself is missing.

6. When estimating the random background, the authors arbitrarily set a threshold at 10-fold improvement (of affinity or potency? it remains unclear—refer to my major concern). It is unclear why the authors have decided to include 2 analogs that were below the threshold. Although they are indeed close to the cut-off, if included the authors needs the present a different narrative about their selection method or exclude the 2 values altogether. Of note, this change would only reduce the estimated random background down to 13.6%.

7. l. 208 (SI Table 2): do the authors mean Ext. Data Table 2? If so, this table is about PK properties, not cryo-EM structure resolutions.

8. Ext. Data Table 2 is missing the information about mOR and the 16 analogs generated and interpreted.

9. Fig. 6b: The authors mentioned the higher potency of 4905 compared to 3629 in a cAMP assay and suggest that the second phase of 4905 is due to a low affinity Gs coupling. Have the authors confirmed the Gs coupling of 4905 using methods available to the team? If so, and the position of 4905 and 3629 are almost identical in the binding site, how can 4905 produce a specific Gs response that is not observed with 3629? Could the authors provide a scientific explanation?

Referee #3

(Remarks to the Author)
A. Summary of the key results

The authors attempt to establish a background expectation for ligand potency optimisation through the design and synthesis of a variety of analogs of known reference (parent) compounds. These analogs differ from the parents through the substitution of C-H bonds with four different substituents and aromatic carbons with aromatic nitrogens, where the modifications were synthetically accessible, the synthesis was affordable, and the changes had no effect on overall compound charge. A subset of analogs with C-Cl or C-Me substitutions showed some improvement in potency, which was accompanied by a worsening of measured pharmacokinetic properties. In some cases crystal structures of parents and analogs was used to rationalize changes in potency. Predicting these improvements in potency computationally was shown to be challenging in the absence of experimentally determined crystal structures. The authors claim a surprise increase in potency of 14% for their random changes compared to 6% of an analysis of non-random similar matched molecular pairs from ChEMBl.

B. Originality and significance: if not novel, please include reference

This is a well written paper with some interesting observations but suffers from a lack of novel insight and discussion of well-

established concepts in medicinal chemistry that have followed the Topliss Tree and QSAR era of the 1970-1980's, primarily that increasing logP through C-Cl and C-Me substitution leads to improvements in potency at the expense of pharmacokinetic properties primarily through the hydrophobic effect. The changes in key physicochemical properties such as logP of these analogs is not discussed, despite the fact that numerous studies looking at ligand optimisation in the context of physiochemical properties have been published, establishing commonly used metrics such as ligand efficiency (LE) and lipophilic efficiency (LipE). There are many relevant references including Miller et al., J. Med. Chem., 2020, 63, 12156-12170.

The analysis of the structural bases for the affinity changes is comprehensive and well explained but also suffers from a similar lack of new insight into protein-ligand interactions. C-F is a well-known substitution for C-H (Meanwell, J. Med. Chem., 2018, 61, 5822-5880) and so causes minimal structural perturbation. Similarly, lipophilic substituents (C-Cl, C-Me) will make favourable interactions with hydrophobic side chains and when solvent exposed will lead to little change in potency (Freire, Drug Disc. Today, 2008, 13, 869-874). The observation that FEP+ performs better with experimental data is also well known (Schindler et al., J. Chem. Inf. Model., 2020, 60, 5457-5474).

The paper concludes by presenting an example of improving a parent compound (3629 -> 4905) where the increase in potency is not offset by a decrease in PK performance to the same degree. This example highlights the whole reason drug discovery is possible yet so challenging. Medicinal chemistry optimisation as a field would not exist if these exceptions did not exist. Although this is a nice, well explored example of how single atom changes can be important it is not a novel concept or finding and has been described extensively. See for example Young et al., J. Med. Chem., 2018, 61, 6421-6467 (ref 34 in manuscript).

C. Data & methodology: validity of approach, quality of data, quality of presentation

The study would benefit from a statistical analysis and discussion of what number of molecules would be sufficient to establish a random background and why the background isn't being evaluated per parent; although over 250 molecules are made, this is across a large series of parent compounds. The number of analogs per parent is actually quite low when compared to the number that could be evaluated based on the authors' criteria, and seemingly below the threshold to establish a truly random sample. Although systematic, the analogs made seem hardly unbiased and H, F, Cl, Me, OH, N are pretty standard SAR changes and not random at all. I question the claim that the dataset in ChEMBL is "biased" more than I would question that this study is "unbiased." Both datasets suffer from the same bias of organic chemistry in that there's not a huge number of single atom substitutions you can put on or substitute C for that are chemically stable and synthesizable.

D. Appropriate use of statistics and treatment of uncertainties

I have a limited knowledge of statistics, but for a manuscript which endeavours to establish a link between molecular changes and the chance of changes in potency and ADME properties, errors in fundamental measurements and error propagation to the derived values used in the headline analyses are notably lacking. For a majority of analogs, affinities have been derived mainly from IC50 values using the Cheng-Prusoff equation. The Z' values for the assays used to determine EC50s are not reported, nor are any errors for the IC50 values or derived Ki's. It is therefore difficult to accept many of the claims of affinity or ADME differences, particularly those below ten-fold without a more rigorous statistical comparison. Small potency changes (<3x) and dependent conclusions may evaporate when assay variability and error bars are taken into account. For example on line 224 the authors rationalise a lower potency of a para-fluoro analogue (5 uM) compared to a para-chloro analogue (2 uM) which is not supported without knowledge of error in these IC50 values.

E. Conclusions: robustness, validity, reliability

As discussed above, many of the smaller affinity differences (less than ten-fold) are likely not differences at all, rendering many of the conclusions and rationalisations questionable. That being said, the observations around structural changes that lead to greater than ten-fold changes in affinity are supported by many well-established concepts in medicinal chemistry, along with the effects of these changes on pharmacokinetic properties.

F. Suggested improvements: experiments, data for possible revision

Z'-values for assays should be included as well as a discussion of the error in the derived affinity values. Examination of the LE and LipE of the analogs should be included as well as analysis of the correlation between logP and affinity improvements. The conclusions of the paper would only be of interest if they shed new light on drug optimisation trends or strategies.

G. References: appropriate credit to previous work?

Young et al., J. Med. Chem., 2018, 61, 6421-6467 is cited (ref 34), along with other papers regarding ligand efficiency metrics, however only in the context of worsening PK properties with improvements in potency. The huge body of work on the strong links between physicochemical properties (especially lipophilicity) and their effect on the many axes of small molecule optimisation (potency, selectivity, toxicity, ADME, etc.) are not adequately referenced or considered in the manuscript.

H. Clarity and context: lucidity of abstract/summary, appropriateness of abstract, introduction and conclusions

Overall the paper is well written and the abstract and summary are appropriate. I've outlined my issues with the introduction and conclusions above. There are a few minor typos and inconsistencies:
• Sentence starting "Among" on line 90 doesn't make sense.
• Physchem props are not shown in in ED Table 2 as claimed.
• No color in ED Table 5 as claimed.
• Improvement in ADME needs to be explained better. What does 2-fold improvement in microsomal stability mean – half life, Clint, other? Same for the rest of the ADME.

Referee #4

(Remarks to the Author)
I co-reviewed this manuscript with one of the reviewers who provided the listed reports.

Version 1:

Reviewer comments:

Referee #1

(Remarks to the Author)
The authors have addressed all my concerns thoroughly - many thanks!

Derek Lowe

Referee #2

(Remarks to the Author)
I believe the authors have addressed my concerns. I am comfortable to agree for publication of this work.

Referee #3

(Remarks to the Author)
This revised manuscript is much improved. The authors have addressed all of my concerns regarding the original failure to account for physicochemical properties and assay variability when assessing differences between compounds. They have also adequately resolved Reviewer 2's concerns about GPCR pharmacology. I consider the manuscript acceptable for publication, subject to a few minor modifications and clarifications outlined below.

Page 5:

"18 parents cover a wide range of physico-chemical properties (Ext. Data Table 2)". I think they mean "Fig. 2" and they should add physico-chem data (at least Rof5 properties) below compounds in the figure to display the range of physico-chem spread.

"suggesting also a systematic strategy for optimization, even though that was not our primary goal." This sentence is a little unclear. Do they mean "suggesting also a strategy for optimization which includes making simple single-atom changes systematically, even though that was not our primary goal."?

Page 7:

"3H-rauwolscine or [^{3}H]CP-55940" "[3H]-citalopram". Normalise radioligand nomenclature.

Page 10:

"there was a strong linear correlation between both ligand efficiency and activity change and between lipophilic ligand efficiency and activity change—in both cases, substantially improved activity was accompanied by improved ligand and lipophilic efficiency." Maybe I'm being dumb, but the graphs in Ext. Data Fig. 4 don't obviously support the observation that "analogs that improved activity >10-fold had ligand efficiency of 1.4 for a single added atom (Ext. Data Fig. 4), close to the efficiency limit 31 (several even exceeded single atom ligand efficiencies of 2.0). Similarly, every analog that improved activity by >10-fold also improved lipophilic efficiency (Ext. Data Fig.4)." Can the data be presented differently to show this clearly?

In addition, it is at least odd and probably meaningless to calculate a correlation between terms which are dependent on each other.
LE = -1.4pIC50/N
LiPE = pIC50 - clogP

It would be better to show improvement beyond easy clogP gains on LiPE by using LiPE isoclines as shown on the LiPE wiki page https://en.wikipedia.org/wiki/Lipophilic_efficiency and something similar for LE.

Page 13:

“If many of the effects of the small perturbation analogs could be explained post hoc from their structures, however, fewer were easily anticipated.” Remove “If”.

Page 14:

“Meanwhile, in the complex between ‘4905 and the a2a adrenergic receptor, an added methyl group fits into a pre-existing sub-pocket and qualitatively its improved potency is reasonable. Quantitatively, its 52-fold effect on EC50 is at the outer edge of what might be expected by ligand-efficiency, especially since the methyl buries a serine hydroxyl. This is echoed by the free energy calculation, which here too correctly predicts improved activity, but only by 2- not 52-fold.” This is a really interesting observation and shows limitations of SBDD and FEP. Why no Ext. Data figure for this case?

Page 15:

“suggests that there may be merit in systematic small perturbations as a strategy, though showing that was not a goal of this study.” This is repeated near verbatim on p. 5 and I suggest removing here.

Page 16:

Why no mention of solubility and hERG in main text even though it’s been measured for 10-fold compound set in Ext. Data Fig. 7?

Page 17:

“This is born out on an atom-by-atom level: those atoms that most often improved affinity/potency substantially, like methyls and chloros (Figure 2a),” This is very pedantic but “moieties” or “groups” better than “atoms” as methyl is not an atom. Check for other similar uses throughout the text.

Page 20:

“experimental (grey) poses…” Looks more light blue than grey. Protein is grey.

Page 24:

“The individual in vitro pharmacokinetic terms were orthogonal to each other, and several were anti-correlated with affinity/potency improvement (Fig. 4).” It would be worth expanding here with a sentence and references on the known correlations between hydrophobicity and desired properties (target potency (e.g. LiPE)/permeability), and undesired properties (mic and plasma instability/low solubility/off-target potency (hERG)/low Fu) as this dichotomy is the fundamental challenge in lead optimisation.

DISCUSSION

I think it would be worth adding a few sentences on the limitations of docking and SBDD. The systematic approach in this study clearly highlights little correlation between structure, high level calculations, and potency and even some odd things like the ligand completely changing orientation with little effect on potency (Z8990523176 in Mac1). Summarising these examples and findings in the discussion will be a sobering fact for the cohort of AI ligand design groups who claim to be able to routinely use ML methods in ligand optimisation.

Referee #4

(Remarks to the Author)
I co-reviewed this manuscript with one of the reviewers who provided the listed reports.

Open Access This Peer Review File is licensed under a Creative Commons Attribution 4.0 International License, which permits use, sharing, adaptation, distribution and reproduction in any medium or format, as long as you give appropriate credit to the original author(s) and the source, provide a link to the Creative Commons license, and indicate if changes were made.
In cases where reviewers are anonymous, credit should be given to 'Anonymous Referee' and the source.
The images or other third party material in this Peer Review File are included in the article's Creative Commons license, unless indicated otherwise in a credit line to the material. If material is not included in the article's Creative Commons license and your intended use is not permitted by statutory regulation or exceeds the permitted use, you will need to obtain permission directly from the copyright holder.
To view a copy of this license, visit https://creativecommons.org/licenses/by/4.0/

Reviewer 1
Reviewer 1 was enthusiastic, noting that they "*enjoyed this paper a great deal!"* We are grateful for their support and the thoughtful suggestions they made to improve it:

1.1. They caught several errors in our references: "*reference 45 should be (https://doi.org/10.1002/anie.201303207)... the Chiodi et al. "Magic Chloro" paper really needs to be cited (https://pubs.acs.org/doi/abs/10.1021/acs.jmedchem.2c02015).. consider adding a citation to the Cramer et al. paper on hydroxy substitution in medicinal chemistry (https://pubs.acs.org/doi/abs/10.1021/acs.jmedchem.9b00179)... (and on) adding fluorines to (https://pubs.acs.org/doi/abs/10.1021/acs.jmedchem.9b00179).*

We regret these oversights and now cite all four papers.

1.2 (line 41). Referring to our statement that thousands of analogs can be made in a hit-to-candidate campaign, they note, "*The "thousands" figure shows up a lot…but I have only been involved in one project in the past 35 years that synthesized that many analogs. However, that shows it is possible, so this statement (which reappears in line 64) can stand…*

We thank the Reviewer for saving us from overstatement; we have revised this to read "hundreds, occasionally thousands of analogs…"

1.3. (line 115-16): The Reviewer worries that we come on too strong when we write that our small perturbations were, "…systematically and comprehensively applied without design, ensuring an unbiased approach," noting that, *It's true that these are applied without design, but there is a bias towards synthetic tractability…That may seem like a nit-picking point, but it's one of the systematic biases in the synthetic chemistry literature that has made machine-learning approaches across it very difficult."*

This too is well-taken. We now write, "…systematically and comprehensively applied without design, ensuring an approach unbiased by anything other than synthetic accessibility and price (which we admit are substantial constraints, as we make clear below)."

1.4 (line 155). Referring to "…while 41.7% reduced activity by >10-fold…", *I was waiting and hoping to see how many of these reduced potency! This is about the ratio of harm/help that I would have predicted. After all, you're starting with molecules that are already…at the tip of the spear, so…any changes have a greater chance of being for the worse.*

We share the Reviewer's intuition here. As their interest in this point will be shared by others, we have added a new panel **C** to **Ext Data Figure 1** that reports the fraction of analogs that *reduce* potency by >10x on a parent-by-parent basis.

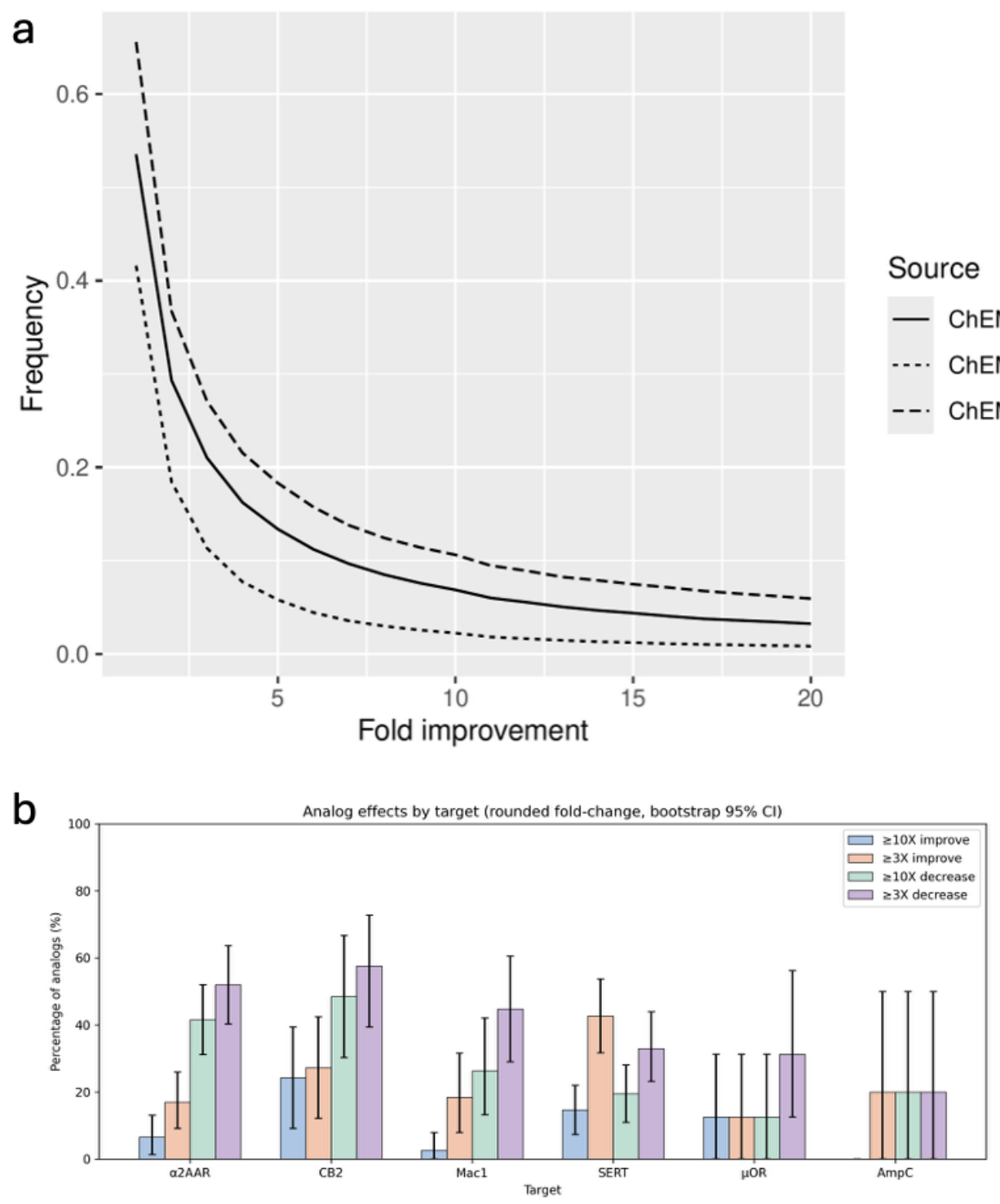

c

Parent	target	n_analogs	n_worse_3x	n_worse_10x
Z1039063794	mac1	12	11	9
Z6969215903	cb2	6	4	3
Z4376630014	alpha2	10	6	5
Z2750653629	alpha2	31	15	12
Z2573292509	sert	9	4	3
Z2573292480	sert	11	4	2
Z2009978218	sert	14	5	2
Z4407498716	MOR	16	5	2
Z5398393122	mac1	5	1	0
Z7534253453	mac1	22	3	0
Z3034773248	alpha2	18	2	1
Z8731642686	sert	19	2	0
Z8184698918	cb2	20	2	0
Z8727395870	alpha2	18	1	1
Z6971277399	sert	15	0	0
Z8727393896	sert	14	0	0
Z2610488449	ampc	10	0	0
Z52076138	cb2	7	0	0
ALL	ALL	257	65	40

Extended Data Fig. 1 Frequency of activity changes from single-atom substitutions in ChEMBL and in this study. **a,** Cumulative frequency of single non-hydrogen atom substitutions (CH_3, F, Cl, Br, N, OH) in ChEMBL that improve activity, stratified by that of the parent compound. “Potent” denotes parents with activity ≤32 nM, “mid” 32 nM–1 μM, and “weak” 1 μM–1 mM. **b,** For each target in this study, the percentage of analogs that improve or decrease activity by ≥3-fold or ≥10-fold versus their parent. Fold changes were rounded to the nearest integer prior to thresholding (e.g., 9.6–9.9 counted as 10-fold). Error bars denote 95% bootstrap confidence intervals (percentile method; 20,000 resamples). Compounds yielding no measurable curves were counted as ≥10-fold decreases. **c**, Parent-level summary of analog effects in this study (rounded fold-change).

1.5 (line 166). *“The “magic methyl effect” gets some more empirical confirmation!”.*

We also thought it a cool result and for us a bit of a surprise. For us, “magic” methyl has meant an unexpectedly large activity improvement on methyl addition. But if over 20% of methyls do this then perhaps it isn’t so “magic”, perhaps it is predictable and expected (same for chloros).

1.7 (line 170). “*Interesting comparison with F. Most of the time… medicinal chemists (use) fluorine for metabolic stability, and these smaller changes in potency are in line with my own experience….*

Good point, we now write, “Conversely, fluorine substitutions, *which are often used to increase metabolic stability*, yielded no $\geq$10-fold and few 3-fold improvements in activity” (italics added).

1.8 (line 178): Regarding our analysis of what, if any, properties of the parent or the binding site might correlate with activity improvement, the Reviewer wrote, “*If these questions hadn’t been raised explicitly in the paper, I was going to insist on them myself! Glad to see this analysis*.”

We are grateful for their support. Responding to a critique of Reviewer 3, we also added further statistics to this analysis, giving confidence limits to the trends.

1.9 (line 231). About our observation of ligand binding mode change on small perturbations, and their likely under-reporting in the PDB, the Reviewer wrote, "*I am absolutely certain that this point is correct, and I'm glad to see it made.*"

Here too, we are grateful for the support. Initially we weren't sure if we should draw attention to that, but we are happy we did and share the Reviewer's confidence.

1.10 (line 250-257). *Were there any analogs that showed less favorable PK? The biggest possibility I can see would be adding a phenol to an aromatic core.*

We now discuss this point, adding a new figure. We write,

> "We also observed a substantial fraction of analogs with ≥3-fold decreases in these same properties (**Extended Data Figure 6**). Considering what are perhaps the three most impactful in vitro PK properties, microsomal stability, fraction unbound, and permeability, 36% of the analogs suffered a >3-fold loss in one or more of them, almost all (96%) suffered at least some deterioration by the same criterion, and 37% deteriorated in all three in vitro PK properties."

a

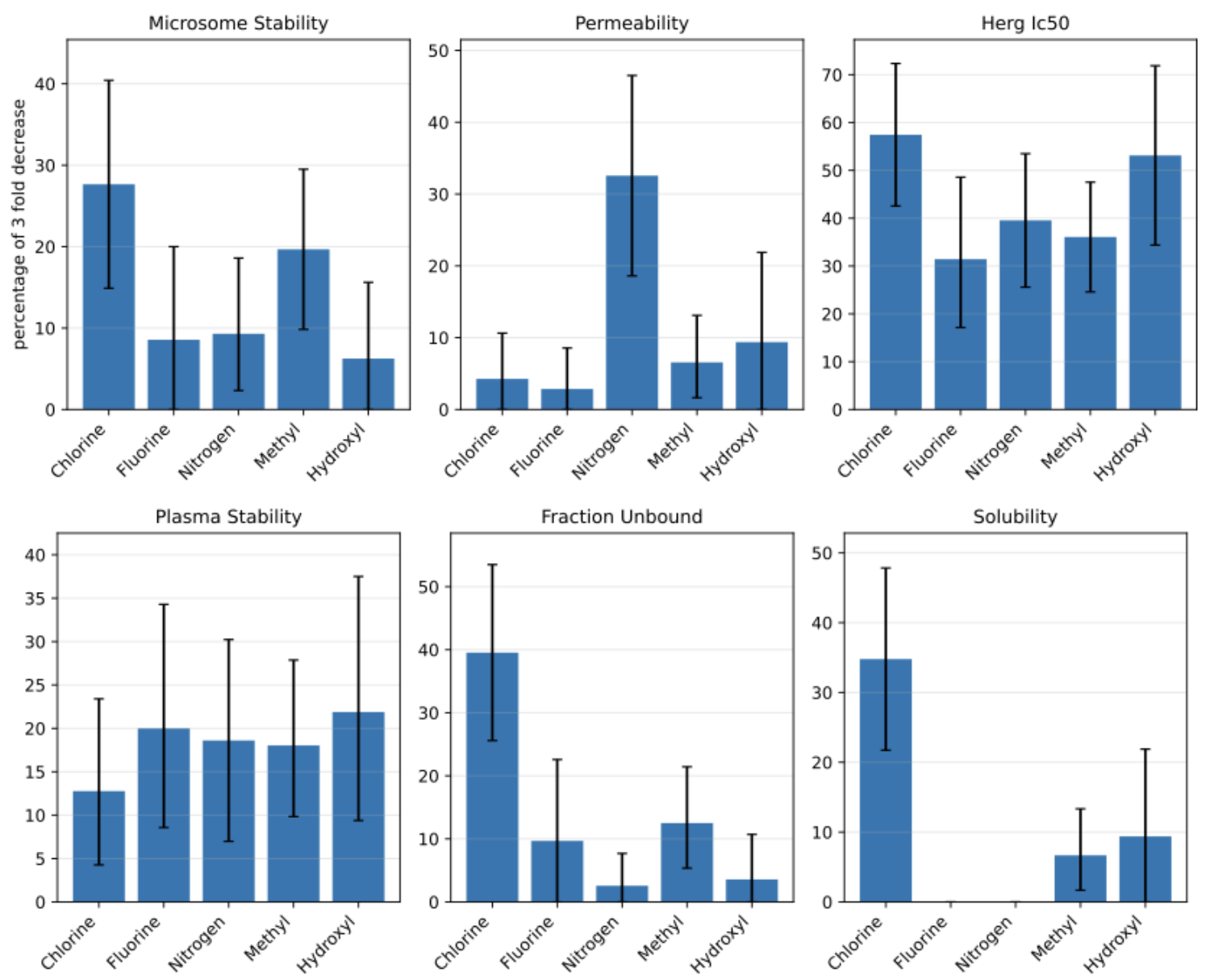

b

Parameter	Analogs	Measured	>3x loss	>10x loss
Microsomal stability t1/2 (min)	257	226	35	10
Plasma stability t1/2 (min)	257	226	39	10
Fraction unbound (% unbound)	257	199	35	11
Permeability (cm/s)	257	226	24	11
Solubility (μM)	257	224	23	8
hERG IC50 (μM)	257	226	43	14

Extended Data Fig. 6 Single-atom substitutions frequently worsen PK properties **a.** Fraction of single-atom substitutions with ≥3-fold decreases in PK properties. Error bars represent 95% confidence intervals, estimated from 10,000 iterations of bootstrap resampling.**b**. Counts of analogs with measured PK parameters and >3-fold or >10-fold losses.

To the aromatic hydroxyl question, these didn't stand out as particularly harmful, indeed after aromatic nitrogens (C→N), addition of a hydroxyl, often a phenol, was the most likely perturbation to *improve* in vitro PK, *including microsomal stability*, except permeability. This

must be taken with a grain of salt, however, because we used liver microsomes, not hepatocytes, in which phenols might have undergone secondary metabolism. We now note this in our caveats paragraph in the Discussion.

1.11 (Lines 259+). About the trade-off between affinity and PK properties, they write, "*It's good to see this laid out so openly - medicinal chemists generally discover this, but for some reason people seem reluctant to say it.*"

Thank you. We agree this is tacit knowledge among expert teams, but to others it may come as a surprise, and perhaps even to experts the quantification will be useful.

1.12 (Lines 306+). *I would sum this up as "FEP calculations are often most accurate in the situations where you don't need them as much".*

Ha!

1.13 (Lines 330+). *This seems particularly relevant given the current FDA leadership's stated desires to do more through calculation/AI/ML and less by experiment, which personally I think is extremely ill-advised.*.

No kidding. We'd add a comment to this effect but we want to be careful; for while we recruited domain experts to undertake the FEP calculations, the predictive PK was done by us using web-based tools. Experts may have had better results and we don't want to tar a field (predictive ADME) in which we are not expert.

1.14 (line 407). *To me, the two halves of this sentence don't quite line up - the following sentence mitigates this appropriately, but I'd like to see this reworded to stop people from reading the first one and walking away…*

This is well-taken. We have rewritten this sentence to read, "**Third**, computational prediction of affinity and PK had some success anticipating the trends we observed (Figure 5) implying that they may eventually guide compound selection." The added word "*eventually*" does the hard cautionary work here; we hope it's sufficient.

1.15 (line 411). *If it weren't possible, the drug industry wouldn't exist...*
For sure. But we wanted to demonstrate it within this set of 257 analogs, which spoke to relevance to the field and also that the effects of improved EC50, corrected for changes in PK, translated in vivo; at least for the alpha2a agonist, '4905, they do—it is the most potent analgesic we've discovered against this target, and far better than its parent.

1.16 (line 433). Speaking to our characterization of the old tag "methyl, ethyl, butyl, futile"*, the Reviewer writes, "*I've come around to wearing it like a badge of honor for when we can overcome the problem!*".

Same!

<u>**Reviewer 2:**</u>
Reviewer 2 also had kind things to say about this study ("…*a compelling and timely research question*…"), for which we are grateful. They also had substantial critiques, sketched at the beginning of this letter, which we have taken to heart. We take each of their points in order.

2.1.a (affinity and potency) *A major limitation significantly affects the study's reliability and*

interpretability: the interchangeable use of … affinity and potency throughout…. These are distinct pharmacological concepts.... As a result, the manuscript frequently shifts between discussing affinity and potency—sometimes within the same sentence or paragraph—without clear distinction. This is problematic, as these parameters are not interchangeable and should not be pooled as a single global measure of "activity." Affinity does not always correlate directly with potency. In systems with high target expression, changes in potency may reflect changes in affinity, efficacy, or both—but without additional mechanistic data, it is impossible to determine the relative contribution of each. Only in systems with low target expression can shifts in potency (affinity) and efficacy (maximal response) be more reliably distinguished in functional assays. However, the manuscript does not clearly report the expression levels of the various biological targets, which further complicates interpretation.

Two points are raised: first the conflation of affinity and potency, and second the impact of receptor expression on ΔEC_{50}s between parents and analogs, which is crucial for this study. ***Both are well taken***. We begin with the conflation of affinity and potency.

We have unified our activity readouts and re-measured selected compounds where warranted; the updated values are now reported in the **SI Table S1**, where we have added a column indicating for each entry whether the assay readout is K_i, EC_{50}, or IC_{50}. We also have added a column that identifies whether we are reporting K_is, IC_{50}s, or EC_{50}s for each parent/analog pair to main text Table 1. We also note where the concentration-response values for these pairs may be found (**SI Figure 2**), so that the reader can see for themselves whether they reliably reflect changes in the half-maximal response.

Importantly, we no longer mix K_i and EC_{50}/IC_{50} within the same system. For SERT and for AmpC, we report only K_i values, for Mac1, all measurements are IC_{50}. For GPCR targets, in line with common pharmacological practice, we use EC_{50} when the parent compound is an agonist and Ki when the parent compound is an antagonist to better reflect their physiological mode of action (and reflecting the biophysics of G protein coupling, which naturally left-shifts activity values, but typically does so in tandem for an agonist and its agonist analogs). Thus, because the MOR parents are all agonists, the entire MOR series is reported as EC_{50}s, whereas for CB2 and α2A, which include both agonist and antagonist parents, some series are reported as EC_{50}s and others as K_is, but in the revised manuscript we never report mixed readouts within the same parent series. As mentioned, this is now reflected in both main text Table 1 and in SI Table 1.

Finally, we have added text to the beginning of the Results and in the penultimate paragraph of the Discussion to draw the reader's attention to this distinction. In the Results we write (p.7):

> For every parent, and for most receptors overall, we only report changes for one type of activity, i.e., if a parent K_i is reported than only K_i values are reported for its analogs, and if a parent is an agonist, only EC_{50}s are reported for its analogs. Each functional or inhibition assay is done in reference to a literature positive control molecule (Supplementary Figure2). Representative concentration–response curves and the corresponding Z values for each assay are provided in **Supplementary Fig. 3**. Especially for agonists, the effect of receptor expression is controlled for (**Supplementary Figure 4 and 5**). Because changes in agonist efficacy can sometimes affect interpretation of EC_{50}, we inspect efficacy values to ensure that they are similar between parent and analog; full concentration-response curves and analyses are show for every parent-analog pair, allowing for direct inspection of these results.

And in the Discussion (p.26):

While ligand competition is highly correlated with K_i, agonist EC_{50}s are affected by receptor expression. Because we always compared the relative activities of analogs versus their parents, controlled for receptor expression, and measured effects in a low expression level domain (**Supplementary Figure 5**), the effects of receptor expression on relative activity should be modest. Still, they cannot be completely discounted. For these and related reasons, changes of EC_{50} between agonist parent and analogs cannot be read as changes in affinity the way that changes in K_i can be, though EC_{50} changes remain the relevant metric for agonist activity. Overall, the impact of these effects may be inspected case by case in full concentration-response for any parent ligand pair in activity (**Supplementary Figure 2**).

2.1.b (expression level). Turning to the Reviewer's concern about receptor expression, we now show that our functional assays were run under low-expression conditions. For the three GPCRs, α2A, CB2, and MOR, we quantified relative receptor expression by antibody staining and flow cytometry, obtaining normalized levels of 100, 35.9, and 14.9, respectively (**Supplementary Figure 4**). For α2A we also performed radioligand saturation binding, which yielded Bmax values of 870.7–1322 (≈1.8 pmol/mg), consistent with a low receptor density. Both results indicate that the GPCR assays were conducted in the low-expression regime. For instance, in Manglik et al, *Nature* 2016 (doi:10.1038/nature19112), the authors reported a μOR expression level by saturation binding with [3H]-diprenorphine with a Bmax of 4–6.5 pmol/mg protein, about 3-fold higher than ours. In their GloSensor assay they co-transfected at μOR:biosensor ratio of 1:1. Conversely, we used a ratio of 1:3, a lower fraction of receptor plasmid under comparable transfection conditions, which should yield lower receptor expression. Compared with Fink et al., *Science* 2022 (https://doi.org/10.1126/science.abn7065) for assaying α2A, which used 200 ng receptor plasmid; 1 µg total DNA per 3 × 10^5 cells, we only transfected 100 ng receptor plasmid per 96-well plate. In this regime changes in potency should more closely track changes in apparent affinity and to be physiologically relevant. Still, we now explicitly state in the Discussion that we do not interpret potency shifts as pure affinity, which we agree is important to make transparent (see quoted text in **2.1.a**, above).

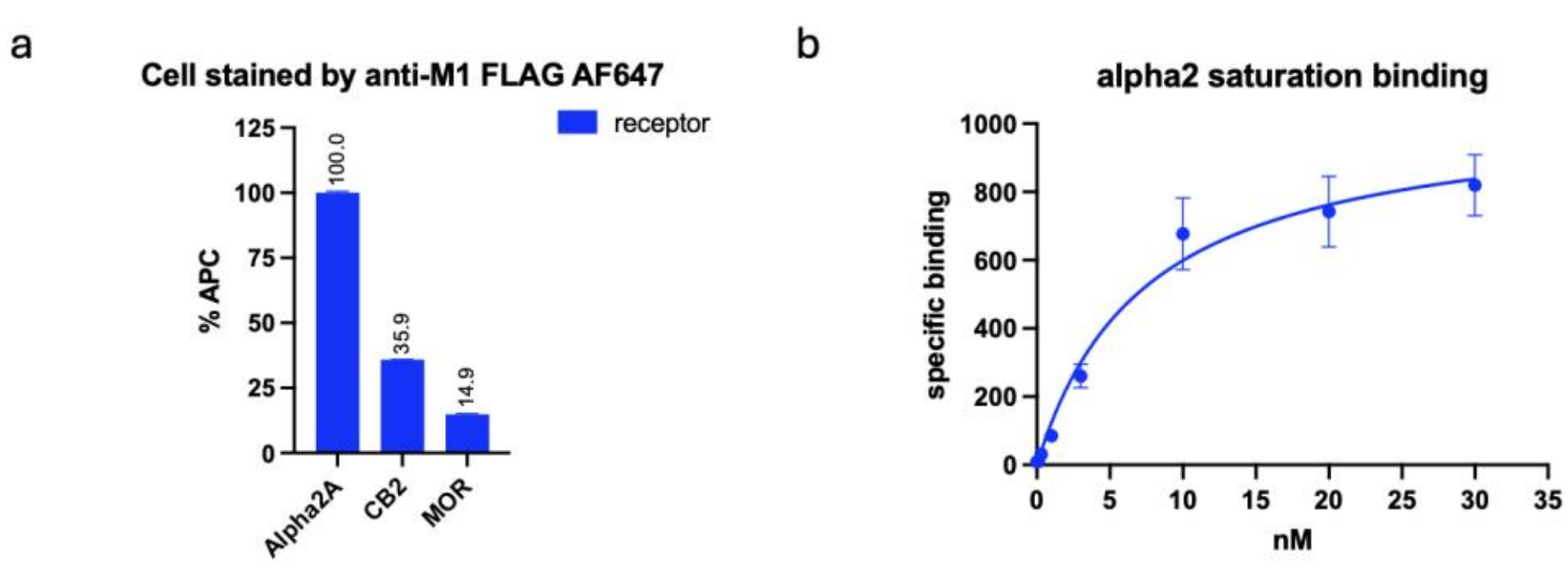

96 plate	Plasmid	Expression
Alpha2A	100ng	100
CB2	300ng	35.9
MOR	600ng	14.9

Best-fit values	
Bmax	1046
Kd	7.466
95% CI (profile likelihood)	
**Bmax**	**870.7 to 1322**
Kd	4.216 to 14.00

SI Figure 4. Receptor expression levels for α2A, CB2, and MOR (relative and absolute measurements). **a.** Relative quantification of surface receptor expression for α2A, CB2, and MOR by flow cytometry using anti-M1 FLAG–AF647 staining (normalized to α2A = 100%). **b.** Absolute quantification of α2 receptor expression by radioligand saturation binding, reporting best-fit Bmax and Kd (with 95% confidence intervals).

Taking this one step further, we systematically examined how receptor density affects functional EC_{50} values. Using the DNA amount employed in the main study as a reference (**SI Figure 4** above), we titrated the transfection both up and down and tested a set of well-characterized ligands as controls (**SI Figure 5**). At lower expression levels we observed *larger* fold-differences in EC_{50} between parents and analogs, suggesting that the expression levels we used do not inflate EC_{50} differences between parents and analogs; if anything, they may understate them—the relative effects we measure seem if anything conservative. More broadly, we are partly insulated from expression effects by our focus on relative differences. Naturally, even these can be affected by expression, but we hope our further controls have allayed some of these concerns. Additionally, to test whether overexpression inflates fold-changes, we compared binding vs functional readouts across our 256 analogs. Large effects were more common in Ki than in EC_{50}/IC_{50} (Ki: ≥3× 46/131**,** 35.1%**;** ≥10× 19/131**,** 14.5%**;** EC_{50}/IC_{50}: ≥3× 23/126**,** 18.3%**;** ≥10× 10/126, 7.9%), arguing that our functional assays do not exaggerate differences and, if anything, are conservative.

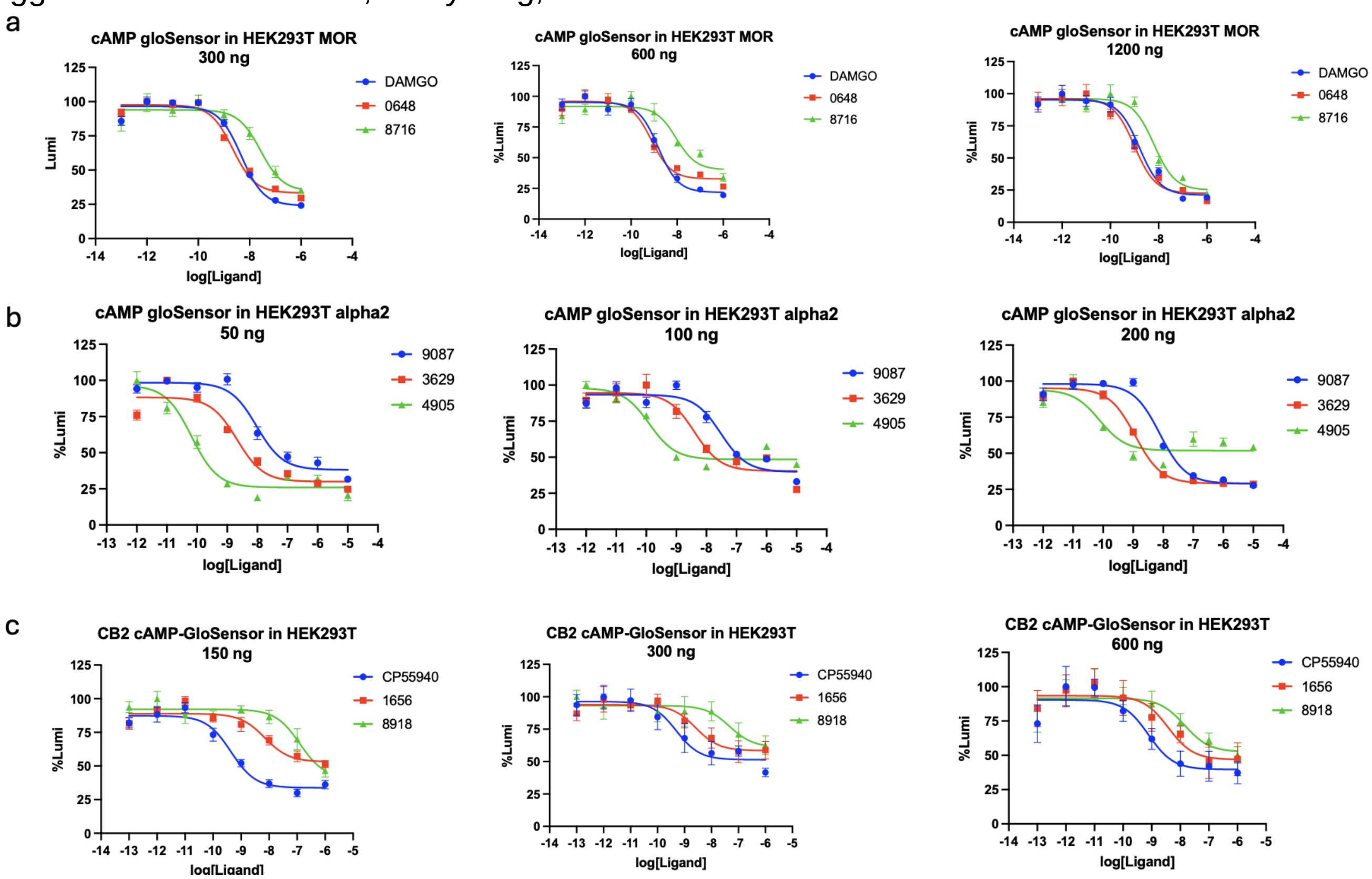

Supplementary Figure 5. cAMP responses at different receptor expression levels **a**. MOR cAMP GloSensor concentration–response curves measured in HEK293T cells transfected with increasing amounts of MOR plasmid (300, 600, and 1200 ng), comparing DAMGO and two test ligands. **b**. α2AR cAMP GloSensor concentration–response curves measured at increasing receptor plasmid amounts (50, 100, and 200 ng), comparing 9087 and two test ligands. **c**. CB2 cAMP GloSensor concentration–response curves measured at increasing receptor plasmid amounts (150, 300, and 600 ng), comparing CP55940 and two test ligands. Points show mean ± error (as plotted) with fitted dose–response curves.

2.2. Writing further on the role of expression, the Reviewer writes, "*This ambiguity limits the robustness of the conclusions and weakens the generalizability of the findings. For example, depending on the system, a single chemical modification could alter affinity without affecting potency (only affecting maximal response), or vice versa. Has the team observed cases where a compound affected efficacy (… change in maximal response) without altering potency?*

Contrastingly, have there been instances where a potency shift was misinterpreted as a change in affinity, when in fact it was driven by efficacy and system-specific factors (e.g., receptor overexpression)? Has any well-known strong agonist used in parallel to support the robustness of the study? When both potency and affinity have be quantified, which one is reported and used for interpretation? These considerations are essential for accurate interpretation and … SAR analysis, and in my view are unclear at this stage.

These points are well taken and clarifying them has strengthened the manuscript. Taking the questions in order:

i. We have seen few cases where an analog changed maximal response without altering potency, though that is possible. More crucially we have seen no analog that led to a change in efficacy that was misinterpreted as a change in EC_{50}. Our focus has been on those analogs that strongly affect EC_{50} while leaving efficacy relatively unaffected, and this is essentially all those that we report. The full set of concentration response curves is now included in **Supplementary Figure 2**. We also provide 95% confidence intervals and p-values for parent–analog differences (**Supplementary Table 1**), computed using a two-sided Wald (z) test on $\log_{10}(EC_{50}/IC_{50}/K_i)$ with standard errors derived from the confidence intervals, enabling readers to interrogate the data for these effects.

ii. The question about using control molecules ("*any strong agonist used in parallel*") is a good one and we regret omitting these results (which we had measured but hadn't included). In the revision we show control molecules (Amitriptypine for SERT, DAMGO for MOR, '9087 for α2a, and CP-55940 for CB2, ADPr for MAC1) for every parent-analog pair (**Supplementary Figure 2).** As noted, for each parent/analog series we report either affinity or functional activity, but not a mix of both: following common practice, we use EC_{50} for receptor agonist parents and Ki for receptor antagonist parents (for the enzyme Mac1 we use IC_{50} measured at a constant concentration of displaceable peptide). Thus, within any given parent series there is no selective choice of which parameter to report and interpret.

2.3. *This misuse of affinity vs potency is found throughout the whole manuscript. Here is listed a sample of the places where affinity and potency have been used…:*
- Table 1: only EC50 are mentioned—why not affinity? and if measuring both, why not presenting both?
- Fig. 2a: only affinity is mentioned … why not potency?
- Fig. 3: when stating "structural basis of affinity changes…" is this only for the biological targets for which affinity estimates where quantified?
- Fig. 4 legends: this time the term potency is used; was this only for the biological targets where potency where measured?

We agree, and in the revised manuscript we have systematically corrected all of the instances mentioned (Table 1, Figs. 2–4), no longer using affinity and potency interchangeably. Instead, we refer to the neutral term "activity readout ($K_i/EC_{50}/IC_{50}$)" and specify for each target in the Table and Figure legends whether the underlying measure is K_i, EC_{50}, or IC_{50}.

2.4. Reviewer 2's second overarching critique concerned the statistics of the behavioral pharmacology of parent α2a agonist '3629 and its 50-fold more potent analog, '4905:

"Another major limitation … is the lack a clarity in …Fig. 6c-e. It is unspecified if the authors performed power calculation and determined the number of animals for their study. There is no associated table that present the data as mean +/- error and animal number …. Also, what

type of statistical analysis and post-hoc analysis was performed to present significance? Without the summarising table of data, it is unclear how dosing might be significantly different from vehicle. In particular, 4905 at 5 mg/kg, is pretty much at the same level as vehicle, but yet significantly higher? Similar comment for PS75 at 2.5 and 5 mg/kg? These outcomes seem relatively unlikely, but without the table summarising the data, it is impossible to assess. Some group for dosing only had 5 animals; is that sufficient? Have the authors been reusing the animals from one assay to the other after a wash-out period? In figure 6d, the y-axis stipulates "mechanical threshold (/baseline). It is unclear how the baseline can be at 100% then?

These are fair points. To address them, we have: **first,** added supplementary table (**Supplementary Table 12,** and below) reporting, for each assay and dose group, the number of animals, mean, standard deviation, and the statistical test and analysis used; this makes the basis of the significant effects in **Fig. 6c–e** explicit. **Second**, we have split the original **Fig. 6c** into three panels (now **Fig. 6c–e**), making the dose-dependent differences easier to see and reduces confusion. We did not perform formal power calculations; instead, we based our group sizes on our previous extensive behavioral studies and on published work using similar approaches, which have been sufficient to detect significant effects, and we now acknowledge this in the Methods, writing:

> "Behavioral data are summarized as group size (n), mean, and SD (SI Table 12). For the SNI experiment, we used two-way ANOVA followed by Tukey's post-hoc multiple comparisons. For the hotplate assay, we used one-way repeated-measures ANOVA with Friedman post-hoc testing. For tail-flick, the 4905 dose–response was analyzed by two-way ANOVA with Dunnett's post-hoc comparisons to vehicle/control, whereas PS75 and 3629 dose groups were analyzed by one-way ANOVA with Kruskal–Wallis post-hoc testing (SI Table X).For sample size, we did not perform formal a priori power calculations. Instead, group sizes were guided by our prior experience with these assays and by precedent in the literature. This approach may limit sensitivity to small effects.".

For the dose–response analyses and SNI experiments, some mice were re-used: animals received up to three injections (including vehicle), with a minimum wash-out period of 3 days between injections; this has been added to the Methods. Finally, for panel **d** the data were normalized to each animal's own baseline, which is why the baseline appears at 100%; we now state this explicitly in the figure legend.

Assay	Cpds	Concentration	Animal Number	Mean	SD	Analysis
SNI	baseline		10	100	55.46	2way ANOVA with Tukey's
	SNI		10	17.08	21.68	
	SNI + vehicle		12	42.28	33.38	
	SNI + 4905	5 mg/kg	5	138.96	56.04	
	SNI + 3629	5 mg/kg	5	42.46	46.27	
	SNI + PS75	5 mg/kg	5	118.02	43.48	
Hotplate	Vehicle		10	12.07	3.64	One-way ANOVA (repeated measures) with Friedman post-hoc
	3629	5 mg/kg	10	18.9	3.30	
	4905		10	26.21	6.88	
Tail flick	Baseline		10	2.65	0.24	2way ANOVA with Dunnett's post-hoc
	Vehicle		10	2.96	0.40	
	4905	0.5 mg/kg	5	2.60	0.43	
	4905	1 mg/kg	5	3.08	0.37	
	4905	2.5 mg/kg	10	3.41	0.36	
	4905	5 mg/kg	10	5.33	1.67	
	Vehicle<br>PS75	 2.5 mg/kg	10<br>5	2.84<br>3.79	0.42<br>0.64	One-way ANOVA with Kruskal-Wallis post-hoc
	PS75	5 mg/kg	10	3.99	1.03	
	Vehicle<br>3629	 2.5 mg/kg	10<br>5	2.63<br>2.96	0.43<br>0.52	
	3629	5 mg/kg	5	3.63	0.77	
	3629	10 mg/mg	5	3.52	0.39	

Supplementary Table 12: Summary statistics and analyses for in vivo analgesia assays

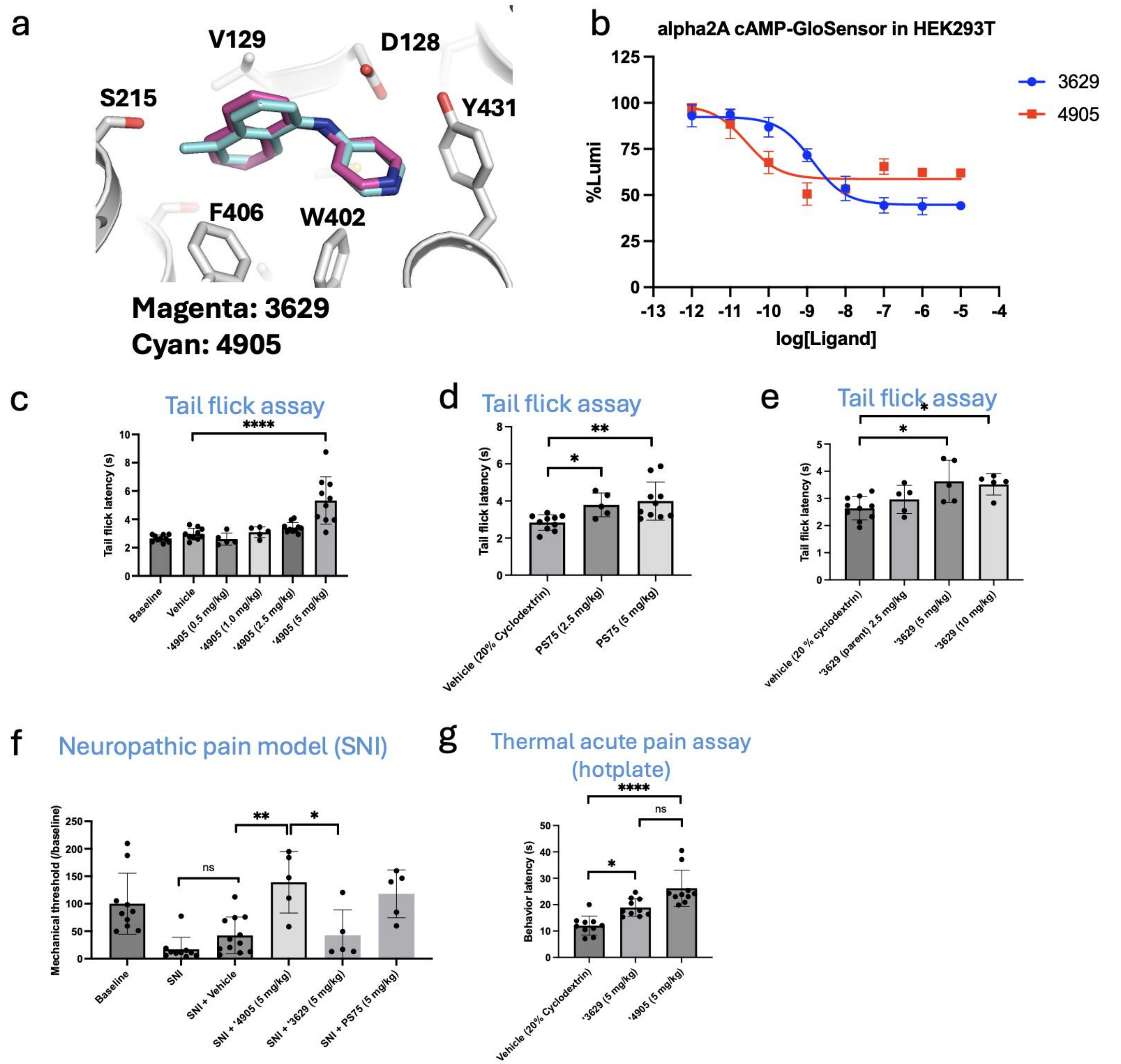

Revised Figure 6: A single-atom addition to the alpha2a receptor parent agonist leads to analog '4905 which is a more potent analgesic in vivo

Minor limitations.

2.4.1 *How were the 18 parent molecules selected? Why these and not others? What was the rationale for selecting 3 class A GPCRs (no class B, or C or others?), 1 transporter, and 2 enzymes? Why selecting only 1 parent for mOR, whilst in contrast the authors selected 4 parents for a2A, or 6 for SERT? Can these bias in some way the "randomness" of the background method intended?*

We agree that the possible, if unintended, biases innate in any restricted sense such as this one merit more emphasis. We now write, in the Introduction (p. 4-5):

> To create what we will refer to as a random background set for ligand optimization, we chose 18 parent ligands spanning six targets, including three GPCRs (alpha2A adrenergic receptor (α2a), μ-

> opioid receptor (MOR) and cannabinoid receptor type 2 (CB2)), one transporter (the serotonin transporter, SERT) and two soluble enzymes (AmpC β-lactamase (AmpC), and Macrodomain 1 of SARS-CoV-2 (Mac1)). This set was chosen for targets that we had under experimental control and is admittedly far from comprehensive: we explore only class A GPCRs, no ion channels nor kinases are represented, only one transporter, and only two of almost innumberable soluble enzymes. The 18 parent compounds were chosen for similar pragmatic reasons—they were molecules to which we had ready access. Still, these proteins provide a range of target classes, while the 18 parents cover a wide range of physico-chemical features (**Extended Data Table 2**) and a wide range of potency values, from 1 nM to 214.9 μM (**Table 1**). For each of the 18 parent parents, most of which were topologically unrelated…"

Similarly, in the penultimate (Caveats) paragraph of the Discussion, we now write:

> At 257 analogs and six targets, our set remains small and inevitably, if unintentionally, biased. We do not pretend that three class A GPCRs, one transporter, and two soluble enzymes can adequately express pharmacologically interesting target space, nor that the 18 parent ligands can adequately represent ligand space. Expanding the analysis to additional molecular targets and ligand classes might reveal different trends. We note that the six targets do recognize a range of chemotypes, including large polar co-factors (Mac1), large hydrophobic lipids (CB2), anionic β-lactams (AmpC), cationic neurotransmitters (α2a and SERT), and peptides (μOR).

2.4.2 *Another major concern is the "unbiased" nature of the study. I appreciate the selection of possible single chemical modifications present in Fig. 1a (middle panel), but is there a rationale for not including other simple single atom changes such as N-->C, N-->O, N-->S, O-->S? Would this influence the nature of the random background?*

There are two reasons for our choice of these particular small changes. First, they are ones typically used by medicinal chemists. Here, we modeled our set on that used by Muegge et al., whose strategy, though addressed to a different question, we largely adopted (Pennington, et al & Muegge. Positional Analogue Scanning: An Effective Strategy for Multiparameter Optimization in Drug Design. *J Med Chem* **63**, 8956-8976 (2020)). Second, some of the perturbations aired by the Reviewer would change valence and sometimes charge, making them major perturbations (N→O and N→S). Finally, many medicinal chemists are disinclined to add sulfur to molecules because of oxidative liabilities. We write on p.4 of the Introduction:

> Akin to alanine scanning, an unbiased background for ligand optimization might involve conservative perturbations, systematically and comprehensively applied across multiple unrelated parents across several different targets. Muegge and colleagues have described a "positional analog scanning" approach, which substitutes ligand non-polar hydrogens (C—Hs) with groups like CH_3, OH, Cl, F, and Br, and aromatic carbon atoms with nitrogen[21]

2.4.3. *Why a mix of binding and/or functional assays for the selected biological targets? There is no obvious rationale for why using only a functional assay for mOR, AmpC, only binding assay for sert and Mac1, and both binding and functional assays for a2A and CB2. Nowhere in the manuscript the affinity estimates appear to be reported (only values are EC50).*

This point is well-taken, and we hope we largely addressed it in our earlier responses. We now use a single, consistent activity readout per parent ligand: for SERT and AmpC, all measurements are K_i. for Mac1, all measurements are IC_{50} (we do not use Ki for Mac1 because a reliable and scalable activity assay, reflecting ADP-ribosyl hydrolysis, has not been developed for it; instead we use peptide displacement, which is quantitative and reliable). For the three GPCRs we use EC_{50} for agonist parents and K_i for antagonist parents, in line with common pharmacological practice. Thus MOR (only agonist parents) is reported entirely as EC_{50}, whereas α2A and CB2 include both agonist and antagonist parents, so some parent

series are reported as EC_{50} and others as K_i, but we never mix readouts within the same parent series. We now state this rationale explicitly in the Methods, writing,

> "**Assay selection.** We use one consistent readout per parent series (no mixing within a series). SERT and AmpC are reported as Ki; Mac1 is reported as IC50 from peptide displacement (a scalable functional hydrolysis assay is unavailable); GPCR agonist series are reported as EC50 and antagonist series as Ki, such that MOR is EC50-only whereas α2A and CB2 include EC50 and Ki depending on the parent series.".

2.4.4. *Table 1 needs to clearly present which parameters (potency or affinity or both) in the column titles*. We have updated Table 1 with a new column that reflects these parameters.

Table 1. Analogs from all parents improving affinity/EC50 by ≥3x or ≥10x

Protein	Assay readout	Parent Ligand	Parent Affinity/EC50	Total Analogs	Analogs with ≥3X Affinity/EC_{50} improvement	Analogs with ≥10X Affinity/EC_{50} improvement
MOR	EC50	Z4407498716	9.2 nM	16	2	2
alpha2	EC50	Z2750653629	1.4 nM	31	7	4
alpha2	Ki	Z3034773248	22.1 µM	18	3	1
alpha2	EC50	Z4376630014	8.3 nM	10	2	0
alpha2	Ki	Z8727395870	13 µM	18	1	0
AmpC	Ki	Z2610488449	24.2 µM	10	2	0
cb2	Ki	Z52076138	2.3 µM	7	7	6
cb2	Ki	Z6969215903	0.4 µM	6	0	0
cb2	EC50	Z8184698918	0.1 µM	20	2	2
Mac1	IC50	Z5398393122	2.2 µM	5	0	0
Mac1	IC50	Z1039063794	0.7 µM	12	0	0
Mac1	IC50	Z7534253453	8.9 µM	22	8	2
SERT	Ki	Z2009978218	1.1 µM	14	4	0
SERT	Ki	Z2573292480	0.08 µM	11	1	0
SERT	Ki	Z2573292509	0.2 µM	9	2	1
SERT	Ki	Z6971277399	13.4 µM	15	10	4
SERT	Ki	Z8727393896	42.7 µM	14	8	3
SERT	Ki	Z8731642686	1.8 µM	19	10	4
Total		All Parents		257	69	29

2.4.5. *Ext. Data Table 1 is apparently missing 67 analogs … What are all the parameters presented in column titles, i.e., exp_dg, fep_dg, fep_unc, fep_ddg, fep_dg_err_abs and fep_ddg_err_abs? As well, only EC50 are reported, not affinity, but for at least 2 targets, only affinity estimated were measured … Additionally, it is unclear which atom was modified in ED Table 1. Although there is information about the replacement atom type (Cl, N, F, …), the position of the atom itself is missing.*

The missing 67 analogs correspond to compounds for which we did not initially obtain a reliable full concentration–response curve. These analogs either showed weak or no effect in single-point screening, or produced curves that did not allow a robust fit and thus could not be

meaningfully compared to FEP predictions. In the revised manuscript, we have returned to these compounds and re-measured full DRCs wherever possible, adding another 30 compounds to the FEP comparisons; these have been added to Extended Data Table 1. In total, 220 compounds now have complete dose–response measurements, while 37 small molecules still lack measurable affinity or potency estimates and are therefore not included in the FEP comparison table.

We have added footnotes to explain the column parameters and adjust the column title to read EC_{50} or affinity, depending on the assay type. In Extended Data Table 1 we only report the replacement atom type (Cl, N, F, etc.) to keep the table readable—it already is freighted with multiple compounds—preventing us from showing exact position of the modified atom. However, the full structures (including SMILES) for all parents and analogs are provided in **Supplementary Table 1**, which we now record in the Table notes, "Full chemical structures (including SMILES) for all parent compounds and analogs are provided in **SI Table S1**."

2.4.6. *When estimating the random background, the authors arbitrarily set a threshold at 10-fold improvement (of affinity or potency? it remains unclear—refer to my major concern). It is unclear why the authors have decided to include 2 analogs that were below the threshold. Although they are indeed close to the cut-off, if included the authors needs the present a different narrative about their selection method or exclude the 2 values altogether. Of note, this change would only reduce the estimated random background down to 13.6%.*

This is fair. In the manuscript tables, fold-changes are displayed to the nearest-integer with the unrounded values reported in **SI Table S1**. We applied the same rounding convention when processing the ChEMBL-derived data to ensure consistency. We now write in **Table 1** notes that "Fold-changes are displayed to the nearest-integer; exact (unrounded) fold-changes are provided in **SI Table S1**. Using exact fold-changes, N = 62 analogs are ≥3-fold improved and 25 improve ≥10-fold." In free energy, the difference between 10 and 9.7 is less than 0.02 kcal/mol. We now write in the main text:

> "We note that 4 of these 29 had fold-changes of between 9.7 and 9.9 to 1 decimal place; we consider these effectively 10-fold improvements and used the same convention in analyzing the public ChEMBL data (see **Table 1** and **Supplementary Table S1** for details)" (p.8)

2.4.7. (line 208). (*SI Table 2): do the authors mean Ext. Data Table 2? If so, this table is about PK properties, not cryo-EM structure resolutions.* We are indeed referring to **Supplementary Table 2,** which gives cryo-EM statistics and is the appropriate Table to call.

2.4.8. *Ext. Data Table 2 is missing the information about mOR and the 16 analogs generated*... This Table summarizes in vitro PK properties, and the MOR series was not profiled for PK, and so are not included. We have added a note to the table legend to make this explicit.

2.4.9. *Fig. 6b: The authors mentioned the higher potency of 4905 compared to 3629 in a cAMP assay and suggest that the second phase of 4905 is due to a low affinity Gs coupling. Have the authors confirmed the Gs coupling of 4905 …? If so, and the position of 4905 and 3629 are almost identical in the binding site, how can 4905 produce a specific Gs response that is not observed with 3629? Could the authors provide a scientific explanation?*

There is precedence for α2a agonists engaging Gs as a secondary G protein, so this idea seemed plausible. To test directly whether 4905 does engage Gs, we performed additional

cAMP assays in the presence of pertussis toxin (PTX), which inhibits Gi/o revealing any secondary coupling. Under PTX, 3629 no longer produced a detectable cAMP response, while 4905 retained a residual, concentration-dependent increase in cAMP, consistent with activation of Gs (now **Supplementary Figure 6**, and shown below, left panel).

The cryo-EM structure of '4905/α2a does suggest an explanation for why the analog '4905, but not its parent '3629, can engage Gs despite their similar poses. The added methyl in '4905 fills a hydrophobic sub-pocket and induces an outward rotation of Y409$^{6.55}$ (**Supplementary Figure 6**). This Tyr is important for Gs activation (DOI: 10.1016/j.celrep.2019.10.088). We propose that the methyl-induced shift of Y409$^{6.55}$ stabilized receptor conformations more favorable for Gs coupling. Naturally, this remains a hypothesis, but the PTX experiments and structural differences together support a ligand-specific G-protein engagement for '4905 that is not observed with 3629. We have added an explanation in the main text:

> "From the cAMP dose–response curves, we observed an upward deflection at higher concentrations for '4905. Consistent with this, PTX-treated cAMP assays showed a residual, concentration-dependent increase in cAMP for 4905 but not 3629 (**Supplementary Figure 6**), suggesting secondary Gs coupling by 4905. Structurally, the extra methyl in 4905 fills a hydrophobic sub-pocket and shifts Y409$^{6.55}$ (implicated in Gs activation), which may bias the receptor toward conformations permissive for Gs engagement."

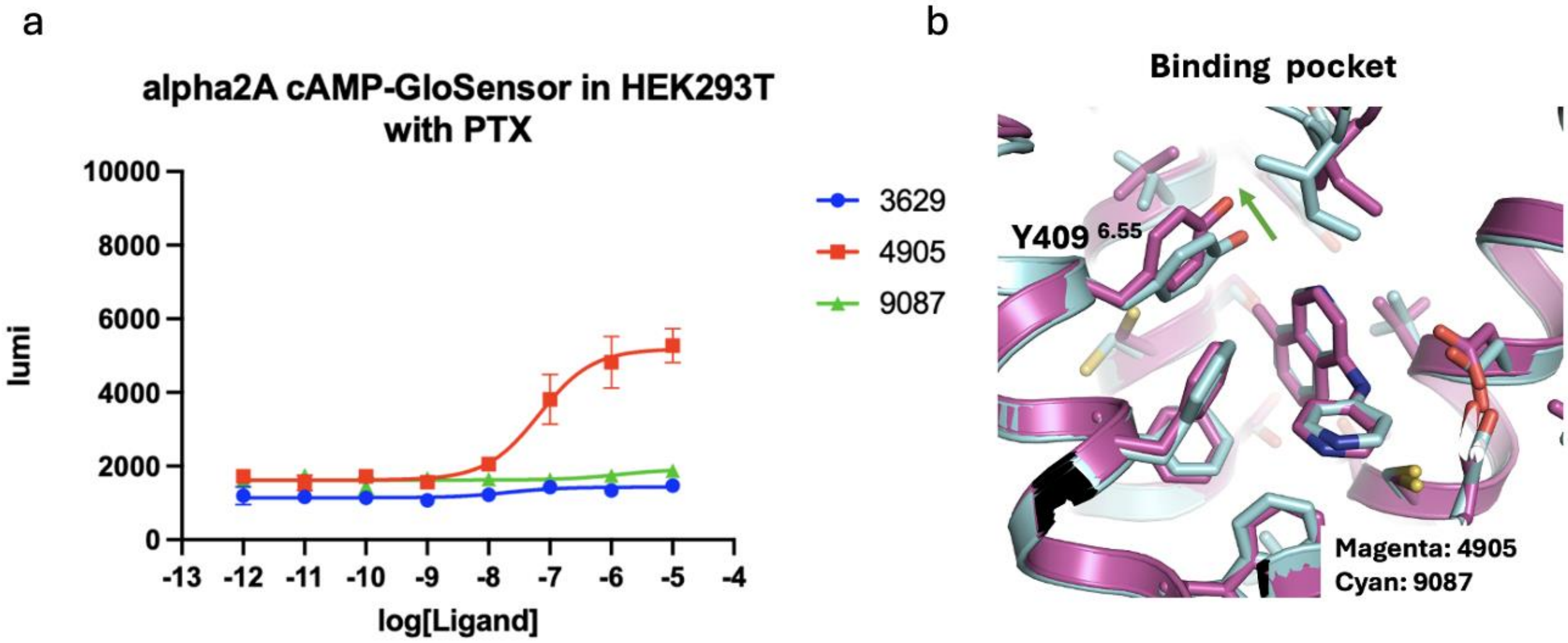

Supplementary figure 6 PTX-treated cAMP responses and a structural rationale for ligand-specific Gs coupling by '4905. **a**. Pertussis toxin (PTX)-treated α2A cAMP-GloSensor concentration–response curves in HEK293T cells for '3629, '4905, and '9087. A residual, concentration-dependent increase in cAMP is observed for 4905 at higher concentrations, whereas '3629 and '9087 remain flat under PTX treatment. **b**. Overlay of the α2A binding pocket comparing complexes for '4905 and the related '9087 (magenta, 4905; cyan, 9087), highlighting an outward shift of Y409^6.55 associated with the additional methyl group in 4905.

Reviewer 3:

Reviewer 3 seemed more critical, though they too found kind things to write about this study, for which we thank them. Four main concerns stood out: **a**. How the activity changes we observe relate to modern ideas of ligand efficiency and lipophilic ligand efficiency; **b**. How statistically powered this study is, **c**. Requests for confidence values on assay measurements, and **d**. Whether we shed new light on drug optimization trends and strategies. We take their concerns in order.

3.1. *(The) paper suffers from lack of insight and discussion of well-established concepts in medicinal chemistry, primarily that increasing logP through C-Cl and C-Me substitution leads to improvements in potency at the expense of pharmacokinetic properties primarily through the hydrophobic effect. The changes in key physicochemical properties such as logP of these analogs is not discussed, despite the fact that numerous studies looking at ligand optimisation in the context of physiochemical properties have been published, establishing commonly used metrics such as ligand efficiency (LE) and lipophilic efficiency (LipE)…(e.g.) Miller et al., J. Med. Chem., 2020, 63, 12156-12170.*

This is well-taken: considering ligand efficiency (LE) and lipophilic efficiency (LipE) allows one to distinguish substantial increases from more trivial ones that can be laid at the door of hydrophobicity and come with physical property liabilities. Encouragingly, analogs that improved activity even ≥3-fold had 0.65 kcal/mol/atom improvements in LE (and meaningful improvements for the overall molecule) and analogs that improved activity ≥10-fold were at what is considered the efficiency limit for a single atom change. Similarly, the effects on LipE were typically far larger than expected for hydrophobicity alone. We now show this in a new supporting table, which reports the per-atom changed/added and per-molecule LE and LipE effects for the analogs that improve ≥10-fold. We also now include new **Ext. Data Figure 4** that shows the how affinity changes correlate with cLogP, LE and LipE (reproduced below). As can be seen, whereas there is a correlation between activity improvement and hydrophobicity, it is weak, with an R^2 of only 0.1. Meanwhile, the correlation between LE and change in activity is strong, with an R^2 above 0.9—without fail, molecules that have activity improved ≥3-fold have strongly improved LEs. While the spread with LipE is slightly higher, the correlation between it and affinity changes is also strong, with R^2 above 0.8.

We have added a paragraph addressing this and now also cite Miller, 2020. We write (p.9):

> Because increases in hydrophobicity will increase affinity simply by disfavoring the unbound state and come with physical property liabilities, it is useful to consider the improved affinity of the analogs in light of ligand efficiency (LE) and liphophilic efficiency (LiPe), which control for these effects. If affinity improvements were largely driven by hydrophobicity, we would expect to see both terms deteriorate for the analogs. Instead, for analogs that improved >10-fold both ligand efficiency and lipophilic efficiency much improved. For instance, an analog with an added methyl, chloro or other atom that improved in binding or in EC_{50} by even 3-fold enjoyed a ligand efficiency of 0.65 kcal/atom for the added atom, well above the 0.3 kcal/atom that is thought to be useful for drug optimization[29,30]**.** Meanwhile, analogs that improved activity >10-fold had ligand efficiency of >1.4 for a single added atom (Ext Data Figure 4) close to the efficiency limit [31] (several exceeded single atom ligand efficiencies of 2.0, which is rare indeed). Similarly, every analog that improved activity by >10-fold also improved lipophilic efficiency (Ext. Data Figure 4d). More broadly, while there was little correlation between ΔclogP and ΔpKi or $\Delta logEC_{50}$ over the full range of the 257 analogs investigated here (**Ext Data Figure 4b**), there was a strong linear correlation between both ligand efficiency and lipophilic ligand efficiency versus activity change—in both cases, substantially improved activity was accompanied by improved ligand and lipophilic efficiency. For analogs with such improved activity—especially those improved by ≥10-fold but also for those improved by ≥3-fold—the improvements cannot be simply laid at the door of increased hydrophobicity.

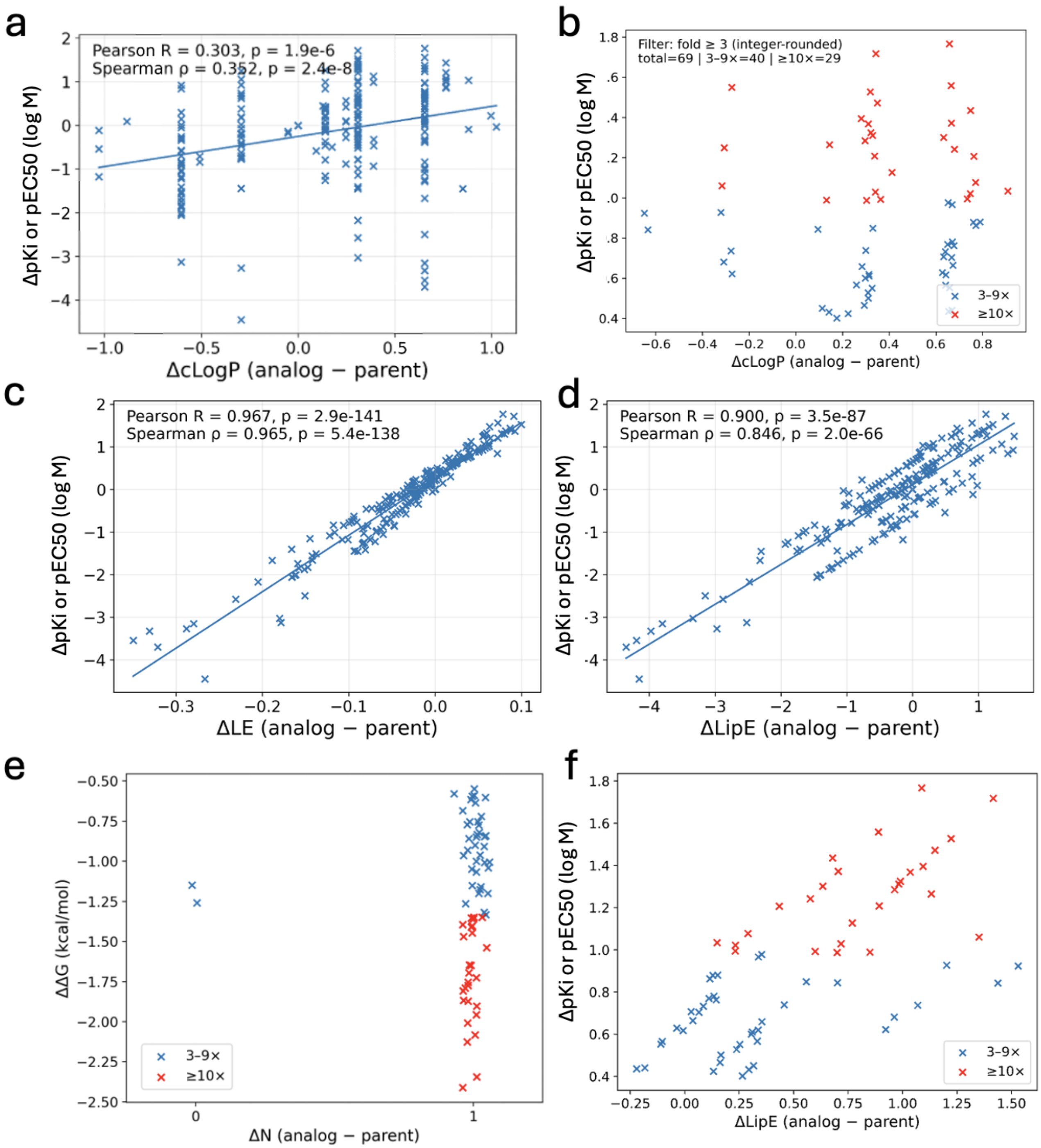

Extended Data Fig. 4 Correlations between physicochemical descriptors and ΔpKi/pEC50 (log M). **a**. Scatter plot of ΔpKi or pEC50 (log M) versus cLogP (RDKit). Each point represents one analog; the solid line indicates a linear fit. Pearson's R and Spearman's ρ are shown. **b**. ΔpKi or pEC_{50} (analog − parent) against ΔcLogP for all analog–parent pairs with ≥3× rounded potency/affinity improvement; ≥10× improvements are highlighted in red. **c.** Scatter plot of ΔpKi or pEC50 (log M) versus ΔLE (analog − parent), where LE = 1.37 × pKi or pEC50 / Nheavy. Pearson's R and Spearman's ρ are shown. **d.** Scatter plot of ΔpKi or pEC50 (log M) versus ΔLipE (analog − parent), where LipE = pKi or pEC50 − cLogP. Pearson's R and Spearman's ρ are shown. **e**. Binding free-energy change versus heavy-atom change for improved analogs. The y-axis shows ΔΔG (kcal/mol), computed from the affinity metric (Ki/IC50/EC50) as ΔG = RT ln(K) (298 K) and ΔΔG = ΔG_analog − ΔG_parent; negative ΔΔG indicates improved binding. The x-axis is ΔN (analog − parent; heavy-atom count difference). Points with rounded fold ≥10 are highlighted in red; rounded 3–9 are shown in blue. **f**. Affinity/potency gains versus changes in lipophilic efficiency. Scatter plot of ΔpKi or pEC50 (analog − parent) against ΔLipE (analog − parent) for all analog–parent pairs with ≥3× rounded potency/affinity improvement; ≥10× improvements are highlighted in red.

3.2. *The analysis of the structural bases for the affinity changes is comprehensive and well explained but also suffers from a similar lack of new insight into protein-ligand interactions. C-F is a well-known substitution for C-H (Meanwell, J. Med. Chem., 2018, 61, 5822-5880) and so causes minimal structural perturbation. Similarly, lipophilic substituents (C-Cl, C-Me) will make favourable interactions with hydrophobic side chains and when solvent exposed will lead to little change in potency (Freire, Drug Disc. Today, 2008, 13, 869-874). The observation that FEP+ performs better with experimental data is also well known (Schindler et al., J. Chem. Inf. Model., 2020, 60, 5457-5474).*

At the risk of pandering, we agree with the Reviewer—our initial analysis of the structural results lacked insight. It's a shame because it squanders an unusual coupling of structural analyses with this med chem-driven paper: 28 structures were determined, including 27 to ultra-high resolution (0.97 and 1.02 Å), and several of the results were surprising. While many of the structures explained the affinity changes in retrospect, many could not have been easily predicted, sometimes even by FEP. This perhaps supports the systematic small perturbation strategy, though establishing such a strategy was not our goal. We now write (p.15):

> If many of the effects of the small perturbation analogs could be explained *post hoc* from their structures, however, fewer were easily anticipated. Even the apparent simplicity of the '3453 series is belied by structural accommodations in the enzyme site. For instance, the para-chloro of the 2μM '6343 would have clashed with the enzyme without Phe132 and Ile131 rotating away. In doing so, Phe132 adopts a partially eclipsed conformation. Presumably the resulting strain is overcome by the improved packing of the buried para-chloro, but the outcome of this balance of terms seems difficult to intuit (encouragingly, free energy calculations did correctly predict that the para-and meta-chloro '6343 and '1304, respectively, would improve affinity while the ortho-chloro '0676 would lose it, though the agreement was more qualitative than quantitative; see below). Conversely, the methyl analog ('9249, IC_{50} 53 μM) of '0676 (IC_{50} 22 μM) is buried from solvent, not exposed, and might be expected to be more potent than its chloro cousin, but instead it loses another 2.5-fold in K_i (p-value <0.01; here, the opposite effect was anticipated by the free energy calculations, below). Meanwhile, in the complex between '4905 and the α2a adrenergic receptor, an added methyl group fits into a pre-existing sub-pocket and qualitatively its improved potency is reasonable. Quantitatively, its 52-fold effect on EC_{50} is at the outer edge of what might be expected by ligand-efficiency, especially since the methyl buries a serine hydroxyl. This is echoed by the free energy calculation, which here too correctly predicts improved activity, but only by 2- not 52-fold. These are examples of complexes where the analogs largely superpose on the parent and each other. In complexes where the single-atom changes led to substantial inhibitor movement, or to the adoption of multiple ligand conformations, prediction was harder still (**Extended Data Fig 5**). For example, the Mac1 methyl analog '3176 repositions the inhibitors in the pocket (**Extended Data Fig. 5b**), while compounds '9249 and '3194 adopt two distinct conformations in the site (**Extended Data Fig. 5b**), a phenomenon that is likely underreported in the PDB[34] but is readily seen at ultra-high resolution. Taken together, this set of perturbations may provide an interesting and sometimes challenging test for prediction methods and suggests that there may be merit in systematic small perturbations as a strategy, though showing that was not a goal of this study.

3.3. *The paper concludes by presenting an example of improving a parent compound (3629 -> 4905) where the increase in potency is not offset by a decrease in PK performance to the same degree. This example highlights the whole reason drug discovery is possible yet so challenging…Although this is a nice, well explored example of how single atom changes can*

be important it is not a novel concept or finding and has been described extensively. See for example Young et al., J. Med. Chem., 2018, 61, 6421-6467 (ref 34 in manuscript).

We agree that these trade-offs will be familiar to those versed in the art, and that the Young & Leeson paper—a classic that we teach at UCSF—well-captures them, and the importance of being careful about ligand efficiency and LipE (or LLE as in Young). We also agree that we could have done more to give this drug discovery background in the manuscript and we now add text to do so (see below).

Still, perhaps the Reviewer will agree that including this example adds to the impact of this manuscript. It takes the reader through a specific example of how the trade-offs play out up in vivo, including in vitro EC_{50} improvement, an atomic resolution structure showing where that improvement comes from, in vivo and in vitro pharmacokinetics illustrating the trade-offs, and finally an in vivo efficacy experiment that says just what the Reviewer writes (and also Reviewer 1)—multiparameter optimization is after all possible in drug discovery, especially when one is given as much freedom-to-operate as a 52-fold improvement affords. To better contextualize this result with current drug discovery principles, we now write (p. 22),

> "If ligand optimization is a frustrated landscape, it remains a navigable one, as is well-known to medicinal chemistry[41]. In addition to beginning to provide a random background for such efforts, this study supports a strategy by which that landscape may be reconnoitered[21,24] ."

3.4. "*The study would benefit from a statistical analysis and discussion of what number of molecules would be sufficient to establish a random background and why the background isn't being evaluated per parent; although over 250 molecules are made, this is across a large series of parent compounds. The number of analogs per parent is actually quite low when compared to the number that could be evaluated based on the authors' criteria, and seemingly below the threshold to establish a truly random sample."*

This critique is well-taken. We now explicitly address how many molecules are needed to define a useful "random background" and why our main analysis is performed at the global rather than per-parent level. Specifically, we treat each analog as a Bernoulli trial with respect to a given threshold (e.g. ≥10-fold change in the activity readout) and report confidence intervals for the estimated frequency as a function of sample size (new **SI Figure S1**, below). This analysis shows that, at the aggregate level of >250 analogs, the estimated background probability is well-constrained whereas per-parent sample sizes are often too small to yield informative confidence intervals. For this reason, we emphasize the global background in the main text and provide per-parent summaries in the Supplementary Information. We write (p.7):

> Importantly, the random background we're establishing answers the question: for an arbitrary hit molecule at an arbitrary target, what are the odds that a random close analog, selected without constraints other than synthetic accessibility, improves potency $\geq$10x? With a pooled sample size of ~200 analogs, the estimated ≥10× success frequency is constrained to within roughly ±5 percentage points (95% binomial CI), whereas typical per-parent sample sizes (often <20–30) yield confidence intervals too wide to be informative. For this reason, all 257 analogs should be pooled together for analysis, cancelling the noise from a single scaffold or single target (**Supplementary Figure 1**).

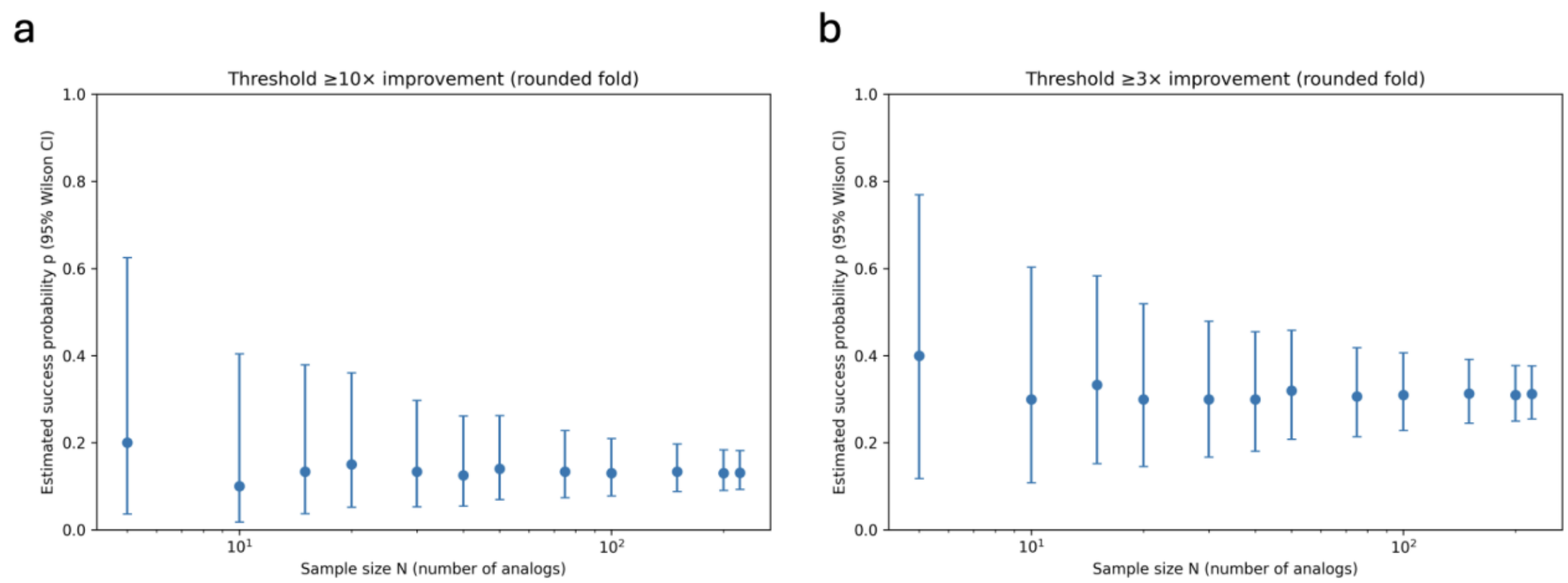

Supplementary figure 1. Sample size required to constrain the "random background" success rate. Each analog was treated as a Bernoulli trial for a threshold outcome (success = integer-rounded fold improvement ≥10× in panel a; ≥3× in panel b). Points show the estimated success frequency (p^=k/N) at each sample size N; error bars indicate two-sided 95% binomial confidence intervals computed using the Wilson score method. As N increases, the confidence intervals narrow, showing that pooled analyses with large N provide a well-constrained estimate of the background probability, whereas per-parent sample sizes (typically much smaller) yield wide and often uninformative intervals.

3.5. "*Although systematic, the analogs made seem hardly unbiased and H, F, Cl, Me, OH, N are pretty standard SAR changes and not random at all. I question the claim that the dataset in ChEMBL is "biased" more than I would question that this study is "unbiased." Both datasets suffer from the same bias of organic chemistry in that there's not a huge number of single atom substitutions you can put on or substitute C for that are chemically stable and synthesizable.*

We have added caveats to our introduction of a random background (p.5):

> The single atom modifications involved substituting carbon hydrogens with one of methyl25, hydroxyl[26], chloro[27], fluoro[28], or rarely bromo, or substituting aromatic carbon atoms with nitrogens (Figure 1). These changes are not comprehensive but they do represent changes preferred by medicinal chemists while avoiding those that would introduce oxidation liabilities (e.g., adding a thiol or aromatic sulfur) or changes of valence (e.g., nitrogen to oxygen or sulfur); moreover, they were made without design and as systematically as pragmatism would allow.

That admitted, this is one place where we might partly differ with the Reviewer. These are standard changes, but that's a feature, and they were made without design, systematically. In the manuscript we compare them to the random backgrounds used in biology, including alanine scanning. Here, a single amino acid is chosen to represent "random", but since it is always the same choice it is considered unbiased. We argue the same here.

Apropos of ChEMBL being more biased, here too perhaps reasonable scientists may differ reasonably. We *suspect* that investigators are more likely to report analogs that improve affinity rather than reduce it, but of course that's just our opinion. We now note this, writing, "Intriguingly, the likelihood of improving a parent in the random set is comparable to what is observed among these ChEMBL matched pairs (Figure 2c), despite what ***may be*** reporting biases towards potency in the latter" (emphasis added).

More quantitatively, there are 54,021 parents and 79,984 small-change analogs in ChEMBL (about 1.5 analogs per parent) and only 4.7% of parents have more than 3 analogs. Our study is 10-fold more comprehensive by number of analogs/parent and more systematic. We argue that it's a meaningful start that, despite its limitations, illuminates an important problem.

3.6. *… for a manuscript which endeavours to establish a link between molecular changes and the chance of changes in potency and ADME properties, errors in fundamental measurements and error propagation … used in the headline analyses are notably lacking. For a majority of analogs, affinities have been derived mainly from IC_{50} values using the Cheng-Prusoff equation. The Z' values for the assays used to determine EC_{50}s are not reported, nor are any errors for the IC_{50} values or derived Ki's. It is therefore difficult to accept many of the claims of affinity or ADME differences, particularly those below ten-fold without a more rigorous statistical comparison. … For example on line 224 the authors rationalise a lower potency of a para-fluoro analogue (5 uM) compared to a para-chloro analogue (2 uM) which is not supported without knowledge of error in these IC50 values.*

We agree that improved statistics will strengthen the manuscript. We now report error ranges for all measured values, including K_i, EC_{50} and IC_{50} in **Supplementary Table S1**, and ADME properties in **Supplementary Tables 3** to **8**. All dose–response curves are now provided in the **SI Figure 2**. For instance, in the example highlighted by the Reviewer, '6404 has an IC_{50} of 5.5 μM (95% CI: 5.01–6.11 μM), while 6343's is 2.1 μM (95% CI: 1.8–2.4 μM), supporting a statistically meaningful difference.

To address the *Z' values* concern, we include the example of concentration–response curves together with their corresponding Z values in **SI Figure 3**. For completeness, the full set of dose–response curves for all analogs is shown in **SI Figure2**. We have added the following to the main text "Representative concentration–response curves and the corresponding Z values for each assay are provided in Supplementary Figure 3."

a

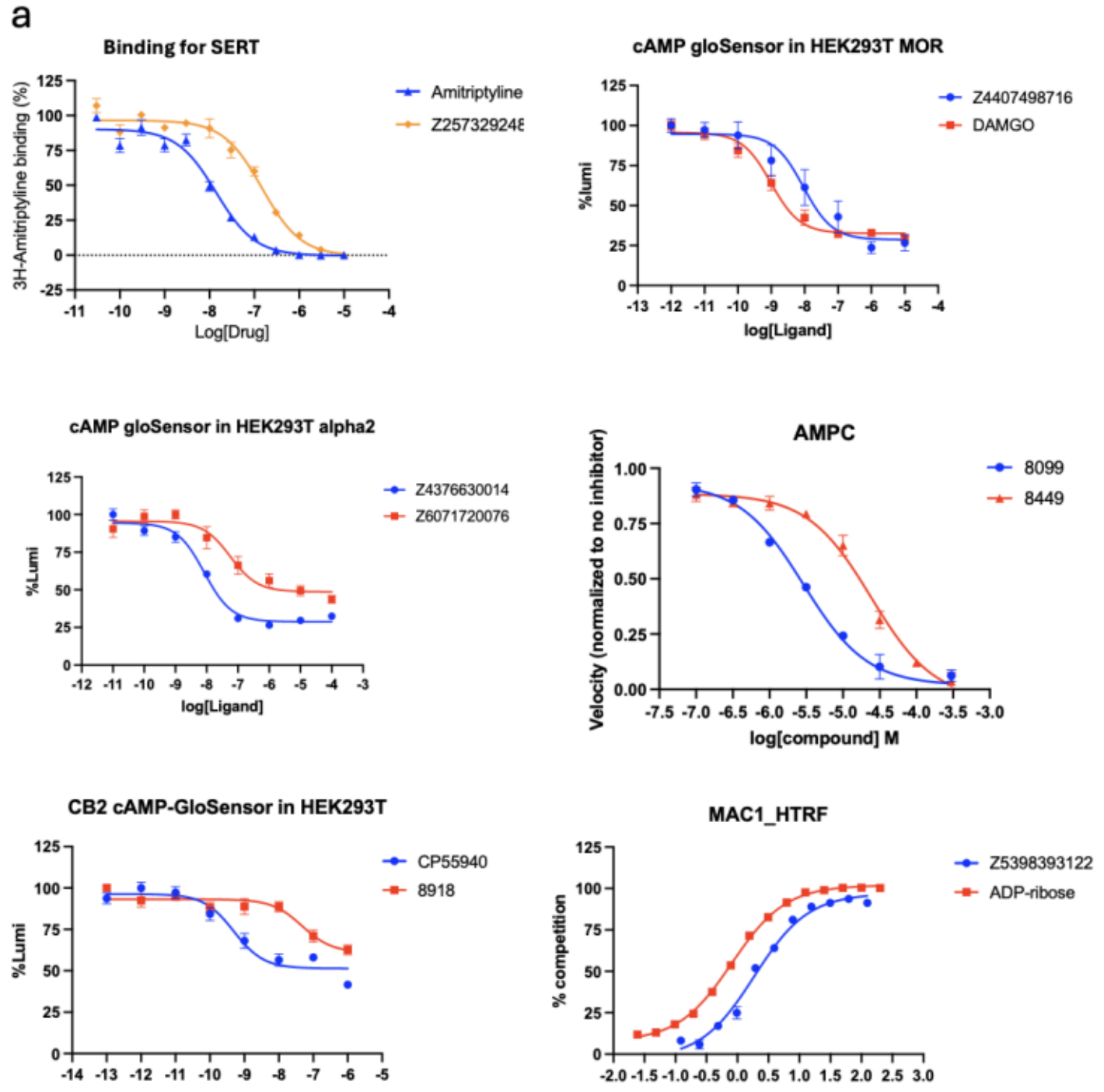

b

Target	Readout	Parent	Z-value
SERT	Ki	Z2573292480, Z2009978218, Z2573292509, Z6971277399, Z8727393896, Z8731642686	0.658
MOR	EC50	Z4407498716	0.5728
alpha2	EC50	Z2750653629, Z4376630014	0.409
alpha2	Ki	Z3034773248, Z8727395870	0.787
AmpC	Ki	Z2610488449	0.493
cb2	Ki	Z52076138, Z6969215903	0.576
cb2	EC50	Z8184698918	0.571
Mac1	IC50	Z5398393122, Z1039063794, Z7534253453	0.880

Supplementary Figure 3. **Representative concentration–response curves and Z-values. a.** Representative concentration–response curves for assays used in this study (SERT, MOR, α2a, CB2, AmpC, and Mac1). Corresponding Z-values and the parent compounds used for each target/readout. Full concentration–response curves for all analogs are shown in Supplementary Figure 2.

3.7. *As discussed above, many of the smaller affinity differences (less than ten-fold) are likely not differences at all, rendering many of the conclusions and rationalizations questionable.*

That being said, the observations around structural changes that lead to greater than ten-fold changes in affinity are supported by many well-established concepts in medicinal chemistry, along with the effects of these changes on pharmacokinetic properties.

We empathize with the Reviewer's critique—small changes can be over-interpreted in medicinal chemistry, one reason we focused on ≥10-fold changes in the text. In our experience, measurements supported by full concentration response in well-behaved assays (**Supplementary Figure 2**) typically *do* support meaningful differences of ≥3-fold. Accordingly, we now report 95% confidence intervals for Ki/EC50/IC50 values and assess significance on the $\log_{10}$ scale using a two-sided Wald/z test, with standard errors derived from the reported confidence intervals. When we restrict the analysis to improvements (parent-to-analog) and round fold-changes to the nearest integer, 66 of 71 (93%) ≥3× improvements are statistically significant ($p < 0.01$); moreover, all 31 of 31 ≥10× improvements are significant ($p < 0.01$). With the few exceptions noted in the tables, these differences are readily apparent by inspection of the curves (**Supplementary Figure 2**). We thank the Reviewer for insisting on this, as it has strengthened the manuscript.

3.8. *Z'-values for assays should be included as well as a discussion of the error in the derived affinity values. Examination of the LE and LipE of the analogs should be included as well as analysis of the correlation between logP and affinity improvements. The conclusions of the paper would only be of interest if they shed new light on drug optimisation trends or strategies.*

We have addressed the statistical concerns of the Reviewer and now put our results in context of ligand and lipophilic ligand efficiency, as discussed above. Whereas this study did not set out to develop a new strategy for ligand optimization, a simple strategy for doing so nevertheless emerges: systematic small perturbations can achieve large activity improvements in activity at an unexpectedly high rate.

3.9. *Young et al., J. Med. Chem., 2018, 61, 6421-6467 is cited (ref 34), along with other papers regarding ligand efficiency metrics, however only in the context of worsening PK properties with improvements in potency. The huge body of work on the strong links between physicochemical properties (especially lipophilicity) and their effect on the many axes of small molecule optimisation (potency, selectivity, toxicity, ADME, etc.) are not adequately referenced or considered in the manuscript.*

We regret these oversights and have added references to papers suggested by Reviewer 3 throughout their critique, as well as to those suggested by Reviewer 1, and have deepened our discussion of the science that they represent.

Referee #1:
The authors have addressed all my concerns thoroughly - many thanks! Derek Lowe

Referee #2:
I believe the authors have addressed my concerns. I am comfortable to agree for publication of this work.

Referee #3:
This revised manuscript is much improved. … I consider the manuscript acceptable for publication, subject to a few minor modifications and clarifications outlined below.

3.1 Page 5: "*18 parents cover a wide range of physico-chemical properties (Ext. Data Table 2)". I think they mean Fig. 2 and they should add physico-chem data (at least Rof5 properties) below compounds in the figure to display the range of physico-chem spread*.

Well-spotted, we *did* mean Ext Data Figure 2—this has been corrected. We have also added clogP values under the ligands; molecular weights are already presented in the Figure.

3.2 "suggesting also a systematic strategy for optimization, even though that was not our primary goal*." This sentence is a little unclear. Do they mean "suggesting also a strategy for optimization which includes making simple single-atom changes systematically, even though that was not our primary goal."?*
We now write, "As we will show, these undesigned changes had an unexpectedly high success rate, suggesting a systematic strategy for optimization, even though that was not our goal", hoping that it is clearer.

3.3. Page 7: *"3H-rauwolscine or [^{3}H]CP-55940" "[3H]-citalopram". Normalise radioligand nomenclature.* We have done so.

3.4. Page 10: "there was a strong linear correlation between both ligand efficiency and activity change and between lipophilic ligand efficiency and activity change—in both cases, substantially improved activity was accompanied by improved ligand and lipophilic efficiency." *… the graphs in Ext. Data Fig. 4 don't obviously support the observation that* "analogs that improved activity >10-fold had ligand efficiency of 1.4 for a single added atom (Ext. Data Fig. 4), close to the efficiency limit (several even exceeded single atom ligand efficiencies of 2.0). Similarly, every analog that improved activity by >10-fold also improved lipophilic efficiency (Ext. Data Fig.4)." *Can the data be presented differently to show this clearly*?

We think the Reviewer is suggesting that by calling Ext. Data Figure 4 immediately after we write, "for analogs that improved activity ≥10-fold the single atom LE was of ≥1.4 , close to the efficiency limit[31]", it makes it seem that that Figure supports this point, when the correspondence between activity and LE simply comes from the LE equation. Accordingly, we have cut the reference to Ext Data Figure 4 here (we refer to it several times in this paragraph, so no problem cutting it here).

To the Reviewer's second point, "*Can the data* (on improving lipophilic efficiency) *be presented differently…"* we have modified **Extended Data Figure 3a** to show the isoclines to which they refer in critique **3.5** (shown below), addressing both concerns**.** With the renumbering of the Extended Data figure set, the former Extended Data Figure 4a is now Extended Data Figure 3a.

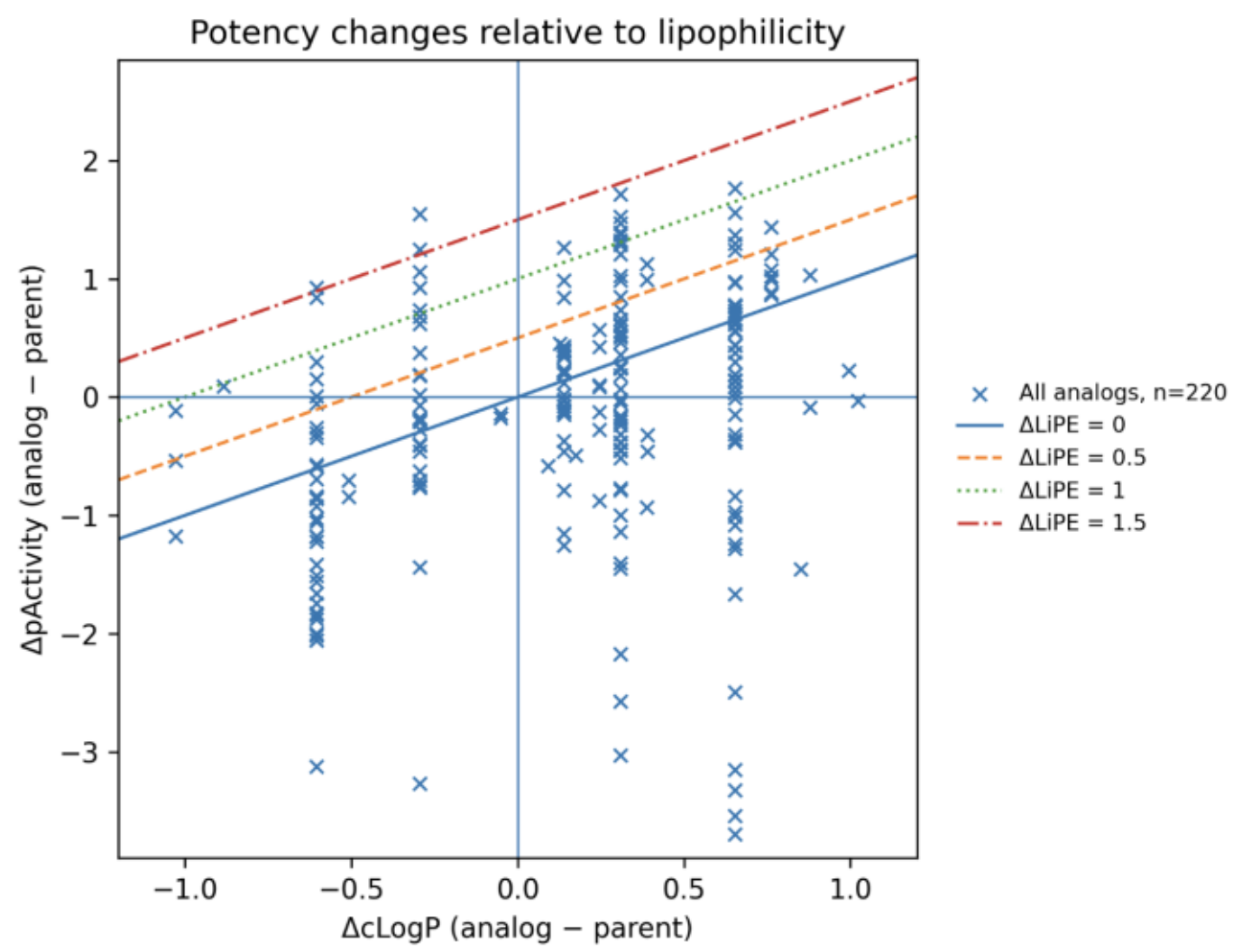

Revised **Extended Data Figure 3a** (renumbered from former Ext. Data Figure 4a).

3.5. *In addition, it is at least odd and probably meaningless to calculate a correlation between terms which are dependent on each other.* LE = -1.4pIC50/N; LiPE = pIC50 - clogP
It would be better to show improvement beyond easy clogP gains on LiPE by using LiPE isoclines as shown on … https://en.wikipedia.org/wiki/Lipophilic_efficiency and something similar for LE.

We have done so, shown above. Following the reduction and renumbering of the Extended Data figure set, this result is now shown in Extended Data Figure 3a.

3.6. Page 13: “If many of the effects of the small perturbation analogs could be explained post hoc from their structures, however, fewer were easily anticipated.” *Remove “If”.* Well taken. We have done so and revised the sentence to read, “Many of the effects of the small perturbation analogs could be explained post hoc from their structures; however, fewer were easily anticipated.”

3.7. Page 14: “Meanwhile, in the complex between ‘4905 and the a2a adrenergic receptor, an added methyl group fits into a pre-existing sub-pocket and qualitatively its improved potency is reasonable. Quantitatively, its 52-fold effect on EC50 is at the outer edge of what might be expected by ligand-efficiency, especially since the methyl buries a serine hydroxyl. This is echoed by the free energy calculation, which here too correctly predicts improved activity, but only by 2- not 52-fold.” *This is a really interesting observation and shows limitations of SBDD and FEP. Why no Ext. Data figure for this case?*

We share the Reviewer’s enthusiasm for how interesting this is! After careful review, we think the story is told well-enough in the main manuscript **Figure 5**, which is devoted to these data, and by our comments about it quoted by the Reviewer.

3.7. Page 15: “suggests that there may be merit in systematic small perturbations as a strategy, though showing that was not a goal of this study.” *This is repeated near verbatim on p. 5 and I suggest removing here*. This is well-taken, and we have removed it.

3.8. Page 16: *Why no mention of solubility and hERG in main text even though it’s been measured for 10-fold compound set in Ext. Data Fig. 7?*

We now mention both terms on this page, admittedly briefly. In a longer paper we would dwell on them more, but given the space constraints of the journal, we focused on the three most important and guiding PK terms: fraction unbound, permeability, and metabolic stability.

3.9. Page 17: "This is born out on an atom-by-atom level: those atoms that most often improved affinity/potency substantially, like methyls and chloros (Figure 2a)," *This is very pedantic but "moieties" or "groups" better than "atoms" as methyl is not an atom. Check for other similar uses throughout the text.*

We agree and have replaced "atoms" with "groups", "substituents" or "moieties" where appropriate, including the sentence identified by the Reviewer.

3.10. Page 20: "experimental (grey) poses…" *Looks more light blue than grey. Protein is grey.*
We have corrected the Figure 4 legend to describe the experimental pose as light blue; the protein is shown in grey.

3.11. Page 24: "The individual in vitro pharmacokinetic terms were orthogonal to each other, and several were anti-correlated with affinity/potency improvement (Fig. 4)." *It would be worth expanding here with a sentence and references on the known correlations between hydrophobicity and desired properties (target potency (e.g. LiPE)/permeability), and undesired properties (mic and plasma instability/low solubility/off-target potency (hERG)/low Fu) as this dichotomy is the fundamental challenge in lead optimisation.*

We take the Reviewer's point. We note that this paragraph ends with the sentence, "While this trade-off has been noted in medicinal chemistry,[40,41] this set quantifies its systematic impact." We strengthen this further by now writing that "this trade-off has been ***well***-noted…" and soften "this set quantifies" to "this set can quantify…" We already reference refs 40 and 41, the Hopkins 2014 paper on ligand efficiency and the Leeson 2018 paper on optimization in drug discovery.

3.12. *DISCUSSION I think it would be worth adding a few sentences on the limitations of docking and SBDD. The systematic approach in this study clearly highlights little correlation between structure, high level calculations, and potency and even some odd things like the ligand completely changing orientation with little effect on potency (Z8990523176 in Mac1). Summarising these examples and findings in the discussion will be a sobering fact for the cohort of AI ligand design groups who claim to be able to routinely use ML methods in ligand optimisation*.

While the limitations of docking and structure-based drug design are well-known to us, this is not a theme of this study; docking had no role whatsoever, design was specifically avoided, and so we worry this would distract from the study's key messages. Begging your indulgence here, we have not met the Reviewer's request.
